# Synthesis of Heterocycle-Substituted Bicyclo[3.1.1]heptanes and Aza-bicyclo[3.1.1]heptanes via Photocatalytic Minisci Reaction

**DOI:** 10.1021/acs.orglett.3c03684

**Published:** 2024-01-22

**Authors:** Rebecca I. Revie, Benjamin J. Whitaker, Bhaskar Paul, Russell C. Smith, Edward A. Anderson

**Affiliations:** † Department of Chemistry, Chemistry Research Laboratory, 12 Mansfield Road, Oxford OX1 3TA, United Kingdom; ‡ Drug Discovery Science and Technology (DDST), AbbVie, North Chicago, Illinois 60064, United States

## Abstract

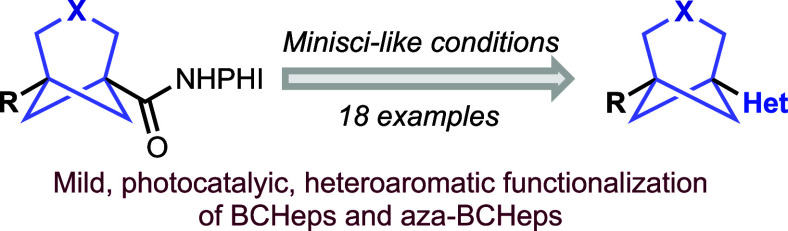

A route toward heterocycle-functionalized bicyclo[3.1.1]heptanes (BCHeps) and aza-bicyclo[3.1.1]heptanes (aza-BCHeps) has been developed, using mild, photocatalytic Minisci-like conditions to introduce various heterocycles at the bridgehead position from readily available *N-*hydroxyphthalimide esters of the corresponding carboxylic acids. This chemistry enables access to heterocycle-functionalized BCHep-containing structures that are highly relevant in medicinal chemistry research as potential bioisosteres of *meta-*substituted arenes and pyridines.

Bioisosterism is a valuable strategy in drug discovery for improving the performance of drug candidates.^1^ Bicyclo[3.1.1]heptanes (BCHeps, **1**, Figure 1a) have recently emerged as new bioisosteres for *meta*-substituted arenes,^2^ where replacement of such arenes with BCHeps in certain drugs has been shown to improve metabolic stability and lipophilicity compared to the parent arene-containing drug. BCHep-containing structures can be accessed through ring-opening reactions of [3.1.1]propellane,^2,3^ by analogy to the well-established preparation of bicyclo[1.1.1]pentanes from [1.1.1]propellane,^4^ and also by formal (3 + 2) cycloaddition strategies of bicyclo[1.1.0]butanes.^5^ However, some ring-opening reactions that are well-established for [1.1.1]propellane, such as anionic methods, do not translate well to [3.1.1]propellane, and thus the range of currently accessible functionalized BCHeps is not yet as extensive as that of bicyclopentanes (BCPs). In order for functionalized BCHeps to be more widely considered in drug design, more strategies for their synthesis must therefore be found, in particular approaches that enable the late-stage diversification of the bridgehead substituents.

**Figure 1:**
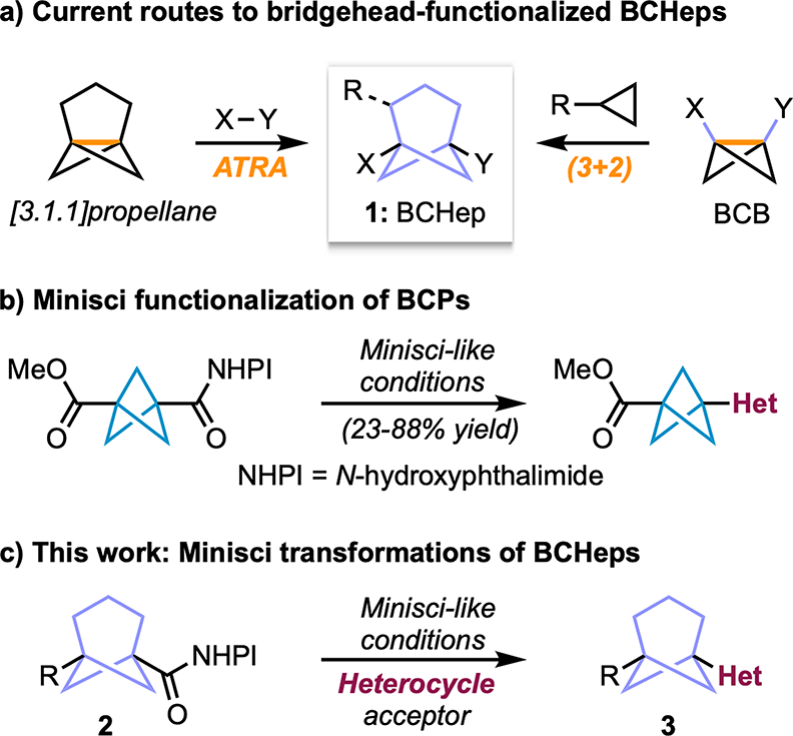
Routes to BCHeps and Minisci functionalization of redox-active bicyclo[*n*.1.1]alkyl bridgehead esters.

The mild photocatalyzed Minisci-like decarboxylative functionalization of aliphatic carboxylic acids, via the corresponding activated (hydroxyphthalimide) esters, was previously developed by Sherwood and co-workers.^6^ This reaction improves upon traditional Minisci conditions^7,8^ by avoiding the need for an external oxidant through use of a redox-active ester (RAE) and also employs an organic photocatalyst instead of an expensive metal catalyst.^9^ The use of these conditions was demonstrated on a variety of tertiary esters, including bridgehead bicyclo[2.2.2]octanes, and was later successfully applied to the synthesis of heterocycle-functionalized BCPs by Mousseau et al. (Figure 1b).^10^ BCHeps with ester-functionalized bridgehead positions can be readily prepared by double alkylation of cyclohexane 1,3-diesters with diiodomethane,^11^ and we questioned whether these convenient building blocks, which obviate the need for [3.1.1]propellane, could be converted to RAEs (**2**, Figure 1c) and then functionalized via the Sherwood–Minisci reaction (**3**). Here we report the realization of this goal, which increases the repertoire of strategies for the synthesis of BCHep-containing structures by introducing a new route toward heterocycle-functionalized BCHeps and aza-BCHeps.^12^

BCHep diester **4** (Scheme 1) was first prepared by esterification of commercially available cyclohexane-1,3-dicarboxylic acid followed by double alkylation in a one-pot procedure. The latter step streamlines the previously reported stepwise protocol,^11,13^ although the overall yield of **4** remained moderate. This BCHep diester was then desymmetrized by monoester hydrolysis (**5**), using barium hydroxide to minimize double hydrolysis.^14^ Finally, the carboxylic acid in **5** was activated and converted to the RAE **2a** upon reaction with *N*-hydroxyphthalimide.

**Scheme 1:**
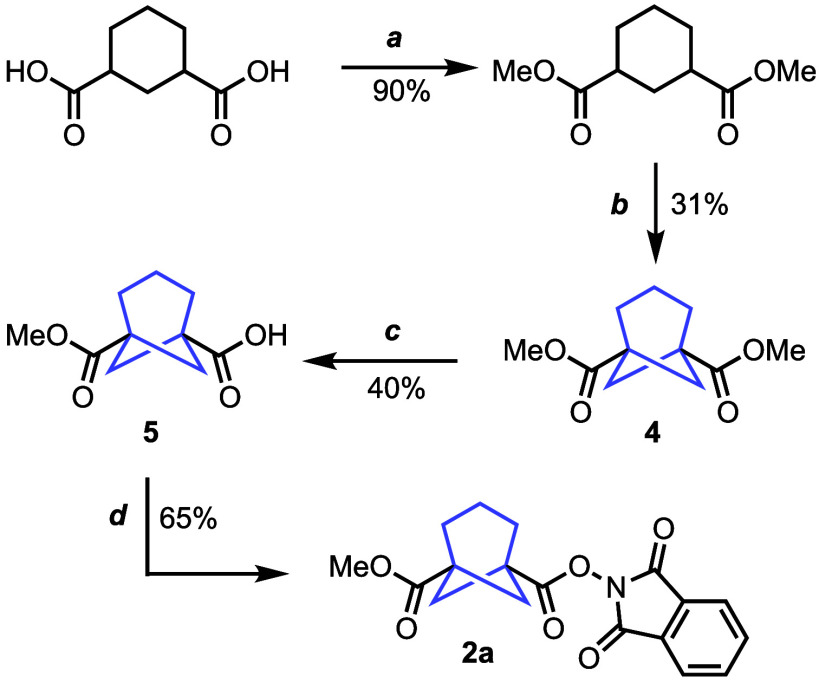
Preparation of the BCHep Redox-Active Ester (RAE) **2a** from Cyclohexane-1,3-dicarboxylic Acid Reaction conditions: (a) H_2_SO_4_, MeOH, 70 °C, 17 h; (b) LDA, DMPU, THF, −78 °C, 1 h; CH_2_I_2_, 0 °C to rt, 17 h; (c) Ba(OH)_2_, MeOH, H_2_O, 0 °C to rt, 20 h; (d) SOCl_2_, DMF, DMAP, *N-*hydroxyphthalimide, CH_2_Cl_2_ rt, 18 h.

The Minisci-like transformation was initially tested under conditions similar to those reported previously (Table 1, entry 1) using 4-methylquinoline as acceptor for the formation of BCHep adduct **3a**.^10^ Several polar solvents were screened (entries 2–6), with dimethylacetamide and DMF giving the highest yields. Various sulfonic acid activators also gave good yields (entries 7–9), but TFA was retained, as it gave the best yield and was deemed likely to display superior functional group tolerance. The additives NaI and PPh_3_ have been shown by Fu and co-workers to enable radical formation without a photocatalyst;^15^ however, these did not lead to an improvement in yield (entry 10), and it was found that a photocatalyst was still necessary in the presence of these ingredients (entry 11). Both 2,4,5,6-tetrakis(diphenylamino)isophthalonitrile (4DPAIPN)^16^ and 2,4,5,6-tetrakis(9*H*-carbazol-9-yl)isophthalonitrile (4CzIPN) photocatalysts performed well, and we were pleased to find that the catalyst loading could be reduced from 5 to 1 mol % without any significant decrease in yield, although a moderate reduction in reaction efficiency was observed at lower catalyst loadings (entries 12–19). The photocatalyst was confirmed as necessary for the reaction, since no transformation was observed in its absence (entry 20). The conditions highlighted in entry 17 offered the best yield with low catalyst loading and no extra additives. Pleasingly, the isolated yield of **3a** was maintained at 54% on a 1.0 mmol scale, compared to 60% on a 0.15 mmol scale (entry 21)

**Table 1:**
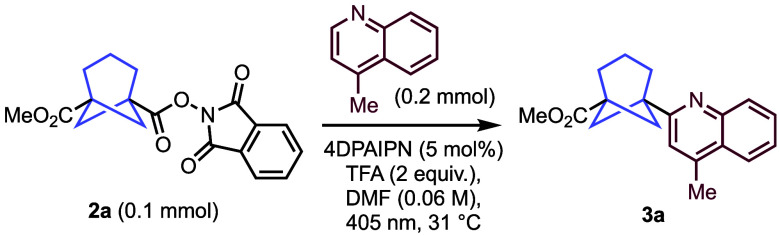
Reaction Optimization^a^

entry	deviation from conditions	yield (%)^b^
1	none	90
2	DMA instead of DMF	90
3	MeCN instead of DMF	48
4	^*t*^BuCN instead of DMF	76
5	DMSO instead of DMF	17
6	acetone instead of DMF	17
7	TfOH instead of TFA	81
8	TsOH instead of TFA	74
9	10-CSA instead of TFA	81
10	NaI (10 mol %) + PPh_3_ (20 mol %)	76
11	NaI (10 mol %) + PPh_3_ (20 mol %) no catalyst	5
12	4DPAIPN (2.5 mol %)	88
13	4DPAIPN (1 mol %)	88
14	4DPAIPN (0.5 mol %)	83
15	4DPAIPN (0.1 mol %)	76
16	4CzIPN (5 mol %)	88
17	4CzIPN (1 mol %)	88 (**60**)^c^
18	4CzIPN (0.5 mol %)	83
19	4CzIPN (0.1 mol %)	78
20	no catalyst	0
21	4CzIPN (1 mol %)	78 (**54**)^d^

a Reactions run on a 0.1 mmol scale of **2a** for 16 h.

b Yield determined by ^1^H NMR using DCE as an internal standard.

c Isolated yield in parentheses.

d Reaction conducted on a 1.0 mmol scale of **2a**.

With optimized conditions in hand, the scope of the reaction was investigated using a variety of radical acceptors and BCHeps (Figure 2). In the course of this work, we found that while in many cases excellent NMR yields were observed with complete consumption of the RAE, the BCHep products were often prone to degradation upon chromatography, leading to reduced isolated yields (shown in parentheses). In terms of scope, we found that a range of *N*-heterocycles gave moderate to good yields using **2a** as a substrate (Figure 2a), with isoquinoline derivatives (**3b** and **3c**) performing particularly well, while 4-methylpyridine and quinoxaline (**3d** and **3e**, respectively) proceeded in moderate yields. Minisci reactions commonly afford regioisomeric products with certain substrates,^7^ which rationalizes the formation of regioisomeric pyridines (**3f** and **3f′**) and bipyridines (**3g** and **3g**′), with substitution at the 2-position preferred in each case. This contrasts with the high selectivity observed with the unsubstituted isoquinoline **3c**, which reacted exclusively at just one of the two available *ortho-*positions, which is presumably due to the enhanced stability of the intermediate *N*-centered radical arising from reaction at the 1-position. Interestingly, dimethyl 2,6-pyridinedicarboxylate (**3h**) showed exclusive selectivity for the 3-position, with the electron withdrawing effect of the esters overturning the normal selectivity for the 4-position of the pyridine ring. Surprisingly, other heterocycles that performed well with BCP scaffolds^10^ proved unsuccessful in this BCHep reaction.^17^ We hypothesize that this could be due to either the increased steric hindrance of the BCHep bridgehead radical compared to the “tied-back” BCP radical or the increased *s* character of the latter, which renders it more stable and therefore selective for productive reaction.

**Figure 2:**
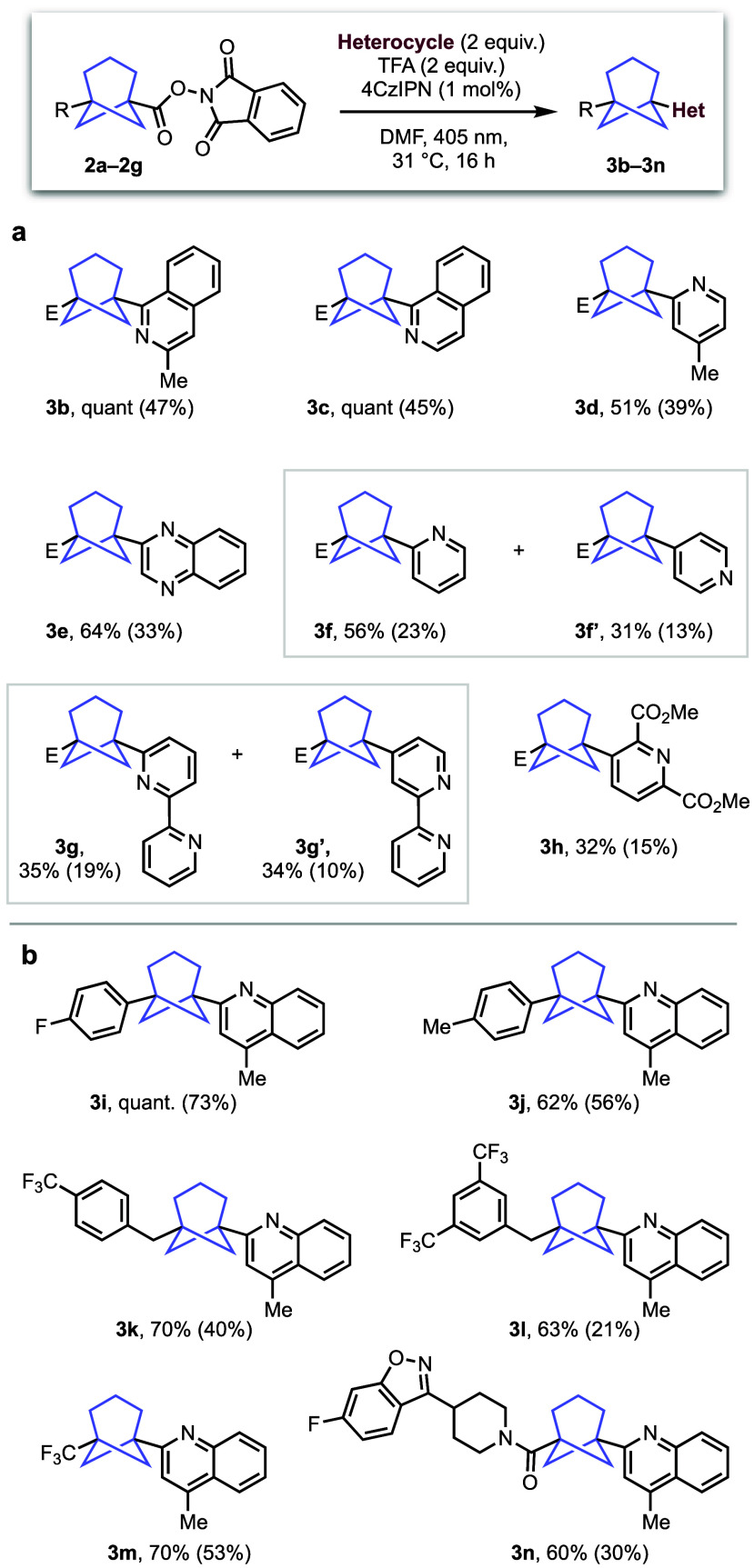
Scope of the Minisci transformation using bicyclo[3.1.1]heptane RAEs. E = CO_2_Me.

The scope of the Minisci reaction was next tested using BCHeps in which the substituent at the opposing bridgehead to the RAE was varied (Figure 2b). These starting materials were prepared from [3.1.1]propellane following adapted literature procedures.^2^ We were pleased to find that aromatic substituents with either electron-withdrawing (**3i**) or electron-donating (**3j**) properties performed well. Benzyl substituents were also tolerated, with **3k** and **3l** delivered in good yields. Pleasingly, the trifluoromethyl BCHep **3m** was also formed with high efficiency (70%), while use of a piperidine amide functionalized with a benzisoxazole motif also afford the Minisci product in respectable yield (**3n**).

With heterocycles successfully introduced onto the carbocyclic BCHep bridgehead, we questioned whether aza-bicyclo[3.1.1]heptanes, which have recently emerged as potential bioisosteres for 3,5-disubstituted pyridines,^12^ would also be compatible with this chemistry (Figure 3). To our delight, the aza-BCHep RAE **6a** underwent a successful Minisci reaction with several heterocycle acceptors, affording the corresponding bis-heterocyclic products **7a**–**7c** in good yields (a small amount of double addition was observed using **7c**). Aza-BCHep **6b**, featuring a bridgehead ester group, also proceeded smoothly in reaction with 4-methylquinoline to deliver adduct **7d**. This part of the work was carried out at AbbVie and represented a crucial element of this industry–academia collaboration, as we were able to take advantage of purification facilities not available in the Oxford laboratories.

**Figure 3:**
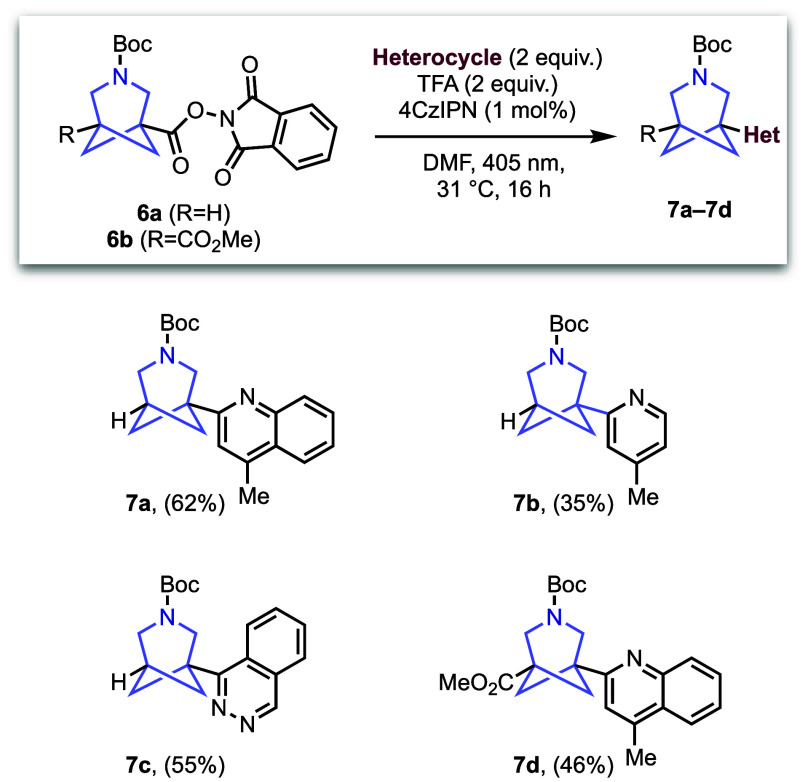
Scope of the Minisci transformation using aza-bicyclo[3.1.1]heptane RAEs.

In conclusion, a mild photocatalytic Minisci-like procedure was developed, allowing access to a variety of heterocycle-substituted BCHeps. Since heterocycles are some of the most common motifs found in drugs, this method is highly relevant for expanding the use of BCHeps in drug discovery and synthesis. While some products proved surprisingly sensitive to chromatographic purification, this chemistry nonetheless demonstrates the ability to form BCHep bridgehead radicals by decarboxylation, which we expect will lead to further opportunities for bioisostere synthesis. Notably, the link between Oxford and AbbVie proved crucial in enabling exploration of aza-BCHeps and optimization of the purification, underlining the value of academia–industry collaborations in the development of end-user-relevant synthetic methodology.

## Data Availability

The data underlying this study are available in the published article and its Supporting Information.

## Supporting Information Available

Experimental procedures, characterization data, and copies of ^1^H and ^13^C NMR spectra (PDF)
