# Supplementary material for: Synthesis of Heterocycle-Substituted Bicyclo[3.1.1]heptanes and Aza-bicyclo[3.1.1]heptanes via Photocatalytic Minisci Reaction
Source: Org Lett. 2024 Jan 22;26(14):2843–6. doi: 10.1021/acs.orglett.3c03684 (PMC11020156; doi:10.1021/acs.orglett.3c03684)
Supplement: Supplementary file 1 — ol3c03684_si_001.pdf [file ol3c03684_si_001.pdf]

Supporting Information

Synthesis of Heterocycle-Substituted Bicyclo[3.1.1]heptanes and Aza-bicyclo[3.1.1]heptanes via Photocatalytic Minisci Reaction

Rebecca I. Revie[a], Benjamin J. Whitaker[a], Bhaskar Paul[a], Russell C. Smith[b], and Edward A. Anderson[a]*

[a]Chemistry Research Laboratory, Department of Chemistry, University of Oxford, 12 Mansfield Road, Oxford, OX1 3TA, United Kingdom
[b]AbbVie Drug Discovery Science & Technology (DDST), North Chicago, IL, USA.

*Corresponding Author. Email: edward.anderson@chem.ox.ac.uk

Table of Contents

Materials and Methods 2

Reaction Schemes 3

General Procedures 4

Experimental Procedures and Characterisation Data 5

Preparation of redox active ester **2a** 5

Preparation of redox active esters **2b** and **2c** 7

Preparation of redox active esters **2d-2f** 9

Preparation of redox active ester **2g** 12

Preparation of redox active esters **6a** and **6b** 14

Minisci Reaction Products 15

NMR Spectra 26

References 80

Materials and Methods

General Experimental Information: All reagents were used directly as supplied. Solvents were either used as commercially supplied, or purified by standard techniques. Anhydrous Et_2O, CH_2Cl_2, dimethylformamide and toluene were obtained from solvent dispenser units having been passed through an activated alumina column under argon. All reactions sensitive to air or moisture were performed under a nitrogen atmosphere with anhydrous solvents and glassware either dried in an oven or with a heat gun under vacuum. Reactions were monitored by thin layer chromatography on pre-coated aluminium-backed plates (Merck Kieselgel 60 with fluorescent indicator UV254). Spots were visualised by quenching of UV fluorescence or by staining with potassium permanganate, phosphomolybdic acid (PMA) stain, vanillin or bromocresol green stain, and retention factors are reported with the solvent system in parentheses. Flash column chromatography was performed on Geduran Silica Gel 60 (40–63 μm) or activated, neutral aluminium oxide (40-160 μm) obtained from Merck. Reverse phase chromatography was performed on a Biotage KP-C18-HS Flash Cartridge.

NMR spectroscopy: ^{1}H and ^{13}C spectra were recorded on a Bruker III HD nanobay spectrometer equipped with a 9.4 T magnet or Bruker NEO 600 with broadband helium cryoprobe equipped with a 11.75 T magnet. ^{1}H spectra were recorded at 400.3 MHz or 600.4 MHz, and ^{13}C spectra were recorded at 100.7 MHz or 151.0 MHz with proton decoupling. Assignments were supported where necessary by 2D COSY, HSQC and NOESY experiments. Peak multiplicities are recorded as s = singlet, d = doublet, t = triplet, q = quartet, quint. = quintet, m = multiplet, or combinations thereof. In ^{1}H NMR spectra, chemical shifts are reported to the nearest 0.01 ppm and coupling constants (J) are reported to the nearest 0.1 Hz. In ^{13}C NMR spectra, chemical shifts are reported to the nearest 0.1 ppm.

Infrared spectroscopy: Infrared spectra were recorded using a Bruker Tensor 27 FT-IR spectrometer with the sample prepared as a thin film on a diamond ATR module. Absorption maxima (ν_{max}) are quoted in wavenumbers (cm^{-1}) and peaks are classified as w = weak, s = strong, br = broad, or combinations thereof.

Mass spectrometry: High resolution mass spectra were recorded on a Bruker Micro Tof mass spectrometer. High resolution values are calculated to 4 decimal places from with molecular formula and all recorded values are within a tolerance of 5 ppm.

Photochemical setup: Photochemical reactions were carried out in an EvoluChem PhotoRedOx Box with an LED lamp (HCK1012-01-010 405 nm or HCK1012-01-002 450-455 nm) positioned 10 cm away from the vial and fan cooling.

Melting points: Melting points were recorded using a Gallenkamp melting point apparatus and are uncorrected.

Reaction Schemes

Scheme 1. Preparation of redox active esters 2b-c.

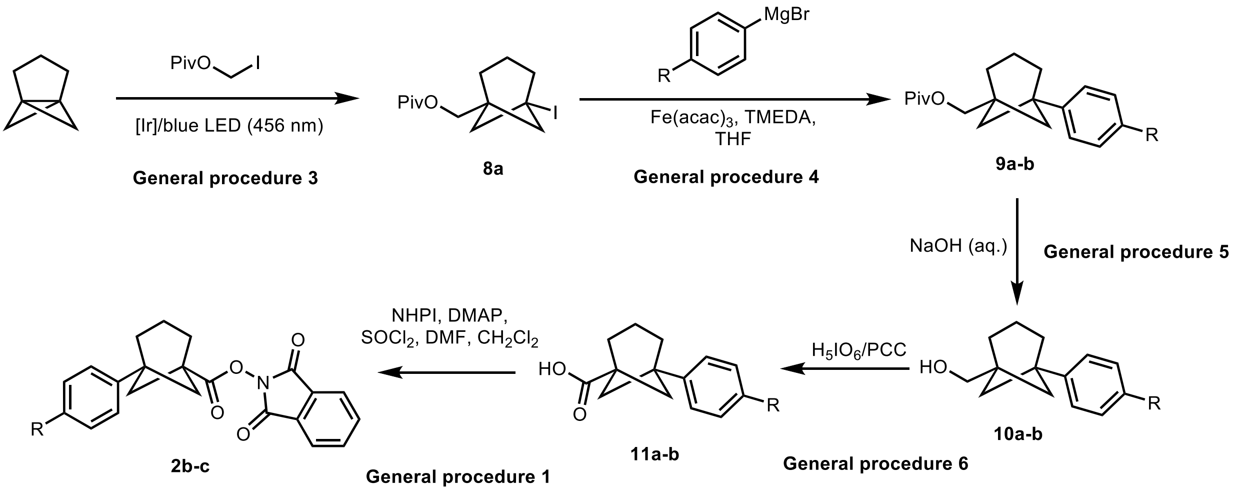

Scheme 2. Preparation of redox active esters 2d-f.

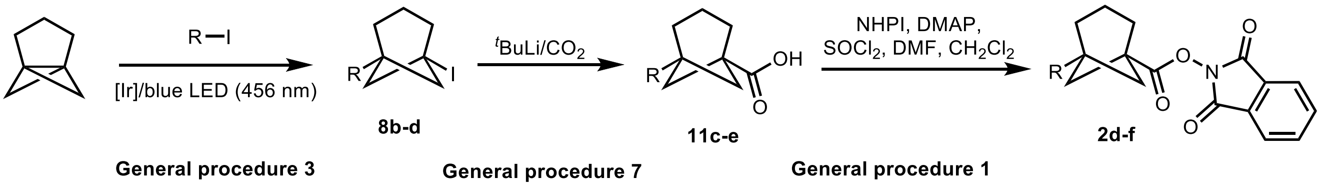

Scheme 3. Preparation of redox active ester 2g.

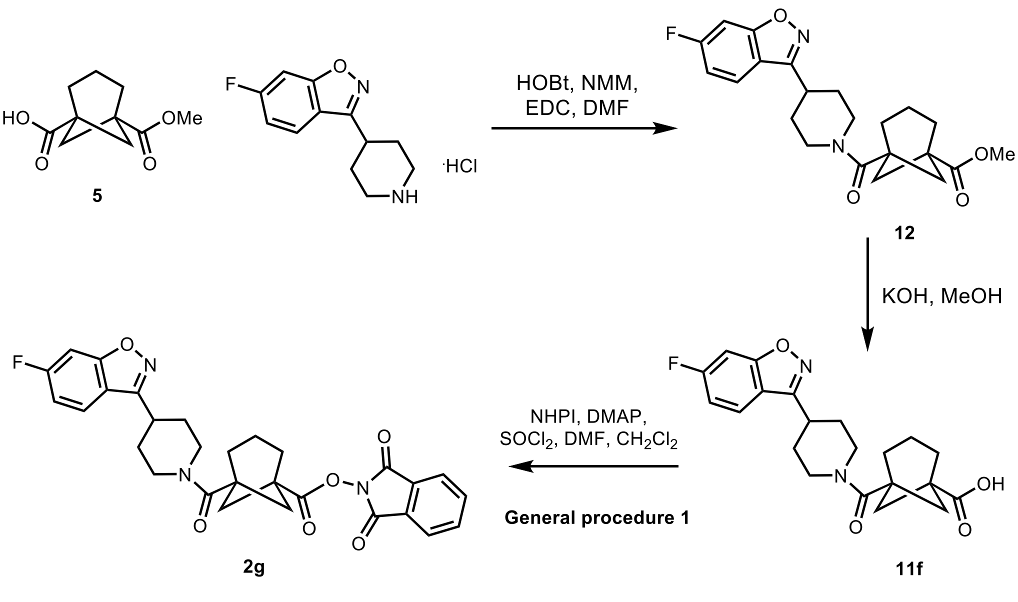

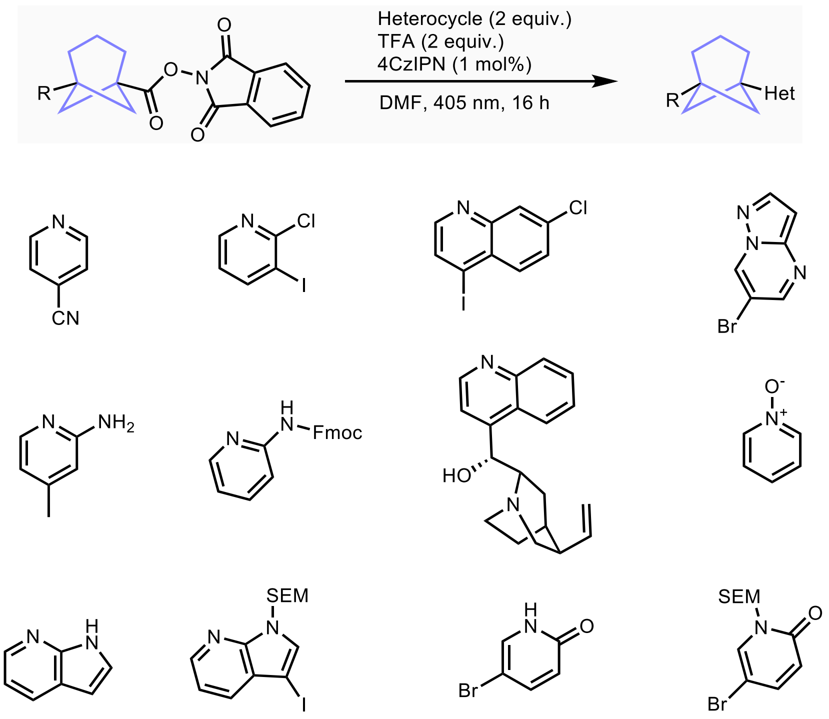

Figure 1: Examples of unsuccessful substrates.

General Procedure 1: Preparation of redox active ester

To a solution of the carboxylic acid substrate (1.0 equiv.) in anhydrous CH_2Cl_2 (0.05 M) was added thionyl chloride (2.0 equiv.) and anhydrous DMF (0.30 equiv.) and the mixture was stirred at rt for 2 h. DMAP (1.0 equiv.) and *N*-hydroxyphthalimide (2.0 equiv.) were added, then the flask was foil wrapped and stirred for 18 h. The mixture was washed with sat. $NaHCO_3$ solution (x3), then brine, then the organic phase was dried with anhydrous $MgSO_4$, filtered and concentrated under reduced pressure.

General Procedure 2: Minisci reaction

To a solution of the redox active ester substrate (1.0 equiv.), heterocycle (2.0 equiv.) and 4CzIPN (0.01 equiv.) in dry DMF (0.06 M) in a vial was added TFA (2.0 equiv.) under a nitrogen atmosphere. The vial was sealed and irradiated at 405 nm with stirring and fan cooling for 16 h. The internal temperature was measured at 31 °C. The solvent was removed under reduced pressure, then CH_2Cl_2, sat. $NaHCO_3$ solution and water were added. The aqueous layer was extracted with CH_2Cl_2 (x3), then the combined organic extracts were dried with $MgSO_4$, filtered and concentrated under reduced pressure.

General Procedure 3: ATRA from [3.1.1]propellane

The BCHep iodides were prepared *via* a modified literature procedure[1]. A solution of alkyl iodide (1.0 equiv.), [3.1.1]propellane (0.41 M in *n*-Bu_2O, 1.4 equiv.) and *fac*-Ir(ppy)$_3$ (0.025 equiv.) in pivalonitrile (0.15 M), in a vial under a nitrogen atmosphere, was irradiated with blue LEDs (Kessil PR160, 456 nm) for 5 h with stirring and fan cooling. The reaction mixture was concentrated under reduced pressure and directly purified by column chromatography.

General Procedure 4: Fe-catalysed Kumada coupling

The Kumada coupling was carried out *via* a modified literature procedure[1]. A solution of BCHep iodide (1.0 equiv.), Fe(acac)$_3$ (0.20 equiv.) and TMEDA (0.40 equiv.) in anhydrous THF (0.45 M) was stirred at rt for 15 min. The arylmagnesium bromide (1.6 equiv.) was added dropwise *via* a syringe pump over 1 h, then the mixture stirred for at rt for a further 1 h. The mixture was quenched with sat. NH_4Cl and extracted with Et_2O (x3). The combined organic layers were washed with brine, dried over anhydrous Na_2SO_4, filtered and concentrated under reduced pressure.

General Procedure 5: Hydrolysis of pivalate ester

To a solution of pivalate ester **9a-b** (1.0 equiv.) in MeOH (0.038 M), was added NaOH (aq., 1M, 6.0 equiv.) and the resulting mixture was heated at 55 °C using an oil bath for 15 h. The mixture was concentrated under reduced pressure and the residue was dissolved in EtOAc, and H_2O. The layers were separated and the aqueous layer was extracted with EtOAc (x2). The combined organic layers were dried over Na_2SO_4, and concentrated under reduced pressure.

General Procedure 6: Oxidation of alcohol to carboxylic acid

The carboxylic acids were prepared *via* a modified literature procedure[2]. A mixture of H_5IO_6 (2.1 equiv.) in MeCN (8.0 mL) was stirred vigorously at rt for 15 min. The mixture was cooled to 0 °C, and a solution of alcohol **10a-b** (1.0 equiv.) in MeCN (0.32 M) was added, followed by a solution of PCC (0.020 equiv.) in MeCN (0.013 M). The reaction mixture was stirred at 0 °C for 1 h and then warmed to room temperature and stirred for another 2 h. The mixture was extracted with EtOAc (x3), and the organic layer was washed with sat. $NaHSO_3$ and brine. The organic layer was dried over Na_2SO_4, filtered and concentrated under reduced pressure.

General Procedure 7: Carboxylation of BCHep

A solution of BCHep iodide (1.0 equiv.) in anhydrous Et_2O (0.11 M) under a nitrogen atmosphere was cooled to -78 °C. Then, *t*-BuLi (1.7 M in pentane, 2.0 equiv.) was added dropwise and the mixture stirred for 30 minutes at -78 °C. CO_2 (g) was then bubbled through the reaction mixture for 45 min. The resulting solution was warmed to room temperature, quenched with sat. NH_4Cl and acidified to pH 1 with HCl (1N). The mixture was extracted with Et_2O (x3), dried over anhydrous Na_2SO_4 and concentrated under reduced pressure. The reaction mixture was passed through a small pad of silica and eluted with pentane, then Et_2O. The Et_2O solution was collected and concentrated under reduced pressure.

Experimental Procedures and Characterisation Data

Preparation of redox active ester 2a

Dimethyl cyclohexane-1,3-dicarboxylate

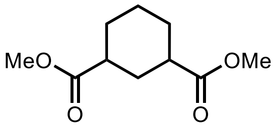

H_2SO_4 (0.668 mL, 12.6 mmol, 0.54 equiv.) was added dropwise to a stirred solution of cyclohexane-1,3-dicarboxylic acid (4.00 g, 23.2 mmol, 1.0 equiv.) in MeOH (40.0 mL, 0.58 M) at room temperature. The mixture was heated at reflux using an oil bath for 17 h, then cooled to rt, and MeOH was removed under reduced pressure. EtOAc (80 mL) and $NaHCO_3$ solution (20 mL, 0.5 M) were added. The organic layer was washed with brine (2 x 20 mL), dried with Na_2SO_4, filtered and concentrated under reduced pressure to yield the product (3.72 g, 18.6 mmol, 80%) as a pale yellow oil. A mixture of diastereomers was observed.

R_f 0.41 (4:1 pentane / EtOAc).

^{1}H NMR (400 MHz, $CDCl_3$) δ_H 3.65 (6H, s), 3.64 (6H, s), 2.65 (2H, quint, *J* = 6.0 Hz), 2.30 (2H, tt, *J* = 12.1, 3.3 Hz), 2.19 (1H, app dtt, *J* = 13.1, 3.6, 1.9 Hz), 1.98-1.92 (4H, m), 1.89-1.83 (1H, m), 1.76-1.63 (5H, m), 1.54-1.47 (3H, m), 1.38-1.22 (2H, m).

^{13}C NMR (101 MHz, $CDCl_3$) δ_C 175.7, 175.6, 51.8, 51.7, 42.6, 39.1, 31.1, 29.4, 28.4, 27.9, 24.9, 22.1.

Analytical data matches that previously reported.[3,4]

Dimethyl bicyclo[3.1.1]heptane-1,5-dicarboxylate (4)

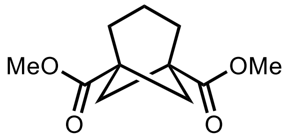

To a solution of anhydrous DIPA (2.82 mL, 24.0 mmol, 2.4 equiv.) in anhydrous THF (25.0 mL) at 0 °C was added *n*-BuLi (17.1 mL of 1.3 M solution in hexanes, 22.2 mmol, 2.2 equiv.). The resulting LDA solution was cooled to –78 °C and anhydrous DMPU (3.14 mL, 26.0 mmol, 2.6 equiv.) was added slowly. A solution of dimethyl cyclohexane-1,3-dicarboxylate (2.00 g, 10.0 mmol, 1.0 equiv.) in anhydrous THF (13.0 mL) was added slowly at –78 °C and the resulting enolate solution was stirred for 1 h. The mixture was warmed to 0 °C then a solution of CH_2I_2 (1.05 mL, 13.0 mmol, 1.3 equiv.) in anhydrous THF (15.0 mL) was added dropwise over 30 min. The mixture was warmed to rt, covered with aluminium foil, and stirred for 19 h. The mixture was quenched with sat. NH_4Cl solution (25 mL) at 0 °C. The aqueous layer was extracted with EtOAc (3 x 50 mL) and the combined organic layers were washed with brine (50 mL), dried with $MgSO_4$, filtered and concentrated under reduced pressure. Purification by column chromatography (SiO_2, pentane/EtOAc 4:1) gave **4** (654 mg, 3.08 mmol, 31%) as a pale yellow oil.

R_f 0.44 (4:1 pentane / EtOAc).

^{1}H NMR (400 MHz, $CDCl_3$) δ_H 3.66 (6H, s), 2.49 (2H, m), 2.00-1.95 (4H, m), 1.93-1.87 (2H, m), 1.83-1.78 (2H, m).

^{13}C NMR (101 MHz, $CDCl_3$) δ_C 175.5, 51.8, 42.2, 38.0, 29.5, 15.9.

Analytical data matches that previously reported.[5]

5-(Methoxycarbonyl)bicyclo[3.1.1]heptane-1-carboxylic acid (5)

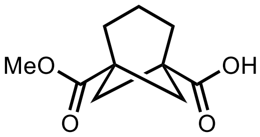

To a stirred solution of **4** (212 mg, 1.00 mmol, 1.0 equiv.) in 80% aqueous MeOH (1.20 mL) at 0 °C was added $Ba(OH)_2$ (85.7 mg, 0.50 mmol, 0.50 equiv.) portionwise. The mixture was warmed to rt and stirred for 20 h. Another equivalent of $Ba(OH)_2$ (85.7 mg, 0.500 mmol, 0.50 equiv.) was then added portionwise at 0 °C and the mixture stirred at rt for 22 h. MeOH was removed under reduced pressure and water (10 mL) was added. The mixture was washed with pentane (3 x 10 mL), acidified with conc. HCl to pH 3, and extracted with EtOAc (3 x 10 mL). The combined extracts were dried over anhydrous Na_2SO_4 and concentrated under reduced pressure. Purification by reverse phase column chromatography (C_{18} reversed phase silica, water/MeCN 1:9) gave the product **5** (79.1 mg, 0.400 mmol, 40%) as a white crystalline solid.

R_f 0.45 (9:1 CH_2Cl_2 / MeOH).

^{1}H NMR (400 MHz, $CDCl_3$) δ_H 9.27 (1H, br s), 3.67 (3H, s), 2.55-2.50 (2H, m), 2.02-1.97 (4H, m), 1.94-1.87 (2H, m), 1.85-1.80 (2H, m).

^{13}C NMR (125 MHz, $CDCl_3$) δ_C 181.5, 175.6, 52.0, 42.3, 42.2, 38.0, 29.5, 29.2, 16.0.

Analytical data matches that previously reported.[6,7]

1-(1,3-Dioxoisoindolin-2-yl) 5-methyl bicyclo[3.1.1]heptane-1,5-dicarboxylate (2a)

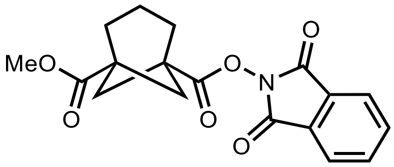

The product was prepared by **General Procedure 1** from **5** (144 mg, 0.726 mmol, 1.0 equiv.), then purified by column chromatography (SiO_2, pentane/EtOAc 7:3) to give the product (31.1 mg, 90.6 μmol, 65%) as a white crystalline solid.

R_f 0.37 (7:3 pentane / EtOAc).

^{1}H NMR (400 MHz, $CDCl_3$) δ_H 7.88 (2H, dd, J = 5.5, 3.1 Hz), 7.79 (2H, dd, J = 5.5, 3.1 Hz), 3.70 (3H, s), 2.79-2.75 (2H, m), 2.23 (2H, t, J = 6.9 Hz), 2.11-1.91 (6H, m).

^{13}C NMR (125 MHz, $CDCl_3$) δ_C 203.7, 170.8, 162.1, 134.9, 129.2, 124.1, 52.1, 42.9, 41.0, 38.3, 29.5, 29.4, 15.9.

IR (thin film, ν_{max} / cm^{-1}; selected peaks): 2953 (w, C-H), 2874 (w, C-H), 1784 (s, C=O), 1747 (s, C=O).

HRMS (ES^+) calc. for $C_{18}H_{17}NO_6Na$ $[M+Na]^+$ 366.0948, found 366.0942, error -1.57 ppm.

m.p. 103-105 °C

Preparation of redox active esters 2b and 2c

(5-Iodobicyclo[3.1.1]heptan-1-yl)methyl pivalate (8a)

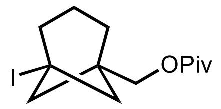

The product was prepared by **General Procedure 3** from iodomethyl pivalate (182 mg, 0.75 mmol, 1.0 equiv.) and [3.1.1]propellane solution (2.6 mL, 0.41 M in *n*-Bu_2O, 1.05 mmol, 1.4 equiv.). Purification of the crude reaction mixture by column chromatography (SiO_2; pentane/EtOAc, gradient 99:1 to 95:5) afforded **8a** (189 mg, 0.56 mmol, 75%) as colourless oil.

R_f 0.30 (19:1 pentane / EtOAc).

^{1}H NMR (400 MHz, $CDCl_3$) δ_H 3.84 (s, 2H), 2.61–2.53 (m, 4H), 2.26–2.21 (m, 2H), 1.92–1.85 (m, 2H), 1.66–1.62 (m, 2H), 1.21 (s, 9H).

^{13}C NMR (101 MHz, $CDCl_3$) δ_C 178.3, 68.5, 48.7, 46.3, 43.6, 39.1, 33.2, 28.9, 27.4, 19.3.

Analytical data matches that previously reported[1].

Preparation of 2b

(5-(4-Fluorophenyl)bicyclo[3.1.1]heptan-1-yl)methyl pivalate (9a)

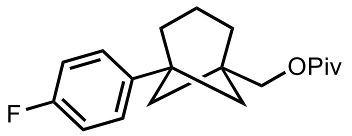

The product was prepared by **General Procedure 4** using **8a** (150 mg, 0.450 mmol, 1.0 equiv.) and (4-fluorophenyl)magnesium bromide (0.360 mL, 2.0 M in THF, 0.710 mmol, 1.6 equiv.). The crude product was purified by column chromatography (SiO_2; pentane/EtOAc, gradient 100:0 to 99:1), to give the product as light-yellow oil (126.3 mg, 0.414 mmol, 93%).

R_f 0.32 (19:1 pentane / EtOAc).

^{1}H NMR (400 MHz, $CDCl_3$) δ_H 7.07–7.01 (m, 2H), 6.99–6.93 (m, 2H), 3.89 (s, 2H), 1.96–1.81 (m, 8H), 1.74 (t, J = 6.9 Hz, 2H), 1.19 (s, 9H).

^{13}C NMR (101 MHz, $CDCl_3$) δ_C 178.6, 161.1 (d, J = 243.1 Hz), 146.1 (d, J = 3.1 Hz), 126.4 (d, J = 7.7 Hz), 115.0 (d, J = 21.1 Hz), 70.2, 42.6, 39.1, 38.9, 38.8, 37.8, 30.6, 27.4, 17.2.

^{19}F NMR (377 MHz, $CDCl_3$) δ_F -118.1.

IR (thin film, ν_{max} / cm^{-1}; selected peaks): 2945 (w, C-H), 1731 (C=O).

HRMS (ES^+) Not found.

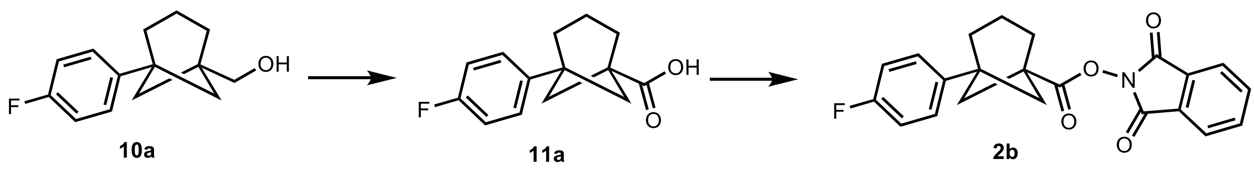

(5-(4-Fluorophenyl)bicyclo[3.1.1]heptan-1-yl)methanol (10a) was prepared by **General Procedure 5** using **9a** (115 mg, 0.377 mmol, 1.0 equiv.) to give the product (79.1 mg, 0.358 mmol, 95%) as a light yellow oil, which was used in the next step without further purification.

5-(4-Fluorophenyl)bicyclo[3.1.1]heptane-1-carboxylic acid (11a) was then prepared by **General Procedure 6** using **10a** (70 mg, 0.317 mmol, 1.0 equiv.) to give the product (64.8 mg, 0.276 mmol, 87%) as a yellow oil, which was used in the next step without further purification.

1,3-Dioxoisoindolin-2-yl 5-(4-fluorophenyl)bicyclo[3.1.1]heptane-1-carboxylate (2b) was then prepared by **General Procedure 1** from **11a** (57.0 mg, 0.243 mmol, 1.0 equiv.), then purified by column chromatography (SiO_2, pentane/EtOAc 7:3) to give the title compound (29.2 mg, 77.0 µmol, 32%) as a yellow oil.

R_f 0.49 (7:3 pentane / EtOAc).

^{1}H NMR (400 MHz, $CDCl_3$) δ_H 7.87 (2H, dd, *J* = 5.5, 3.1 Hz), 7.78 (2H, dd, *J* = 5.5, 3.1 Hz), 7.10-7.05 (2H, m), 7.02-6.96 (2H, m), 2.64-2.59 (2H, m), 2.30-2.24 (4H, m), 2.09-2.02 (2H, m), 1.95 (2H, t, *J* = 7.3 Hz).

^{13}C NMR (101 MHz, $CDCl_3$) δ_C 171.2, 162.1, 161.4 (d, *J* = 243.7 Hz), 144.5 (d, *J* = 3.1 Hz), 134.8, 129.1, 126.4 (d, *J* = 7.9 Hz), 124.0, 115.3 (d, *J* = 21.2 Hz), 42.7, 41.3, 40.0, 36.7, 29.5, 16.8.

^{19}F NMR (377 MHz, $CDCl_3$) δ_F -117.2.

IR (thin film, ν_{max} / cm^{-1}; selected peaks): 2951 (w, C-H), 2867 (w, C-H), 1743 (s, C=O).

HRMS (ES^+) calc. for $C_{22}H_{19}FNO_4$ $[M+H]^+$ 380.1293, found 380.1308, error +4.03 ppm.

<u>Preparation of 2c</u>

(5-(*p*-Tolyl)bicyclo[3.1.1]heptan-1-yl)methyl pivalate (9b)

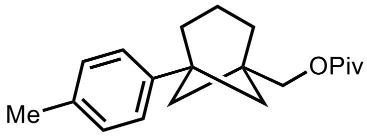

9b was prepared by **General Procedure 4** using **8a** (150 mg, 0.450 mmol, 1.0 equiv.) and *p*-tolylmagnesium bromide (0.71 mL, 1.0 M in THF, 0.71 mmol, 1.6 equiv.). The crude product was purified by column chromatography (SiO_2; pentane/EtOAc, gradient 100:0 to 98:2), to give the product as light-yellow oil (119.3 mg, 0.397 mmol, 89%).

R_f 0.36 (19:1 pentane / EtOAc).

^{1}H NMR (400 MHz, $CDCl_3$) δ_H 7.11 (d, *J* = 7.7 Hz, 2H), 7.0 (d, *J* = 8.2 Hz, 2H), 3.89 (s, 2H), 2.32 (s, 3H), 1.95 – 1.81 (m, 8H), 1.74 (t, *J* = 6.8 Hz, 2H), 1.19 (s, 9H).

^{13}C NMR (101 MHz, $CDCl_3$) δ_C 178.7, 147.4, 135.1, 129.0, 124.8, 70.5, 42.8, 39.1, 38.9, 37.8, 30.7, 27.4, 21.1, 17.3.

IR (thin film, ν_{max} / cm^{-1}; selected peaks): 2944 (w, C-H), 1731 (s, C=O).

HRMS (ES^+) Not found.

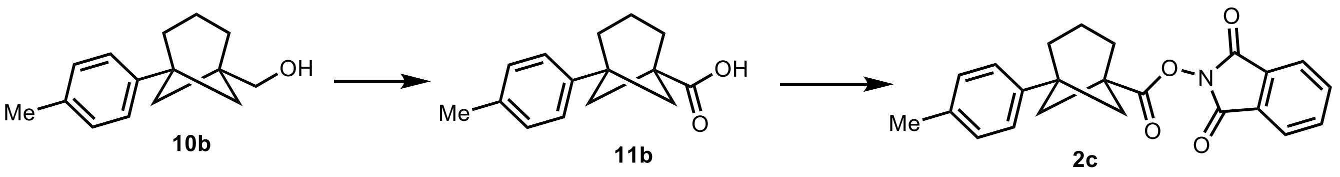

(5-(*p*-Tolyl)bicyclo[3.1.1]heptan-1-yl)methanol (10b) was prepared by **General Procedure 5** using **9b** (115 mg, 0.382 mmol, 1.0 equiv.) to give **10b** (80.3 mg, 0.371 mmol, 97%) as a light yellow oil, which was used in the next step without further purification.

5-(*p*-Tolyl)bicyclo[3.1.1]heptane-1-carboxylic acid (11b) was then prepared by **General Procedure 6** using **10b** (72 mg, 0.332 mmol, 1.0 equiv.) to give the product (65.1 mg, 0.282 mmol, 85%) as a yellow oil, which was used in the next step without further purification.

1,3-Dioxoisoindolin-2-yl 5-(p-tolyl)bicyclo[3.1.1]heptane-1-carboxylate (2c) was then prepared by **General Procedure 1** from **11b** (55.0 mg, 0.239 mmol, 1.0 equiv.), then purified by column chromatography (SiO_2, pentane/EtOAc 7:3) to give the title compound (26.1 mg, 69.5 μmol, 29%) as a yellow oil.

R_f 0.48 (7:3 pentane / EtOAc).

^{1}H NMR (400 MHz, $CDCl_3$) δ_H 7.87 (2H, dd, J = 5.5, 3.1 Hz), 7.77 (2H, dd, J = 5.5, 3.1 Hz), 7.14 (2H, d, J = 7.9 Hz), 7.02 (2H, d, J = 8.1 Hz), 2.65-2.61 (2H, m), 2.33 (3H, s), 2.30-2.24 (4H, m), 2.09-2.02 (2H, m), 1.98-1.95 (2H, m).

^{13}C NMR (101 MHz, $CDCl_3$) δ_C 171.4, 162.2, 145.8, 135.6, 134.8, 129.2, 124.7, 124.0, 42.9, 41.4, 40.1, 36.7, 29.7, 21.2, 16.8.

IR (thin film, ν_{max} / cm^{-1}; selected peaks): 2949 (w, C-H), 2867 (w, C-H), 1781 (s, C=O), 1744 (s, C=O).

HRMS (ES^+) calc. for $C_{23}H_{22}NO_4$ $[M+H]^+$ 376.1543, found 376.1536, error -1.97 ppm.

Preparation of redox active esters 2d-2f

Preparation of redox active ester 2d

1-Iodo-5-(4-(trifluoromethyl)benzyl)bicyclo[3.1.1]heptane (8b)

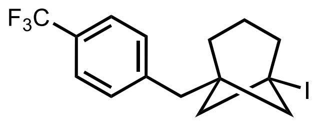

The product was prepared by **General Procedure 3** from 1-(iodomethyl)-4-(trifluoromethyl)benzene (215 mg, 0.75 mmol, 1.0 equiv.) and [3.1.1]propellane solution (2.6 mL, 0.41 M in *n*-Bu_2O, 1.05 mmol, 1.4 equiv.). Purification of the crude reaction mixture by column chromatography (SiO_2; 100% pentane) afforded **8b** (222 mg, 0.58 mmol, 78%) as yellow oil.

R_f 0.24 (100% pentane).

^{1}H NMR (400 MHz, $CDCl_3$) δ_H 7.53 (d, J = 7.8 Hz, 2H), 7.16 (d, J = 7.8 Hz, 2H), 2.75 (s, 2H), 2.59–2.54 (m, 2H), 2.51 (t, J = 7.2 Hz, 2H), 2.33–2.28 (m, 2H), 1.80 (quint., J = 7.0 Hz, 2H), 1.54–1.51 (m, 2H).

^{13}C NMR (101 MHz, $CDCl_3$) δ_C 142.4, 129.8, 128.9 (q, J = 2.3 Hz), 125.3 (q, J = 3.7 Hz), 124.7 (q, J = 272.7 Hz), 51.8, 47.1, 47.0, 43.5, 34.3, 31.0, 19.7.

^{19}F NMR (377 MHz, $CDCl_3$) δ_F -62.4.

Analytical data matches that previously reported[1].

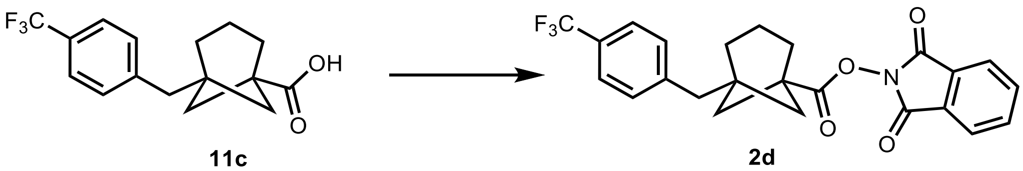

5-(4-(Trifluoromethyl)benzyl)bicyclo[3.1.1]heptane-1-carboxylic acid (11c)

The product was prepared by **General Procedure 7** from **8b** (250 mg, 0.660 mmol, 1.0 equiv.) to give the product (136 mg, 0.450 mmol, 69%) as a yellow oil which was used in the next step without further purification.

1,3-Dioxoisoindolin-2-yl 5-(4-(trifluoromethyl)benzyl)bicyclo[3.1.1]heptane-1-carboxylate (2d) was then prepared by **General Procedure 1** from **11c** (100 mg, 0.335 mmol, 1.0 equiv.), then purified by column chromatography (SiO_2, pentane/EtOAc 8:2) to give the title compound (21.3 mg, 90.6 μmol, 14%) as a yellow oil.

R_f 0.29 (8:2 pentane / EtOAc).

^{1}H NMR (500 MHz, $CDCl_3$) δ_H 7.88 (2H, dd, J = 5.5, 3.1 Hz), 7.78 (2H, dd, J = 5.5, 3.1 Hz), 7.54 (2H, d, J = 7.9 Hz), 7.21 (2H, d, J = 8.1 Hz), 2.76 (2H, s), 2.34-2.30 (2H, m), 2.16 (2H, t, J = 7.3 Hz), 1.89-1.93 (4H, m), 1.62 (2H, t, J = 7.2 Hz).

^{13}C NMR (125 MHz, $CDCl_3$) δ_C 171.4, 162.2, 142.5, 134.8, 129.9, 129.2, 128.7 (q, J = 32.3 Hz), 125.3 (q, J = 4.1 Hz), 124.5 (q, J = 272.0 Hz), 124.1, 47.0, 41.8, 40.3, 40.2, 32.1, 30.0, 16.5.

^{19}F NMR (470 MHz, $CDCl_3$) δ_F -62.3.

IR (thin film, ν_{max} / cm^{-1}; selected peaks): 2948 (w, C-H), 1782 (s, C=O), 1746 (s, C=O).

HRMS (ES$^+$) calc. for $C_{24}H_{21}F_3NO_4$ [M+H]$^+$ 444.1417, found 444.1418, error +0.17 ppm.

<u>Preparation of redox active ester 2e</u>

1-(3,5-Bis(trifluoromethyl)benzyl)-5-iodobicyclo[3.1.1]heptane (8c)

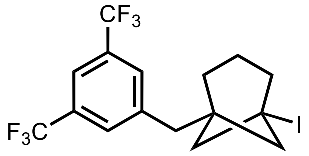

The product was prepared by **General Procedure 3** from 1-(iodomethyl)-3,5-bis(trifluoromethyl)benzene (265.5 mg, 0.75 mmol, 1.0 equiv.) and [3.1.1]propellane solution (2.6 mL, 0.41 M in *n*-Bu_2O, 1.05 mmol, 1.4 equiv.). Purification of the crude reaction mixture by column chromatography (SiO_2; 100% pentane) afforded **8c** (269 mg, 0.6 mmol, 80%) as yellow oil.

R_f 0.24 (100% pentane).

^{1}H NMR (400 MHz, $CDCl_3$) δ_H 7.75 (s, 1H), 7.49 (s, 2H), 2.84 (s, 2H), 2.60-2.55 (m, 2H), 2.51 (t, J = 7.2 Hz, 2H), 2.36–2.31 (m, 2H), 1.81 (quint., J = 7.1 Hz, 2H), 1.51 (t, J = 7.4 Hz, 2H).

^{13}C NMR (101 MHz, $CDCl_3$) δ_C 140.7, 131.7 (q, J = 33.3 Hz), 129.5 (q, J = 3.7 Hz), 123.5 (q, J = 273.7 Hz), 120.6 (quint. J = 4.0 Hz), 51.6, 46.9, 46.8, 43.4, 33.2, 30.8, 19.6.

^{19}F NMR (377 MHz, $CDCl_3$) δ_F -62.8

IR (thin film, ν_{max} / cm^{-1}; selected peaks): 2865 (w, C-H).

HRMS (ES$^+$) Not found.

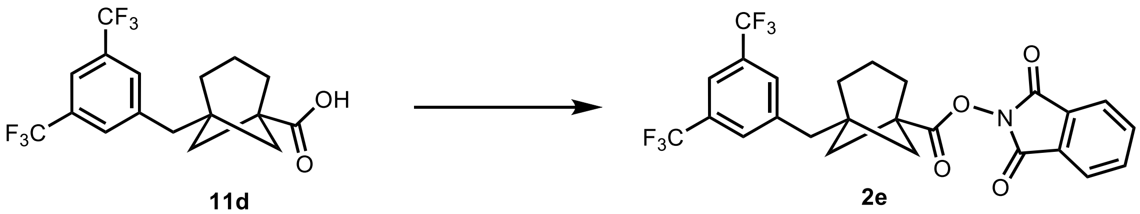

11d → **2e**

5-(3,5-Bis(trifluoromethyl)benzyl)bicyclo[3.1.1]heptane-1-carboxylic acid (11d)

The product was prepared by **General Procedure 7** from **8c** (250 mg, 0.560 mmol, 1.0 equiv.) to give the product (146 mg, 0.400 mmol, 71%) as a yellow oil which was used in the next step without further purification.

1,3-Dioxoisoindolin-2-yl 5-(3,5-bis(trifluoromethyl)benzyl)bicyclo[3.1.1]heptane-1-carboxylate (2e) was then prepared by **General Procedure 1** from **11d** (115 mg, 0.314 mmol, 1.0 equiv.), then purified by column chromatography (SiO_2, pentane/EtOAc 8:2) to give the title compound (114 mg, 0.223 mmol, 71%) as a yellow oil.

R_f 0.34 (8:2 pentane / EtOAc).

^{1}H NMR (500 MHz, $CDCl_3$) δ_H 7.88 (2H, dd, J = 5.5, 3.1 Hz), 7.79 (2H, dd, J = 5.5, 3.1 Hz), 7.75 (1H, s), 7.54 (2H, s), 2.85 (2H, s), 2.36-2.33 (2H, m), 2.17 (2H, t, J = 7.2 Hz), 1.92-1.85 (4H, m), 1.60 (2H, t, J = 7.2 Hz).

^{13}C NMR (125 MHz, $CDCl_3$) δ_C 171.2, 162.2, 140.8, 134.9, 131.7 (q, J = 33.2 Hz), 129.5 (q, J = 3.8 Hz), 129.1, 124.1, 123.5 (q, J = 272.7 Hz), 120.5 (sept, J = 3.9 Hz), 46.8, 41.8, 40.1, 31.9, 29.9, 16.4.

^{19}F NMR (470 MHz, $CDCl_3$) δ_F -62.8.

IR (thin film, ν_{max} / cm^{-1}; selected peaks): 2920 (w, C-H), 1783 (s, C=O), 1745 (s, C=O).

HRMS (ES$^+$) calc. for $C_{25}H_{20}F_6NO_4$ [M+H]$^+$ 512.1291, found 512.1305, error +2.72 ppm.

<u>Preparation of redox active ester 2f</u>

1-Iodo-5-(trifluoromethyl)bicyclo[3.1.1]heptane (8d)

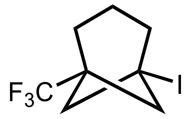

The product was synthesized according to the literature procedure, reported from our group[1]. A solution of [3.1.1]propellane (2.60 mL, 0.41 M in *n*-Bu_2O, 1.05 mmol, 1.0 equiv.) was cooled to –78 °C, then $CF_3I{\cdot}2DMSO$ (1.0 mL) was added. The resulting solution was allowed to warm up to rt and stirred for 1 h. The mixture was extracted with Et_2O (3 x 6 mL) and washed with LiCl solution (2 mL, 10% aq.). The solution was dried over Na_2SO_4, concentrated in vacuo, and purified by column chromatography (SiO_2, pentane) afforded **8d** (177mg, 0.61 mmol, 58%) as a colourless oil. Note: This molecule is volatile, not detectable via TLC and elutes from the column in the first few fractions. It coalesces with the bromobenzene fraction which is detectable by UV. Bromobenzene can be removed in vacuo under partial sacrificial loss of **8d**.

^{1}H NMR (400 MHz, $CDCl_3$) δ_H 2.83 (dt, J = 7.7, 3.9 Hz, 2H), 2.57–2.54 (m, 2H), 2.33–2.28 (m, 2H), 1.98–1.86 (m, 4H).

^{13}C NMR (101 MHz, $CDCl_3$) δ_C 126.0 (q, J = 278.8 Hz), 47.7 (q, J = 31.3 Hz), 45.5 (q, J = 2.4 Hz), 42.7, 28.0, 24.8 (q, J = 2.0 Hz), 18.6.

^{19}F NMR (377 MHz, $CDCl_3$) δ_F -78.0

Analytical data matches that previously reported[1].

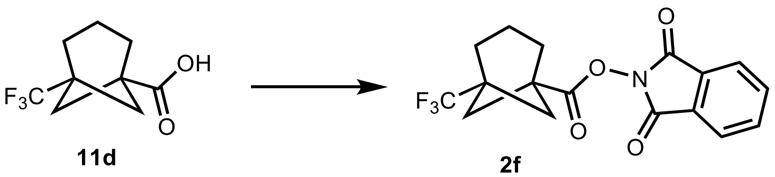

11d

2f

5-(Trifluoromethyl)bicyclo[3.1.1]heptane-1-carboxylic acid (11e)

The product was prepared by **General Procedure 7** from **8d** (100 mg, 0.340 mmol, 1.0 equiv.) to give the product (146 mg, 0.400 mmol, 71%) as a yellow oil which was used in the next step without further purification.

1,3-Dioxoisoindolin-2-yl 5-(trifluoromethyl)bicyclo[3.1.1]heptane-1-carboxylate (2f) was then prepared by **General Procedure 1** from **11e** (67.0 mg, 0.322 mmol, 1.0 equiv.), then purified by column chromatography (SiO_2, pentane/EtOAc 8:2) to give the title compound (42.4 mg, 120 μmol, 37%) as a white solid.

$\mathbf{R_f}$ 0.29 (8:2 pentane / EtOAc).

^{1}**H NMR** (500 MHz, $CDCl_3$) δ_H 7.89 (2H, dd, *J* = 5.6, 3.0 Hz), 7.80 (2H, dd, *J* = 5.6, 3.1 Hz), 2.69-2.64 (2H, m), 2.25 (2H, t, *J* = 6.5 Hz), 2.07-1.97 (4H, m), 1.96-1.91 (2H, m).

^{13}**C NMR** (125 MHz, $CDCl_3$) δ_C 170.4, 162.0, 134.9, 129.1, 126.2 (q, *J* = 277.0 Hz), 124.1, 41.7 (q, *J* = 31.3 Hz), 40.7, 34.8 (q, *J* = 2.7 Hz), 29.5, 26.0 (q, *J* = 2.1 Hz), 15.6.

^{19}**F NMR** (470 MHz, $CDCl_3$) δ_F -78.3.

IR (thin film, ν_{max} / cm^{-1}; selected peaks): 2962 (w, C-H), 1784 (s, C=O), 1745 (s, C=O).

HRMS (ES$^+$) calc. for $C_{17}H_{15}F_3NO_4$ [M+H]$^+$ 354.0948, found 354.0944, error -1.05 ppm.

<u>**Preparation of redox active ester 2g**</u>

Methyl 5-(4-(6-fluorobenzo[d]isoxazol-3-yl)piperidine-1-carbonyl)bicyclo[3.1.1]heptane-1-carboxylate (12)

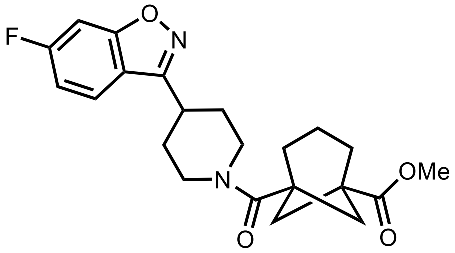

A solution of **5** (300 mg, 1.51 mmol, 1.0 equiv.) and HOBt (232 mg, 1.51 mmol, 1.0 equiv.) in DMF (3.0 mL), was cooled to 0 °C, then NMM (0.170 mL mg, 1.51 mmol, 1.0 equiv.) and EDC.HCl (290 mg, 1.51 mmol, 1.0 equiv.) were added. A pre-cooled solution of 6-fluoro-3-(4-piperidyl)-1,2-benzisoxazole hydrochloride (389 mg, 1.51 mmol, 1.0 equiv.) and NMM (0.170 mL, 1.51 mmol, 1.0 equiv.) in DMF (4.0 mL) was added. The mixture was stirred for 3 h at rt. The solvent was removed under reduced pressure, and ice-cold sat. $NaHCO_3$ (15 mL) was added. The mixture was stirred for 45 min and the precipitated product was taken into $CHCl_3$ (20 mL), then washed with sat. $NaHCO_3$ (2 x 20 mL), water (2 x 20 mL), cold HCl solution (0.1 N, 2 x 20 mL), and brine (2 x 20 mL). The organic layer was dried over anhydrous $MgSO_4$, filtered and concentrated under reduced pressure to give the product as a white foam (274 mg, 0.684 mmol, 45%).

$\mathbf{R_f}$ 0.39 (2:8 pentane / EtOAc).

^{1}**H NMR** (400 MHz, $CDCl_3$) δ_H 7.60 (1H, dd, *J* = 8.7, 5.0 Hz), 7.23 (1H, dd, *J* = 8.4, 1.7 Hz), 7.05 (1H, td, *J* = 8.8, 2.2 Hz), 4.55 (1H, d, *J* = 13.4 Hz), 3.85 (1H, d, *J* = 14.3 Hz), 3.65 (3H, s), 3.30 (1H, tt, *J* = 11.2, 3.9 Hz), 3.19 (1H, t, *J* = 12.5 Hz), 2.86 (1H, t, *J* = 12.7 Hz), 2.50 (2H, d, *J* = 9.4 Hz), 2.12-2.08 (2H, m), 2.00-1.77 (10H, m).

^{13}C NMR (101 MHz, $CDCl_3$) δ_C 175.5, 174.3, 164.3 (d, J = 251.1 Hz), 164.0 (d, J = 13.6 Hz), 160.3, 122.2 (d, J = 11.2 Hz), 117.1 (d, J = 1.0 Hz), 112.7 (d, J = 25.4 Hz), 97.7 (d, J = 26.8 Hz), 51.9, 45.1, 44.0, 41.9, 41.3, 39.4, 39.0, 34.3, 30.8, 30.5, 30.4, 29.5, 16.1.

^{19}F NMR (470 MHz, $CDCl_3$) δ_F -109.1

IR (thin film, ν_{max} / cm^{-1}; selected peaks): 2952 (w, C-H), 1729 (C=O), 1615 (C=O).

HRMS (ES$^+$) calc. for $C_{22}H_{25}FN_2O_4Na$ $[M+Na]^+$ 423.1691, found 423.1694, error +0.80 ppm.

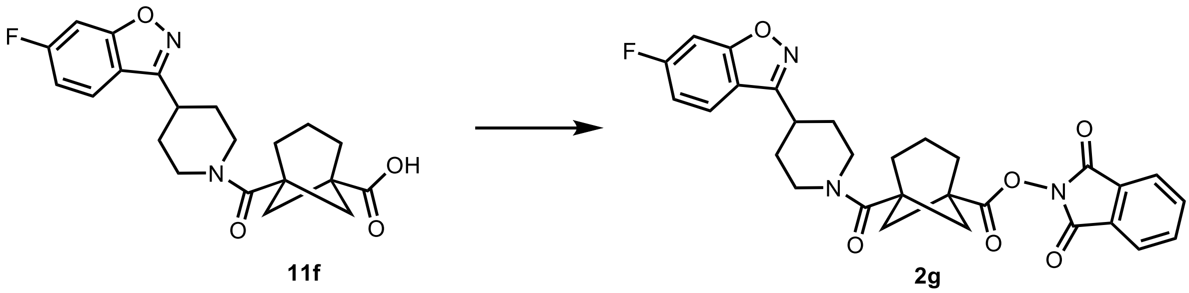

11f **2g**

5-(4-(6-Fluorobenzo[d]isoxazol-3-yl)piperidine-1-carbonyl)bicyclo[3.1.1]heptane-1-carboxylic acid (11f)

To a solution of **12** (274 mg, 0.684 mmol, 1.0 equiv.) in MeOH (20.0 mL) was added KOH (115 mg, 2.05 mmol, 3.0 equiv.). The mixture was stirred for 20 h at rt. The solvent was removed under reduced pressure, then water (20 mL) was added and the mixture was washed with pentane (3 x 20 mL) and acidified with conc. HCl solution to pH 3. The mixture was filtered to collect the precipitated product as a white solid (186 mg, 0.481 mmol, 70%) which was used directly in the next step without further purification.

1,3-Dioxoisoindolin-2-yl 5-(4-(6-fluorobenzo[d]isoxazol-3-yl)piperidine-1-carbonyl)bicyclo[3.1.1]heptane-1-carboxylate (2g) was then prepared by **General Procedure 1** from **11f** (164 mg, 0.424 mmol, 1.0 equiv.), then purified by column chromatography (SiO_2, pentane/EtOAc 8:2) to give the title compound (58.6 mg, 0.110 mmol, 26%) as a white foam.

R$_f$ 0.23 (9:1 CH_2Cl_2 / MeOH).

^{1}H NMR (500 MHz, $CDCl_3$) δ_H 7.86 (2H, dd, J = 5.6, 3.0 Hz), 7.77 (2H, dd, J = 5.6, 3.0 Hz), 7.63 (1H, dd, J = 8.8, 5.0 Hz), 7.24 (1H, dd, J = 8.3, 2.2 Hz), 7.07 (1H, td, J = 8.8, 2.1 Hz), 4.57 (1H, d, J = 13.2 Hz), 3.85 (1H, d, J = 13.6 Hz), 3.33 (1H, tt, J = 11.1, 3.9 Hz), 3.22 (1H, t, J = 12.3 Hz), 2.91 (1H, t, J = 12.3 Hz), 2.78 (2H, d, J = 10.0 Hz), 2.24-1.86 (12H, m).

^{13}C NMR (125 MHz, $CDCl_3$) δ_C 173.6, 170.6, 164.2 (d, J = 251.3 Hz), 163.9 (d, J = 13.5 Hz), 161.9, 160.1, 134.8, 129.0, 123.9, 122.2 (d, J = 11.0 Hz), 117.1 (d, J = 1.2 Hz), 112.7 (d, J = 25.7 Hz), 97.6 (d, J = 26.8 Hz), 45.0, 44.4, 41.9, 39.9, 39.4, 38.9, 34.2, 30.5, 30.2, 29.4, 15.9.

^{19}F NMR (470 MHz, $CDCl_3$) δ_F -109.0.

IR (thin film, ν_{max} / cm^{-1}; selected peaks): 2952 (w, C-H), 1781 (s, C=O), 1743 (s, C=O), 1617 (s, C=O).

HRMS (ES$^+$) calc. for $C_{29}H_{27}FN_3O_6$ $[M+H]^+$ 532.1878, found 532.1874, error -0.84 ppm.

Preparation of redox active esters 6a and 6b

3-(*tert*-Butyl) 1-(1,3-dioxoisoindolin-2-yl) 3-azabicyclo[3.1.1]heptane-1,3-dicarboxylate (6a)

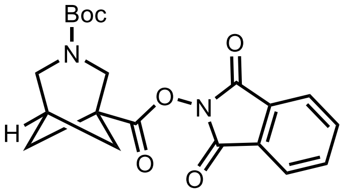

The product was prepared by **General Procedure 1** using 3-(tert-butoxycarbonyl)-3-azabicyclo[3.1.1]heptane-1-carboxylic acid (250 mg, 1.03 mmol, 1.0 equiv.), then purified by column chromatography (24 g SiO_2, heptanes/EtOAc 0-35% over 15CV) to give the product (300 mg, 776 µmol, 75%) as a white amorphous solid.

R_f 0.29 (7:3 heptanes / EtOAc).

1H NMR (600 MHz, $CDCl_3$) δ 7.94 – 7.84 (m, 2H), 7.84 – 7.74 (m, 2H), 3.89 (d, *J* = 28.4 Hz, 2H), 3.59 (dd, *J* = 11.8, 2.6 Hz, 2H), 2.75 – 2.64 (m, 2H), 2.62 – 2.43 (m, 1H), 1.86 – 1.77 (m, 2H), 1.50 (d, *J* = 2.3 Hz, 9H).

^{13}C NMR (101 MHz, $CDCl_3$) δ_C 169.0, 168.8, 161.9, 161.8, 156.0, 134.9, 134.8, 129.1, 129.0, 124.1, 124.1, 80.2, 79.9, 49.4, 49.1, 48.6, 43.0, 43.0, 34.7, 29.1, 28.9, 28.6.

A mixture of rotamers was observed in the ^{13}C NMR at 27 °C.

MS (ESI$^+$) calc. for $C_{20}H_{22}N2O_6$ [M]$^+$ 386.2, found 331.0 [M-tBu].

HRMS (ES$^+$) calc. for $C_{20}H_{22}N_2O_6Na$ [M+Na]$^+$ 409.1370, found 409.1372, error +0.46 ppm.

3-(*tert*-Butyl) 1-(1,3-dioxoisoindolin-2-yl) 5-methyl 3-azabicyclo[3.1.1]heptane-1,3,5-tricarboxylate (6b)

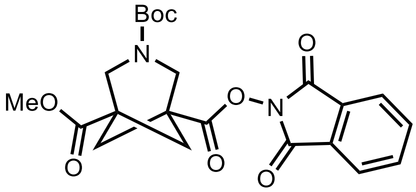

The product was prepared by **General Procedure 1** using 3-(tert-butoxycarbonyl)-5-(methoxycarbonyl)-3-azabicyclo[3.1.1]heptane-1-carboxylic acid (250 mg, 0.835 mmol, 1.0 equiv.), then purified by column chromatography (24 g SiO_2, heptanes/EtOAc 0-35% over 15CV) to give the product (213 mg, 479 µmol, 57%) as a white amorphous solid.

R_f 0.27 (7:3 heptanes / EtOAc).

1H NMR (500 MHz, $CDCl_3$) δ 7.95 – 7.86 (m, 2H), 7.86 – 7.76 (m, 2H), 3.88 (d, J = 24.6 Hz, 2H), 3.80 – 3.68 (m, 5H), 2.95 (qd, J = 7.8, 6.9, 3.0 Hz, 2H), 2.10 – 1.99 (m, 2H), 1.51 (s, 9H).

^{13}C NMR (101 MHz, $CDCl_3$) δ_C 172.2, 172.0, 168.4, 168.3, 161.8, 161.7, 155.6, 135.0, 134.9, 129.0, 129.0, 124.2, 124.2, 80.7, 80.6, 52.5, 52.4, 49.2, 48.9, 48.6, 48.3, 41.7, 41.6, 40.0, 39.9, 37.6, 37.6, 28.6.

A mixture of rotamers was observed in the ^{13}C NMR at 27 °C.

MS (ESI$^+$) calc. for $C_{22}H_{24}N_2O_8$ [M]$^+$ 444.4, found 388.8 [M-tBu].

HRMS (ES$^+$) calc. for $C_{22}H_{24}N_2O_8Na$ [M+Na]$^+$ 467.1425, found 467.1431, error +1.30 ppm.

Minisci Reaction Products

Note on purification of Minisci products: Several methods for chromatography have been investigated. Standard silica column chromatography was attempted first and it was observed that some of the mass was lost during the column. Subjecting the purified product to the same column conditions resulted in the same loss of mass. It was found that the addition of 1% NEt_3 to the column resulted in slightly improved mass recovery. It was also found that using neutral alumina instead of silica resulted in improved mass recovery, but in some cases the separation was not as good. Reversed phase silica sometimes resulted in better separation and improved mass recovery compared to normal silica. Therefore, either alumina or reverse phase silica were used for most compounds.

Methyl 5-(4-methylquinolin-2-yl)bicyclo[3.1.1]heptane-1-carboxylate (3a)

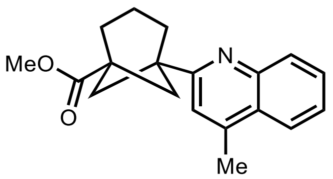

The product was prepared by **General Procedure 2** from **2a** (343 mg, 1.00 mmol, 1.0 equiv.), and lepidine (264 μL, 2.00 mmol, 2.0 equiv.), then purified by reverse phase column chromatography (C_{18} reversed phase silica, water/MeCN 1:9) to give the title compound (159 mg, 0.538 mmol, 54%) as a yellow oil.

R_f 0.41 (7:3 pentane / EtOAc).

^{1}H NMR (400 MHz, $CDCl_3$) δ_H 8.06 (1H, d, *J* = 8.5 Hz), 7.94 (1H, d, *J* = 8.3 Hz), 7.66 (1 H, dd, *J* = 8.4, 6.9 Hz), 7.49 (1H, dd, *J* = 8.3, 6.9 Hz), 7.07 (1H, s), 3.67 (3H, s), 2.67 (3H, s), 2.63-2.59 (2H, m), 2.20-2.15 (2H, m), 2.14-2.00 (6H, m).

^{13}C NMR (125 MHz, $CDCl_3$) δ_C 176.3, 166.6, 147.6, 144.6, 129.7, 129.2, 126.9, 125.7, 123.7, 118.8, 51.8, 44.8, 42.5, 39.2, 34.4, 30.0, 19.0, 16.8.

IR (thin film, ν_{max} / cm^{-1}; selected peaks): 2949 (w, C-H), 1731 (s, C=O).

HRMS (ES^+) calc. for $C_{19}H_{21}NO_2Na$ $[M+Na]^+$ 318.1465, found 318.1480, error +4.86 ppm.

Methyl 5-(3-methylisoquinolin-1-yl)bicyclo[3.1.1]heptane-1-carboxylate (3b)

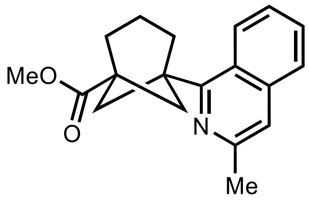

The product was prepared by **General Procedure 2** from **2a** (51.5 mg, 0.150 mmol, 1.0 equiv.) and 3-methylisoquinoline (43.0 mg, 0.300 mmol, 2.0 equiv.), then purified by column chromatography (neutral alumina, pentane/EtOAc 7:3) to give the title compound, containing small traces of catalyst (20.9 mg, 0.0708 mmol, 47%) as a yellow oil.

R_f 0.47 (7:3 pentane / EtOAc).

^{1}H NMR (400 MHz, $CDCl_3$) δ_H 7.98 (1H, d, *J* = 8.6Hz), 7.71 (1H, d, *J* = 8.1 Hz), 7.57 (1H, t, *J* = 7.6 Hz), 7.43 (1H, t, *J* = 7.7 Hz), 7.31 (1H, s), 3.66 (3H, s), 2.76-2.71 (2H, m), 2.64 (3H, s), 2.46-2.41 (2H, m), 2.23-2.06 (6H, m).

^{13}C NMR (125 MHz, $CDCl_3$) δ_C 176.3, 165.6, 150.5, 137.6, 129.5, 127.2, 125.7, 125.5, 123.8, 117.3, 51.8, 46.0, 42.8, 41.1, 34.7, 30.1, 24.5, 17.0.

IR (thin film, ν_{max} / cm^{-1}; selected peaks): 2925 (w, C-H), 1732 (s, C-H).

HRMS (ES^+) calc. for $C_{19}H_{21}NO_2Na$ $[M+Na]^+$ 318.1465, found 318.1461, error -1.11 ppm.

Methyl 5-(isoquinolin-1-yl)bicyclo[3.1.1]heptane-1-carboxylate (3c)

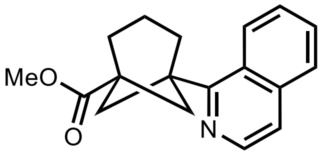

The product was prepared by **General Procedure 2** from **2a** (51.5 mg, 0.150 mmol, 1.0 equiv.) and isoquinoline (35.3 µL, 0.300 mmol, 2.0 equiv.), then purified by reverse phase column chromatography (C_{18} reversed phase silica, water/MeCN 1:9) to give the title compound (18.9 mg, 0.0672 mmol, 45%) as a yellow oil.

R_f 0.55 (6:4 pentane / EtOAc).

^{1}H NMR (400 MHz, $CDCl_3$) δ_H 8.44 (1H, d, J = 5.8 Hz), 8.03 (1H, dq, J = 8.6, 1.0 Hz), 7.82 (1H, d, J = 8.0), 7.64 (1H, ddd, J = 8.2, 6.9, 1.2 Hz), 7.53 (1H, ddd, J = 8.4, 6.9, 1.4 Hz), 7.50 (1H, d, J = 5.8 Hz), 3.66 (3H, s), 2.78-2.73 (2H, m), 2.47-2.42 (2H, m), 2.22-2.07 (6H, m).

^{13}C NMR (125 MHz, $CDCl_3$) δ_C 176.1, 166.3, 141.7, 136.8, 129.8, 127.8, 126.6, 125.8, 125.8, 119.5, 51.8, 46.1, 42.8, 41.0, 34.8, 30.0, 17.0.

IR (thin film, ν_{max} / cm^{-1}; selected peaks): 2950 (w, C-H), 1731 (s, C=O).

HRMS (ES$^+$) calc. for $C_{18}H_{19}NO_2Na$ [M+Na]$^+$ 304.1308, found 304.1309, error +0.31 ppm.

Methyl 5-(4-methylpyridin-2-yl)bicyclo[3.1.1]heptane-1-carboxylate (3d)

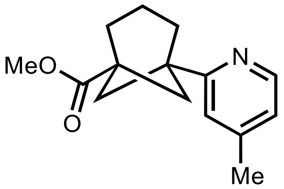

The product was prepared by **General Procedure 2** from **2a** (51.5 mg, 0.150 mmol, 1.0 equiv.) and 4-methylpyridine (29.2 µL, 0.300 mmol, 2.0 equiv.), then purified by column chromatography (neutral alumina, pentane/EtOAc 6:4) to give the title compound (14.5 mg, 0.0591 mmol, 39%) as a yellow oil.

R_f 0.29 (6:4 pentane / EtOAc).

^{1}H NMR (400 MHz, $CDCl_3$) δ_H 8.39 (1H, d, J = 5.1 Hz), 6.93 (1H, d, J = 5.0 Hz), 6.90 (1H, s), 3.66 (3H, s), 2.49-2.44 (2H, m), 2.33 (3H, s), 2.11-2.05 (4H, m), 2.02-1.97 (4H, m).

^{13}C NMR (125 MHz, $CDCl_3$) δ_C 176.3, 166.5, 148.6, 148.1, 122.3, 120.5, 51.8, 44.0, 42.6, 39.3, 34.7, 29.9, 21.3, 16.8.

IR (thin film, ν_{max} / cm^{-1}; selected peaks): 2921 (w, C-H), 1732 (s, C=O).

HRMS (ES$^+$) calc. for $C_{15}H_{19}NO_2Na$ [M+Na]$^+$ 268.1308, found 268.1306, error -0.76 ppm.

Methyl 5-(quinoxalin-2-yl)bicyclo[3.1.1]heptane-1-carboxylate (3e)

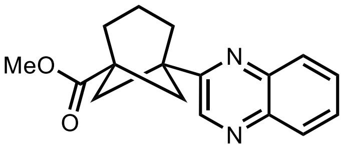

The product was prepared by **General Procedure 2** from **2a** (51.5 mg, 0.150 mmol, 1.0 equiv.) and quinoxaline (34.7 μL, 0.300 mmol, 2.0 equiv.), then purified by reverse phase column chromatography (C_{18} reversed phase silica, water/MeCN 1:9) to give the title compound (14.1 mg, 0.0499 mmol, 33%) as a yellow oil

R_f 0.41 (7:3 pentane / EtOAc).

^{1}H NMR (400 MHz, $CDCl_3$) δ_H 8.73 (1H, s), 8.08-8.04 (2H, m), 7.76-7.69 (2H, m), 3.69 (3H, s), 2.69-2.65 (2H, m), 2.25-2.20 (2H, m), 2.18-2.13 (4H, m), 2.11-2.06 (2H, m).

^{13}C NMR (125 MHz, $CDCl_3$) δ_C 175.9, 161.1, 143.2, 142.0, 141.3, 130.2, 129.3, 129.2, 129.2, 52.0, 43.5, 43.0, 39.1, 34.1, 29.9, 16.7.

IR (thin film, ν_{max} / cm^{-1}; selected peaks): 2925 (w. C-H), 1733 (s, C=O).

HRMS (ES^+) calc. for $C_{17}H_{18}N_2O_2Na$ $[M+Na]^+$ 305.1261, found 305.1263, error +0.81 ppm.

Methyl 5-(pyridin-2-yl)bicyclo[3.1.1]heptane-1-carboxylate and methyl 5-(pyridin-4-yl)bicyclo [3.1.1]heptane-1-carboxylate (3f) and (3f')

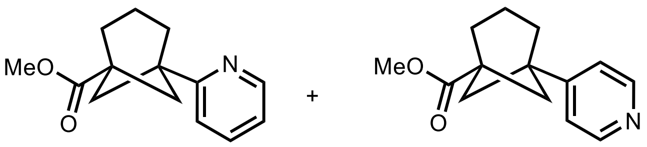

3f **3f'**

The product was prepared by **General Procedure 2** from **2a** (51.5 mg, 0.150 mmol, 1.0 equiv.), and pyridine (24.3 μL, 0.300 mmol, 2.0 equiv.), then purified by reverse phase column chromatography (C_{18} reversed phase silica, water/MeCN 1:9) to give the title compounds as an inseparable mixture of isomers (12.7 mg, 0.0549 mmol, 23%/13%) as a yellow oil.

R_f 0.41, 0.24 (6:4 pentane / EtOAc).

^{1}H NMR (400 MHz, $CDCl_3$) δ_H 8.54-8.52 (3H, m), 7.61 (1H, td, J= 7.7, 1.9 Hz), 7.10-7.07 (2H, m), 7.03 (2H, d, J= 5.2 Hz), 3.67 (3H, s), 3.66 (3H, s), 2.50-2.47 (2H, m), 2.38-2.35 (2H, m), 2.10-1.88 (16H, m).

^{13}C NMR (125 MHz, $CDCl_3$) δ_C 176.3, 175.8, 166.9, 157.9, 149.7, 149.2, 136.6, 121.2, 120.5, 119.5, 51.9, 51.8, 44.3, 42.8, 42.6, 42.2, 39.4, 39.3, 35.9, 34.7, 29.9, 29.7, 16.8, 16.8.

IR (thin film, ν_{max} / cm^{-1}; selected peaks): 2950 (w, C-H), 1731 (s, C=O).

HRMS (ES^+) calc. for $C_{14}H_{18}NO_2$ $[M+H]^+$ 232.1332, found 232.1333, error +0.39 ppm.

Methyl 5-([2,2'-bipyridin]-6-yl)bicyclo[3.1.1]heptane-1-carboxylate (3g)

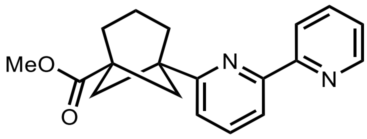

The product was prepared by **General Procedure 2** from **2a** (51.5 mg, 0.150 mmol, 1.0 equiv.) and 2,2'-bipyridine (46.9 mg, 0.300 mmol, 2.0 equiv.), then purified by reverse phase column chromatography (C_{18} reversed phase silica, water/MeCN 1:9) to give the two (separated) products **3g** (8.60 mg, 0.0297 mmol, 19%), **3g'** (4.40 mg, 0.0143 mmol, 10%) as a yellow oils.

R$_f$ 0.55 (6:4 pentane / EtOAc).

^{1}H NMR (400 MHz, $CDCl_3$) δ_H 8.66 (1H, ddd, *J* = 4.8, 1.8, 0.9 Hz), 8.49 (1H, dt, *J* = 8.0, 1.1 Hz), 8.22 (1H, dd, 7.8 Hz, 1.0 Hz), 7.81 (1H, td, *J* = 7.7, 1.8 Hz), 7.74 (1H, t, *J* = 7.8 Hz), 7.29 (1H, ddd, *J* = 7.5, 4.8, 1.3 Hz), 7.13 (1H, dd, *J* = 7.7, 1.0 Hz), 3.69 (3H, s), 2.63-2.59 (2H, m), 2.14-2.07 (6H, m), 2.06-2.00 (2H, m).

^{13}C NMR (125 MHz, $CDCl_3$) δ_C 176.5, 165.9, 156.7, 155.1, 149.1, 137.3, 137.0, 123.7, 121.4, 119.5, 118.3, 51.8, 44.4, 42.7, 39.6, 34.2, 30.1, 16.9.

IR (thin film, ν_{max} / cm^{-1}; selected peaks): 2950 (w, C-H), 1731 (s, C=O).

HRMS (ES$^+$) calc. for $C_{19}H_{20}N_2O_2Na$ [M+Na]$^+$ 331.1417, found 331.1411, error -1.82 ppm.

Methyl 5-([2,2'-bipyridin]-4-yl)bicyclo[3.1.1]heptane-1-carboxylate (3g')

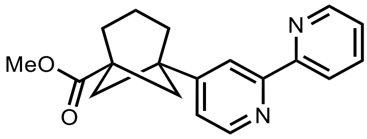

R$_f$ 0.51 (6:4 pentane / EtOAc).

^{1}H NMR (400 MHz, $CDCl_3$) δ_H 8.68 (1H, ddd, *J* = 4.8, 1.8, 1.0 Hz), 8.59 (1H, dd, *J* = 5.0, 0.8 Hz), 8.39 (1H, dt, *J* = 8.0, 1.1 Hz), 8.15 (1H, dd, *J* = 1.8, 0.8 Hz), 7.82 (1H, td, *J* = 7.8, 1.8 Hz), 7.31 (1H, ddd, *J* = 7.5, 4.8, 1.2 Hz), 7.06 (1H, dd, *J* = 5.1, 1.8 Hz), 3.67 (3H, s), 2.48-2.43 (2H, m), 2.17-2.12 (2H, m), 2.11-2.07 (2H, m), 2.04-1.95 (4H, m).

^{13}C NMR (125 MHz, $CDCl_3$) δ_C 175.9, 158.9, 156.3, 156.2, 149.3, 149.2, 137.1, 123.9, 121.5, 120.5, 117.7, 51.9, 42.8, 42.6, 39.6, 35.9, 29.7, 16.8.

IR (thin film, ν_{max} / cm^{-1}; selected peaks): 2951 (w, C-H), 1732 (s, C=O).

HRMS (ES$^+$) calc. for $C_{19}H_{20}N_2O_2Na$ [M+Na]$^+$ 331.1417, found 331.1416, error -0.31 ppm.

Dimethyl 3-(5-(methoxycarbonyl)bicyclo[3.1.1]heptan-1-yl)pyridine-2,6-dicarboxylate (3h)

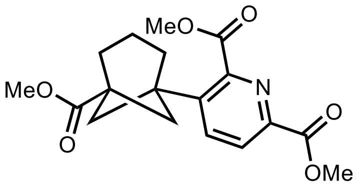

The product was prepared by **General Procedure 2** from **2a** (51.5 mg, 0.150 mmol, 1.0 equiv.) and dimethyl pyridine-2,6-dicarboxylate (58.6 mg, 0.300 mmol, 2.0 equiv.), then purified by column chromatography (neutral alumina, pentane/EtOAc 6:4) to give the title compound (8.00 mg, 0.0230 mmol, 15%) as a yellow oil.

R_f 0.38 (6:4 pentane / EtOAc).

^{1}H NMR (400 MHz, $CDCl_3$) δ_H 8.15 (1H, d, *J* = 8.1 Hz), 7.60 (1H, d, *J* = 8.1 Hz), 3.99 (3H, s), 3.96 (3H, s), 3.65 (3H, s), 2.32-2.27 (2H, m), 2.20-2.16 (2H, m), 2.10-1.94 (6H, m).

^{13}C NMR (125 MHz, $CDCl_3$) δ_C 175.6, 167.0, 165.2, 148.1, 147.5, 145.4, 136.6, 126.6, 53.2, 53.2, 52.0, 42.7, 41.9, 39.7, 35.7, 29.6, 16.8.

IR (thin film, ν_{max} / cm^{-1}; selected peaks): 2953 (w, C-H), 1736 (s, C=O).

HRMS (ES$^+$) calc. for $C_{18}H_{22}NO_6$ [M+H]$^+$ 348.1442, found 348.1455, error +3.82 ppm.

2-(5-(4-Fluorophenyl)bicyclo[3.1.1]heptan-1-yl)-4-methylquinoline (3i)

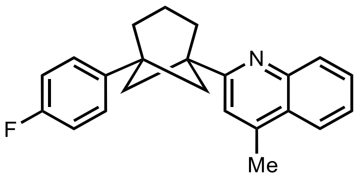

The product was prepared by **General Procedure 2** from **2b** (29.2 mg, 77.0 µmol, 1.0 equiv.) and lepidine (20.4 µL, 0.154 mmol, 2.0 equiv.), then purified by reverse phase column chromatography (C_{18} reversed phase silica, water/MeCN 1:9) to give the title compound (18.6 mg, 56.1 µmol, 73%) as a yellow oil.

R_f 0.46 (9:1 pentane / EtOAc).

^{1}H NMR (400 MHz, $CDCl_3$) δ_H 8.07 (1H, d, *J* = 8.4 Hz), 7.94 (1H, d, *J* = 9.1 Hz), 7.66 (1H, ddd, *J* = 8.4, 6.8, 1.4 Hz), 7.49 (1H, ddd, *J* = 8.2, 6.9, 1.3 Hz), 7.16-7.11 (3H, m), 7.00-6.94 (2H, m), 2.68 (3H, s), 2.45-2.40 (4H, m), 2.16-2.07 (4H, m), 2.02-1.98 (2H, m).

^{13}C NMR (101 MHz, $CDCl_3$) δ_C 167.4, 161.1 (d, *J* = 243.0 Hz), 147.7, 146.2 (d, *J* = 3.1 Hz), 144.4, 129.8, 129.1, 126.8, 126.6 (d, *J* = 7.8 Hz), 125.6, 123.7, 119.0, 115.0 (d, *J* = 20.9 Hz), 45.0, 42.1, 41.1, 37.6, 34.7, 18.9, 17.7.

^{19}F NMR (377 MHz, $CDCl_3$) δ_F -118.1.

IR (thin film, ν_{max} / cm^{-1}; selected peaks): 2945 (w, C-H), 2858 (w, C-H).

HRMS (ES$^+$) calc. for $C_{23}H_{23}FN$ [M+H]$^+$ 332.1809, found 332.1819, error +2.99 ppm.

4-Methyl-2-(5-(p-tolyl)bicyclo[3.1.1]heptan-1-yl)quinoline (3j)

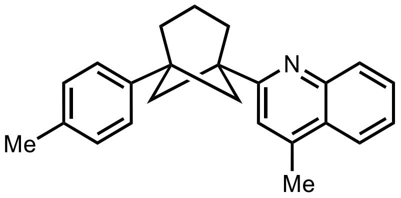

The product was prepared by **General Procedure 2** from **2c** (26.1 mg, 69.5 µmol, 1.0 equiv.) and lepidine (18.4 µL, 0.139 mmol, 2.0 equiv.), then purified by reverse phase column chromatography (C_{18} reversed phase silica, water/MeCN 1:9) to give the title compound (12.7 mg, 38.8 µmol, 56%) as a yellow oil.

R_f 0.31 (9:1 pentane / EtOAc).

^{1}H NMR (400 MHz, $CDCl_3$) δ_H 8.09 (1H, d, *J* = 8.4 Hz), 7.94 (1H, d, *J* = 8.4 Hz), 7.66 (1H, ddd, *J* = 8.4, 6.8, 1.4 Hz), 7.49 (1H, ddd, *J* = 8.2, 6.9, 1.3 Hz), 7.14-7.08 (5H, m), 2.68 (3H, d, *J* = 1.0 Hz), 2.47-2.40 (4H, m), 2.32 (3H, s), 2.18-2.01 (6H, m).

^{13}C NMR (101 MHz, $CDCl_3$) δ_C 167.6, 147.5, 135.0, 129.7, 129.1, 129.0, 126.8, 125.6, 125.0, 123.7, 119.1, 45.1, 42.2, 41.2, 37.5, 34.8, 21.1, 19.0, 17.8.

The missing ^{13}C peaks correlate with two of the quaternary carbons in the quinoline ring. In other Minisici products these peaks are consistently of low intensity and/or broad (at around 144 and 147 ppm). For this compound they are almost broadened into the baseline.

IR (thin film, ν_{max} / cm^{-1}; selected peaks): 2942 (w, C-H), 2858 (w, C-H).

HRMS (ES$^+$) calc. for $C_{24}H_{26}N$ $[M+H]^+$ 328.2060, found 328.2067, error +2.20 ppm.

4-Methyl-2-(5-(4-(trifluoromethyl)benzyl)bicyclo[3.1.1]heptan-1-yl)quinoline (3k)

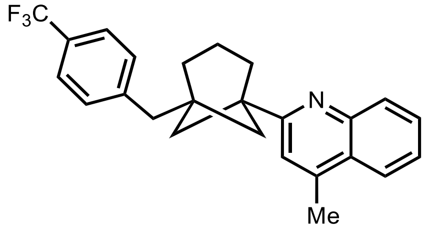

The product was prepared by **General Procedure 2** from **2d** (19.4 mg, 43.8 µmol, 1.0 equiv.) and lepidine (11.6 µL, 87.5 µmol, 2.0 equiv.), then purified by reverse phase column chromatography (C_{18} reversed phase silica, water/MeCN 1:9) to give the title compound (6.9 mg, 17.4 µmol, 40%) as a yellow oil

R_f 0.25 (9:1 pentane / EtOAc).

^{1}H NMR (400 MHz, $CDCl_3$) δ_H 8.08 (1H, d, *J* = 8.5 Hz), 7.93 (1H, d, *J* = 8.3 Hz), 7.66 (1H, ddd, *J* = 8.3, 6.7, 1.5 Hz), 7.53-7.47 (3H, m), 7.24 (2H, d, *J* = 7.9 Hz), 7.05 (1H, s), 2.75 (2H, s), 2.67 (3H, s), 2.14-2.09 (2H, m), 2.04-2.00 (4H, m), 1.95-1.88 (2H, m), 1.68 (2H, t, *J* = 7.1 Hz).

^{13}C NMR (101 MHz, $CDCl_3$) δ_C 167.7, 147.6, 144.4, 143.4, 130.0, 129.7, 129.1, 128.3 (q, *J* = 31.6 Hz), 126.8, 125.6, 125.1 (q, *J* = 3.8 Hz), 124.5 (q, *J* = 271.5 Hz), 123.7, 119.1, 47.4, 45.6, 40.9, 39.5, 35.3, 33.1, 18.9, 17.4.

^{19}F NMR (377 MHz, $CDCl_3$) δ_F -62.3.

IR (thin film, ν_{max} / cm^{-1}; selected peaks): 2943 (w, C-H).

HRMS (ES$^+$) calc. for $C_{25}H_{25}F_3N$ $[M+H]^+$ 396.1934, found 396.1938, error +1.10 ppm.

2-(5-(3,5-Bis(trifluoromethyl)benzyl)bicyclo[3.1.1]heptan-1-yl)-4-methylquinoline (3l)

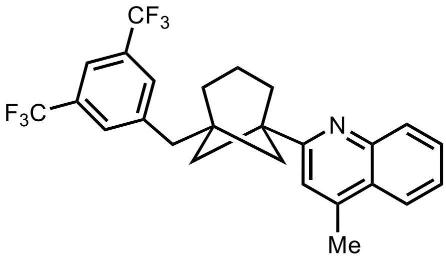

The product was prepared by **General Procedure 2** from **2e** (76.7 mg, 0.150 mmol, 1.0 equiv.) and lepidine (37.9 μL, 0.300 mmol, 2.0 equiv.), then purified by reverse phase column chromatography (C_{18} reversed phase silica, water/MeCN 1:9) to give the title compound (14.7 mg, 31.7 μmol, 21%) as a yellow oil.

R$_f$ 0.26 (9:1 pentane / EtOAc).

^{1}H NMR (400 MHz, $CDCl_3$) δ_H 8.07 (1H, d, *J* = 8.5 Hz), 7.94 (1H, d, *J* = 8.3 Hz), 7.73 (1H, s), 7.67 (1H, ddd, *J* = 8.4, 6.9, 1.5 Hz), 7.57 (2H, s), 7.50 (1H, ddd, *J* = 8.2, 6.9, 1.3 Hz), 7.06 (1H, s), 2.83 (2H, s), 2.68 (3H, d, *J* = 1.0 Hz), 2.17-2.12 (2H, m), 2.06-2.00 (4H, m), 1.96-1.89 (2H, m), 1.65 (2H, t, *J* = 7.1 Hz).

^{13}C NMR (101 MHz, $CDCl_3$) δ_C 167.3, 147.7, 144.5, 147.7, 131.4 (q, *J* = 32.9 Hz), 129.8, 129.6 (q, *J* = 3.1 Hz), 129.1, 126.9, 125.6, 123.7, 123.7 (q, *J* = 272.3), 120.2 (sept, *J* = 3.8 Hz), 119.0, 47.3, 45.7, 40.9, 39.3, 35.3, 32.9, 19.0, 17.4.

^{19}F NMR (377 MHz, $CDCl_3$) δ_F -62.7.

IR (thin film, ν_{max} / cm^{-1}; selected peaks): 2947 (w, C-H).

HRMS (ES$^+$) calc. for $C_{26}H_{24}F_6N$ $[M+H]^+$ 464.1807, found 464.1814, error +1.41 ppm.

4-Methyl-2-(5-(trifluoromethyl)bicyclo[3.1.1]heptan-1-yl)quinoline (3m)

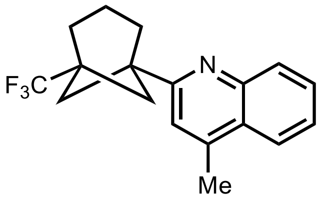

The product was prepared by **General Procedure 2** from **2f** (42.4 mg, 0.120 mmol, 1.0 equiv.) and lepidine (31.7 μL, 0.240 mmol, 2.0 equiv.), then purified by reverse phase column chromatography (C_{18} reversed phase silica, water/MeCN 1:9) to give the title compound (19.4 mg, 63.5 μmol, 53%) as a yellow oil.

R$_f$ 0.33 (7:3 pentane / EtOAc).

^{1}H NMR (400 MHz, $CDCl_3$) δ_H 7.99 (1H, d, *J* = 8.5 Hz), 7.88 (1H, d, *J* = 8.3 Hz), 7.60 (1H, ddd, *J* = 8.4, 6.9, 1.5 Hz), 7.44 (1H, ddd, *J* = 8.3, 6.7, 1.3 Hz), 6.99 (1H, s), 2.61 (3H, s), 2.45-2.40 (2H, m), 2.06-1.95 (8H, m).

^{13}C NMR (126 MHz, $CDCl_3$) δ_C 165.9, 147.5, 145.1, 129.7, 129.4, 127.0 (q, *J* = 276.4 Hz), 126.9, 125.9, 123.7, 188.6, 44.5, 41.2 (q, *J* = 30.4 Hz), 35.5 (q, *J* = 2.5 Hz), 34.5, 26.6 (q, *J* = 2.6 Hz), 19.0, 16.5.

^{19}F NMR (377 MHz, $CDCl_3$) δ_F -78.2.

IR (thin film, ν_{max} / cm^{-1}; selected peaks): 2956 (w, C-H).

HRMS (ES$^+$/ES$^-$) Not found.

(4-(6-Fluorobenzo[d]isoxazol-3-yl)piperidin-1-yl)(5-(4-methylquinolin-2-yl)bicyclo[3.1.1]heptan-1-yl)methanone (3n)

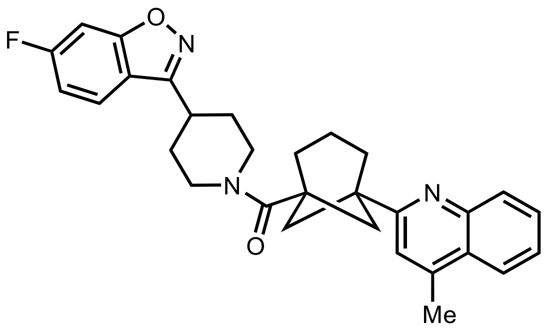

The product was prepared by **General Procedure 2** from **2g** (58.6 mg, 0.110 mmol, 1.0 equiv.) and lepidine (29.2 µL, 0.220 mmol, 2.0 equiv.), then purified by column chromatography (neutral alumina, pentane/EtOAc 2:8) to give the title compound (15.4 mg, 31.8 µmol, 30%) as a white foam.

R_f 0.24 (2:8 pentane / EtOAc).

^{1}H NMR (400 MHz, $CDCl_3$) δ_H 8.05 (1H, d, *J* = 8.4 Hz), 7.95 (1H, dd, *J* = 8.4, 1.4 Hz), 7.66 (1H, ddd, *J* = 8.4, 6.8, 1.5 Hz), 7.62 (1H, dd, *J* = 8.7, 5.1 Hz), 7.50 (1H, ddd, *J* = 8.3, 6.8, 1.3 Hz), 7.25 (1H, dd, *J* = 8.4, 2.1 Hz), 7.12 (1H, s), 7.07 (1H, td, *J* = 8.8, 2.2 Hz), 4.62 (1H, d, *J* = 13.3 Hz), 4.06 (1H, d, *J* = 11.4 Hz), 3.33 (1H, tt, *J* = 11.2, 3.9 Hz), 3.25 (1H, t, *J* = 12.5 Hz), 2.90 (1H, t, *J* = 13.0 Hz), 2.68 (3H, s), 2.62 (2H, d, *J* = 9.0 Hz), 2.33-1.74 (12H, m).

^{13}C NMR (101 MHz, $CDCl_3$) δ_C 175.1, 166.5, 164.7 (d, *J* = 152.1 Hz), 164.1 (d, *J* = 13.6 Hz), 160.4, 129.7, 129.2, 126.9, 125.8, 123.7, 122.3 (d, *J* = 11.2 Hz), 118.9, 117.2 (d, *J* = 1.2 Hz), 112.8 (d, *J* = 25.4 Hz), 97.7 (d, *J* = 26.8 Hz), 45.3, 44.1, 43.6, 42.0, 40.7, 40.2, 34.5, 34.5, 31.4, 30.8, 30.5, 19.0, 17.0.

^{19}F NMR (377 MHz, $CDCl_3$) δ_F -109.1.

IR (thin film, ν_{max} / cm^{-1}; selected peaks): 2950 (w, C-H), 1616 (s, C=O).

HRMS (ES$^+$) calc. for $C_{30}H_{30}FN_3O_2Na$ $[M+Na]^+$ 506.2214, found 506.2237, error +4.48 ppm.

***tert*-Butyl 1-(4-methylquinolin-2-yl)-3-azabicyclo[3.1.1]heptane-3-carboxylate (7a)**

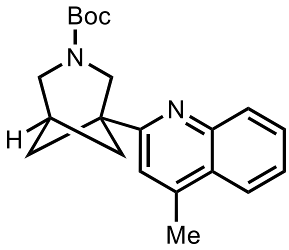

The product was prepared by **General Procedure 2** from **6a** (50.0 mg, 0.129 mmol, 1.0 equiv.) and 4-methyquinoline (34 μL, 37.1 mg, 0.259 mmol, 2.0 equiv.), then purified by column chromatography (5 g SiO_2, heptanes/EtOAc 10-45% over 15 CV) to give the title compound (27 mg, 80.0 μmol, 62%) as a white amorphous solid.

1H NMR (400 MHz, DMSO-d6, 90 °C) δ 8.05 (dd, J = 8.4, 1.4 Hz, 1H), 7.96 (dd, J = 8.5, 1.3 Hz, 1H), 7.76 – 7.65 (m, 1H), 7.62 – 7.48 (m, 1H), 7.33 (s, 1H), 3.72 (s, 2H), 3.63 – 3.51 (m, 2H), 2.69 (s, 3H), 2.52-2.47 (m, 1H), 2.47 – 2.31 (m, 2H), 1.89 – 1.73 (m, 2H), 1.45 (s, 9H).

13C NMR (101 MHz, CDCl3) δ 164.1, 163.5, 156.4, 147.7, 144.8, 129.9, 129.8, 129.4, 129.2, 127.0, 126.0, 125.9, 123.7, 123.7, 119.0, 118.9, 79.5, 79.4, 54.8, 54.0, 49.9, 49.4, 46.9, 46.7, 35.4, 35.4, 28.7, 28.7, 28.3, 28.2, 19.0, 18.9.

A mixture of rotamers was observed in the ^{13}C NMR at 27 °C.

HRMS (ES^+) calc. for $C_{21}H_{27}N_2O_2$ $[M+H]^+$ 339.2067, found 339.2077, error +2.92 ppm.

***tert*-Butyl 1-(4-methylpyridin-2-yl)-3-azabicyclo[3.1.1]heptane-3-carboxylate (7b)**

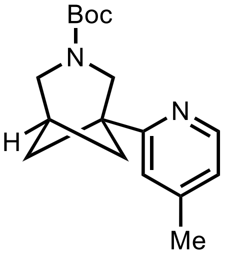

The product was prepared by **General Procedure 2** from **6a** (50.0 mg, 0.129 mmol, 1.0 equiv.) and 4-methypyridine (25 μL, 24.1 mg, 0.259 mmol, 2.0 equiv.), then purified by HPLC purification on a Phenomenex C18(2) 5 micron column (250 x 50 mm) (1 injection). A gradient of acetonitrile (A) and 10 mM ammonium acetate in water (B) was used, at a flow rate of 90 mL/min (0-5.0 min 5% A, 5.0-30.0 min linear gradient 5-95% A, then 95% A hold for 5 min) to give the title compound (13.0 mg, 0.045 mmol, 35%) as a colourless amorphous solid.

1H NMR (400 MHz, DMSO-d6, 90 °C) δ 8.37 (d, J = 5.0 Hz, 1H), 7.13 (s, 1H), 7.10 (d, J = 5.1 Hz, 1H), 3.60 (s, 2H), 3.56 – 3.46 (m, 2H), 2.40 – 2.34 (m, 1H), 2.34 (s, 3H), 2.28 (tt, J = 7.3, 3.6 Hz, 2H), 1.81 – 1.69 (m, 2H), 1.44 (s, 9H).

13C NMR (101 MHz, CDCL3) δ 164.2, 163.6, 156.3, 149.0, 148.9, 147.8, 147.7, 122.7, 121.0, 120.8, 79.5, 79.4, 55.0, 54.4, 49.7, 49.3, 46.2, 46.0, 35.3, 35.3, 28.7, 28.4, 28.2, 21.2, 21.2.

A mixture of rotamers was observed in the ^{13}C NMR at 27 °C.

HRMS (ES^+) calc. for $C_{17}H_{24}N_2O_2Na$ $[M+Na]^+$ 311.1730, found 311.1737, error +2.24 ppm.

tert-Butyl 1-(phthalazin-1-yl)-3-azabicyclo[3.1.1]heptane-3-carboxylate (7c)

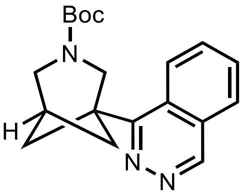

The product was prepared by **General Procedure 2** from **6a** (50.0 mg, 0.129 mmol, 1.0 equiv.) and phthalazine (33.7 mg, 0.259 mmol, 2.0 equiv.), then purified by column chromatography (5 g SiO_2, heptanes/EtOAc 10-100% over 15 CV) to give the title compound (23 mg, 71 µmol, 55%) as a white amorphous solid.

1H NMR (400 MHz, Chloroform-d) δ 9.42 (d, J = 7.0 Hz, 1H), 8.13 – 8.04 (m, 1H), 8.03 – 7.93 (m, 1H), 7.93 – 7.80 (m, 2H), 3.87 (d, J = 13.6 Hz, 2H), 3.71 (dd, J = 9.0, 2.5 Hz, 2H), 2.76 – 2.52 (m, 3H), 2.41 – 2.20 (m, 2H), 1.46 (d, J = 35.4 Hz, 9H).

13C NMR (101 MHz, CDCL3) δ 162.2, 161.9, 156.1, 156.0, 150.6, 132.3, 132.3, 132.0, 132.0, 127.5, 127.4, 127.0, 126.9, 124.8, 124.7, 124.7, 124.4, 79.7, 79.6, 54.4, 53.9, 49.8, 49.3, 46.7, 46.6, 37.1, 36.9, 29.2, 29.1, 28.6, 28.5.

A mixture of ca 1:1 rotamers was observed at 27 °C

HRMS (ES^+) calc. for $C_{19}H_{24}N_3O_2$ $[M+H]^+$ 326.1863, found 326.1868, error +1.51 ppm.

3-(t*ert*-Butyl) 1-methyl 5-(4-methylquinolin-2-yl)-3-azabicyclo[3.1.1]heptane-1,3-dicarboxylate (7d)

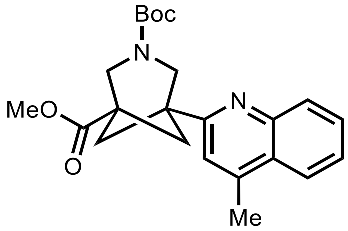

The product was prepared by **General Procedure 2** from **6b** (76.0 mg, 0.171 mmol, 1.0 equiv.) and 4-methylquinoline (45 µL, 49.0 mg, 0.342 mmol, 2.0 equiv.), then purified by column chromatography (10 g SiO_2, heptanes/EtOAc 5-20% over 15 CV) followed by purification by HPLC purification on a Phenomenex C18(2) 5 micron column (250 x 50 mm) (1 injection). A gradient of acetonitrile (A) and 10 mM ammonium acetate in water (B) was used, at a flow rate of 90 mL/min (0-5.0 min 5% A, 5.0-30.0 min linear gradient 5-95% A, then 95% A hold for 5 min) to give the title compound (31 mg, 78 µmol, 46%) as a white amorphous solid.

1H NMR (400 MHz, Chloroform-d) δ 8.06 (dd, J = 8.4, 4.7 Hz, 1H), 7.96 (ddd, J = 8.4, 6.9, 1.5 Hz, 1H), 7.74 – 7.62 (m, 1H), 7.59 – 7.44 (m, 1H), 7.13 (d, J = 6.3 Hz, 1H), 3.87 – 3.66 (m, 7H), 2.83 – 2.72 (m, 2H), 2.69 (d, J = 12.2 Hz, 3H), 2.26 – 2.10 (m, 2H), 1.49 (d, J = 24.4 Hz, 9H).

A mixture of ca 1:1 rotamers was observed at 27 °C

1H NMR (400 MHz, DMSO-d6, 90 °C) δ 8.07 (dd, J = 8.5, 1.4 Hz, 1H), 7.97 (dd, J = 8.6, 1.3 Hz, 1H), 7.73 (ddd, J = 8.4, 6.8, 1.4 Hz, 1H), 7.59 (ddd, J = 8.2, 6.9, 1.3 Hz, 1H), 7.36 (s, 1H), 3.77 – 3.63 (m, 7H), 2.80 – 2.71 (m, 2H), 2.71 (d, J = 1.0 Hz, 3H), 2.13 – 2.02 (m, 2H), 1.46 (s, 9H).

13C NMR (101 MHz, CDCl3) δ 173.6, 173.4, 162.6, 162.0, 156.0, 147.6, 145.2, 129.9, 129.8, 129.5, 129.4, 127.1, 126.2, 126.2, 123.8, 123.7, 118.8, 118.8, 80.1, 80.0, 53.8, 53.1, 52.2, 52.2, 49.8, 49.4, 43.6, 43.4, 41.2, 41.0, 38.5, 38.4, 28.7, 28.6, 19.0, 18.9.

A mixture of ca 1:1 rotamers was observed at 27 °C

MS (ES^+) calc. for $C_{23}H_{28}N_2O_4$ $[M+H]^+$ 396.20, found 397.00

HRMS (ES^+) calc. for $C_{23}H_{29}N_2O_4$ $[M+H]^+$ 397.2122, found 397.2127, error +1.29 ppm.

NMR Spectra

Dimethyl cyclohexane-1,3-dicarboxylate ^{1}**H NMR (400 MHz, CDCl$_3$)**

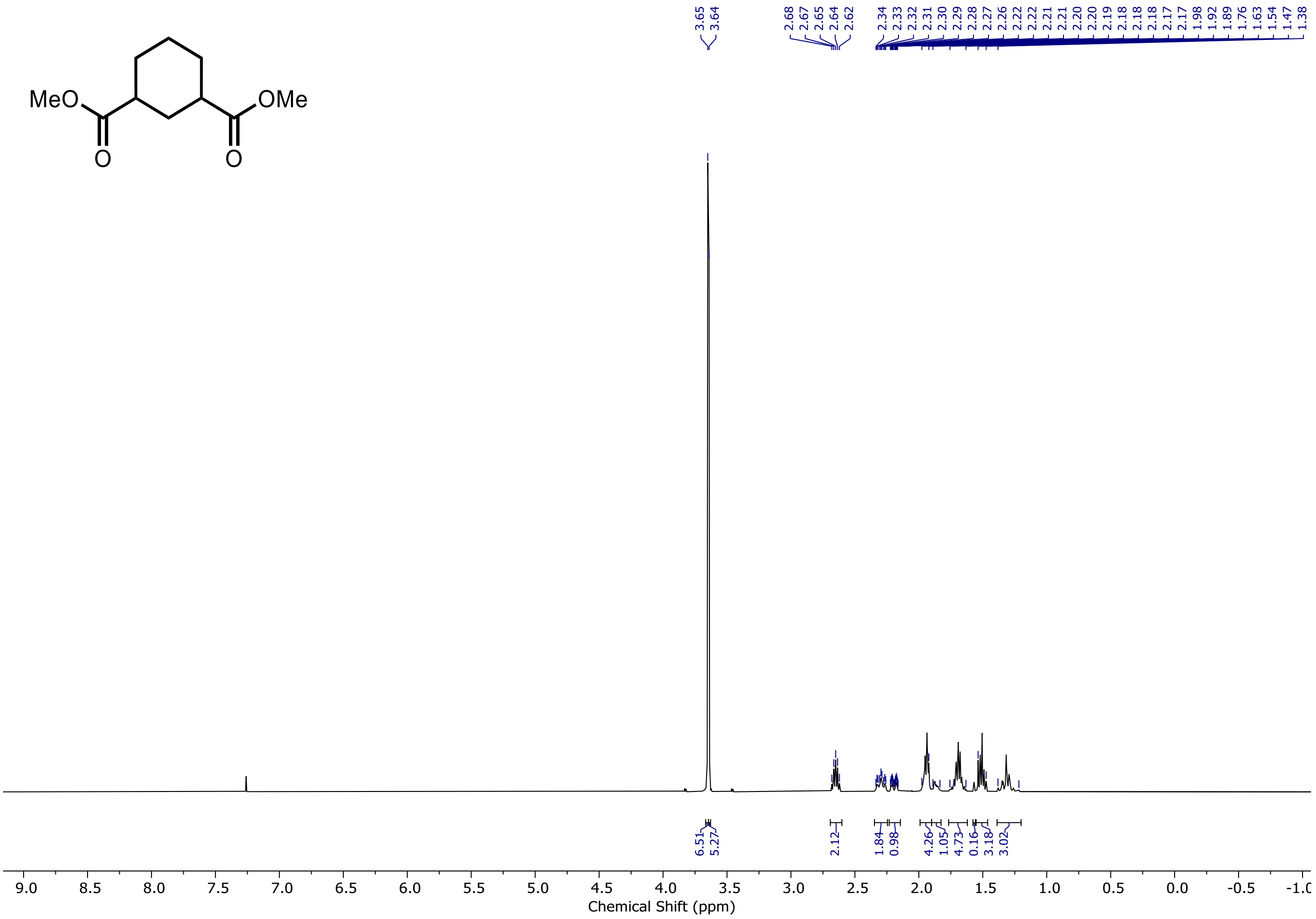

Dimethyl cyclohexane-1,3-dicarboxylate ^{13}**C NMR (101 MHz, CDCl$_3$)**

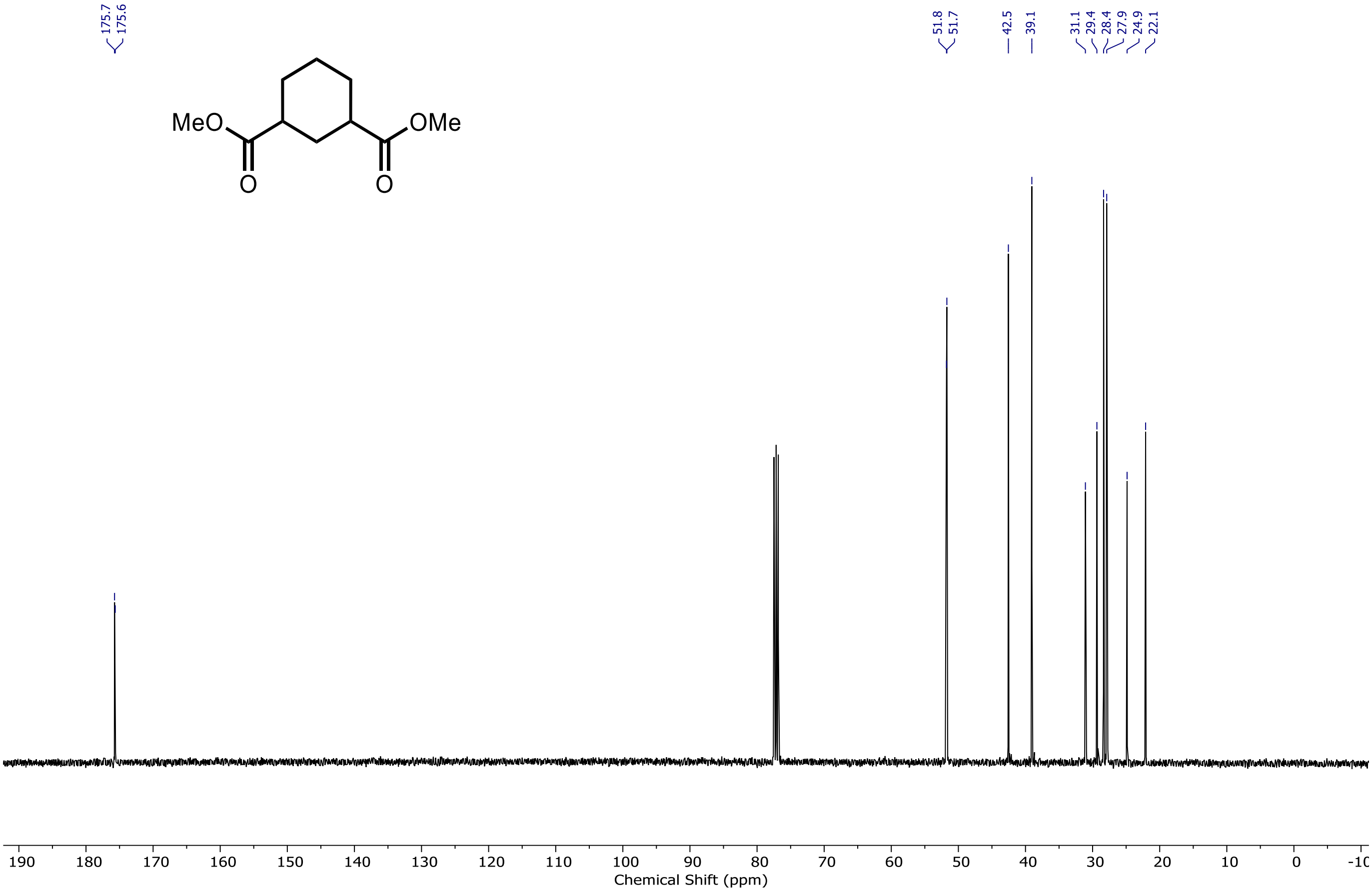

Dimethyl bicyclo[3.1.1]heptane-1,5-dicarboxylate (**4**) **^{1}H NMR (400 MHz, CDCl$_3$)**

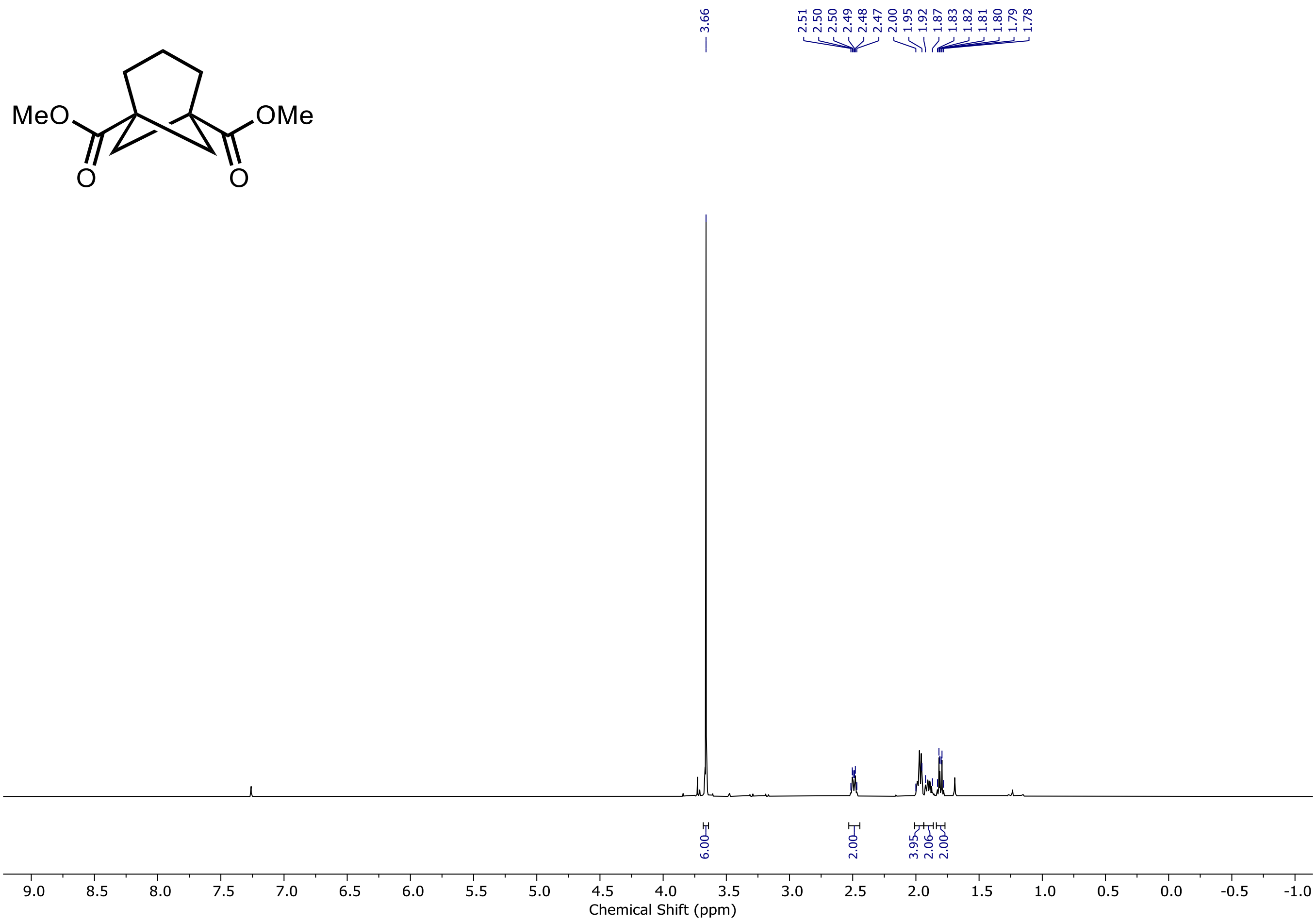

Dimethyl bicyclo[3.1.1]heptane-1,5-dicarboxylate (**4**) **^{13}C NMR (101 MHz, CDCl$_3$)**

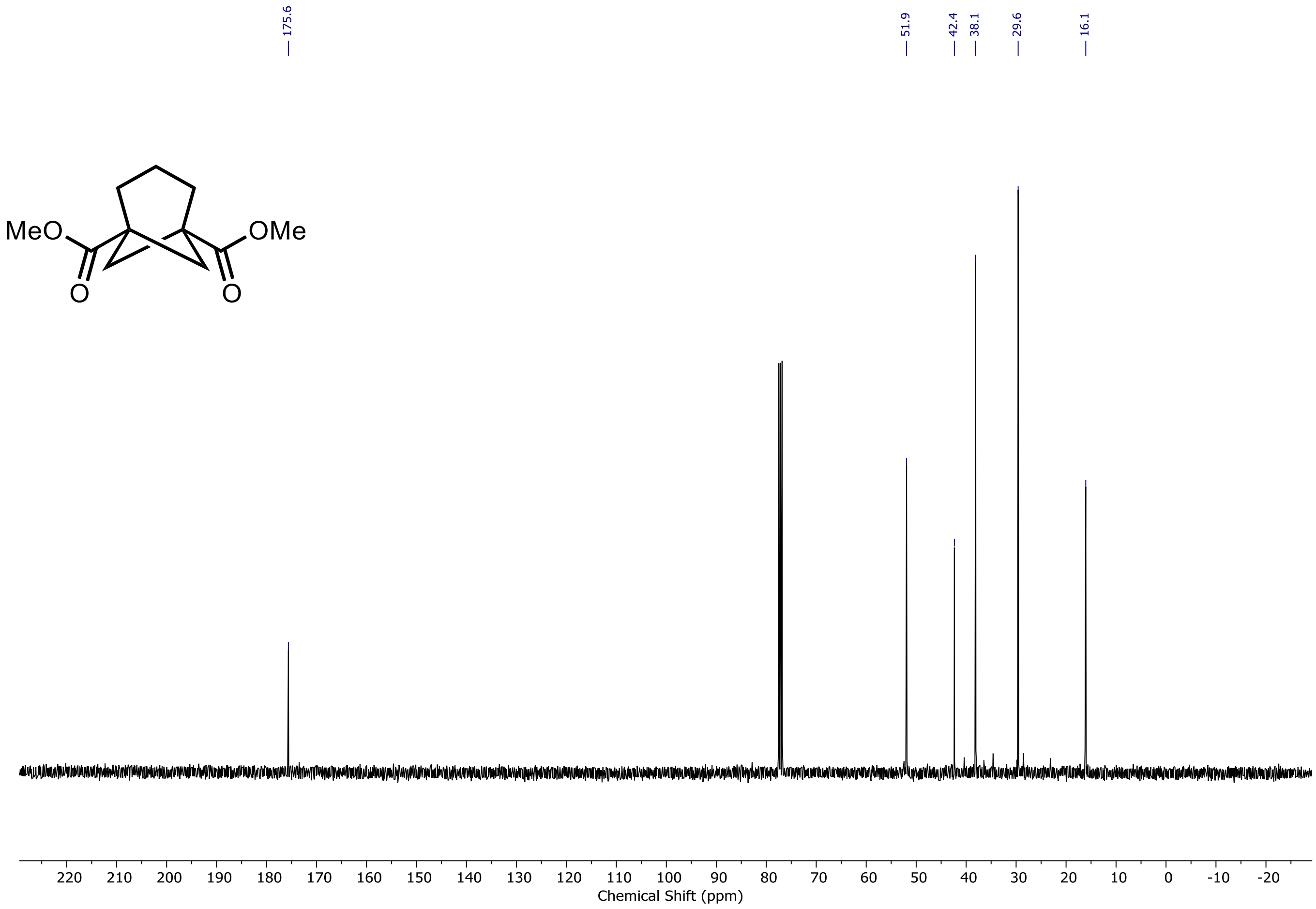

5-(Methoxycarbonyl)bicyclo[3.1.1]heptane-1-carboxylic acid **(5)** **^{1}H NMR (400 MHz, CDCl$_3$)**

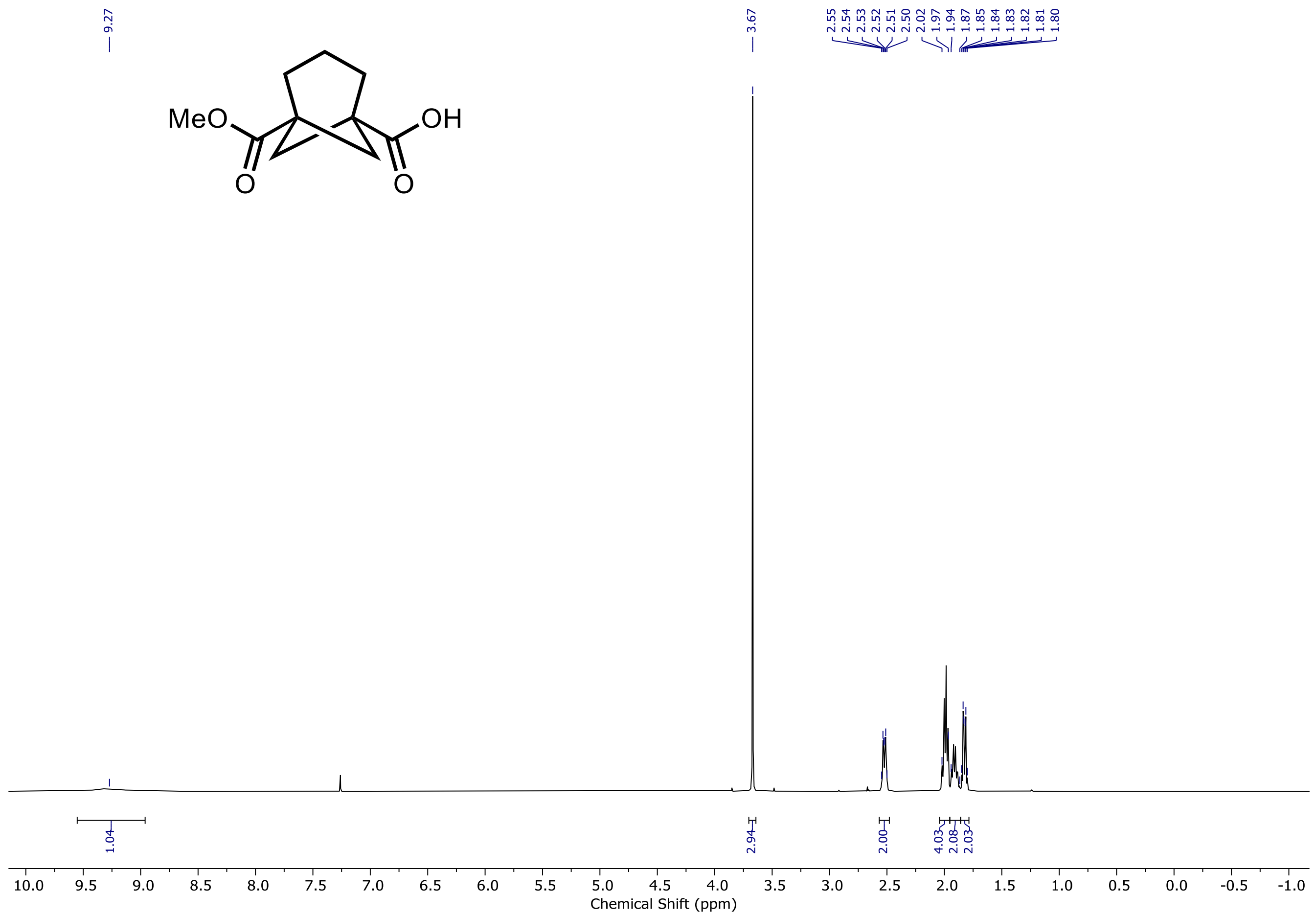

5-(Methoxycarbonyl)bicyclo[3.1.1]heptane-1-carboxylic acid **(5)** **^{13}C NMR (101 MHz, CDCl$_3$)**

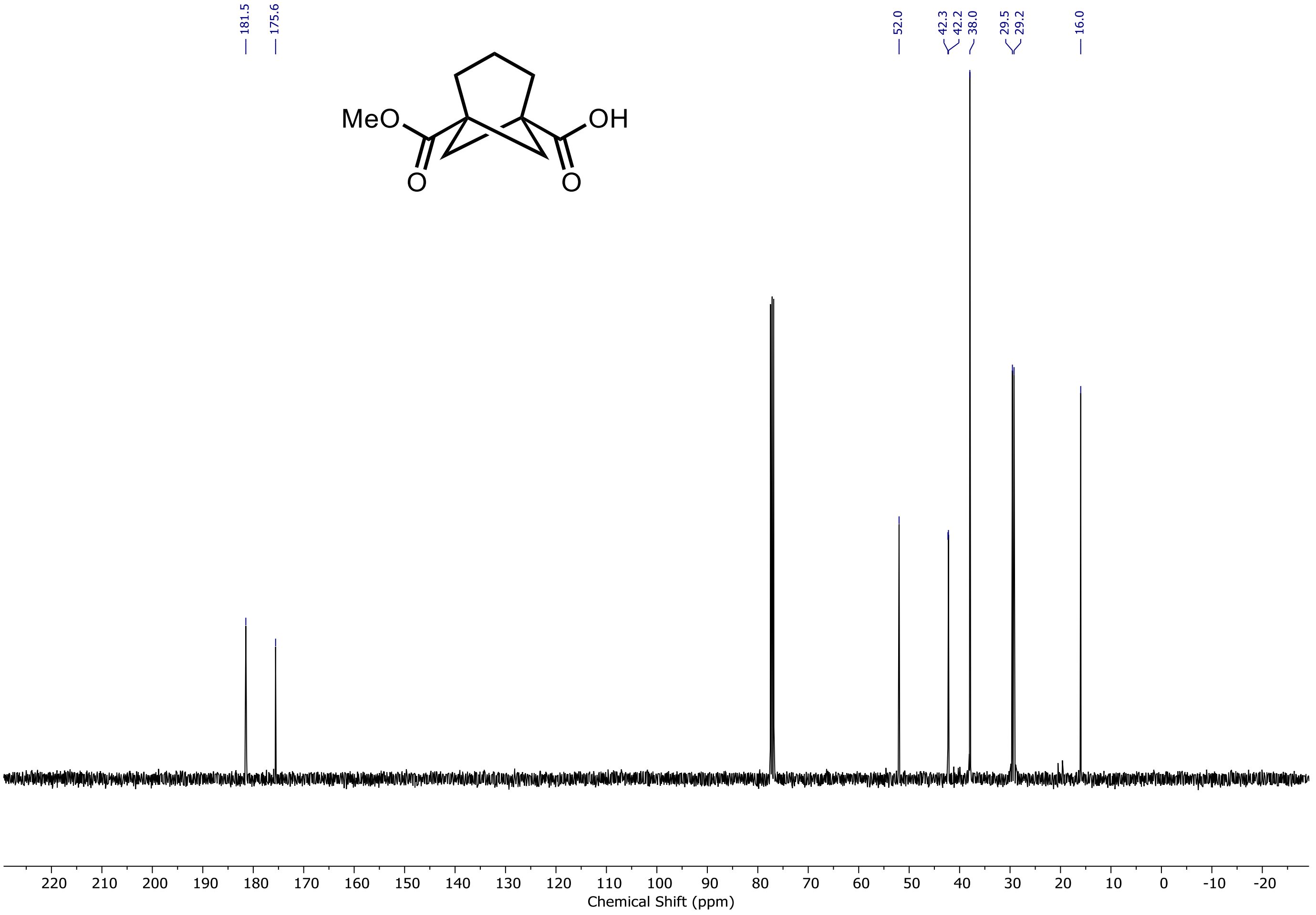

1-(1,3-Dioxoisoindolin-2-yl) 5-methyl bicyclo[3.1.1]heptane-1,5-dicarboxylate (**2a**) **^{1}H NMR (400 MHz, CDCl$_3$)**

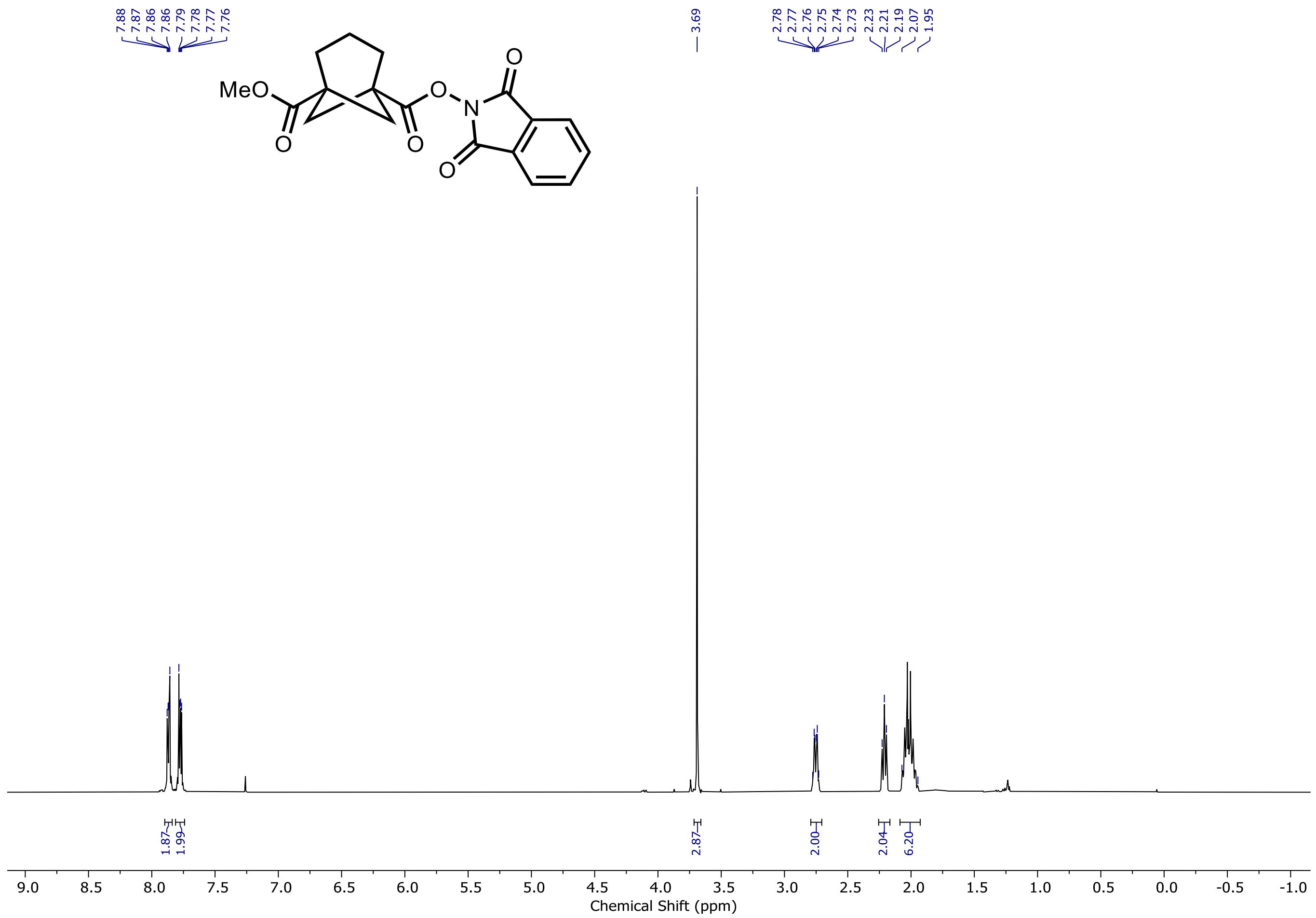

1-(1,3-Dioxoisoindolin-2-yl) 5-methyl bicyclo[3.1.1]heptane-1,5-dicarboxylate (**2a**) **^{13}C NMR (101 MHz, CDCl$_3$)**

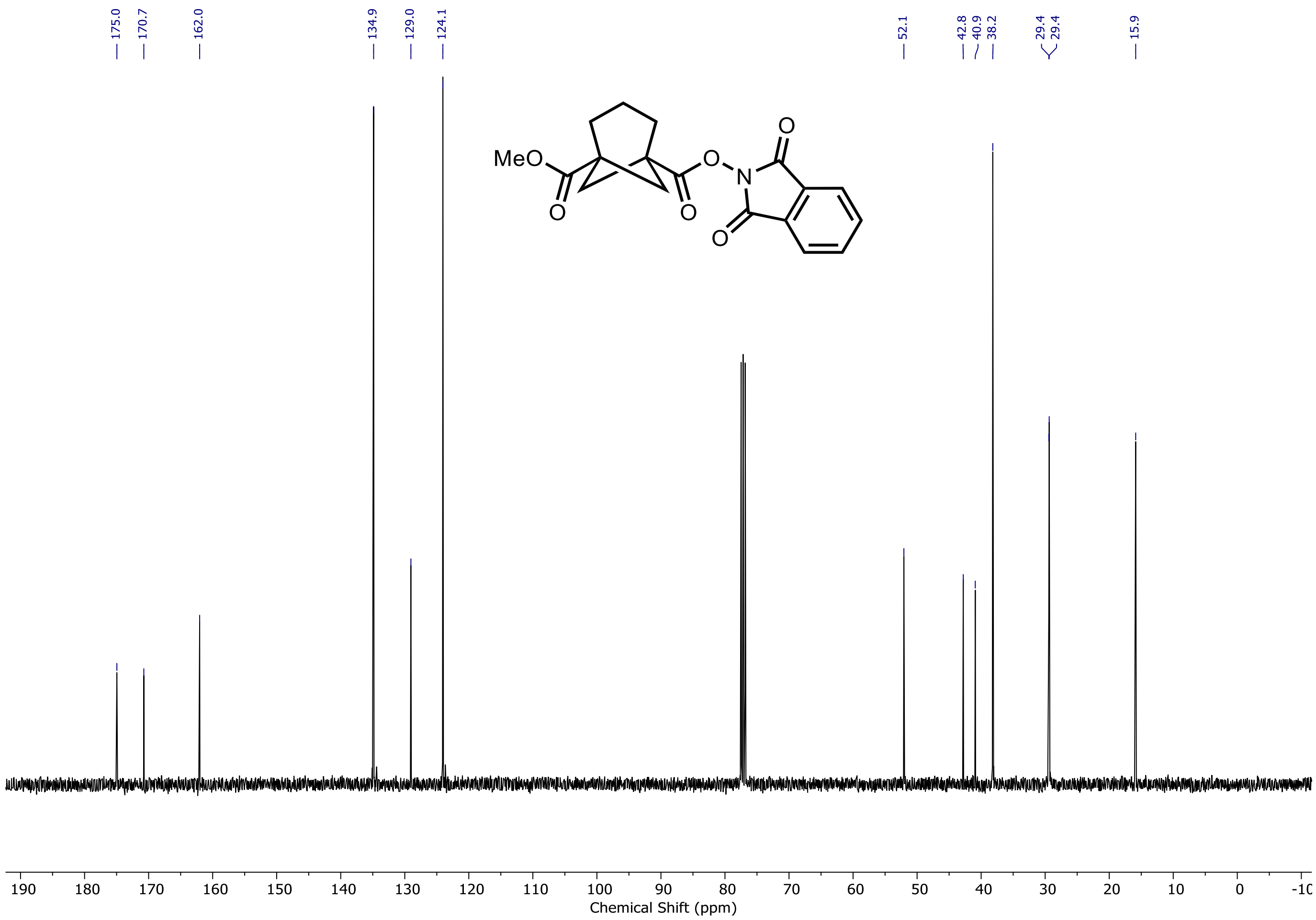

(5-Iodobicyclo[3.1.1]heptan-1-yl)methyl pivalate **(8a)** **^{1}H NMR (400 MHz, CDCl$_3$)**

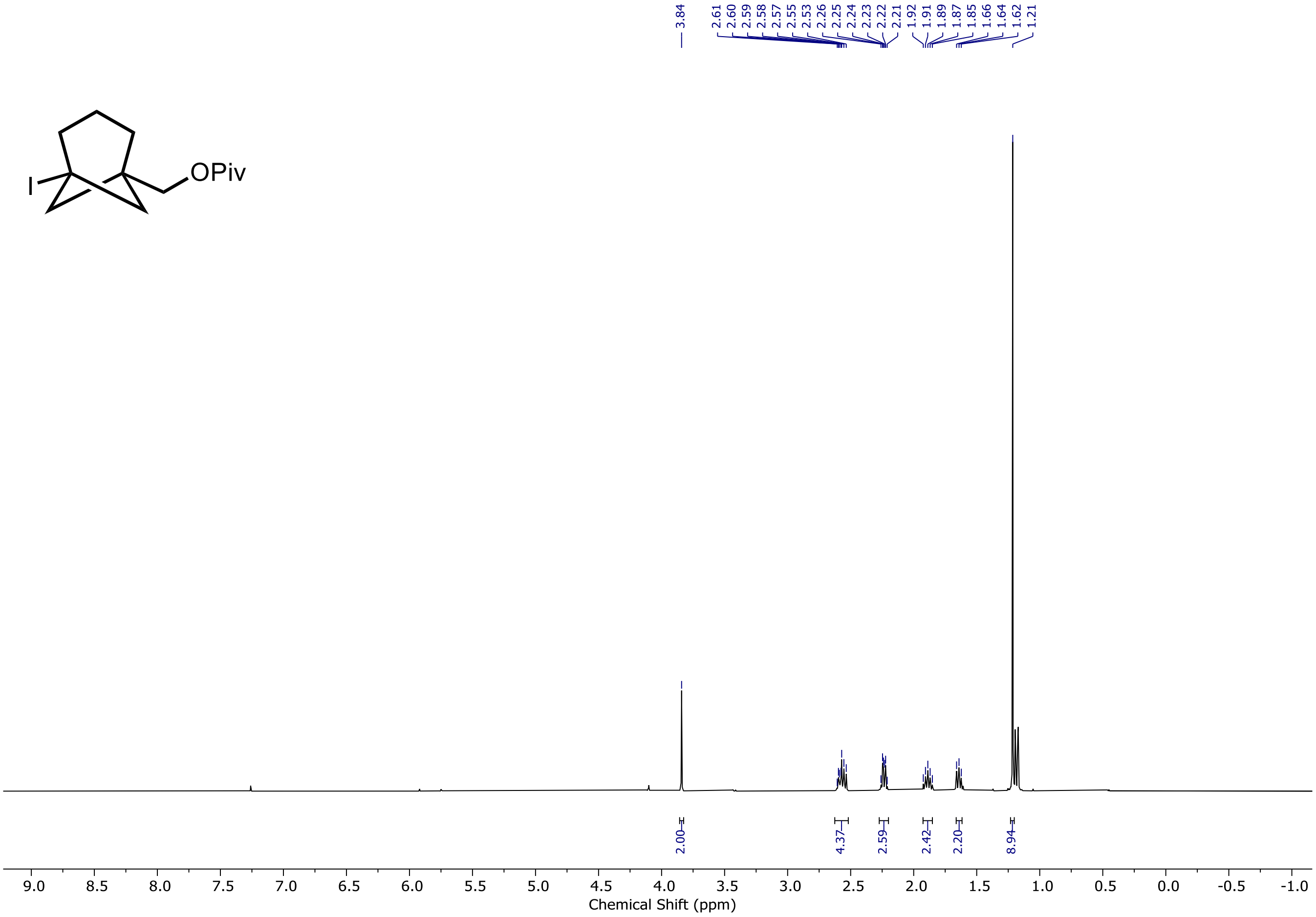

(5-Iodobicyclo[3.1.1]heptan-1-yl)methyl pivalate **(8a)** **^{13}C NMR (101 MHz, CDCl$_3$)**

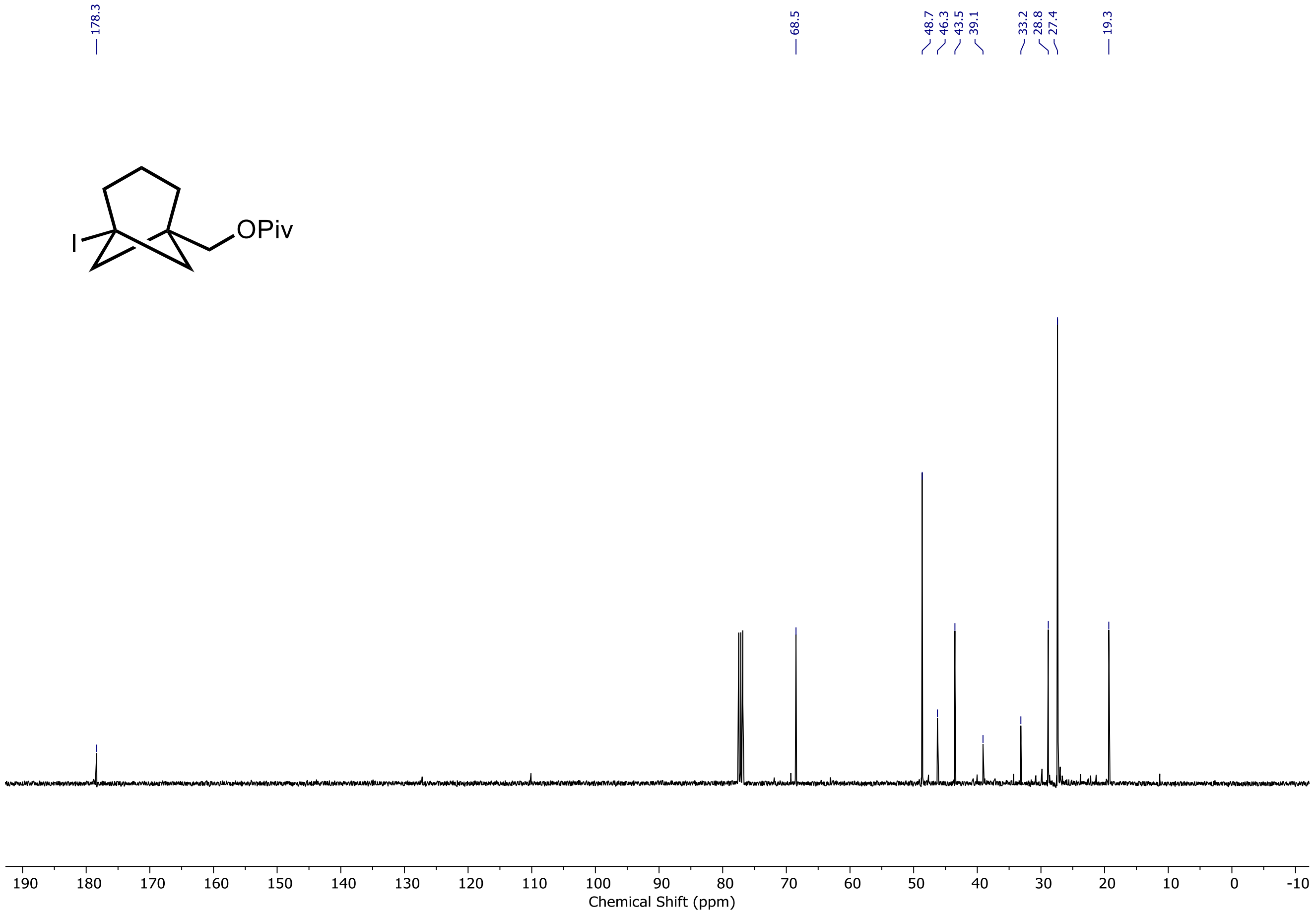

(5-(4-Fluorophenyl)bicyclo[3.1.1]heptan-1-yl)methyl pivalate **(9a)** ^{1}**H NMR (400 MHz, CDCl$_3$)**

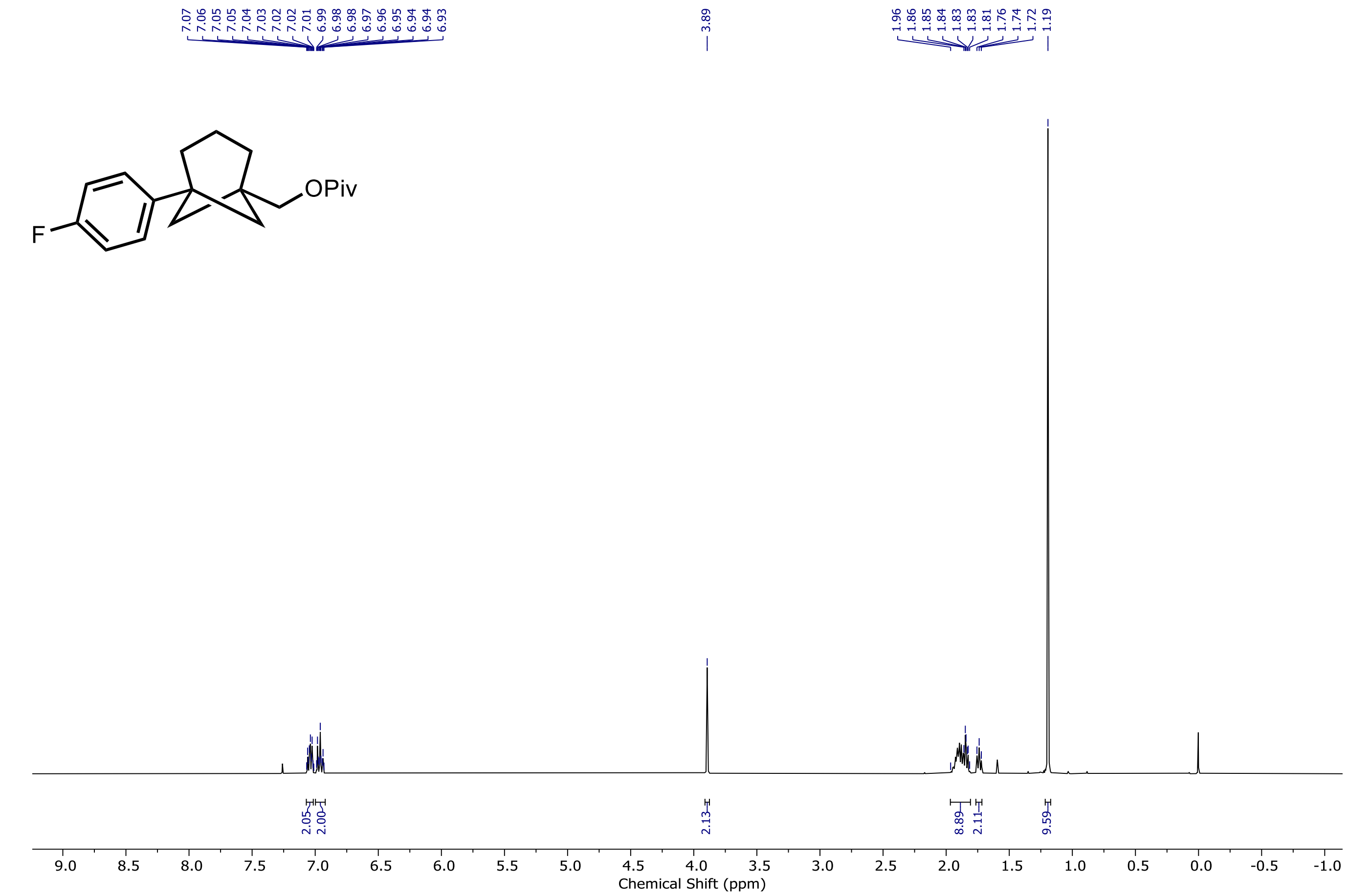

(5-(4-Fluorophenyl)bicyclo[3.1.1]heptan-1-yl)methyl pivalate **(9a)** ^{13}**C NMR (101 MHz, CDCl$_3$)**

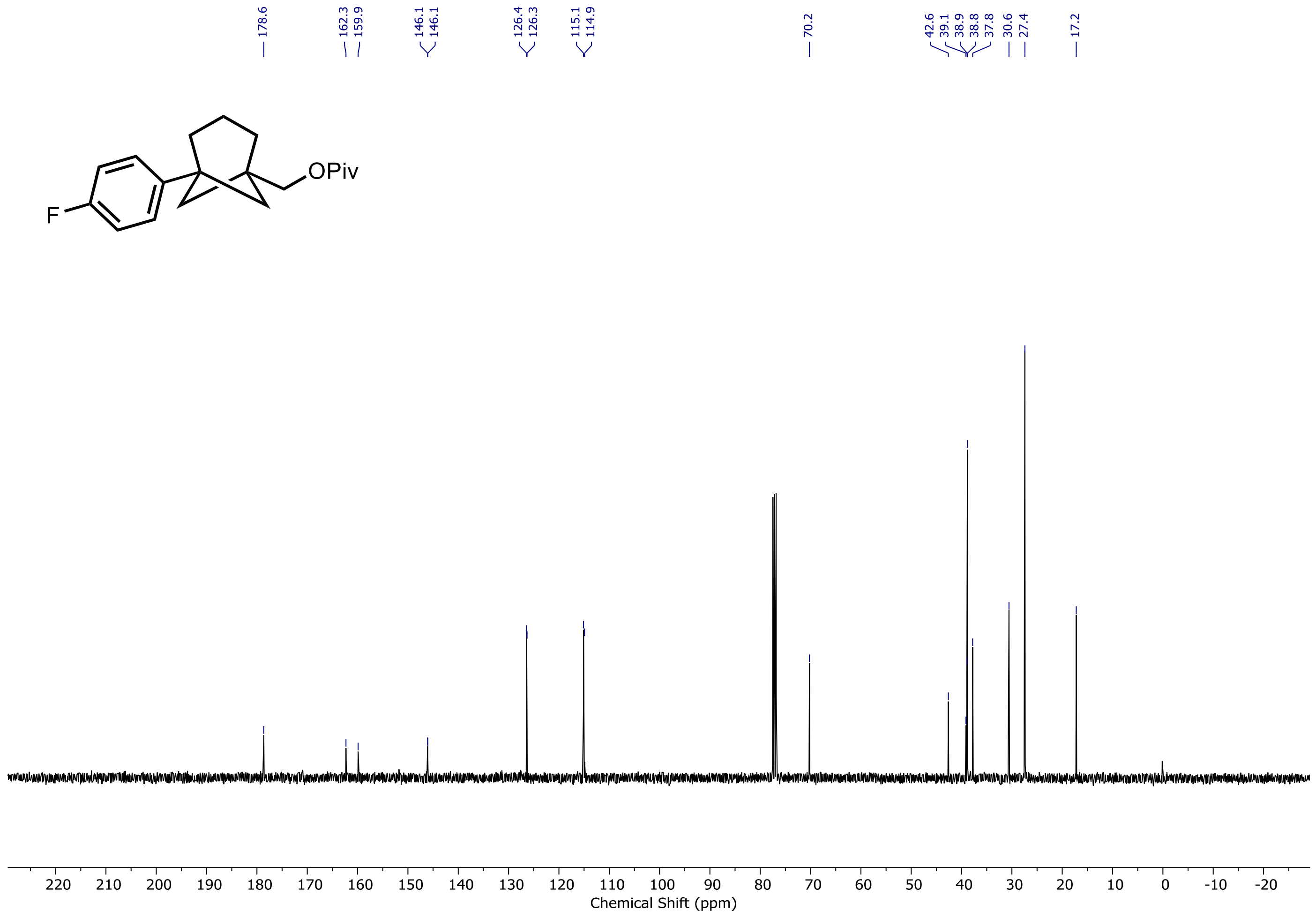

(5-(4-Fluorophenyl)bicyclo[3.1.1]heptan-1-yl)methyl pivalate **(9a)** ^{19}F **NMR (377 MHz, CDCl$_3$)**

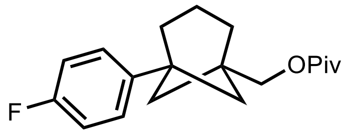

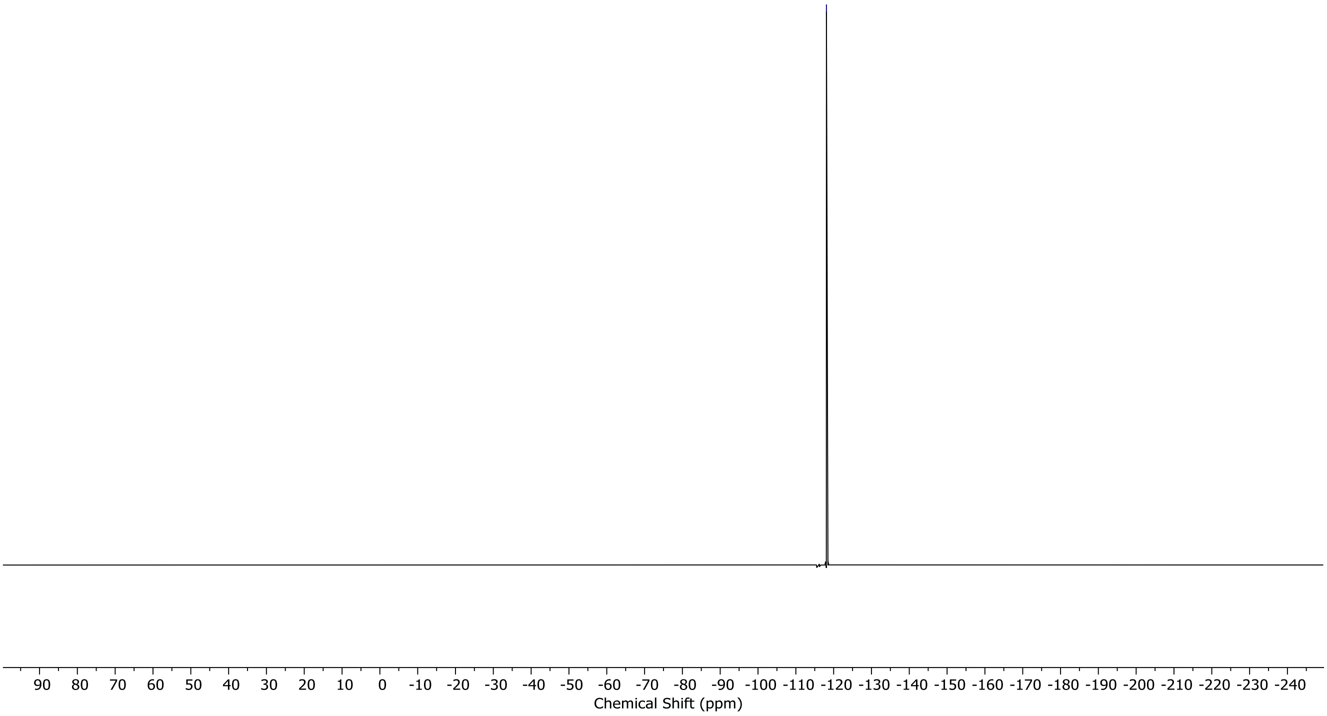

1,3-Dioxoisoindolin-2-yl 5-(4-fluorophenyl)bicyclo[3.1.1]heptane-1-carboxylate (**2b**) **^{1}H NMR (400 MHz, $CDCl_3$)**

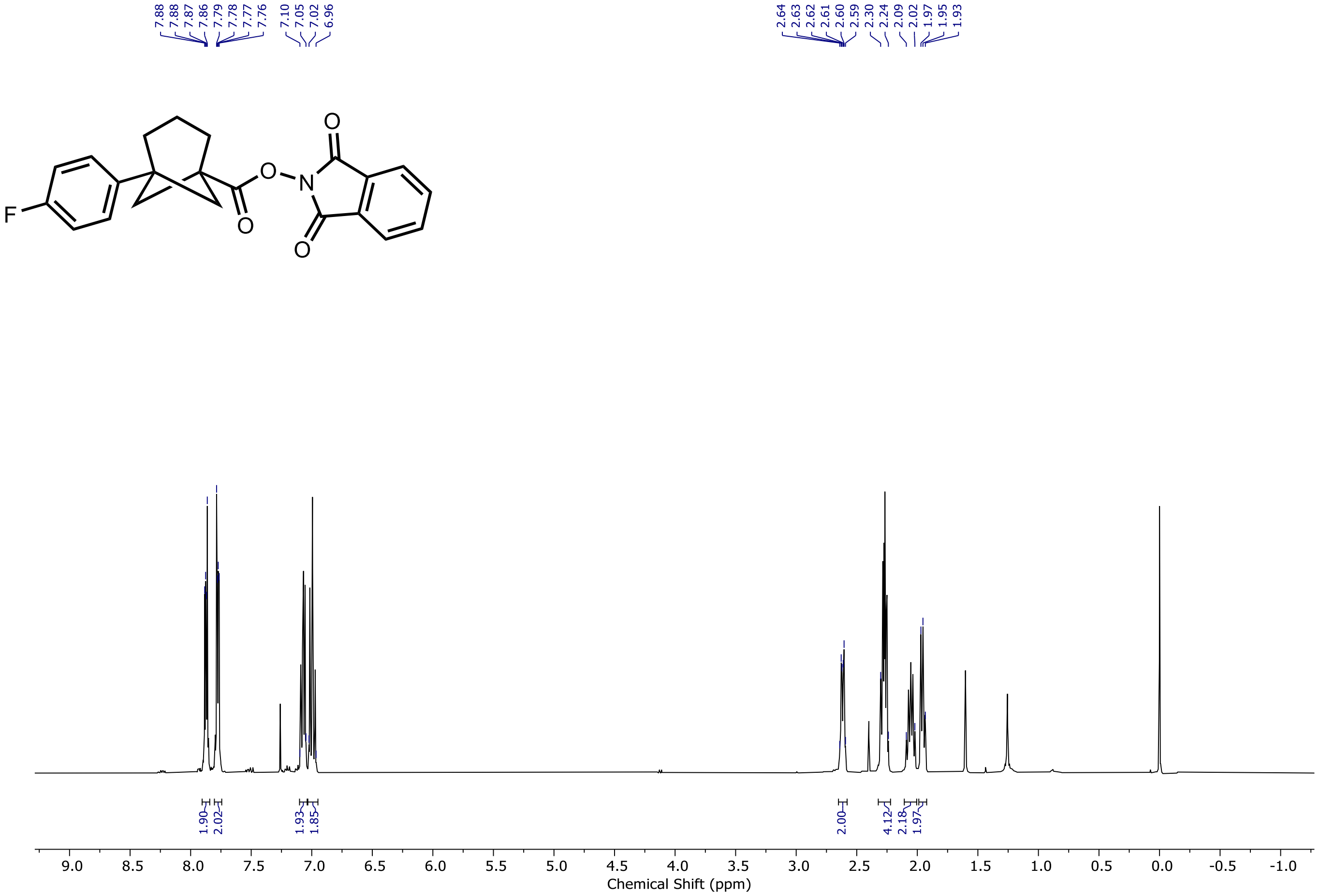

1,3-Dioxoisoindolin-2-yl 5-(4-fluorophenyl)bicyclo[3.1.1]heptane-1-carboxylate (**2b**) **^{13}C NMR (101 MHz, $CDCl_3$)**

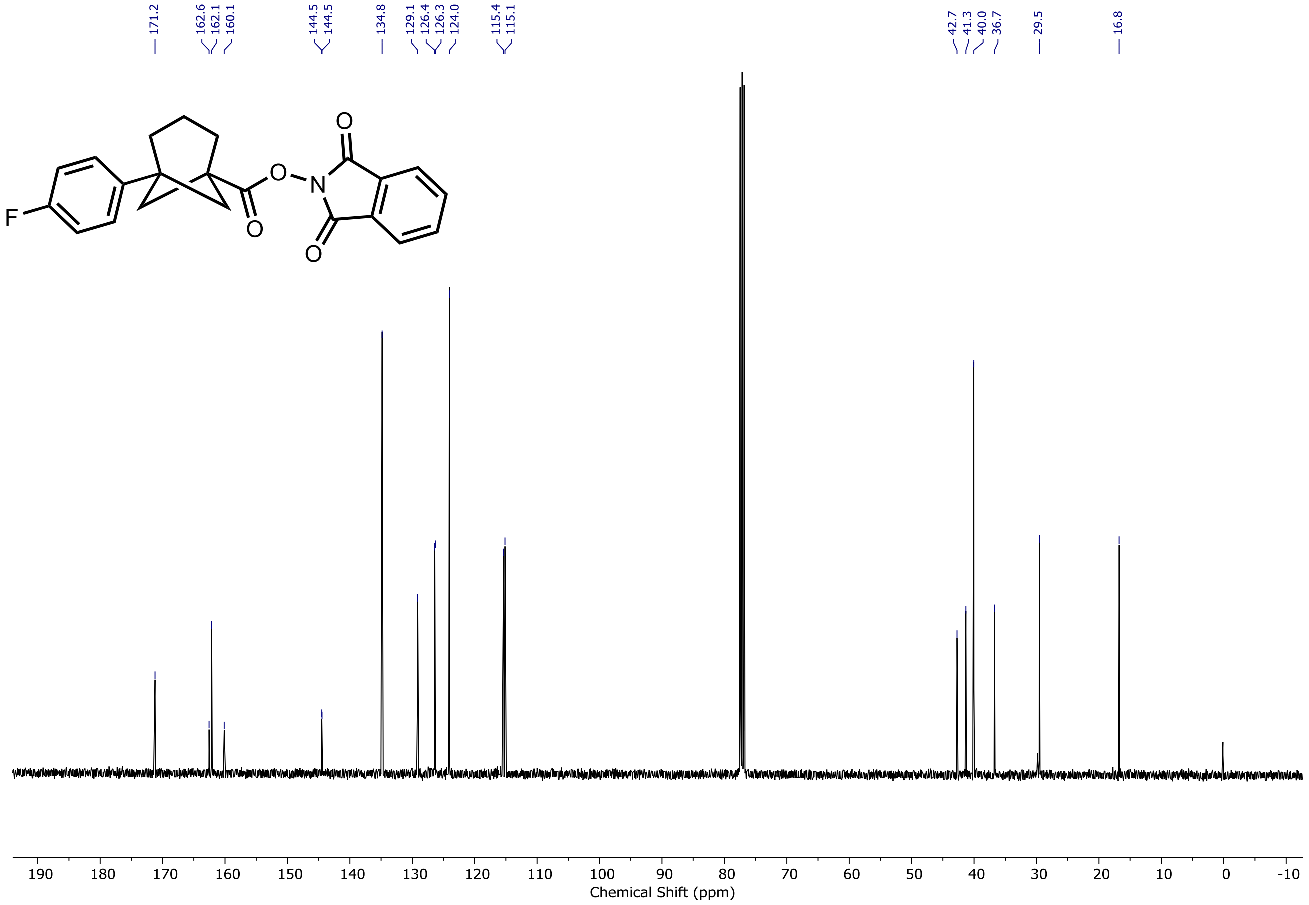

1,3-Dioxoisoindolin-2-yl 5-(4-fluorophenyl)bicyclo[3.1.1]heptane-1-carboxylate (**2b**) **^{19}F NMR (377 MHz, $CDCl_3$)**

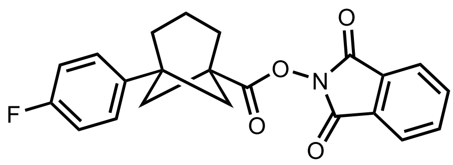

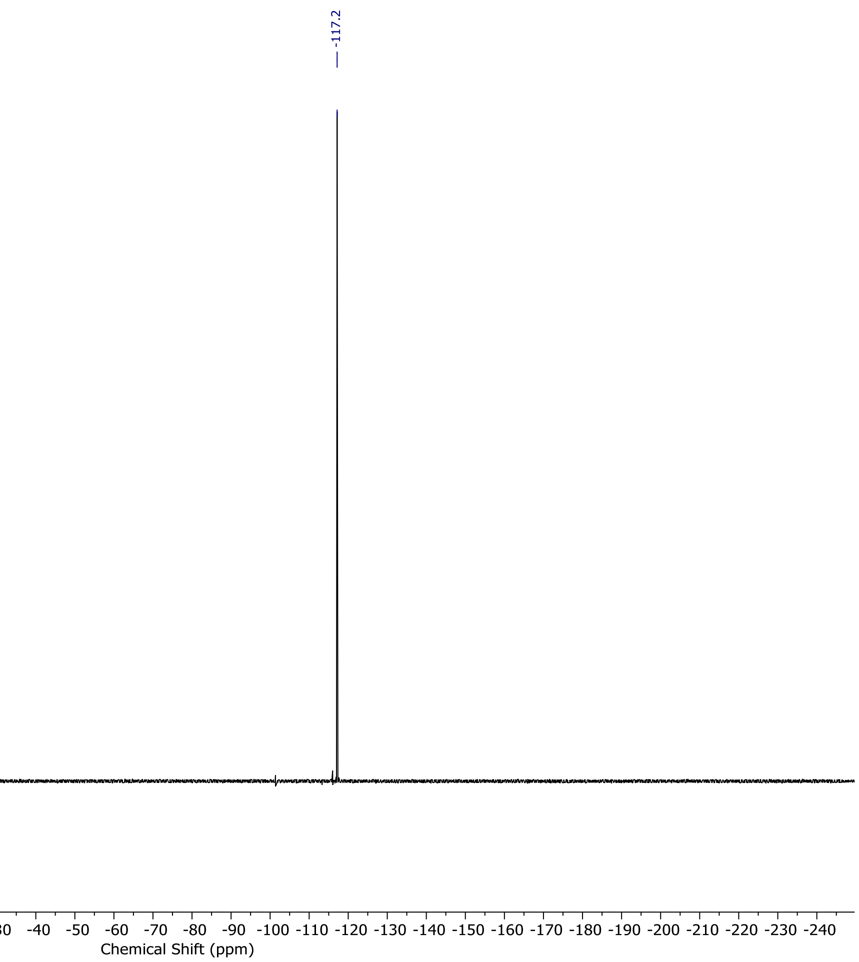
-117.2
90 80 70 60 50 40 30 20 10 0 -10 -20 -30 -40 -50 -60 -70 -80 -90 -100 -110 -120 -130 -140 -150 -160 -170 -180 -190 -200 -210 -220 -230 -240
Chemical Shift (ppm)

(5-(*p*-Tolyl)bicyclo[3.1.1]heptan-1-yl)methyl pivalate **(9b)** ^{1}**H NMR (400 MHz, CDCl$_3$)**

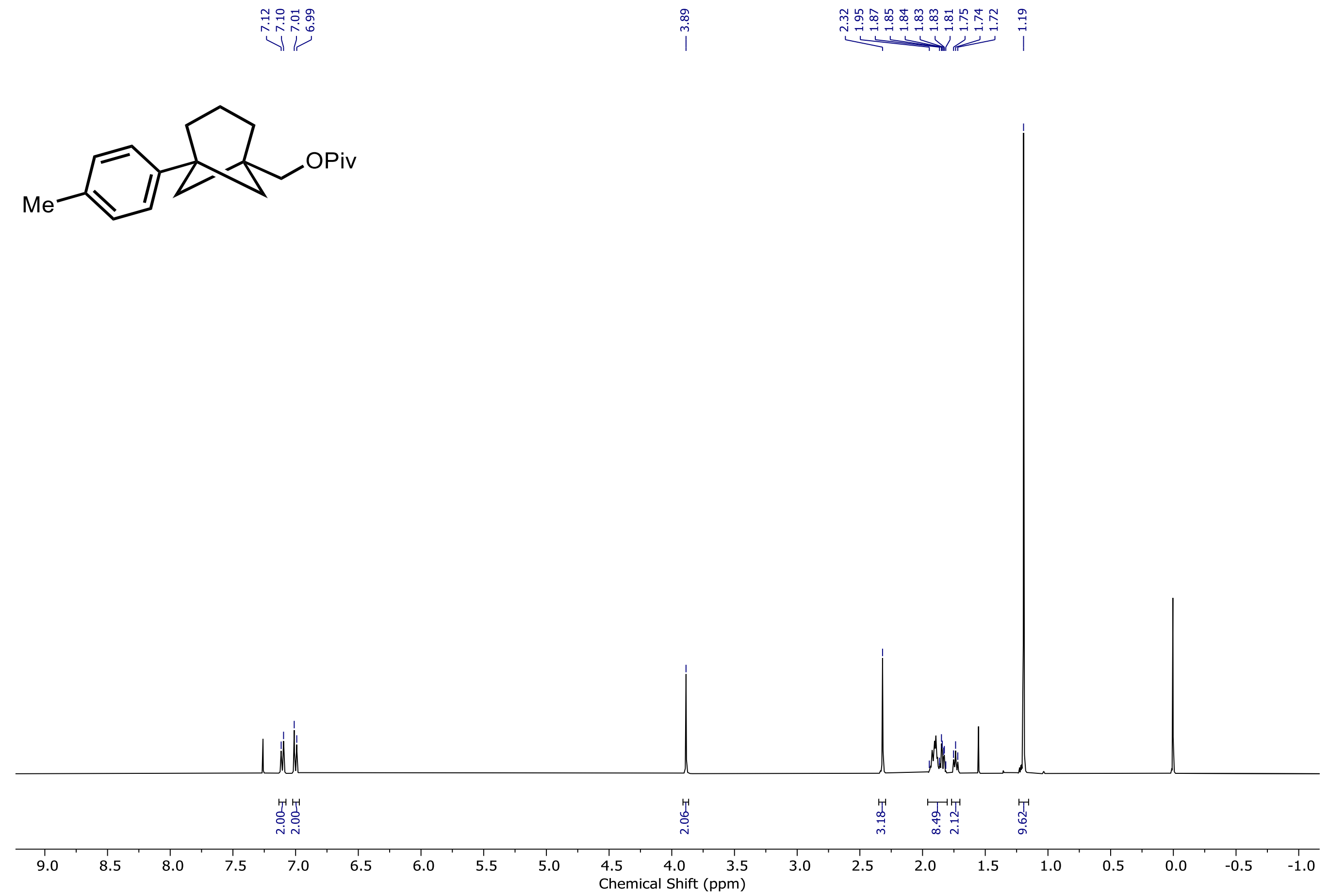

(5-(*p*-Tolyl)bicyclo[3.1.1]heptan-1-yl)methyl pivalate **(9b)** ^{13}**C NMR (101 MHz, CDCl$_3$)**

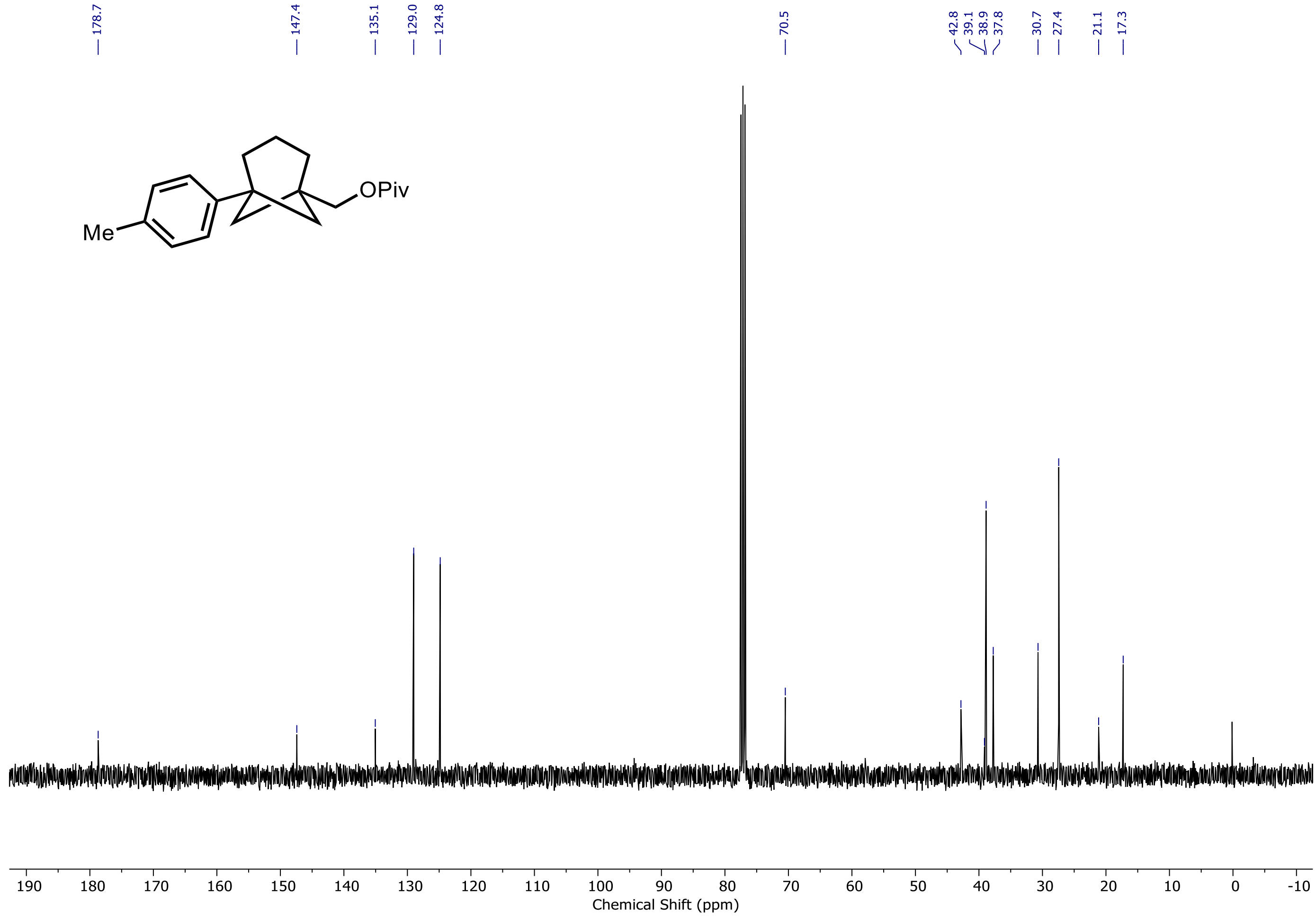

1,3-Dioxoisoindolin-2-yl 5-(p-tolyl)bicyclo[3.1.1]heptane-1-carboxylate (**2c**) **^{1}H NMR (400 MHz, $CDCl_3$)**

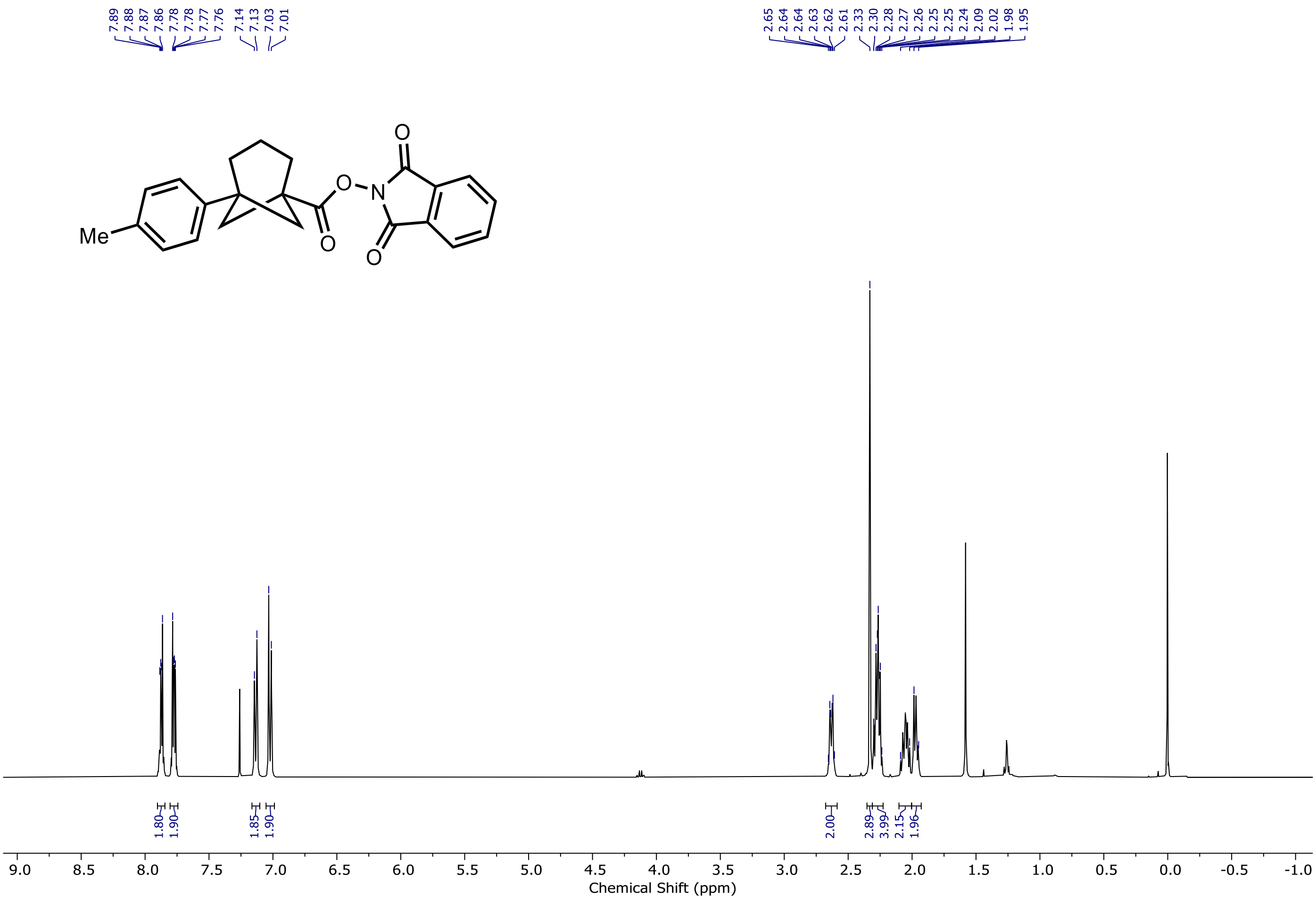

1,3-Dioxoisoindolin-2-yl 5-(p-tolyl)bicyclo[3.1.1]heptane-1-carboxylate (**2c**) **^{13}C NMR (101 MHz, $CDCl_3$)**

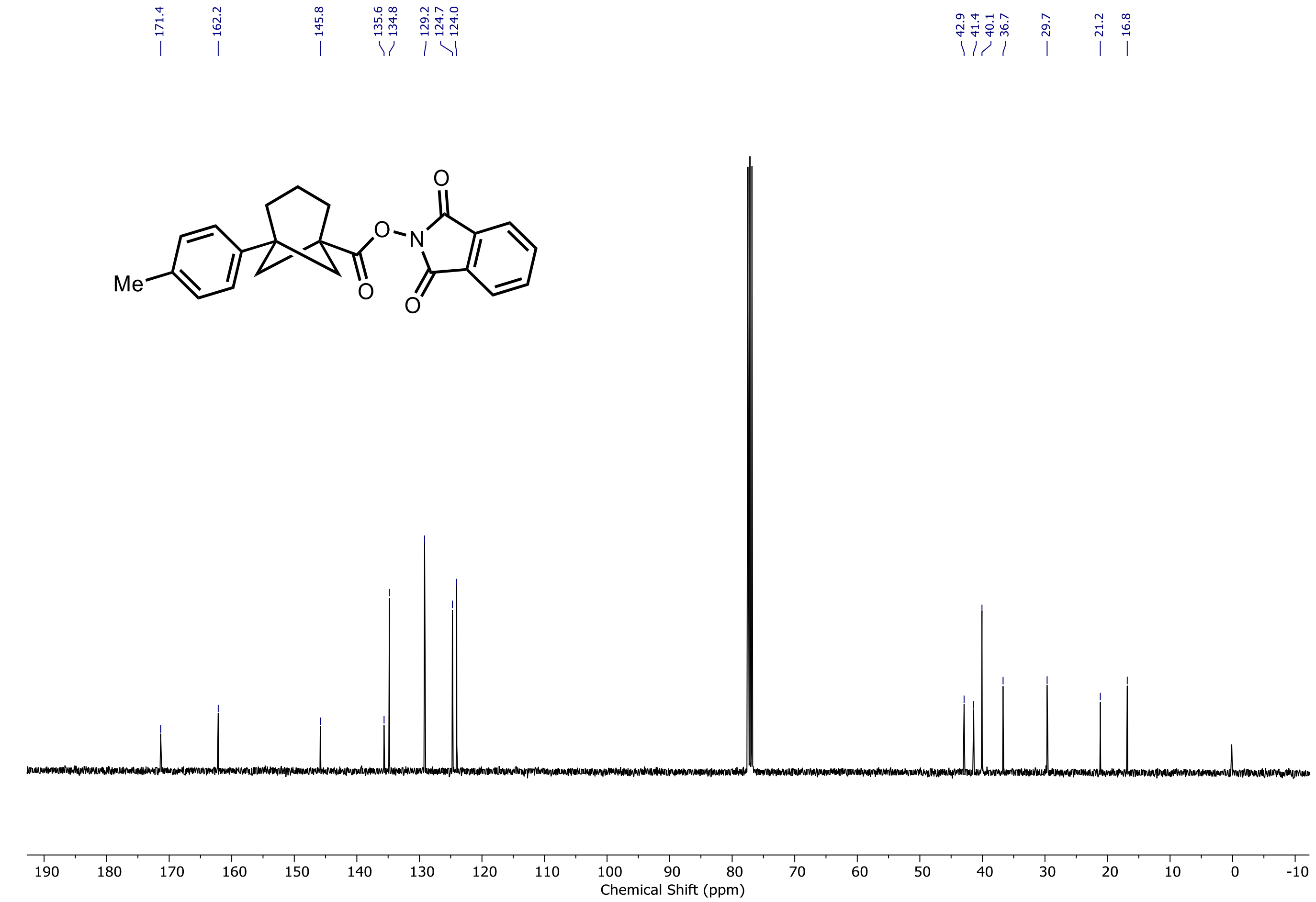

1-Iodo-5-(4-(trifluoromethyl)benzyl)bicyclo[3.1.1]heptane **(8b)** **^{1}H NMR (400 MHz, CDCl$_3$)**

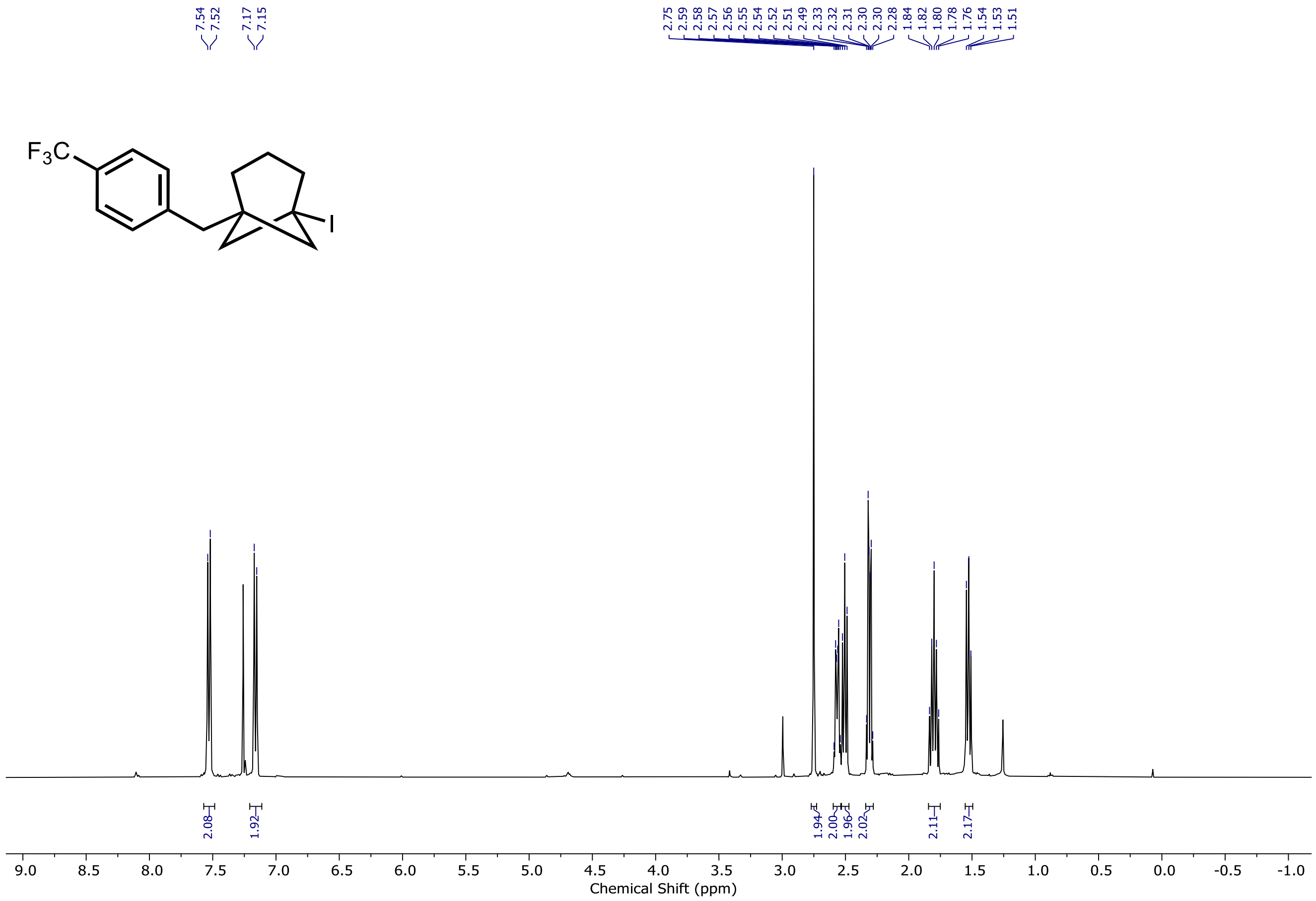

1-Iodo-5-(4-(trifluoromethyl)benzyl)bicyclo[3.1.1]heptane **(8b)** **^{13}C NMR (101 MHz, CDCl$_3$)**

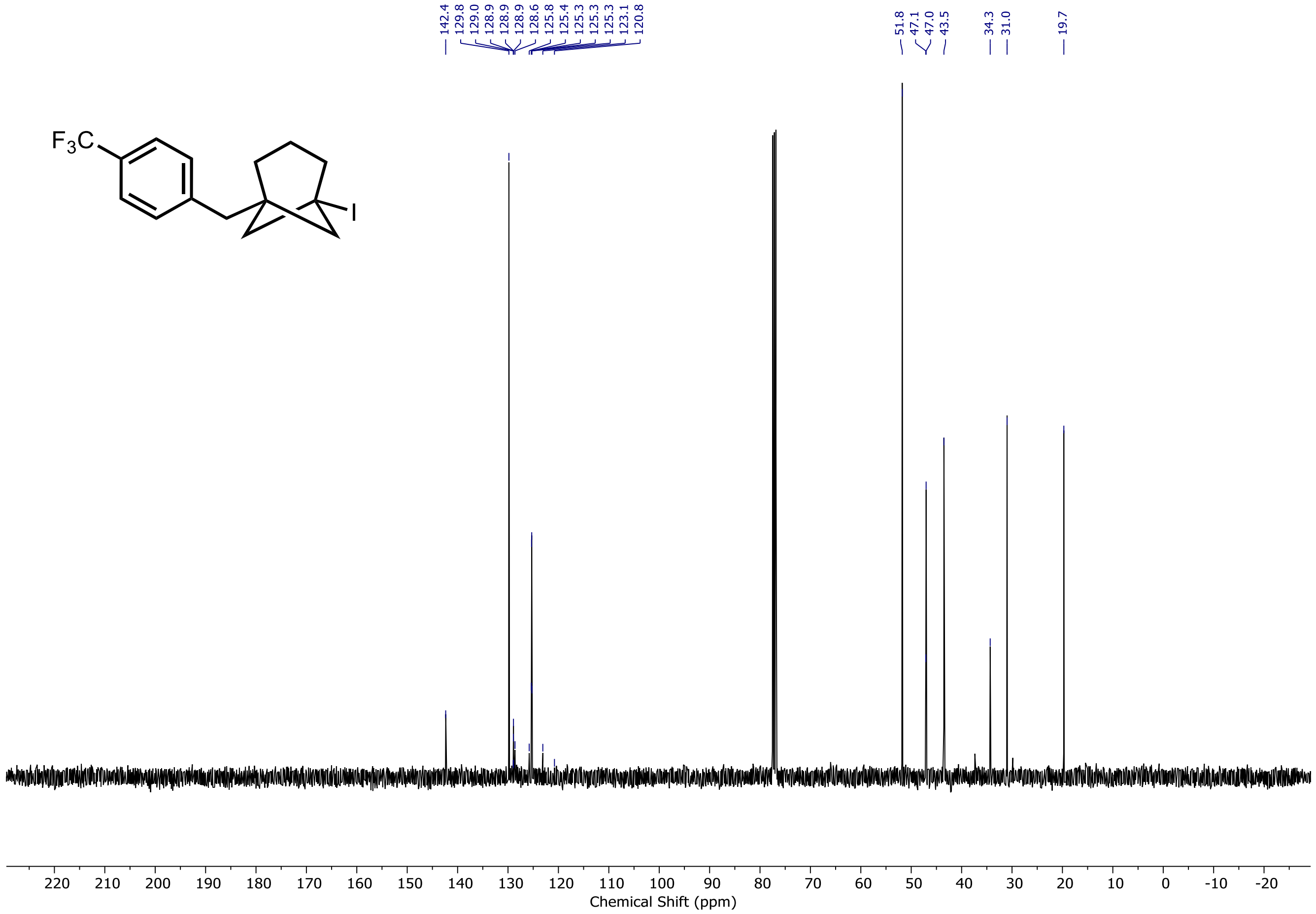

1-Iodo-5-(4-(trifluoromethyl)benzyl)bicyclo[3.1.1]heptane **(8b)** ^{19}F **NMR (377 MHz, $CDCl_3$)**

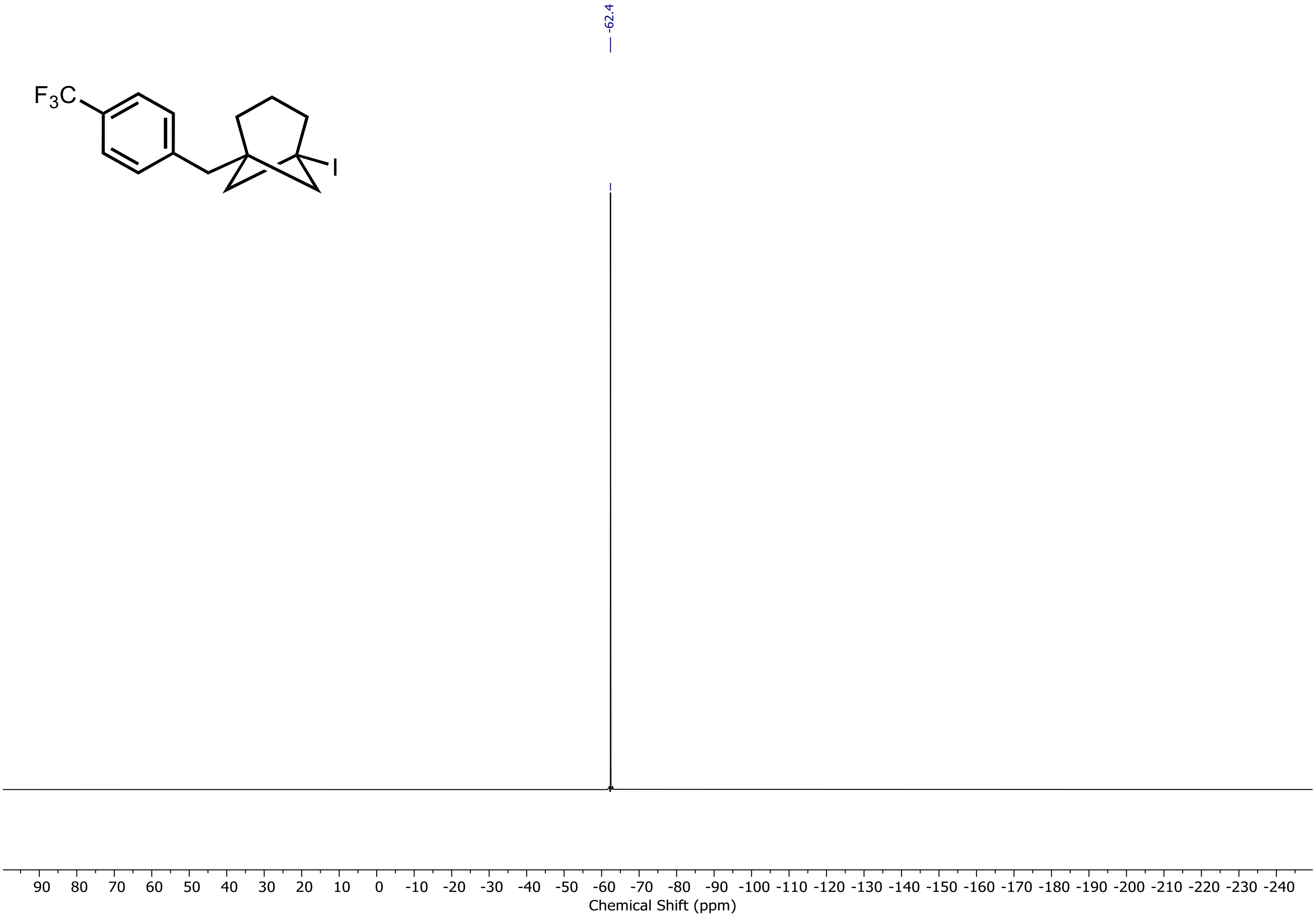

1,3-Dioxoisoindolin-2-yl 5-(4-(trifluoromethyl)benzyl)bicyclo[3.1.1]heptane-1-carboxylate **(2d)** **^{1}H NMR (500 MHz, CDCl$_3$)**

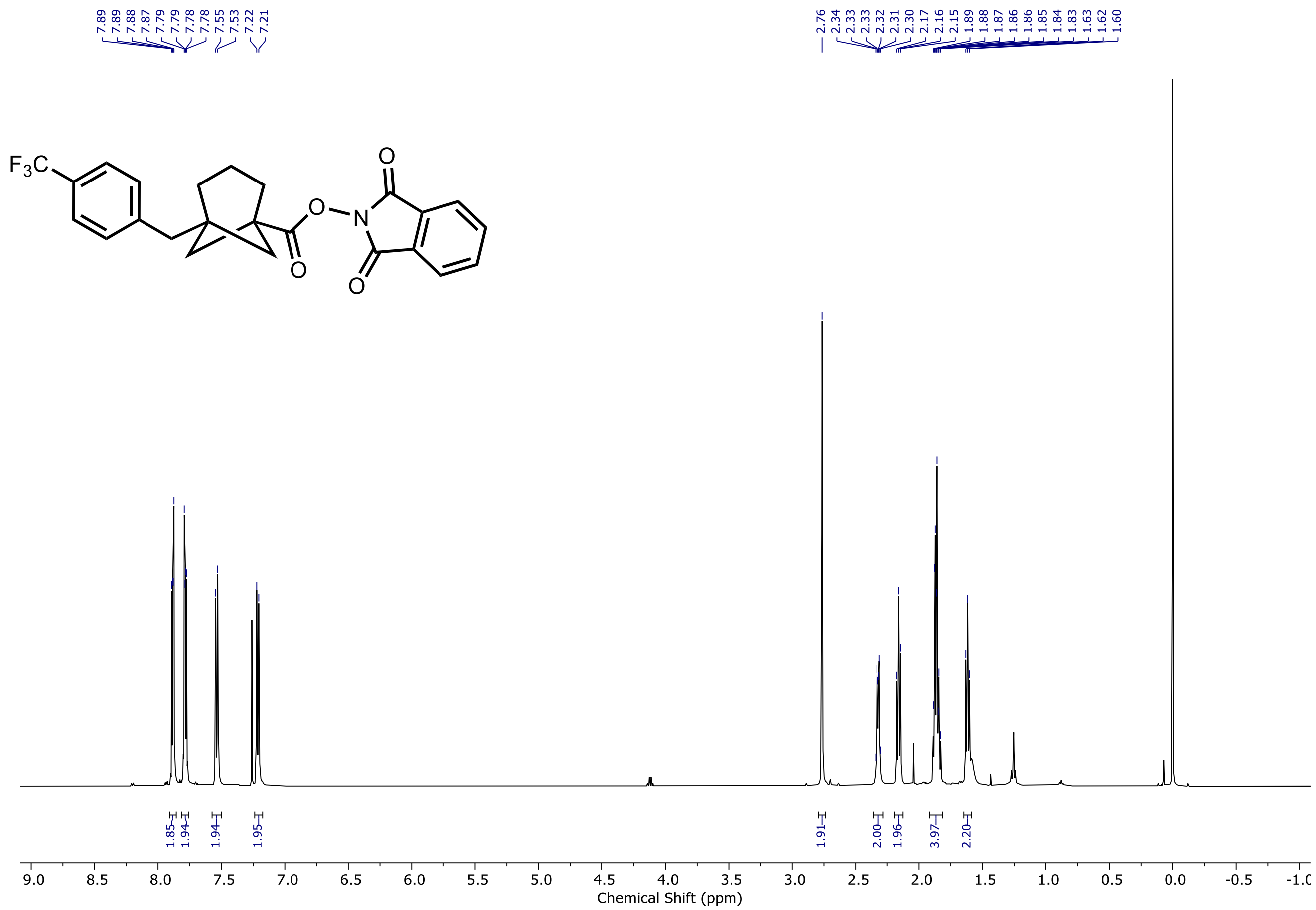

1,3-Dioxoisoindolin-2-yl 5-(4-(trifluoromethyl)benzyl)bicyclo[3.1.1]heptane-1-carboxylate **(2d)** **^{13}C NMR (125 MHz, CDCl$_3$)**

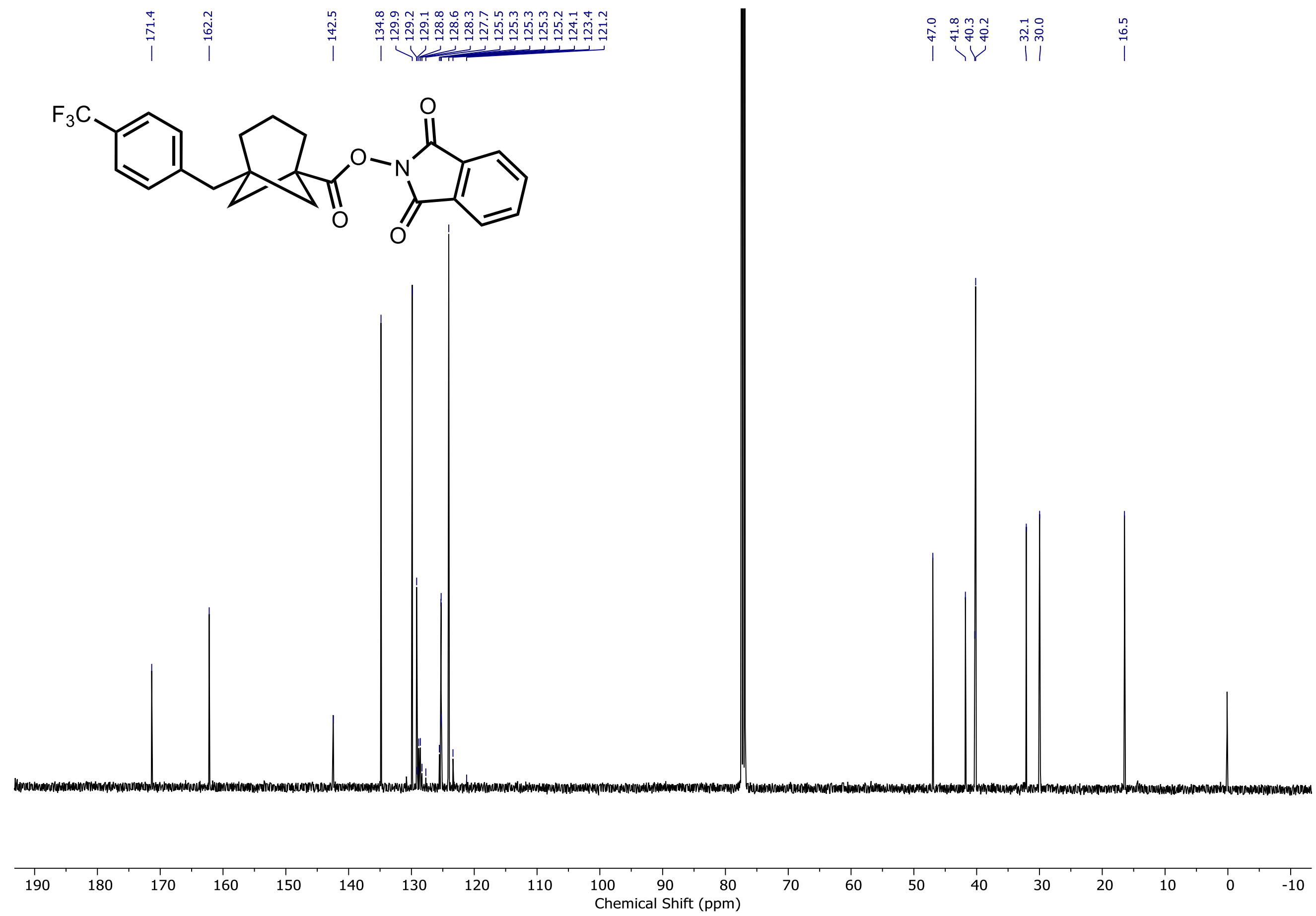

1,3-Dioxoisoindolin-2-yl 5-(4-(trifluoromethyl)benzyl)bicyclo[3.1.1]heptane-1-carboxylate **(2d)** **^{19}F NMR (470 MHz, CDCl$_3$)**

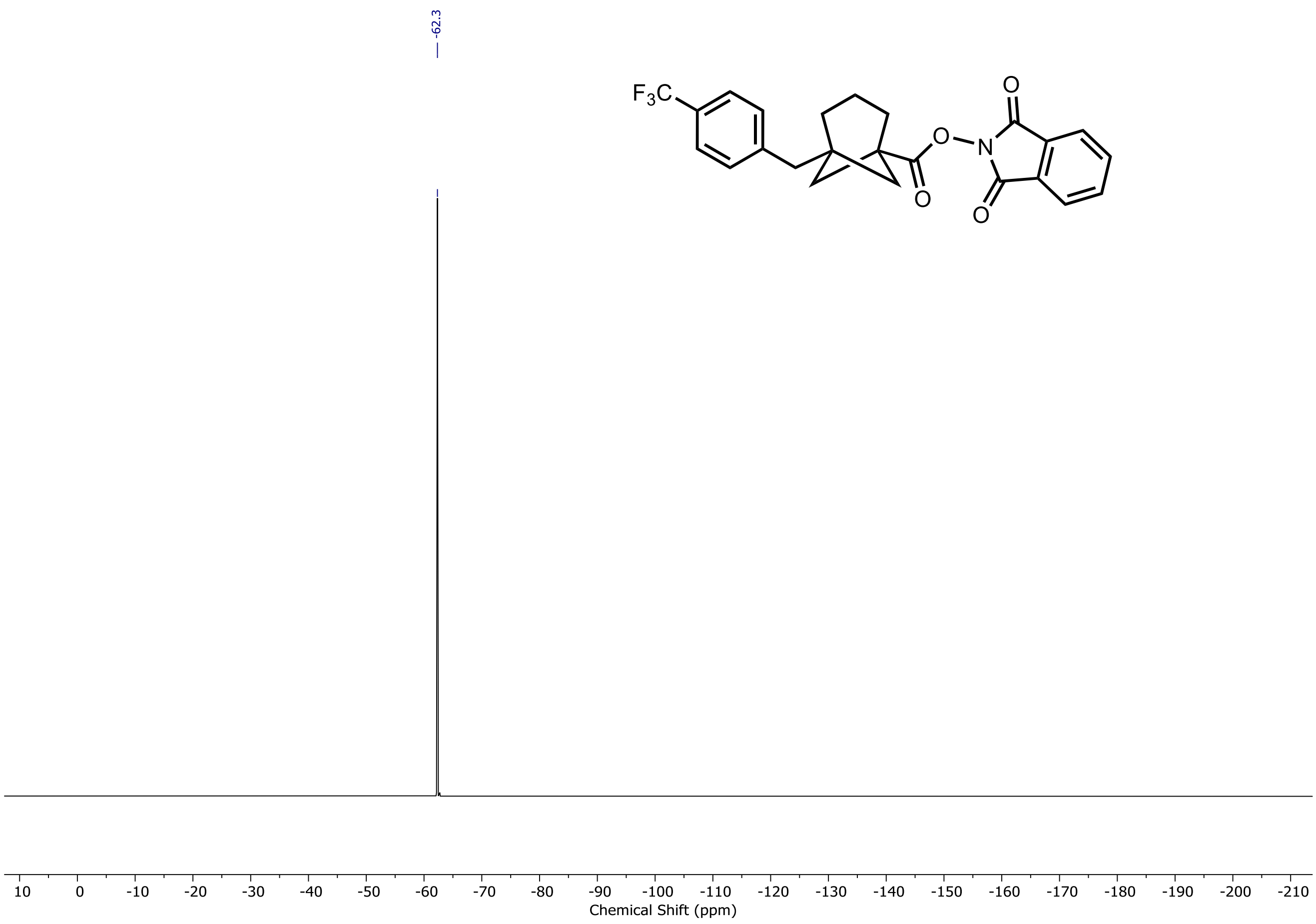

1-(3,5-Bis(trifluoromethyl)benzyl)-5-iodobicyclo[3.1.1]heptane **(8c)** **^{1}H NMR (400 MHz, $CDCl_3$)**

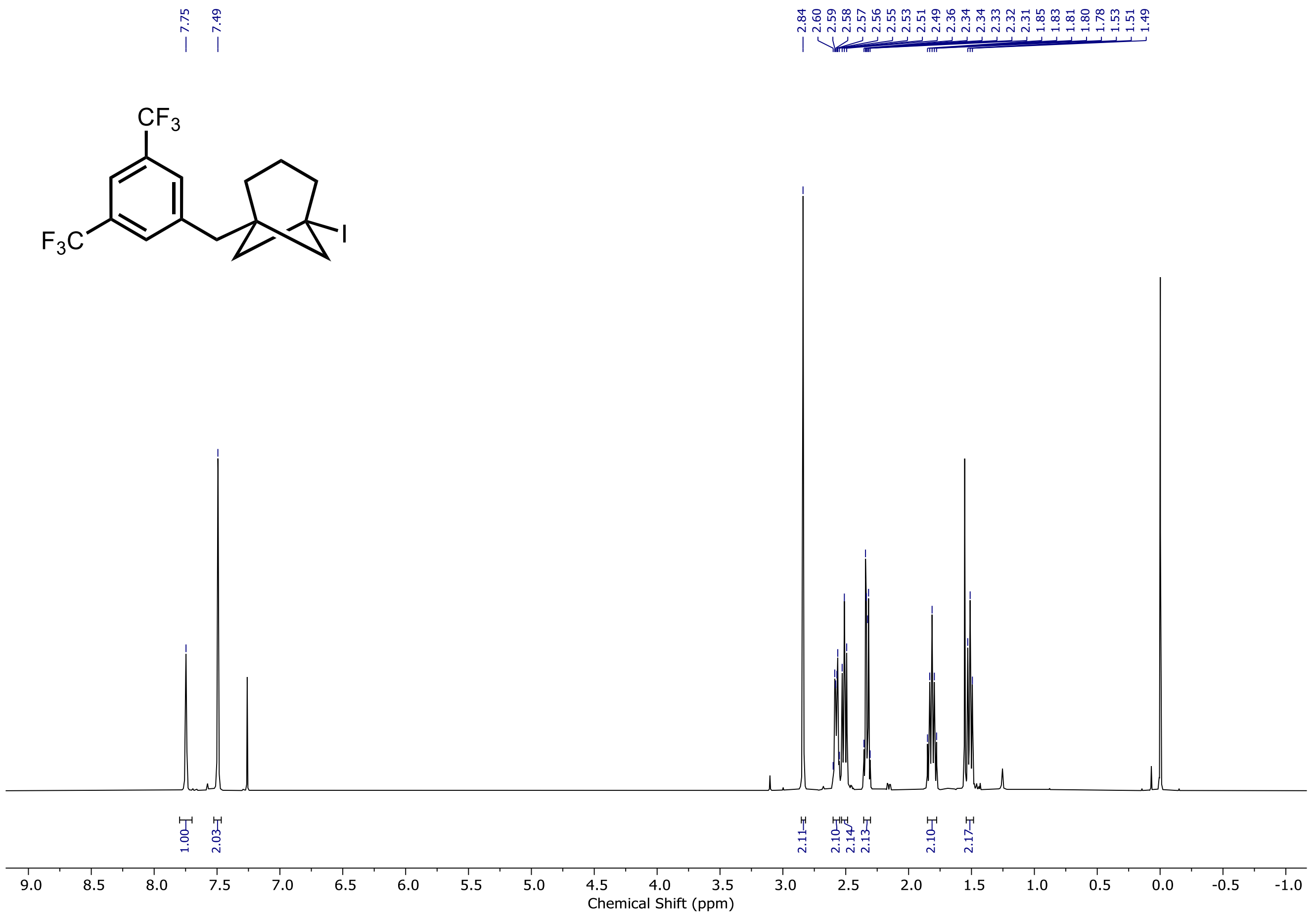

1-(3,5-Bis(trifluoromethyl)benzyl)-5-iodobicyclo[3.1.1]heptane **(8c)** **^{13}C NMR (101 MHz, $CDCl_3$)**

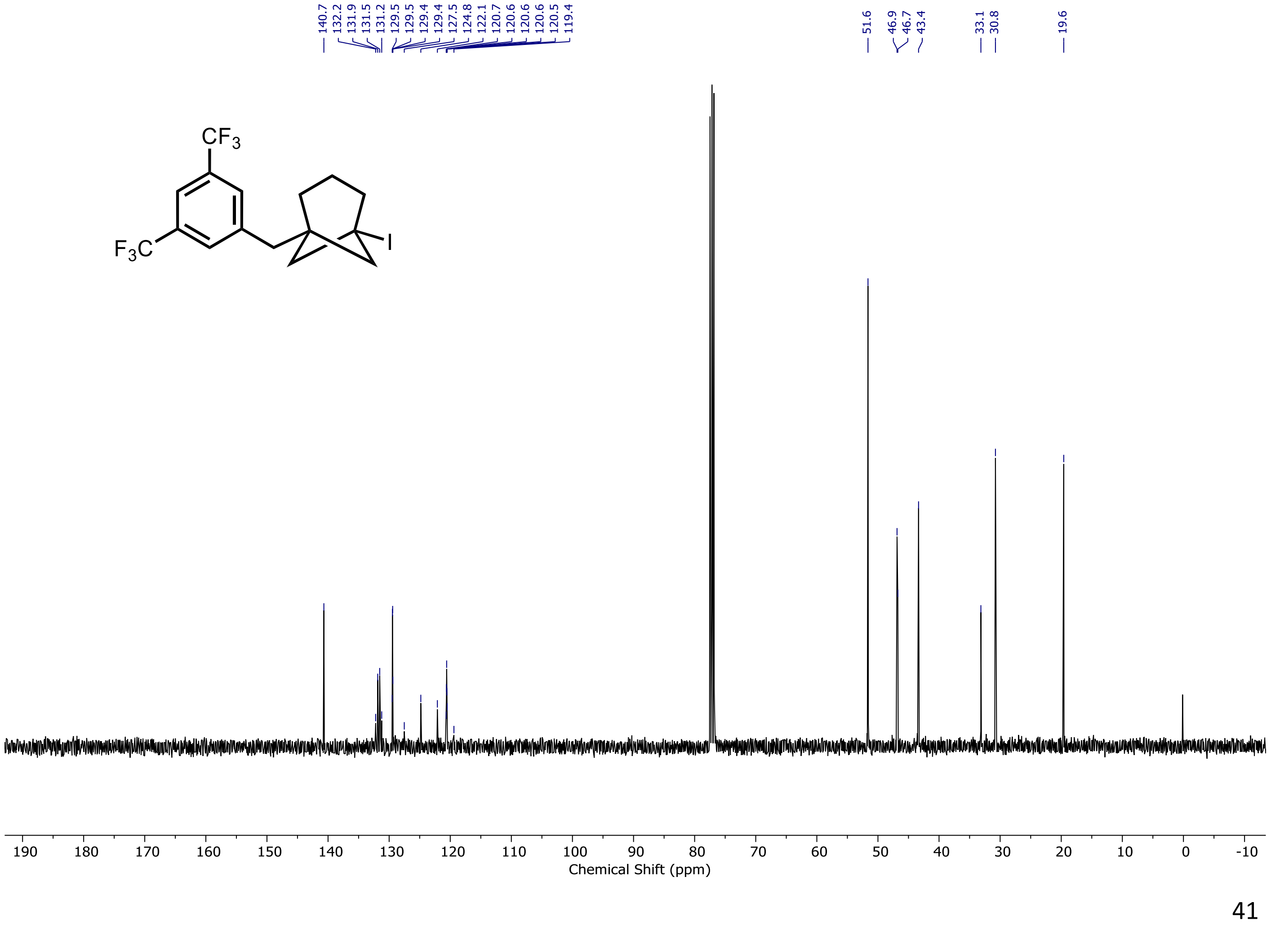

1-(3,5-Bis(trifluoromethyl)benzyl)-5-iodobicyclo[3.1.1]heptane **(8c)** ^{19}F **NMR (377 MHz, $CDCl_3$)**

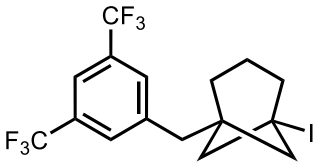

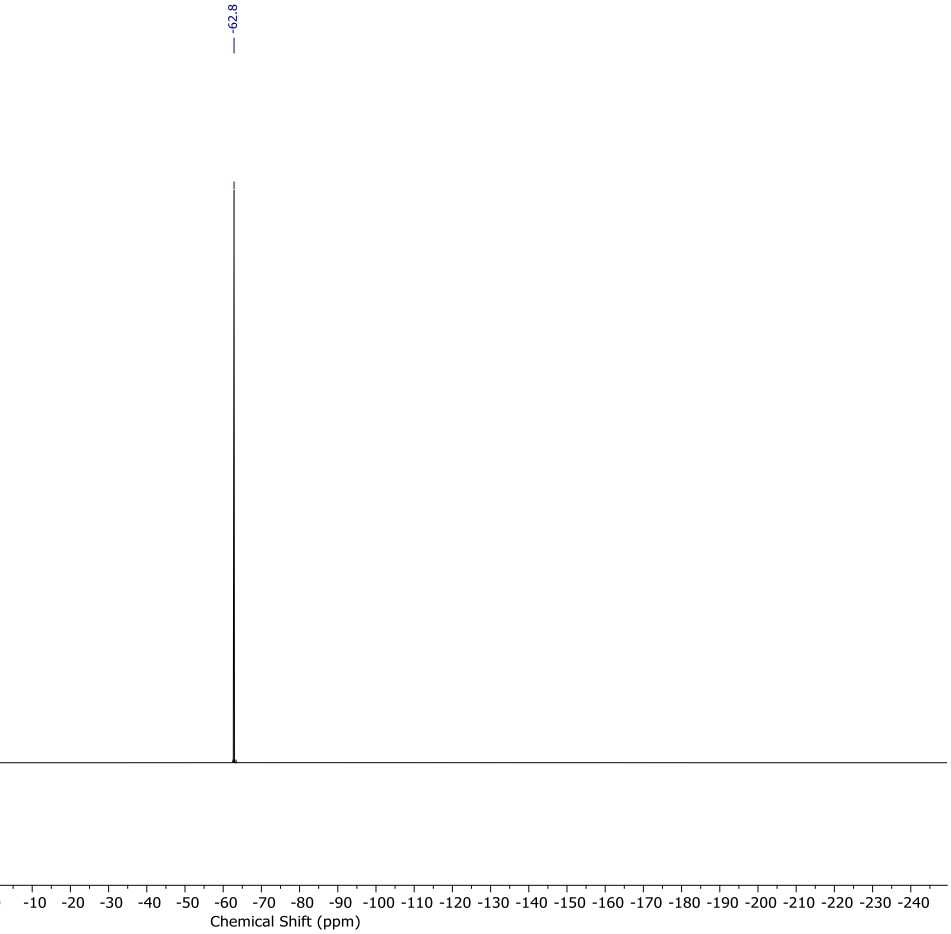

1,3-Dioxoisoindolin-2-yl 5-(3,5-bis(trifluoromethyl)benzyl)bicyclo[3.1.1]heptane-1-carboxylate (**2e**) **^{1}H NMR (500 MHz, $CDCl_3$)**

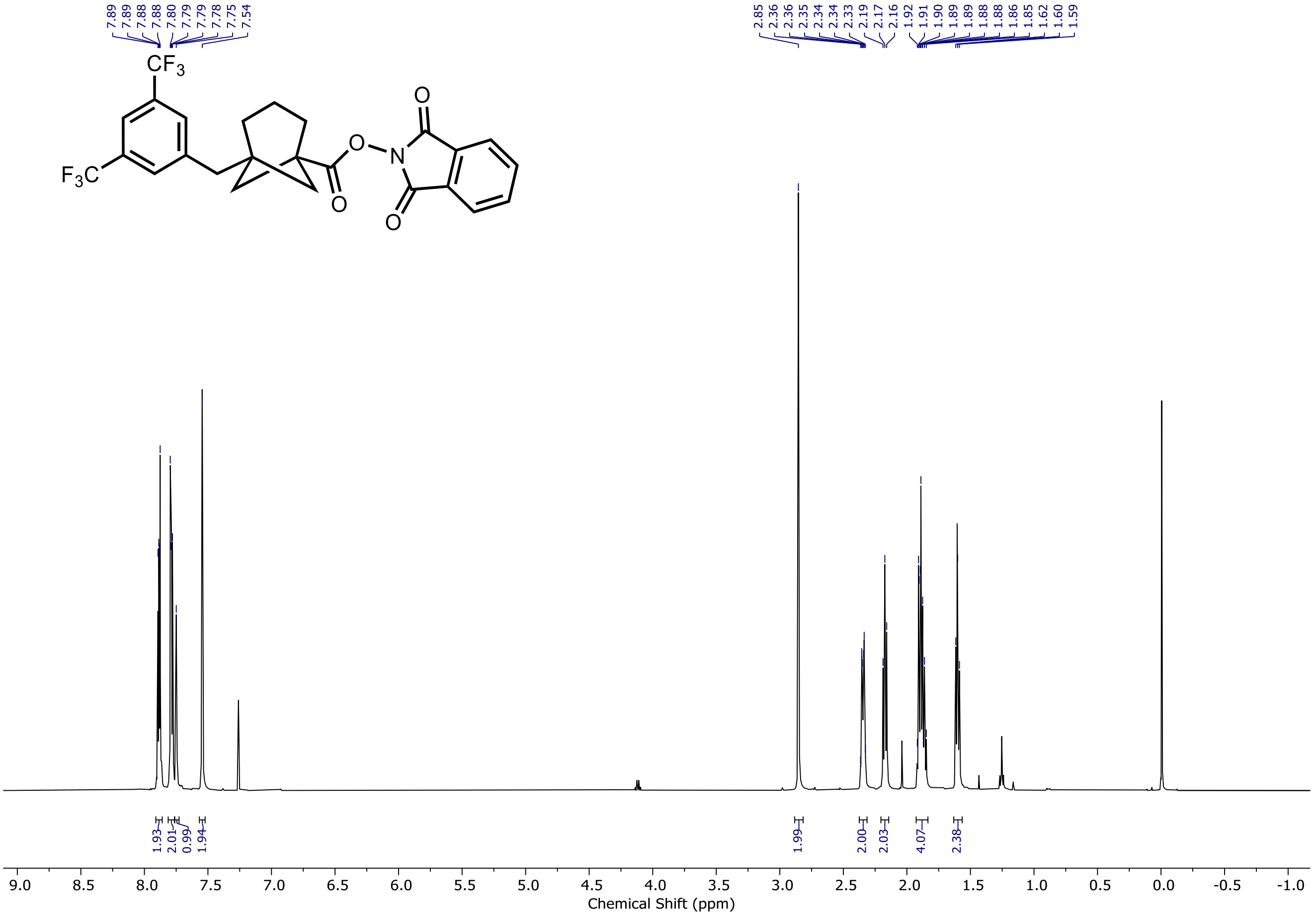

1,3-Dioxoisoindolin-2-yl 5-(3,5-bis(trifluoromethyl)benzyl)bicyclo[3.1.1]heptane-1-carboxylate (**2e**) **^{13}C NMR (125 MHz, $CDCl_3$)**

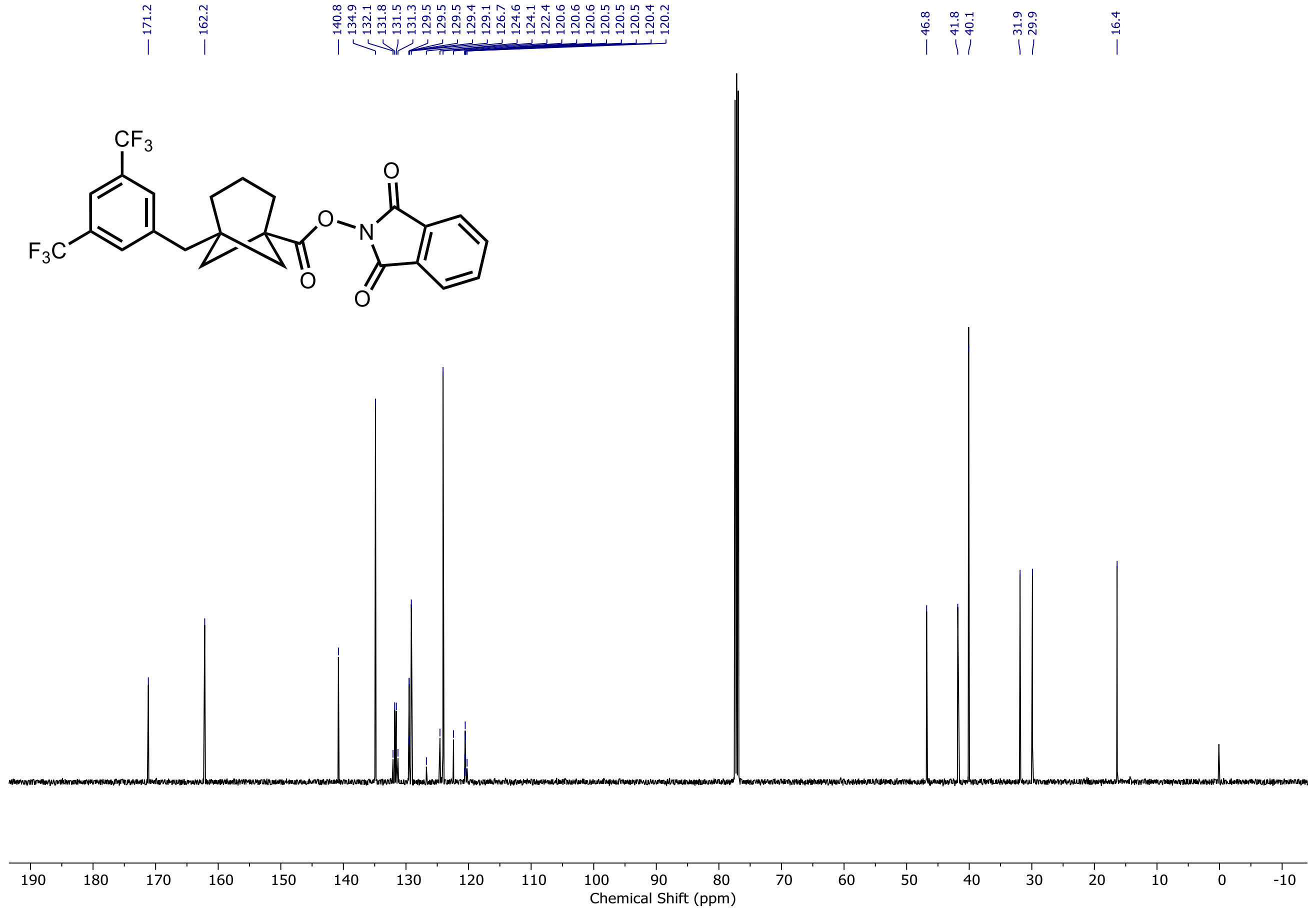

1,3-Dioxoisoindolin-2-yl 5-(3,5-bis(trifluoromethyl)benzyl)bicyclo[3.1.1]heptane-1-carboxylate (**2e**) **^{19}F NMR (470 MHz, $CDCl_3$)**

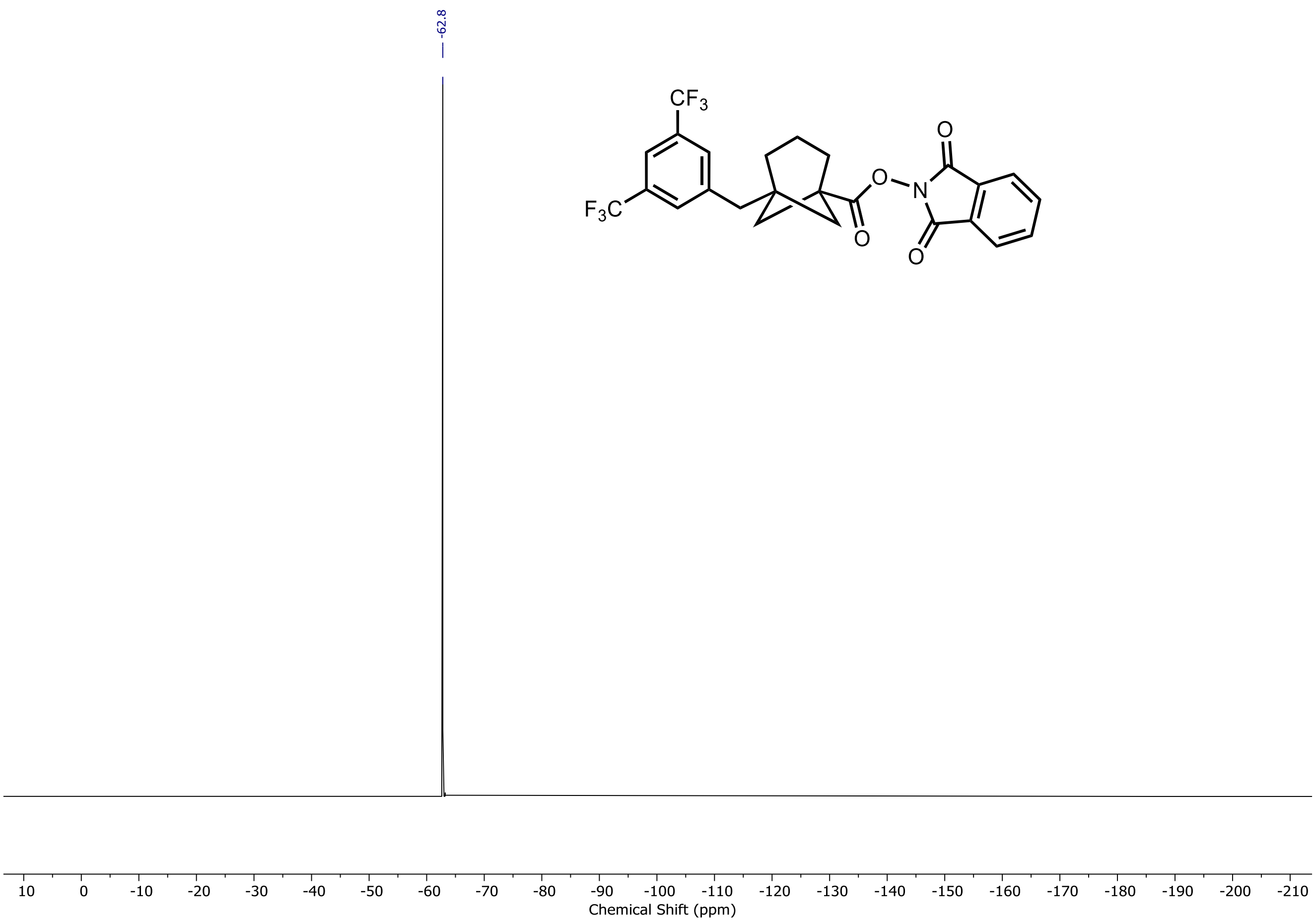

1-Iodo-5-(trifluoromethyl)bicyclo[3.1.1]heptane **(8d)** **^{1}H NMR (400 MHz, CDCl$_3$)**

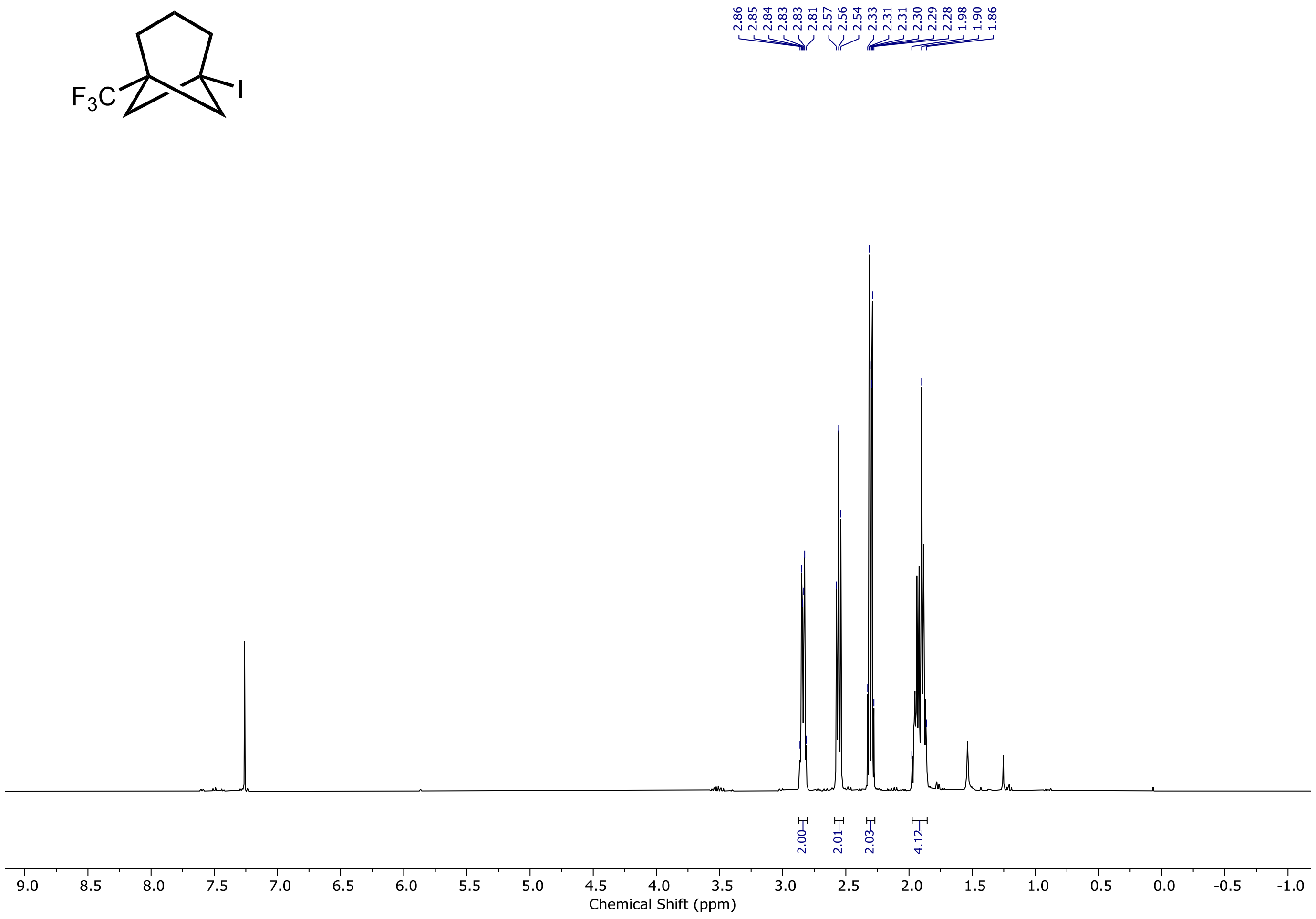

1-Iodo-5-(trifluoromethyl)bicyclo[3.1.1]heptane **(8d)** **^{13}C NMR (101 MHz, CDCl$_3$)**

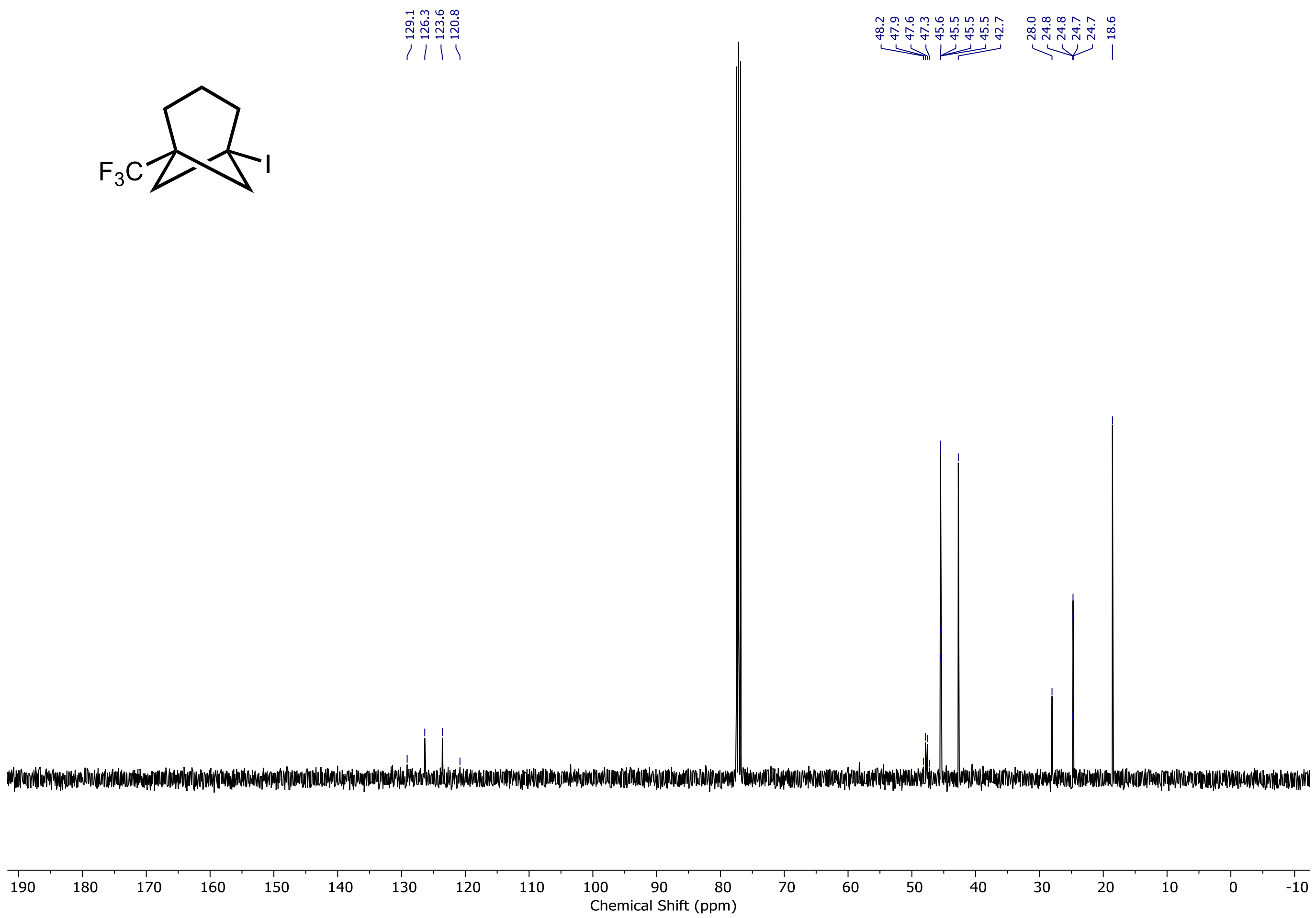

1-Iodo-5-(trifluoromethyl)bicyclo[3.1.1]heptane **(8d)** ^{19}F **NMR (377 MHz, $CDCl_3$)**

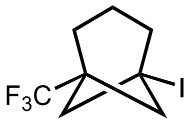

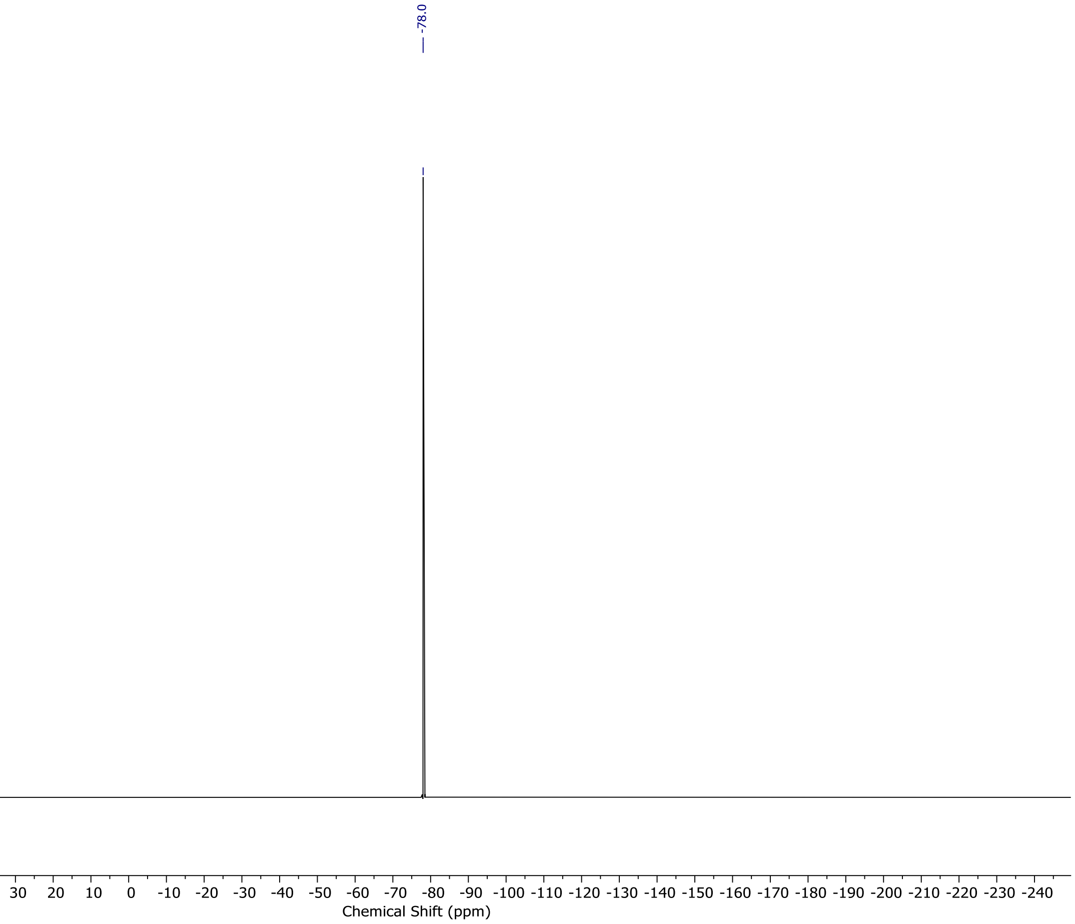

1,3-Dioxoisoindolin-2-yl 5-(trifluoromethyl)bicyclo[3.1.1]heptane-1-carboxylate **(2f)** ^{1}**H NMR (400 MHz, $CDCl_3$)**

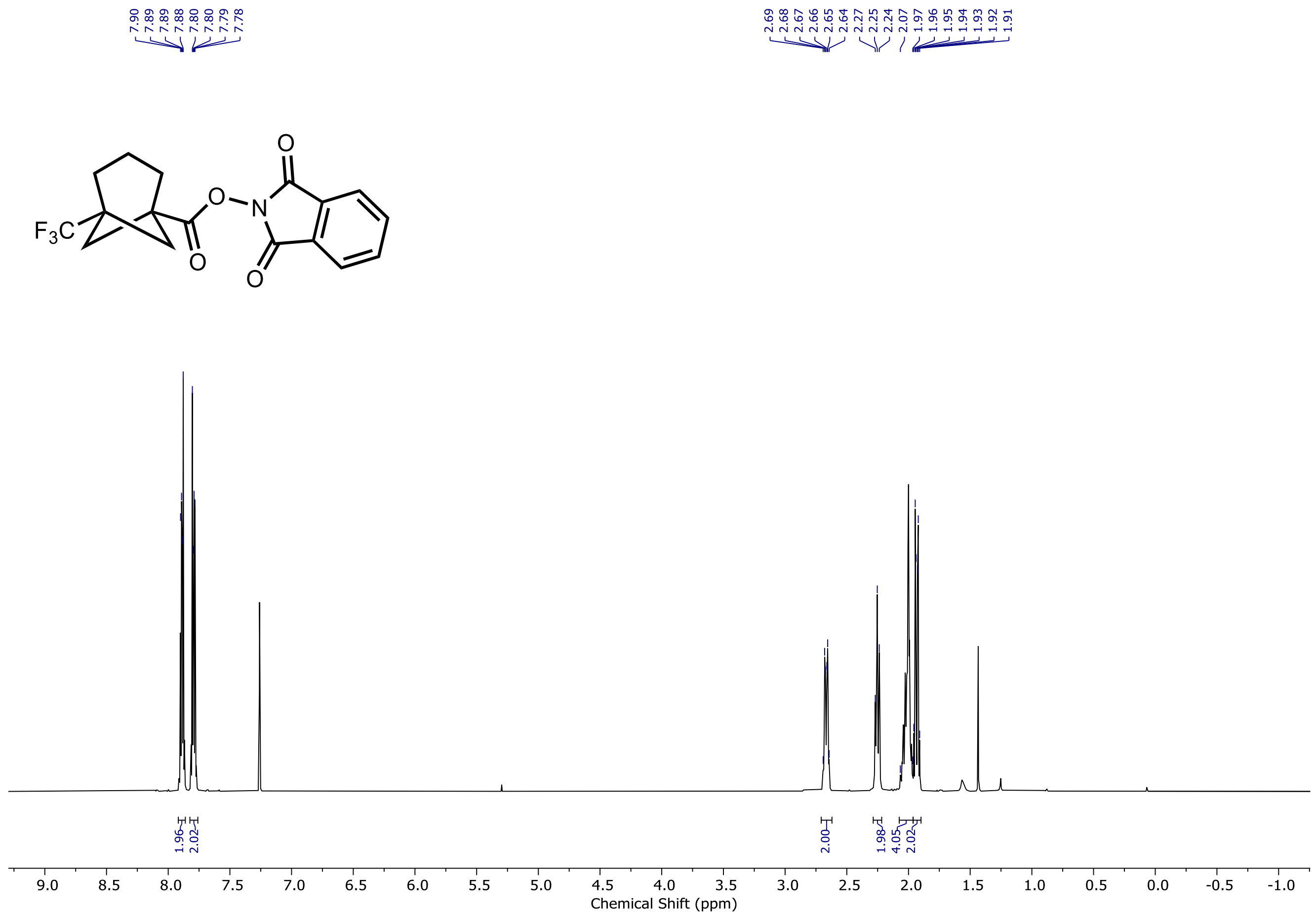

1,3-Dioxoisoindolin-2-yl 5-(trifluoromethyl)bicyclo[3.1.1]heptane-1-carboxylate **(2f)** ^{13}**C NMR (101 MHz, $CDCl_3$)**

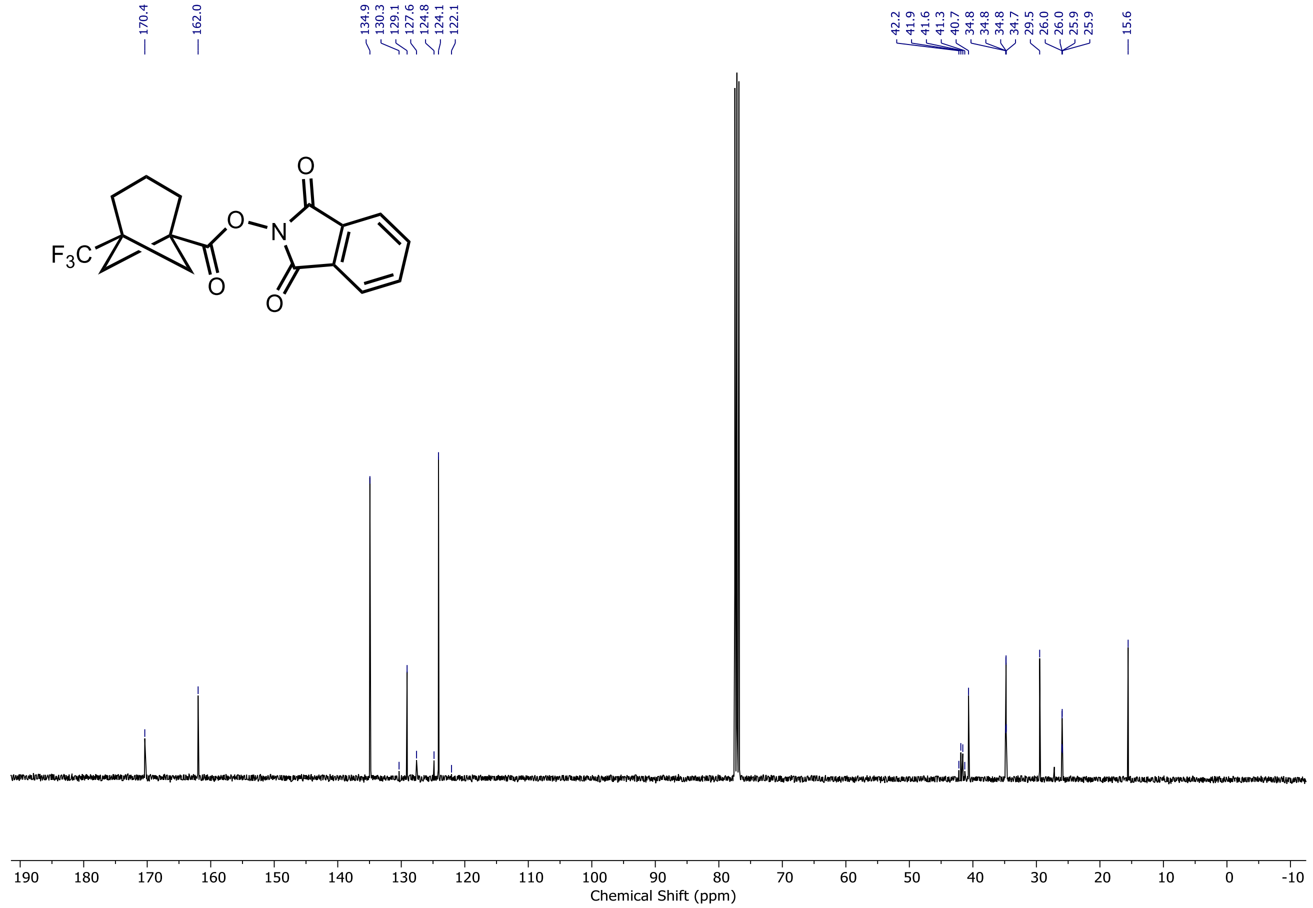

1,3-Dioxoisoindolin-2-yl 5-(trifluoromethyl)bicyclo[3.1.1]heptane-1-carboxylate **(2f)** ^{19}**F NMR (377 MHz, $CDCl_3$)**

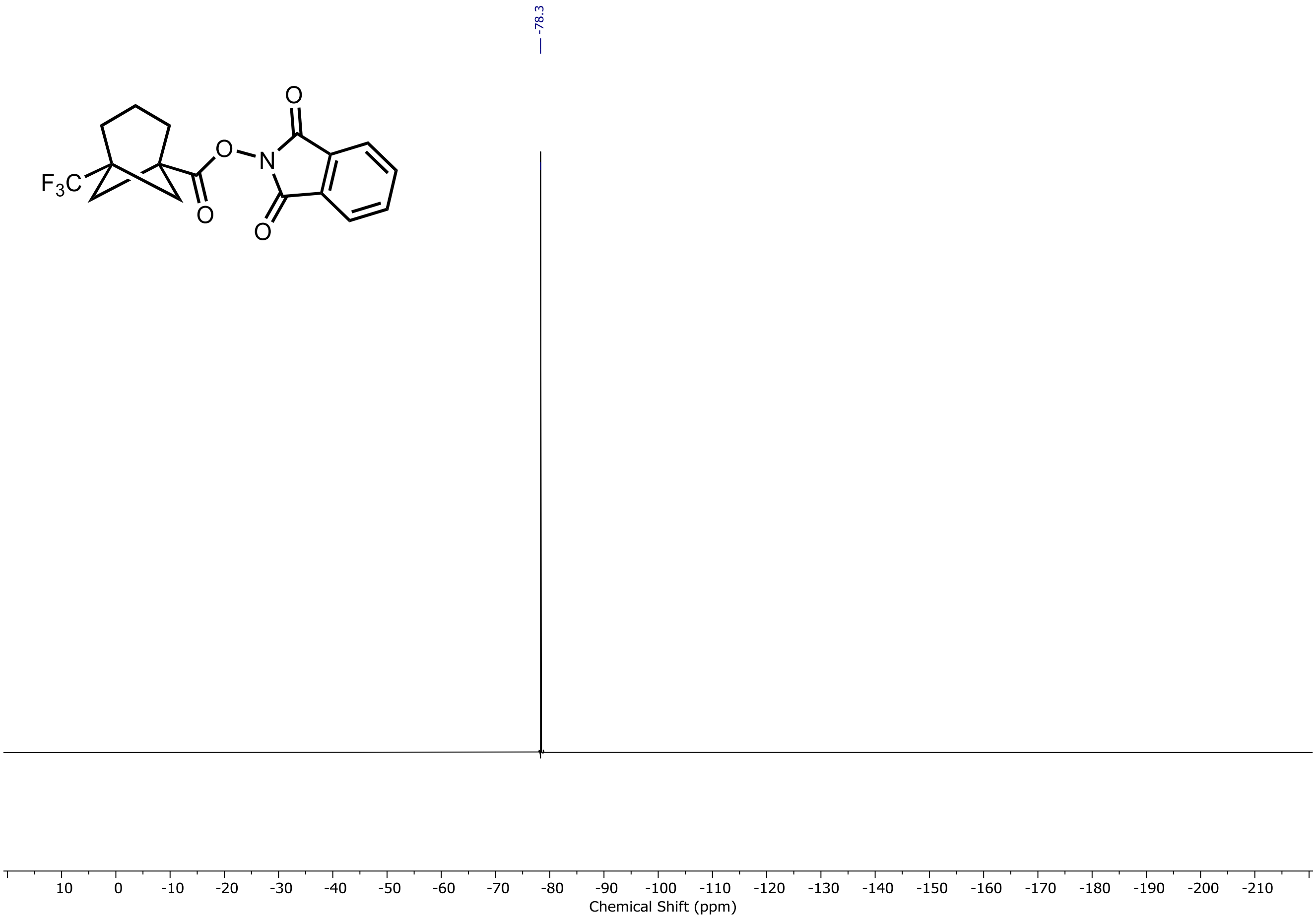
-78.3
F3C
O
N
O
O
O
10 0 -10 -20 -30 -40 -50 -60 -70 -80 -90 -100 -110 -120 -130 -140 -150 -160 -170 -180 -190 -200 -210
Chemical Shift (ppm)

Methyl 5-(4-(6-fluorobenzo[d]isoxazol-3-yl)piperidine-1-carbonyl)bicyclo[3.1.1]heptane-1-carboxylate (**12**) **^{1}H NMR (400 MHz, $CDCl_3$)**

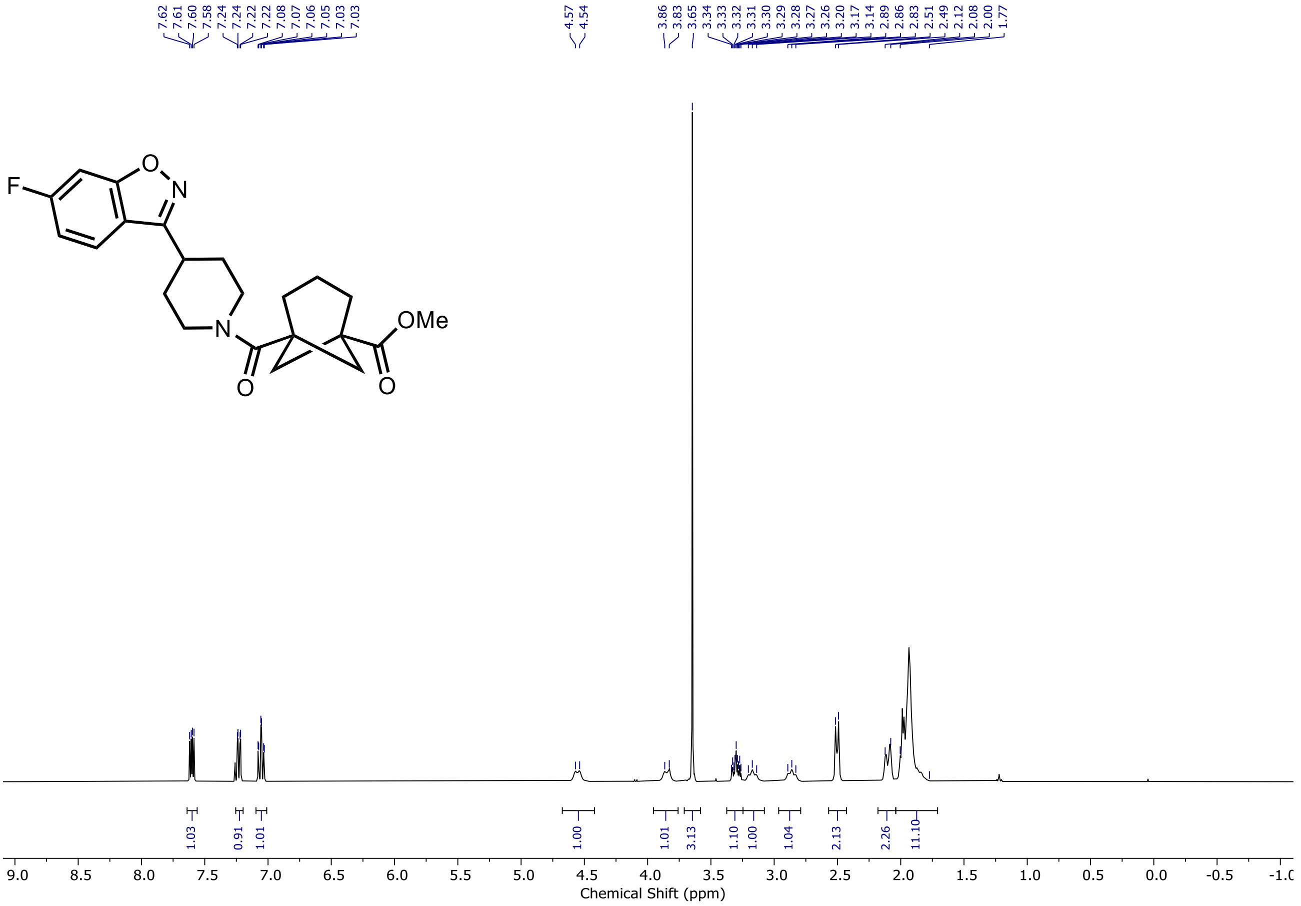

Methyl 5-(4-(6-fluorobenzo[d]isoxazol-3-yl)piperidine-1-carbonyl)bicyclo[3.1.1]heptane-1-carboxylate (**12**) **^{13}C NMR (101 MHz, $CDCl_3$)**

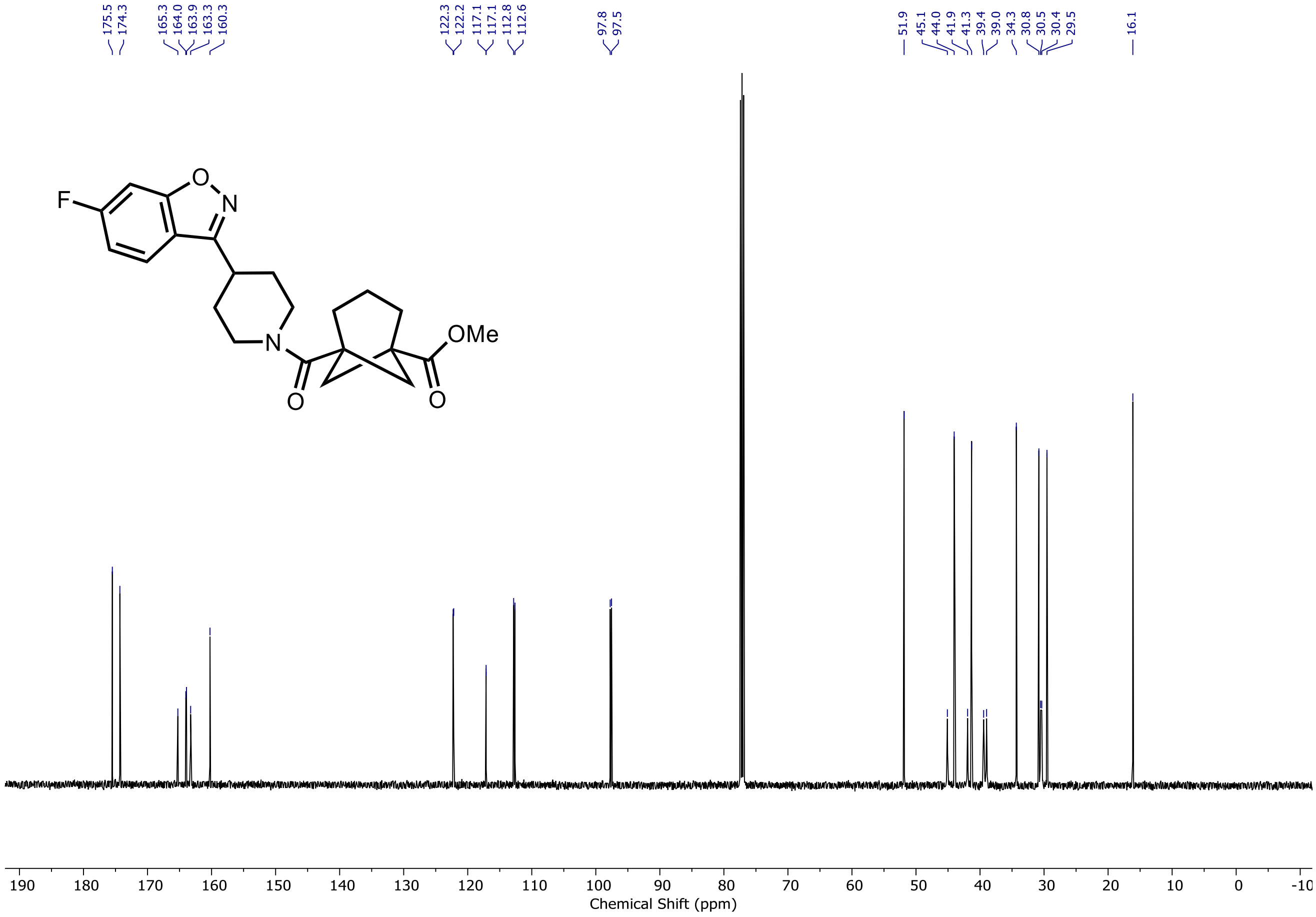

Methyl 5-(4-(6-fluorobenzo[d]isoxazol-3-yl)piperidine-1-carbonyl)bicyclo[3.1.1]heptane-1-carboxylate **(12)** ^{19}F **NMR (470 MHz, CDCl$_3$)**

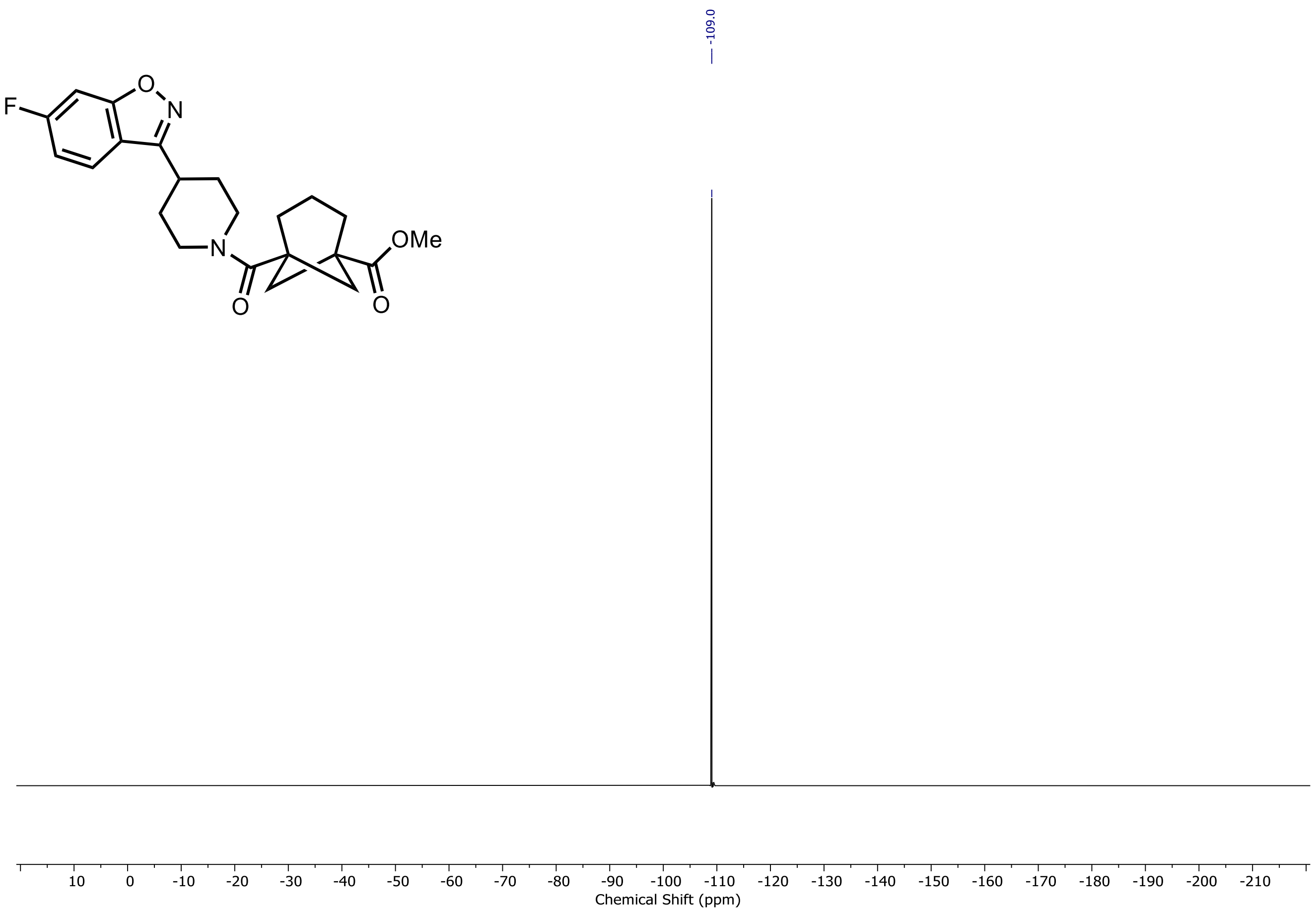

1,3-Dioxoisoindolin-2-yl 5-(4-(6-fluorobenzo[d]isoxazol-3-yl)piperidine-1-carbonyl)bicyclo[3.1.1]heptane-1-carboxylate **(2g)** ^{1}**H NMR (400 MHz, CDCl$_3$)**

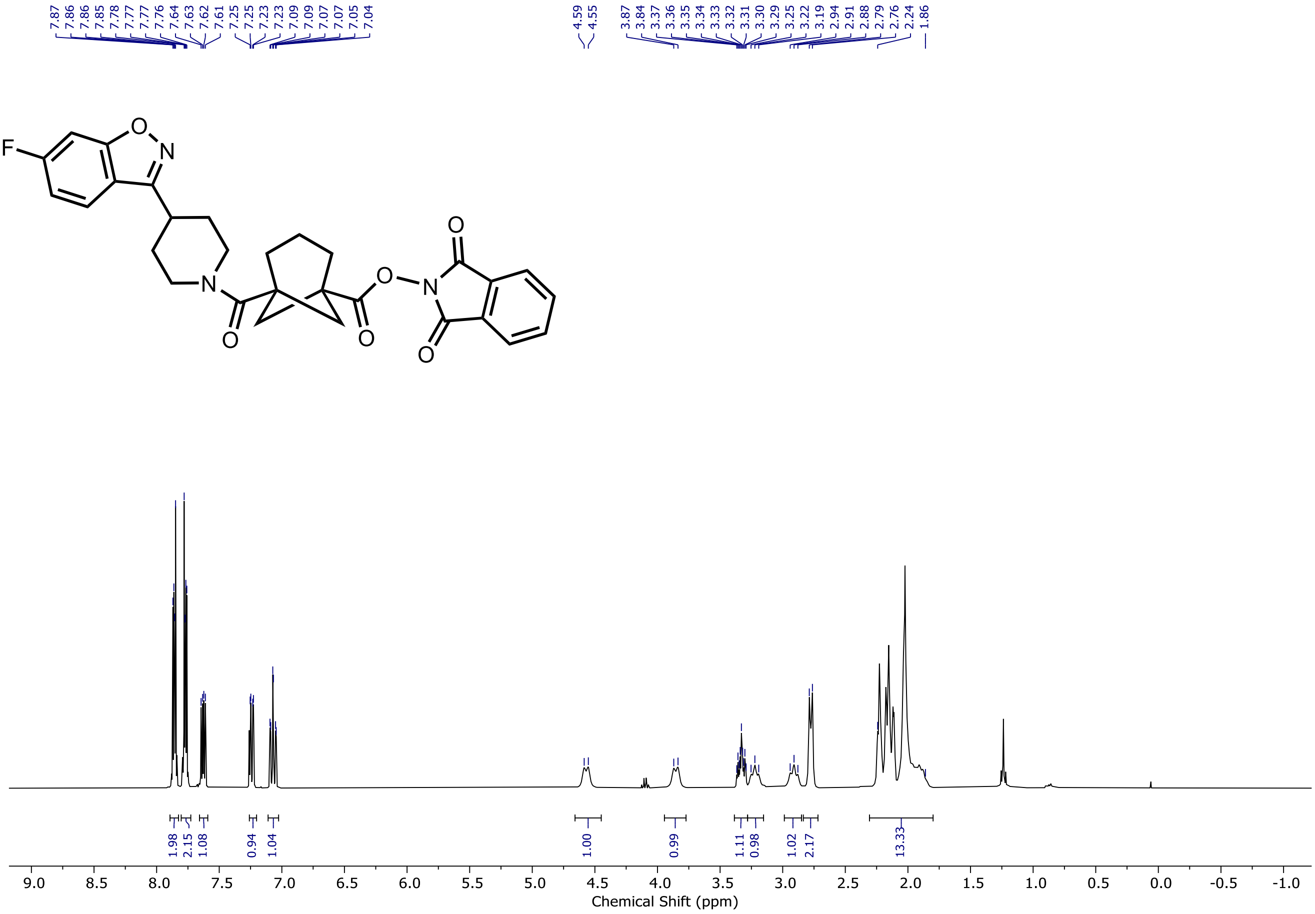

1,3-Dioxoisoindolin-2-yl 5-(4-(6-fluorobenzo[d]isoxazol-3-yl)piperidine-1-carbonyl)bicyclo[3.1.1]heptane-1-carboxylate **(2g)** ^{13}**C NMR (101 MHz, CDCl$_3$)**

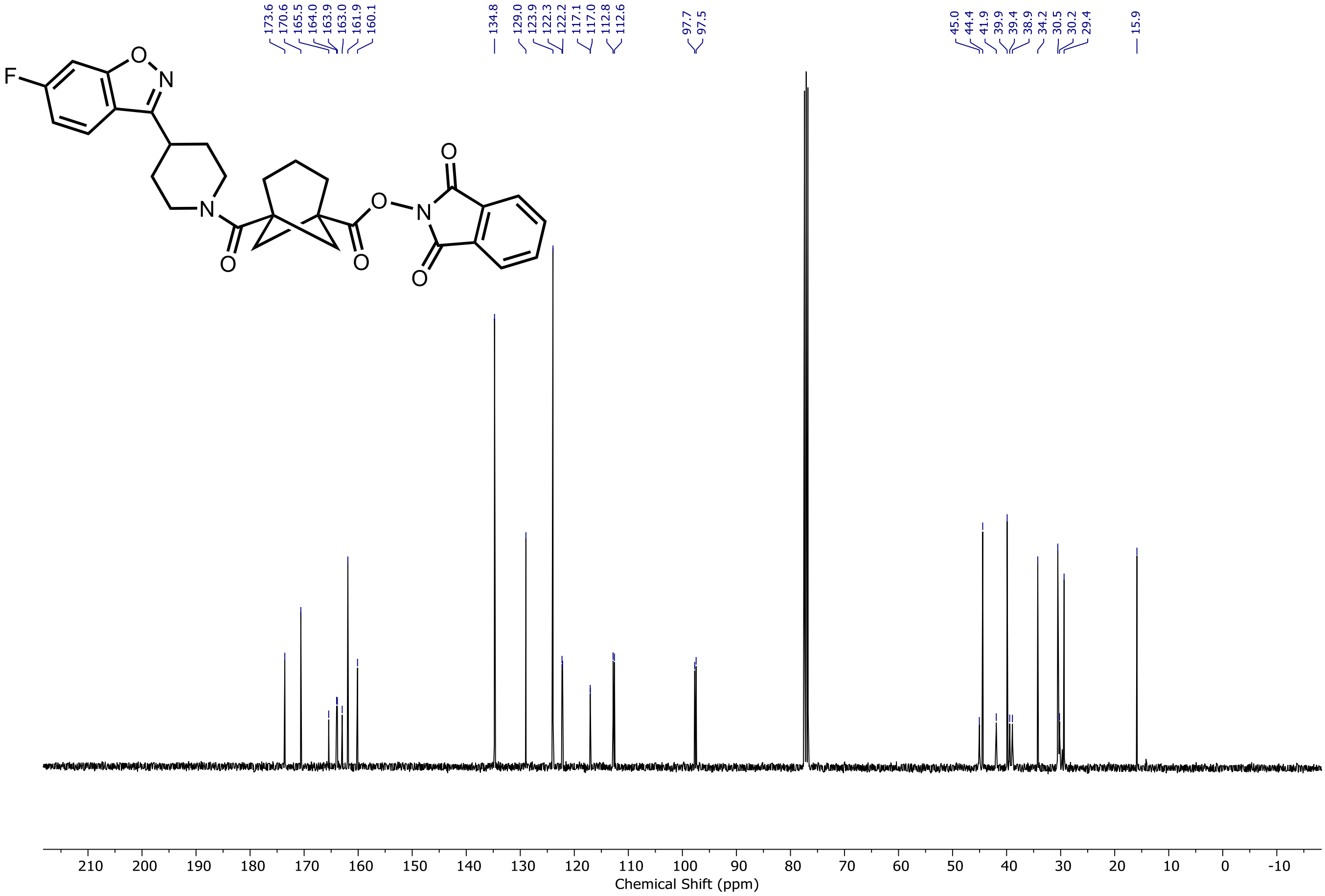

1,3-Dioxoisoindolin-2-yl 5-(4-(6-fluorobenzo[d]isoxazol-3-yl)piperidine-1-carbonyl)bicyclo[3.1.1]heptane-1-carboxylate **(2g)** ^{19}**F NMR (377 MHz, CDCl$_3$)**

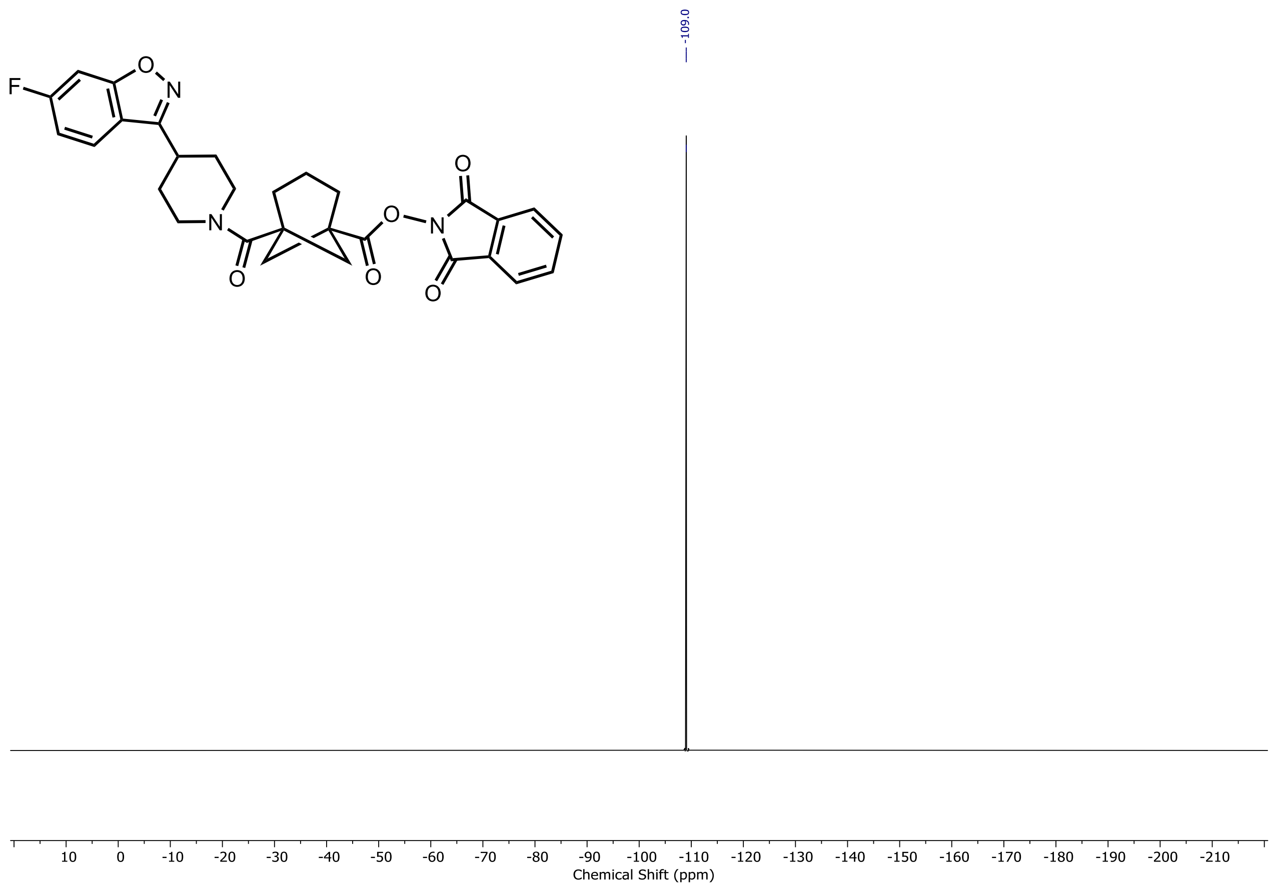

3-(*tert*-Butyl) 1-(1,3-dioxoisoindolin-2-yl) 3-azabicyclo[3.1.1]heptane-1,3-dicarboxylate (**6a**) **^{1}H NMR (600 MHz, $CDCl_3$)**

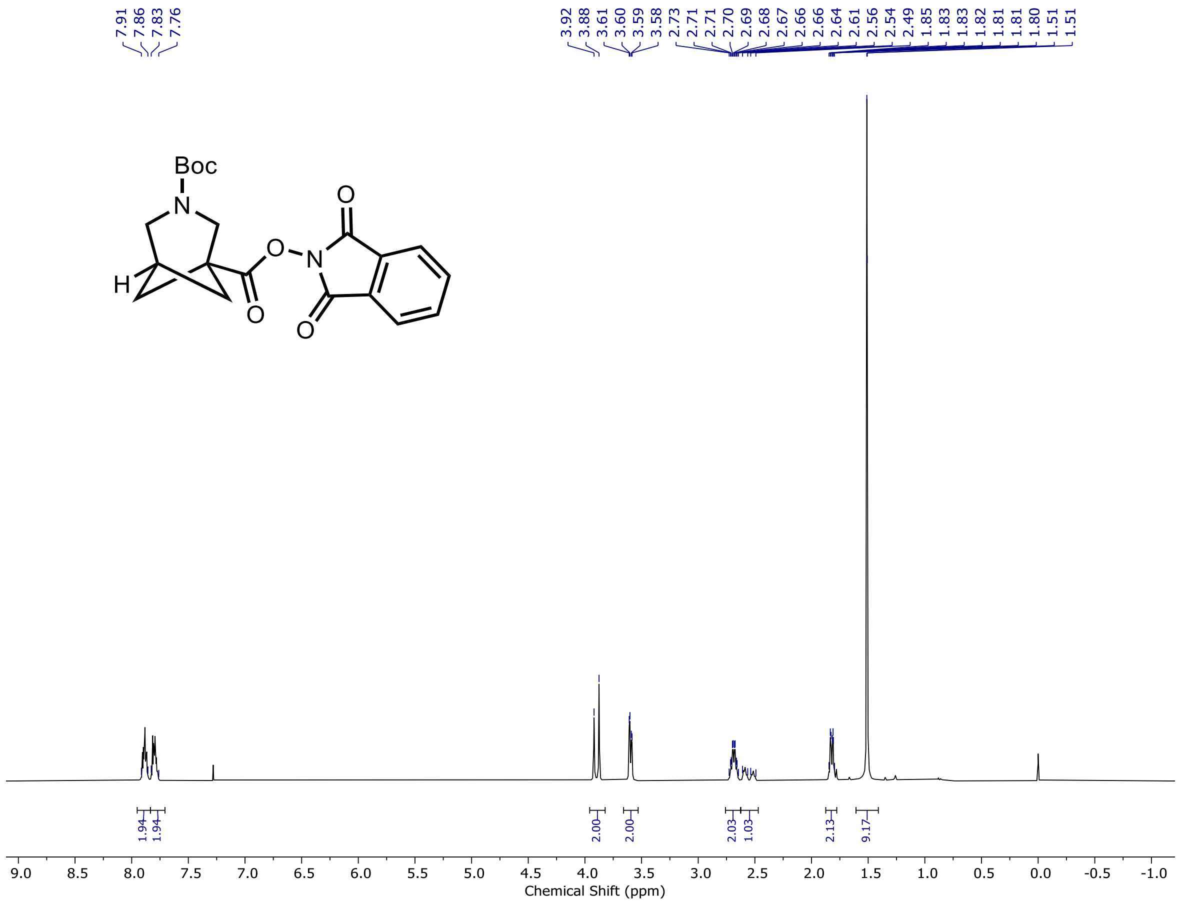

3-(*tert*-Butyl) 1-(1,3-dioxoisoindolin-2-yl) 3-azabicyclo[3.1.1]heptane-1,3-dicarboxylate (**6a**) **^{13}C NMR (101 MHz, $CDCl_3$)**

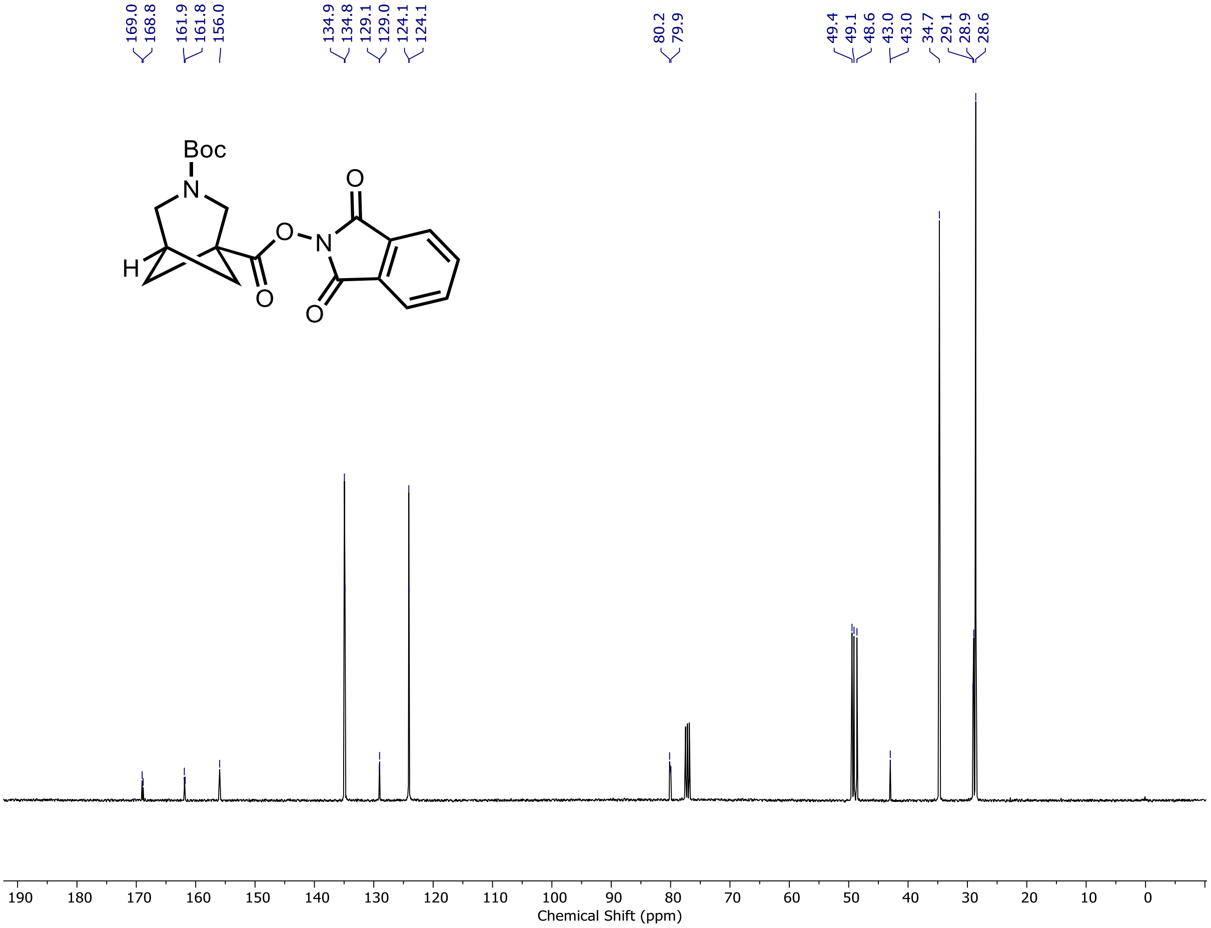

3-(*tert*-Butyl) 1-(1,3-dioxoisoindolin-2-yl) 5-methyl 3-azabicyclo[3.1.1]heptane-1,3,5-tricarboxylate **(6b)** ^{1}**H NMR (500 MHz,** $CDCl_3$**)**

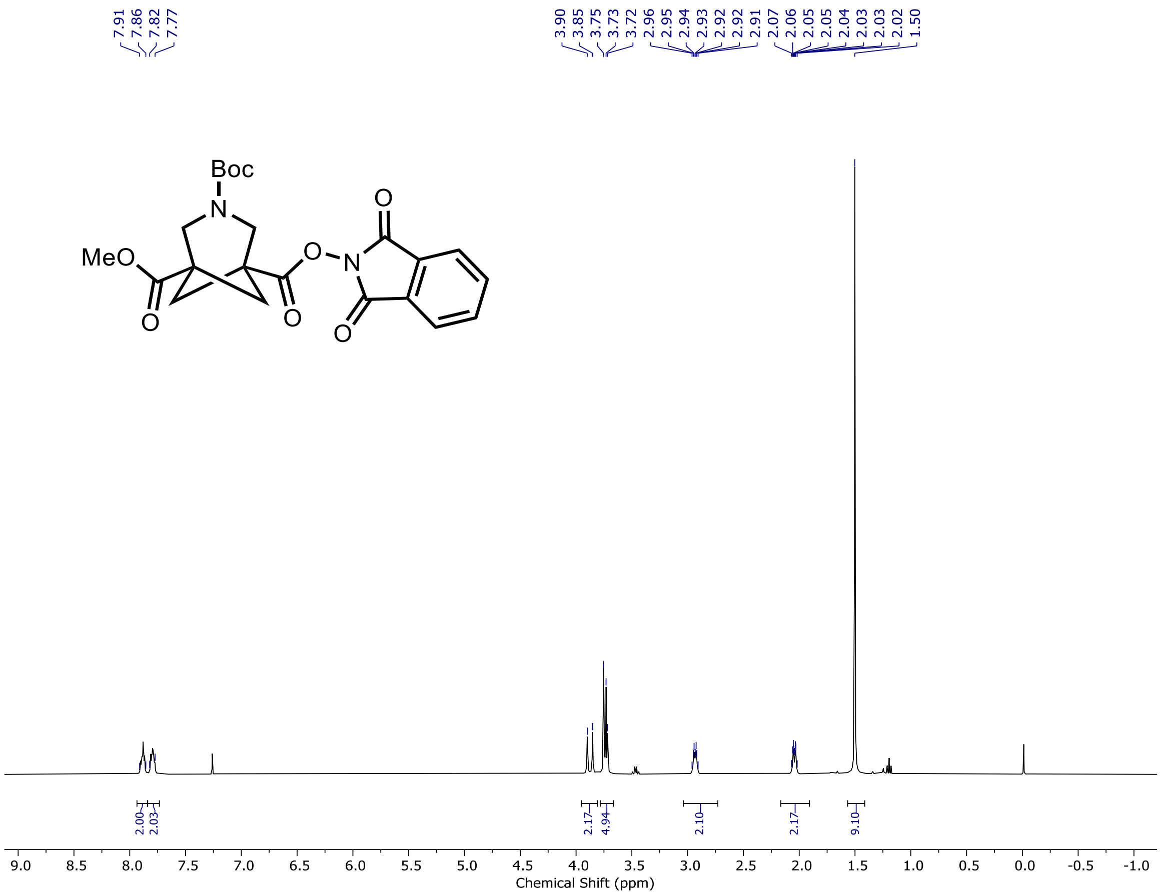

3-(*tert*-Butyl) 1-(1,3-dioxoisoindolin-2-yl) 5-methyl 3-azabicyclo[3.1.1]heptane-1,3,5-tricarboxylate **(6b)** ^{13}**C NMR (101 MHz,** $CDCl_3$**)**

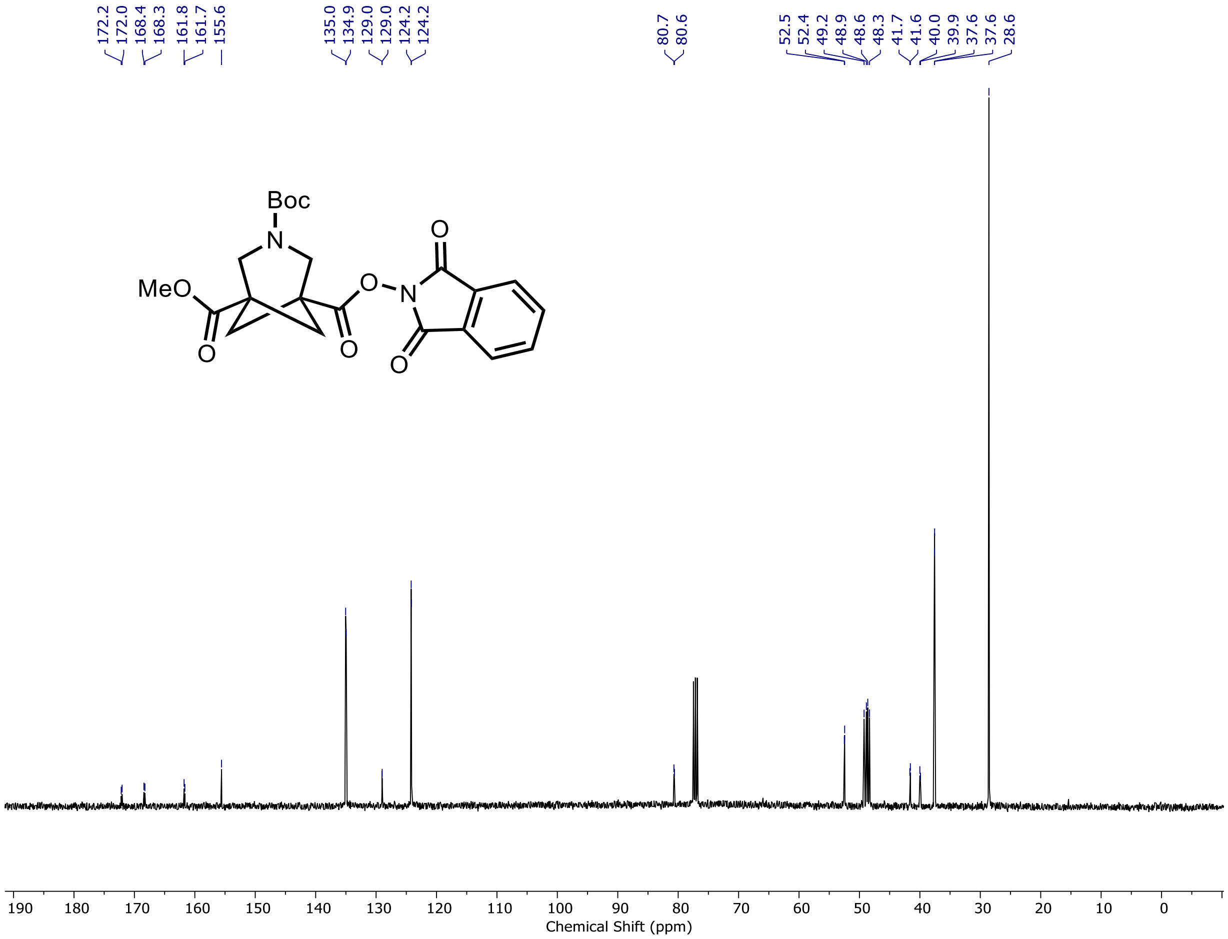

Methyl 5-(4-methylquinolin-2-yl)bicyclo[3.1.1]heptane-1-carboxylate **(3a)** ^{1}H NMR (400 MHz, $CDCl_3$)

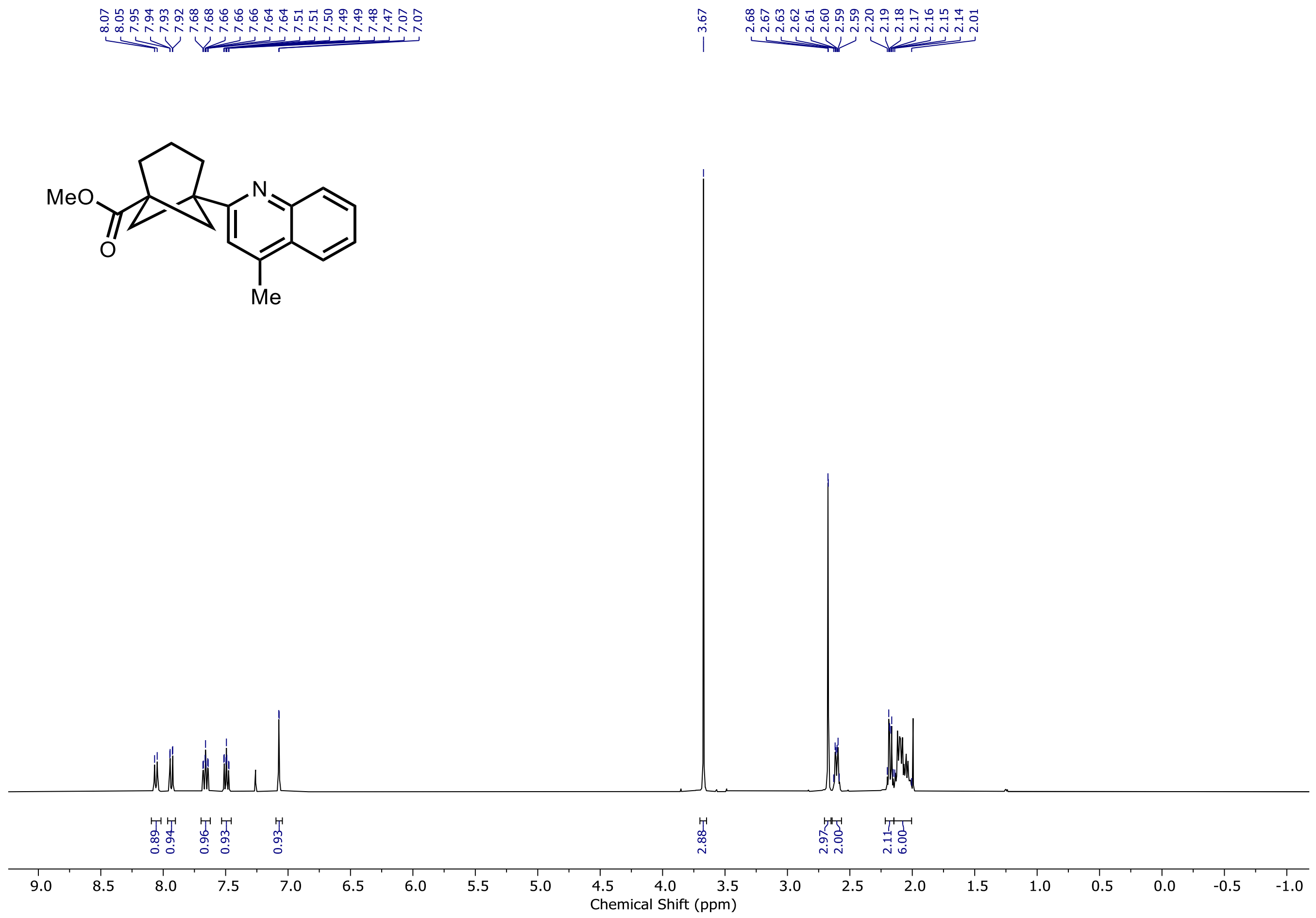

Methyl 5-(4-methylquinolin-2-yl)bicyclo[3.1.1]heptane-1-carboxylate **(3a)** ^{13}C NMR (101 MHz, $CDCl_3$)

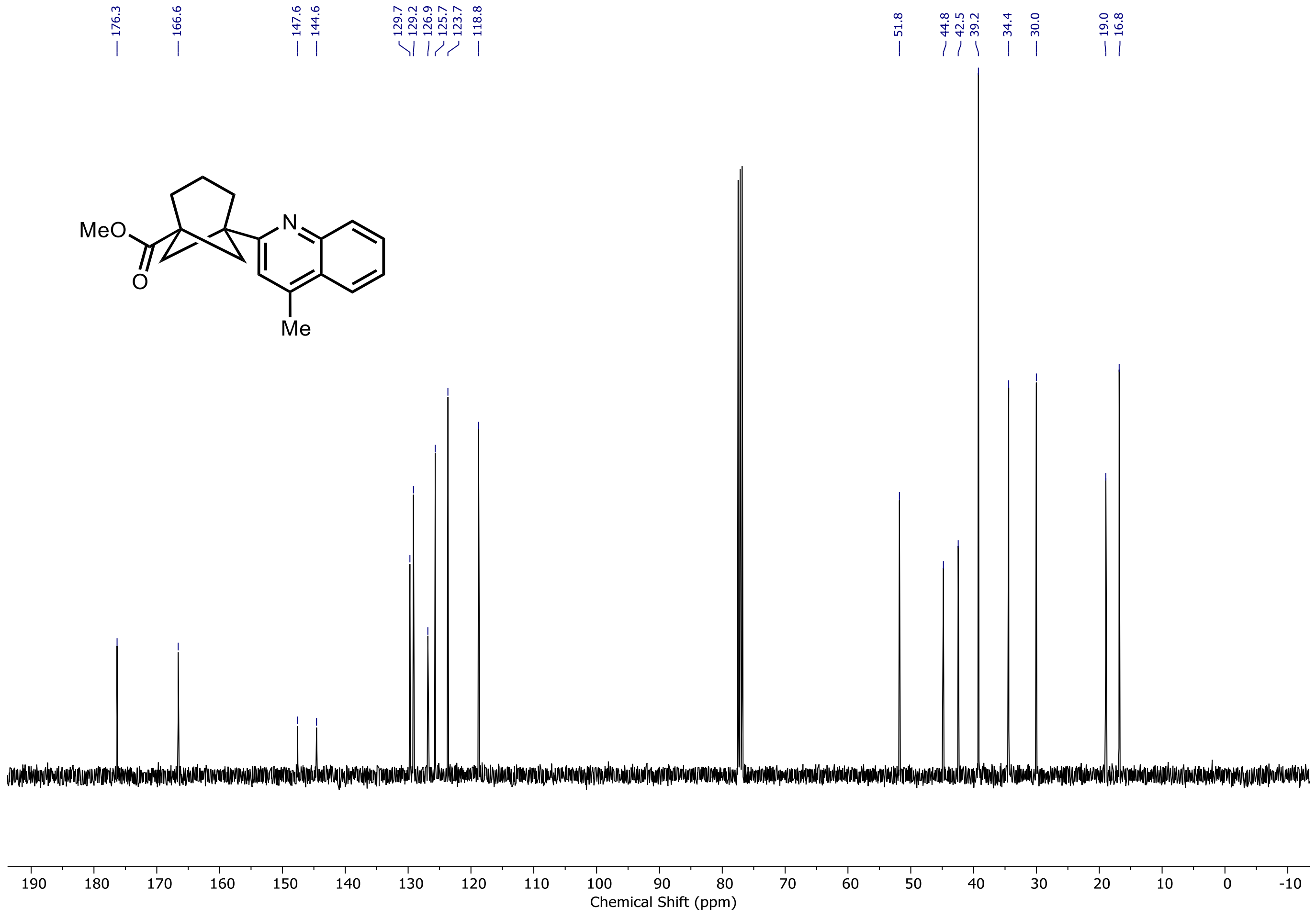

Methyl 5-(3-methylisoquinolin-1-yl)bicyclo[3.1.1]heptane-1-carboxylate **(3b)** ^{1}H NMR **(400 MHz, CDCl$_3$)**

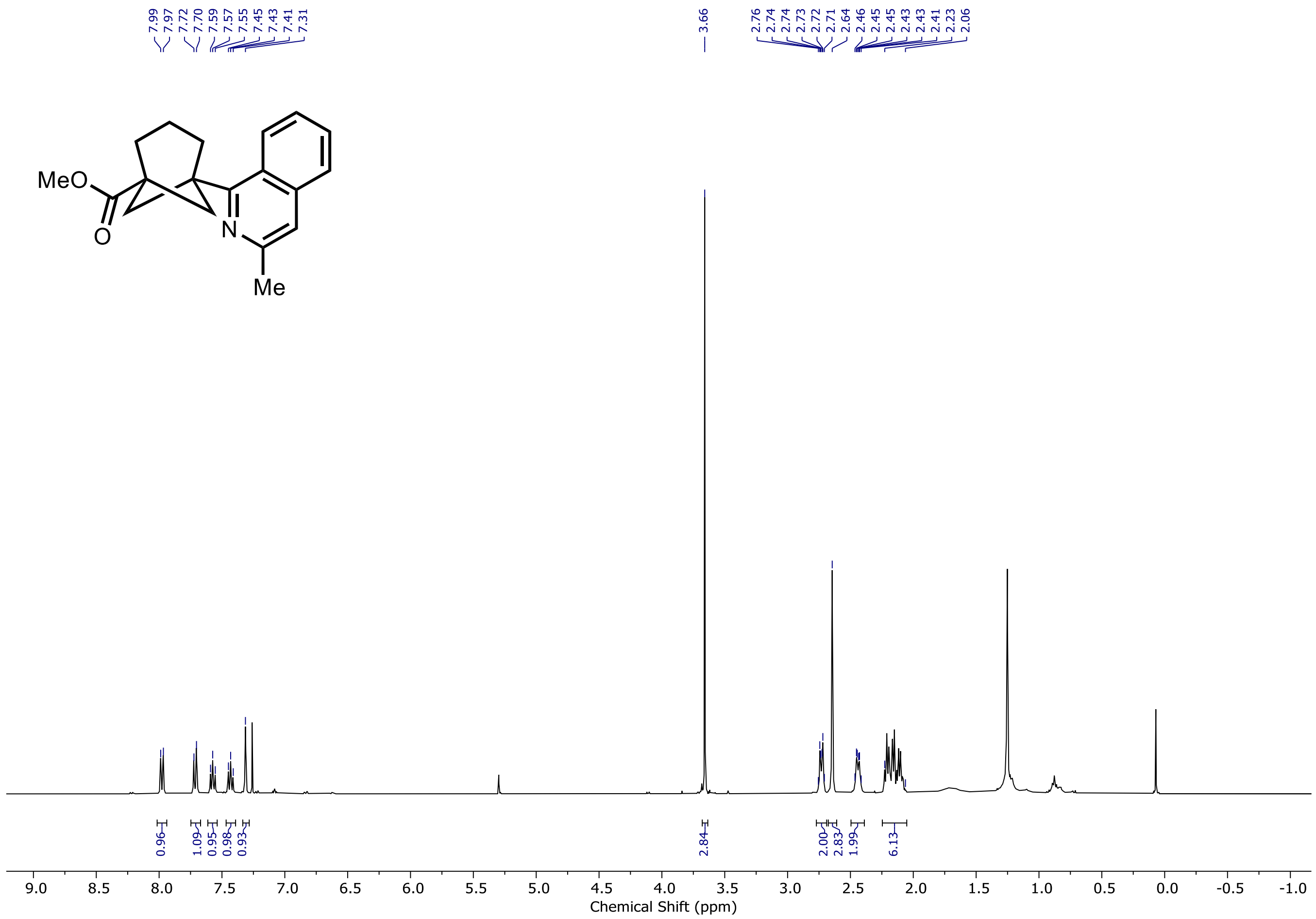

Methyl 5-(3-methylisoquinolin-1-yl)bicyclo[3.1.1]heptane-1-carboxylate **(3b)** ^{13}C NMR **(101 MHz, CDCl$_3$)**

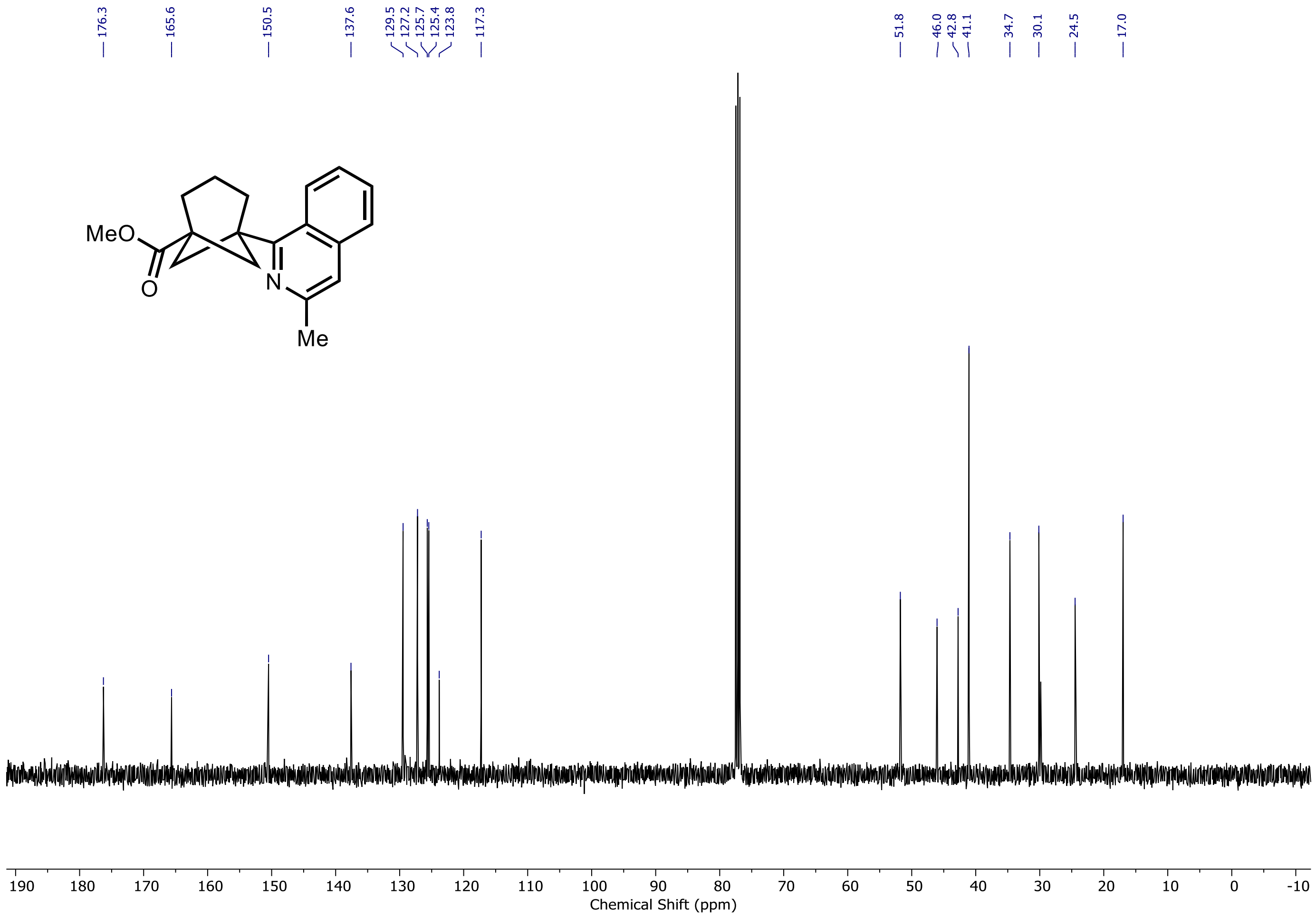

Methyl 5-(isoquinolin-1-yl)bicyclo[3.1.1]heptane-1-carboxylate (**3c**) **^{1}H NMR (400 MHz, $CDCl_3$)**

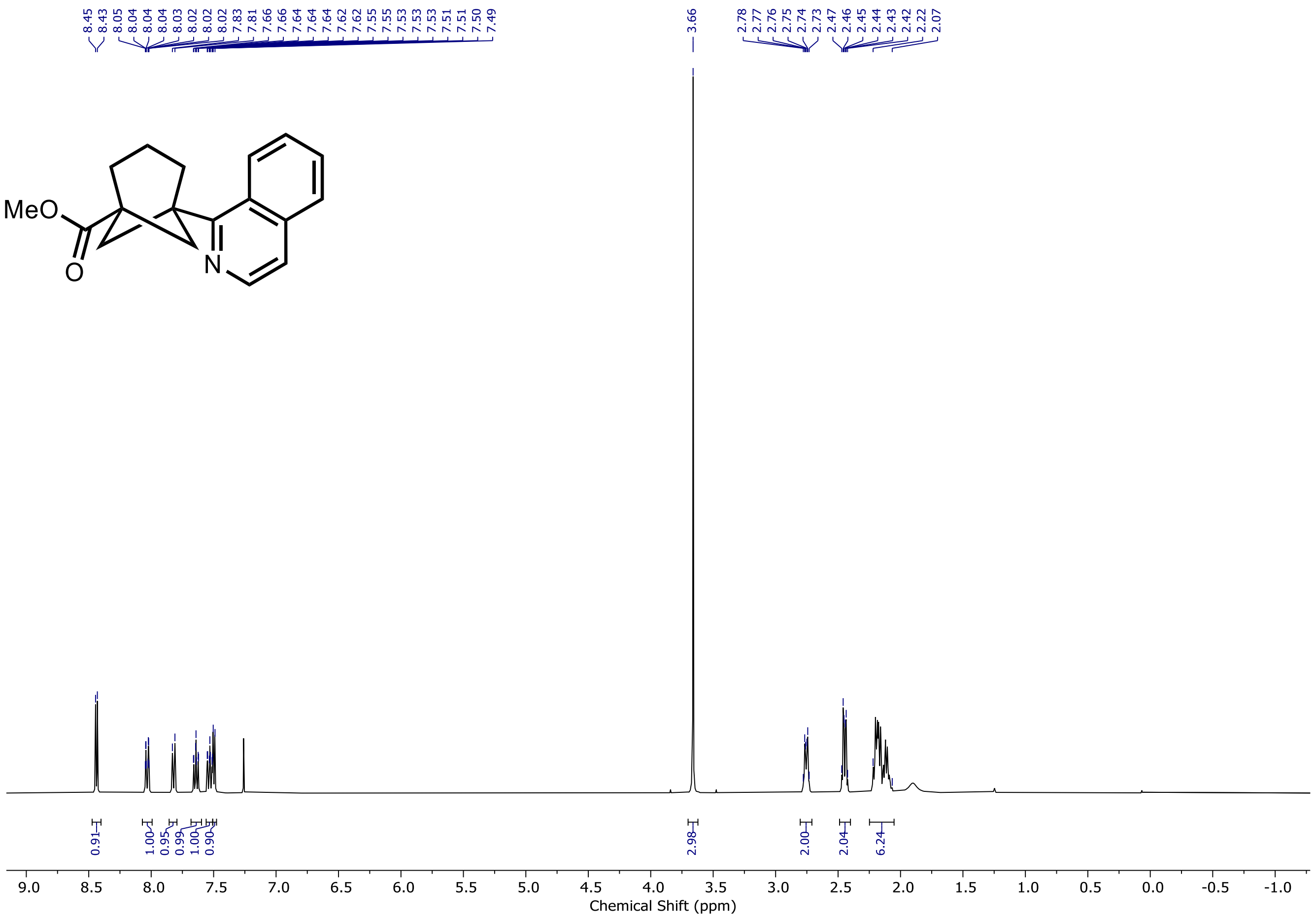

Methyl 5-(isoquinolin-1-yl)bicyclo[3.1.1]heptane-1-carboxylate (**3c**) **^{13}C NMR (101 MHz, $CDCl_3$)**

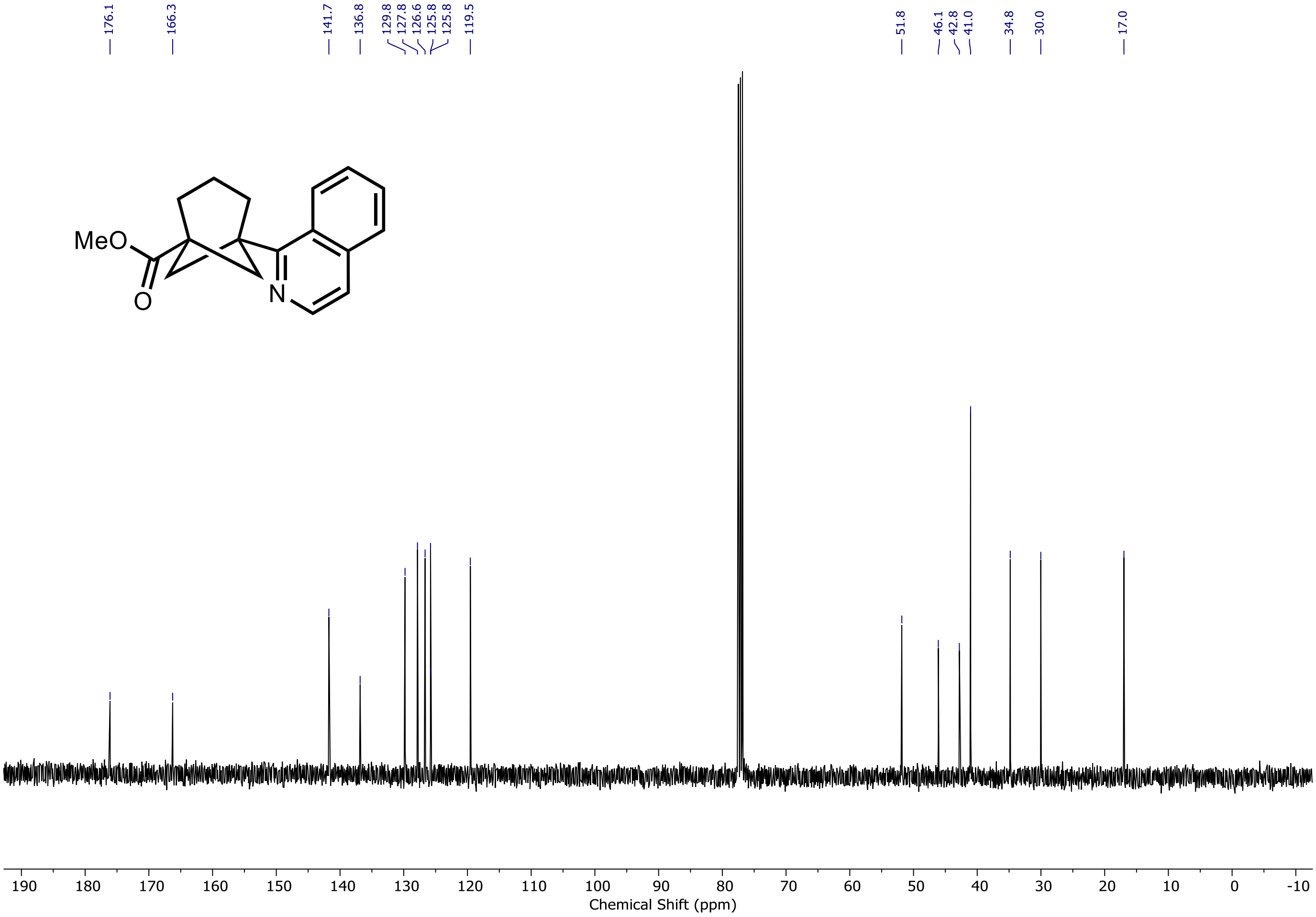

Methyl 5-(4-methylpyridin-2-yl)bicyclo[3.1.1]heptane-1-carboxylate **(3d)** **^{1}H NMR (400 MHz, CDCl$_3$)**

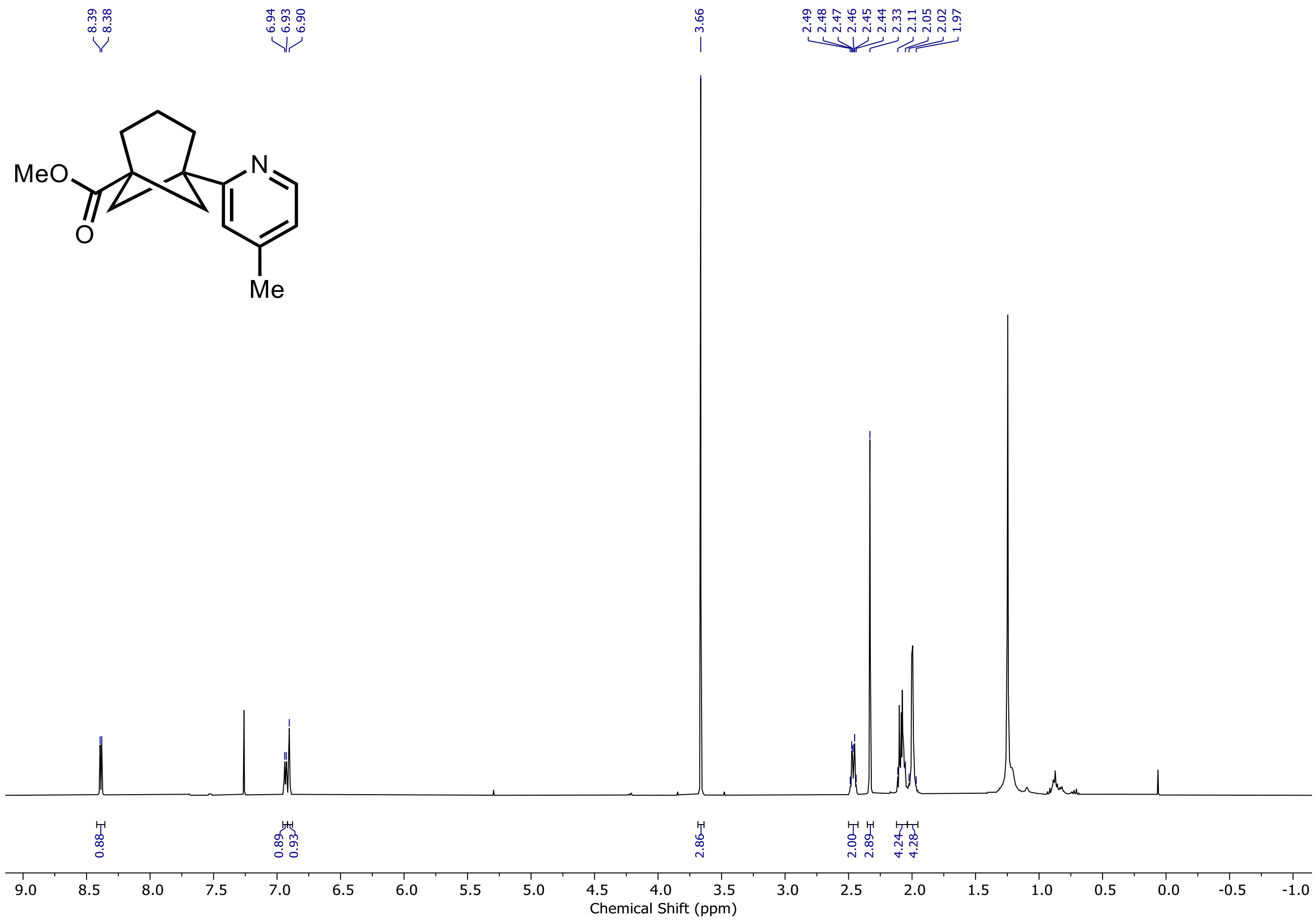

Methyl 5-(4-methylpyridin-2-yl)bicyclo[3.1.1]heptane-1-carboxylate **(3d)** **^{13}C NMR (151 MHz, CDCl$_3$)**

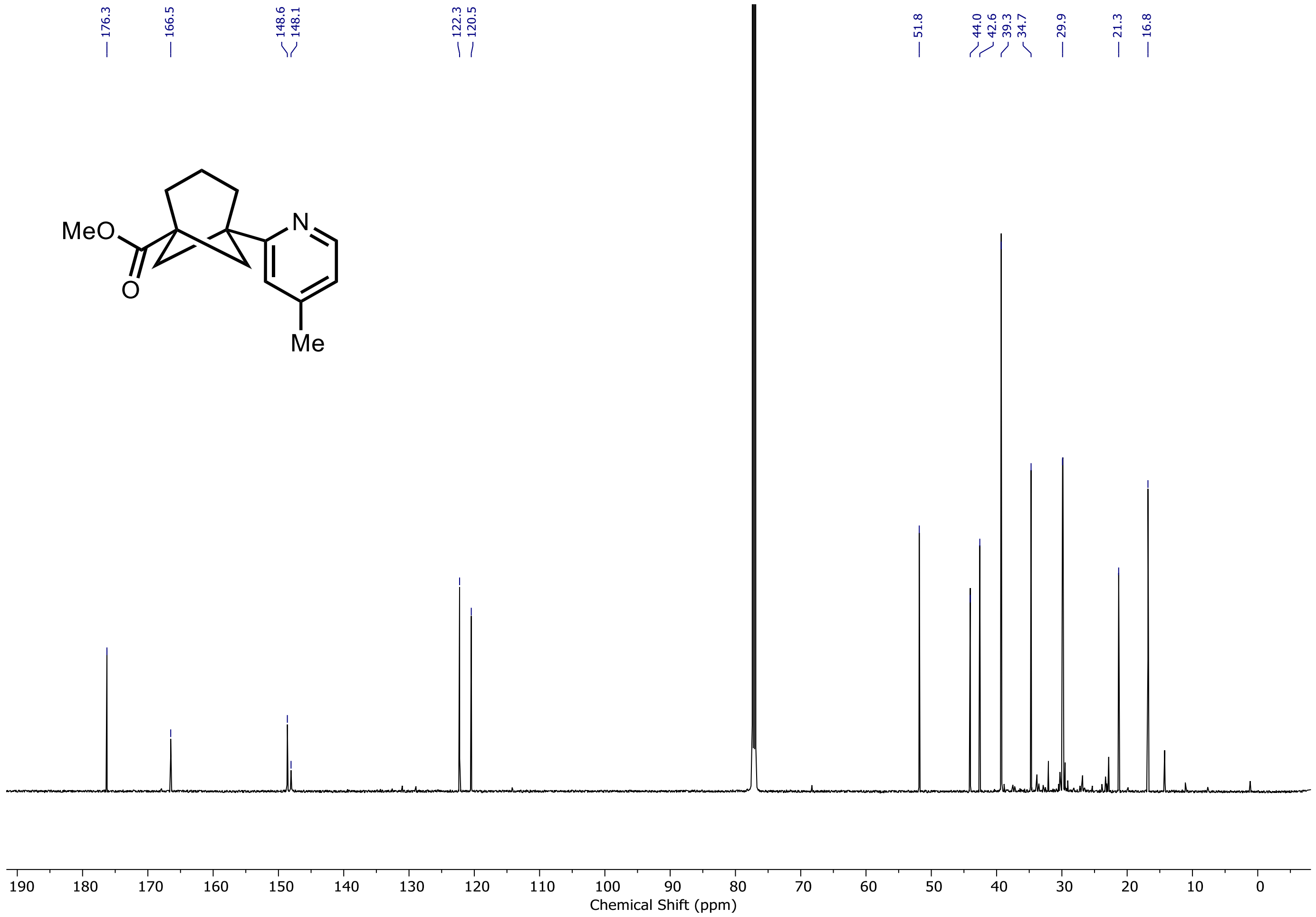

Methyl 5-(quinoxalin-2-yl)bicyclo[3.1.1]heptane-1-carboxylate **(3e)** **^{1}H NMR (400 MHz, $CDCl_3$)**

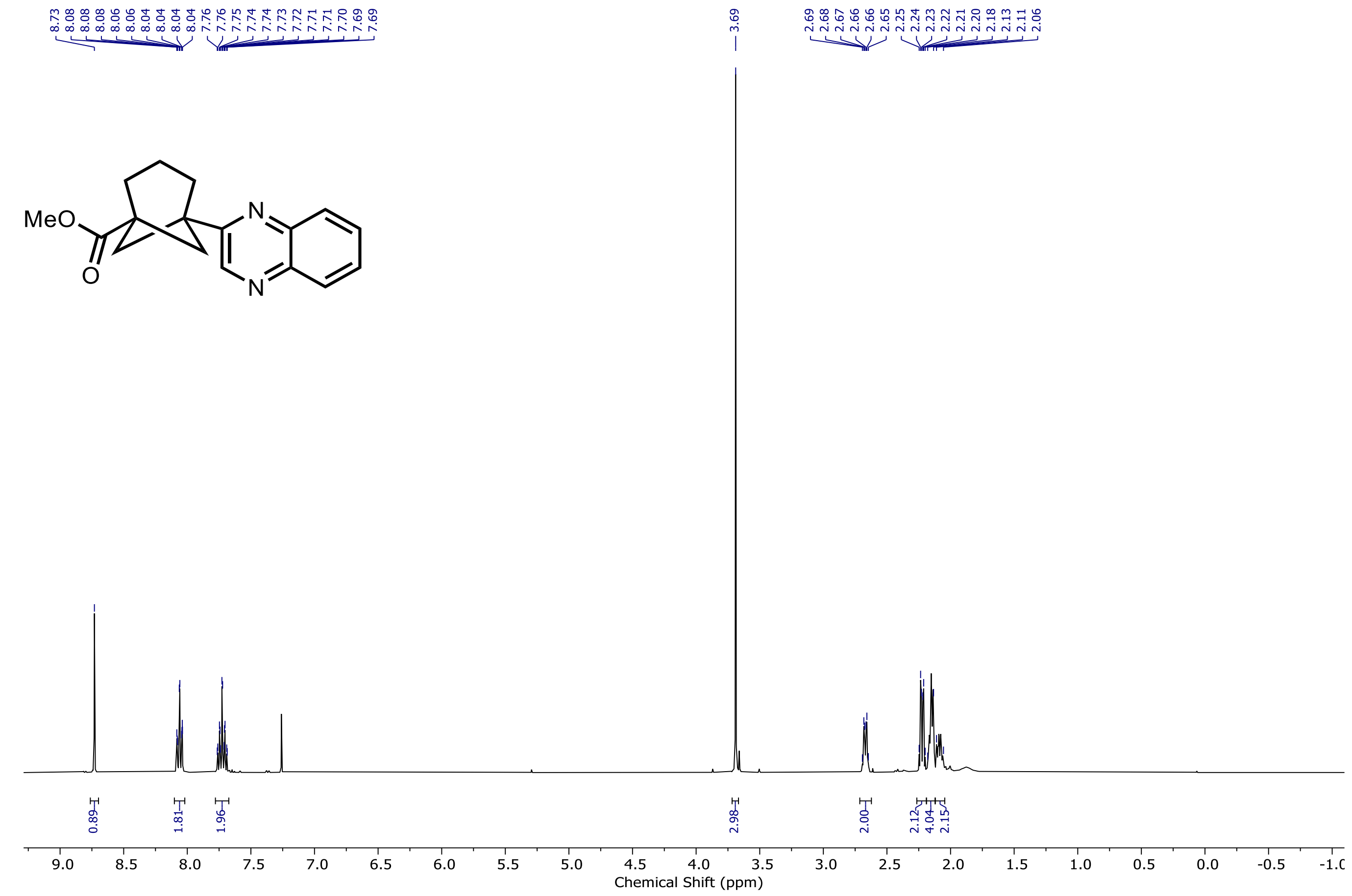

Methyl 5-(quinoxalin-2-yl)bicyclo[3.1.1]heptane-1-carboxylate **(3e)** **^{13}C NMR (101 MHz, $CDCl_3$)**

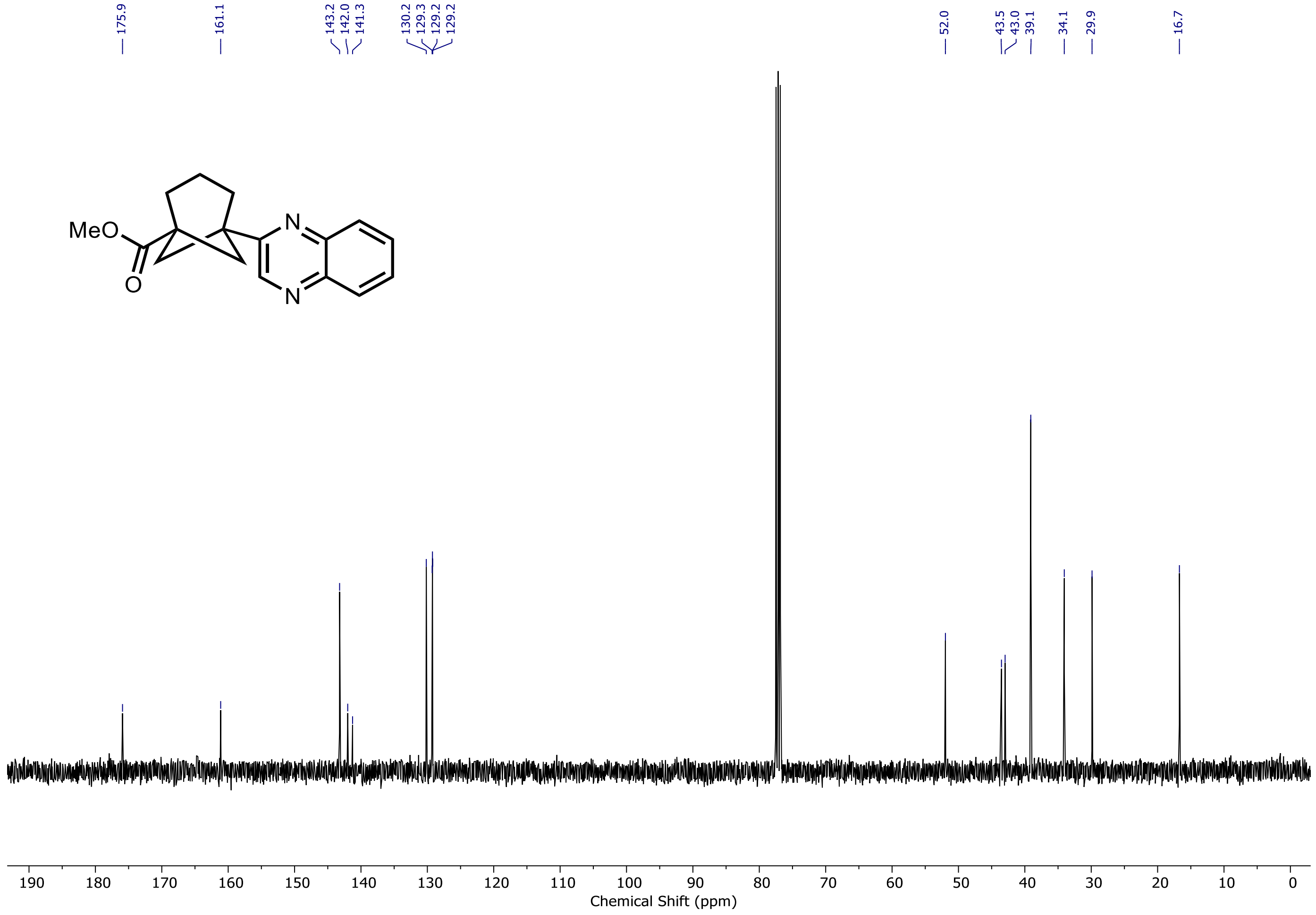

Methyl 5-(pyridin-2-yl)bicyclo[3.1.1]heptane-1-carboxylate **(3f)** and methyl 5-(pyridin-4-yl)bicyclo[3.1.1]heptane-1-carboxylate **(3f') ^{1}H NMR (600 MHz, $CDCl_3$)**

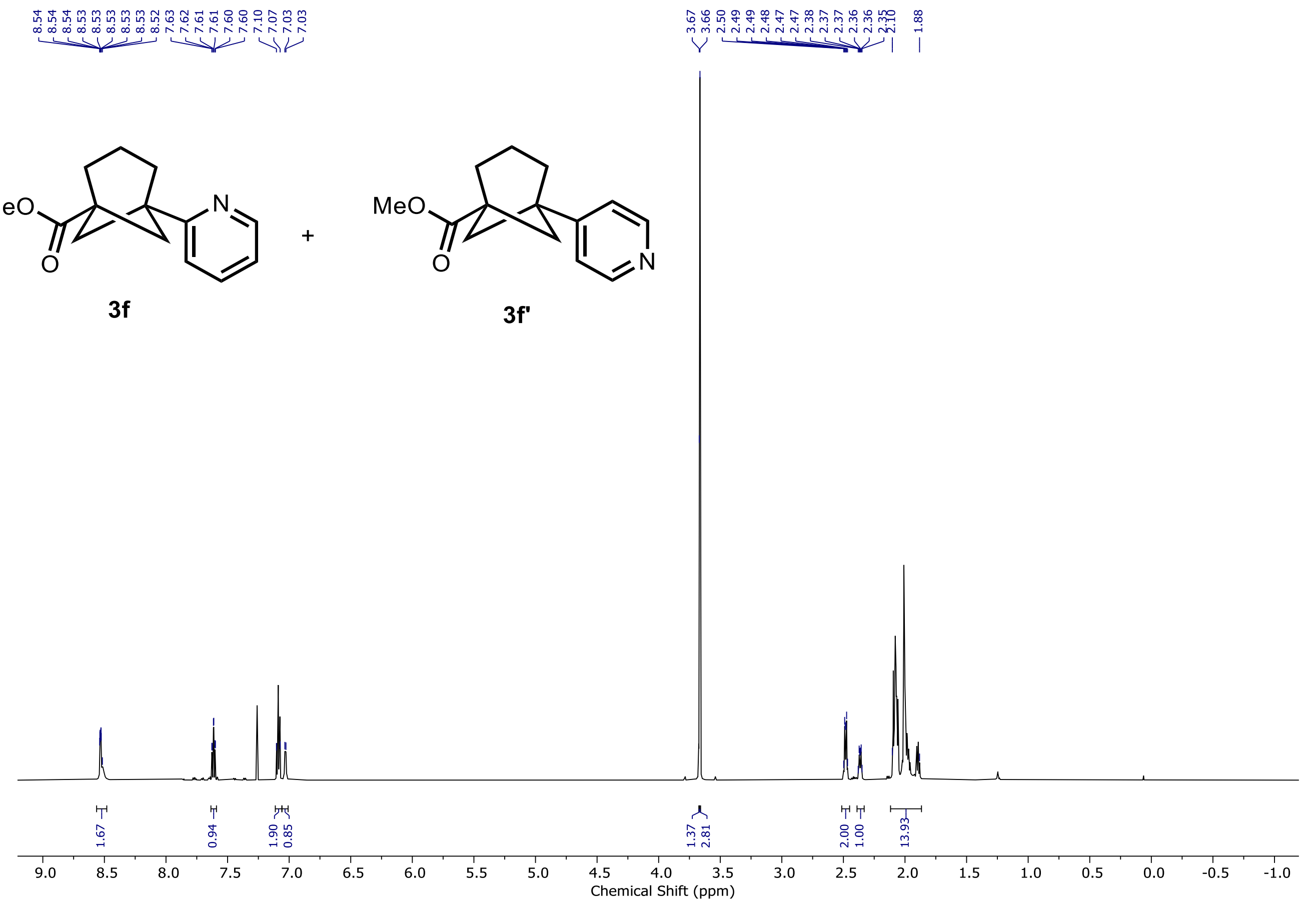

Methyl 5-(pyridin-2-yl)bicyclo[3.1.1]heptane-1-carboxylate and methyl 5-(pyridin-4-yl)bicyclo[3.1.1]heptane-1-carboxylate **(3f) and (3f') ^{13}C NMR (151 MHz, $CDCl_3$)**

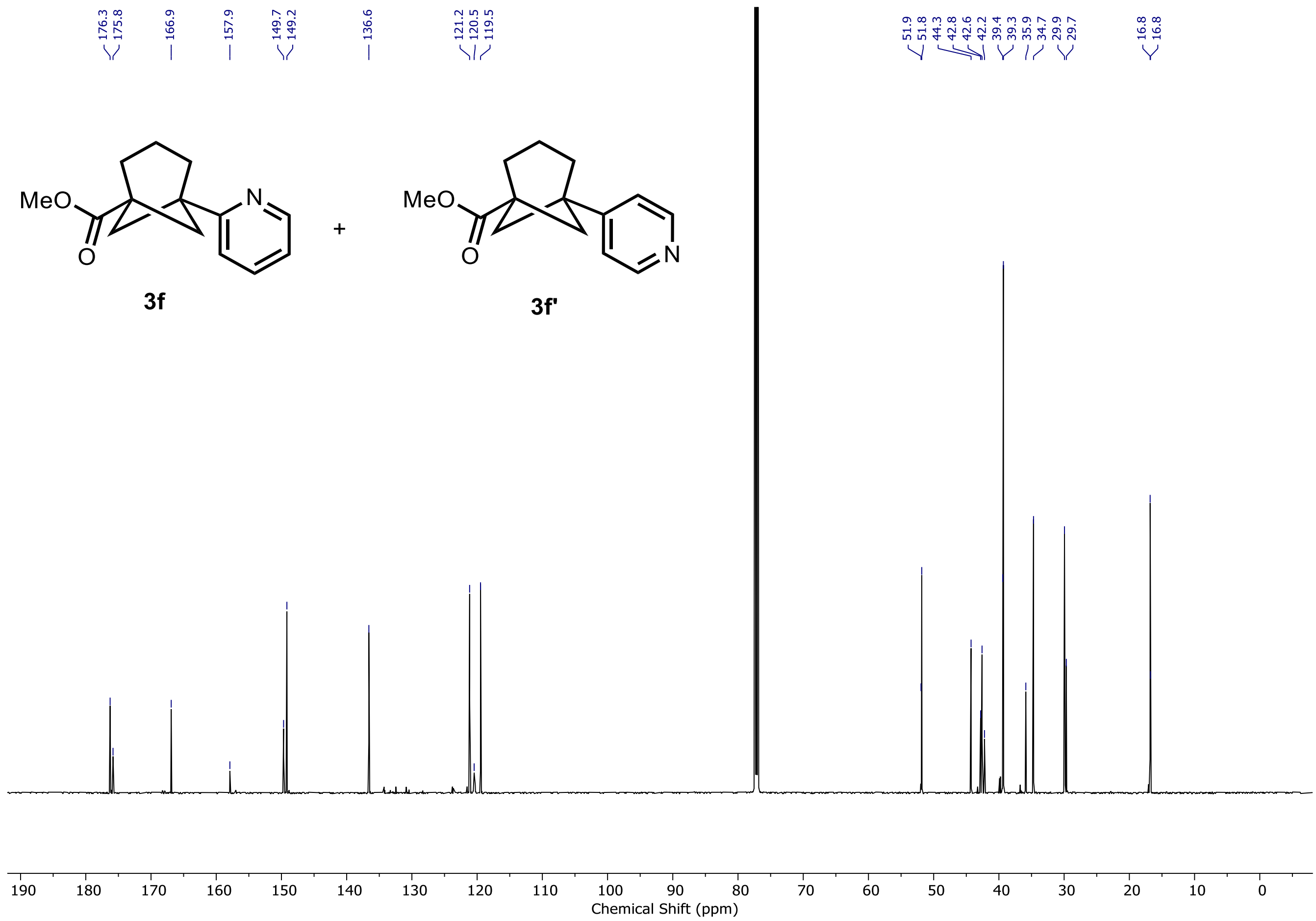

Methyl 5-([2,2'-bipyridin]-6-yl)bicyclo[3.1.1]heptane-1-carboxylate **(3g)** **^{1}H NMR (400 MHz, CDCl$_3$)**

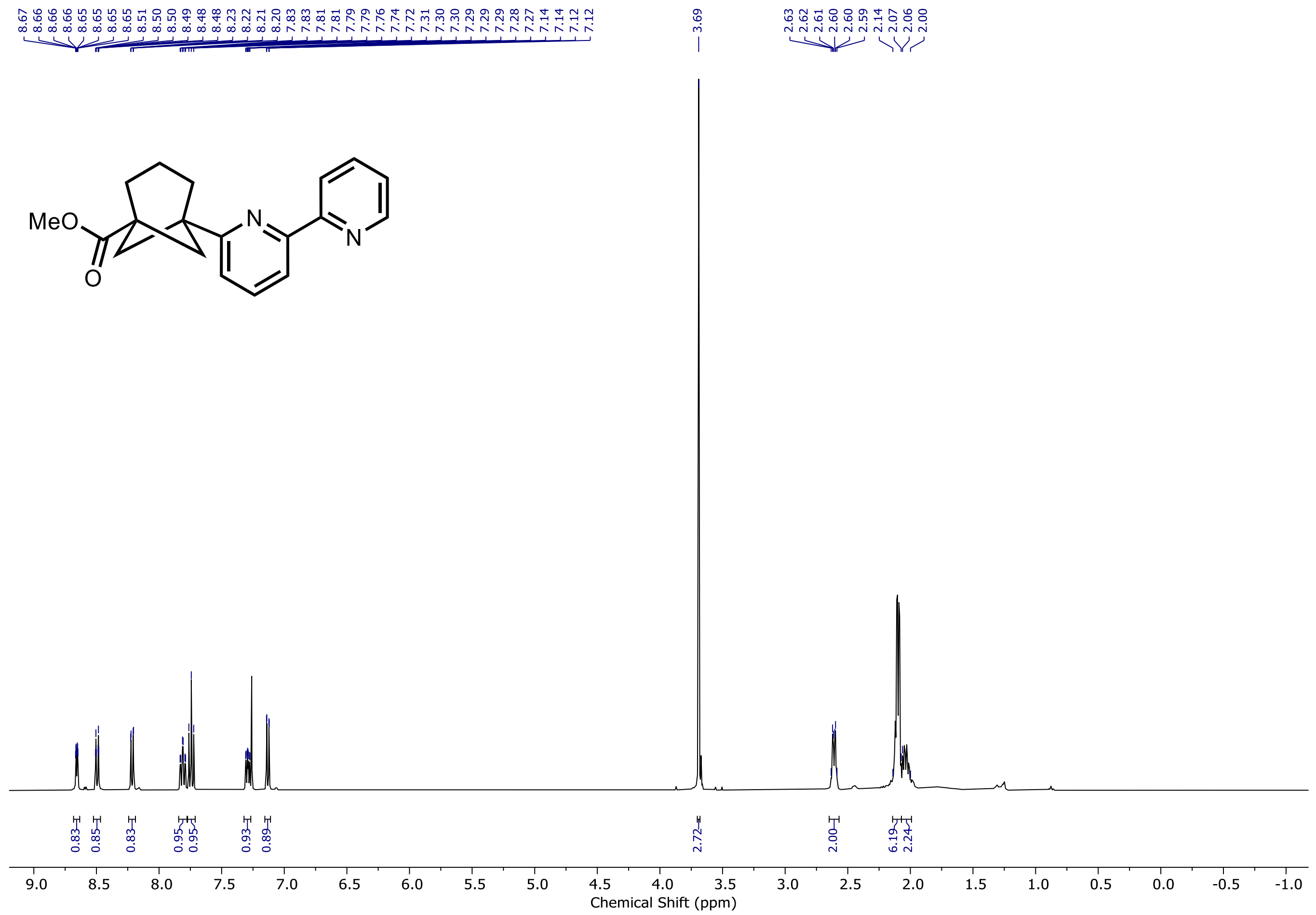

Methyl 5-([2,2'-bipyridin]-6-yl)bicyclo[3.1.1]heptane-1-carboxylate **(3g)** **^{13}C NMR (101 MHz, CDCl$_3$)**

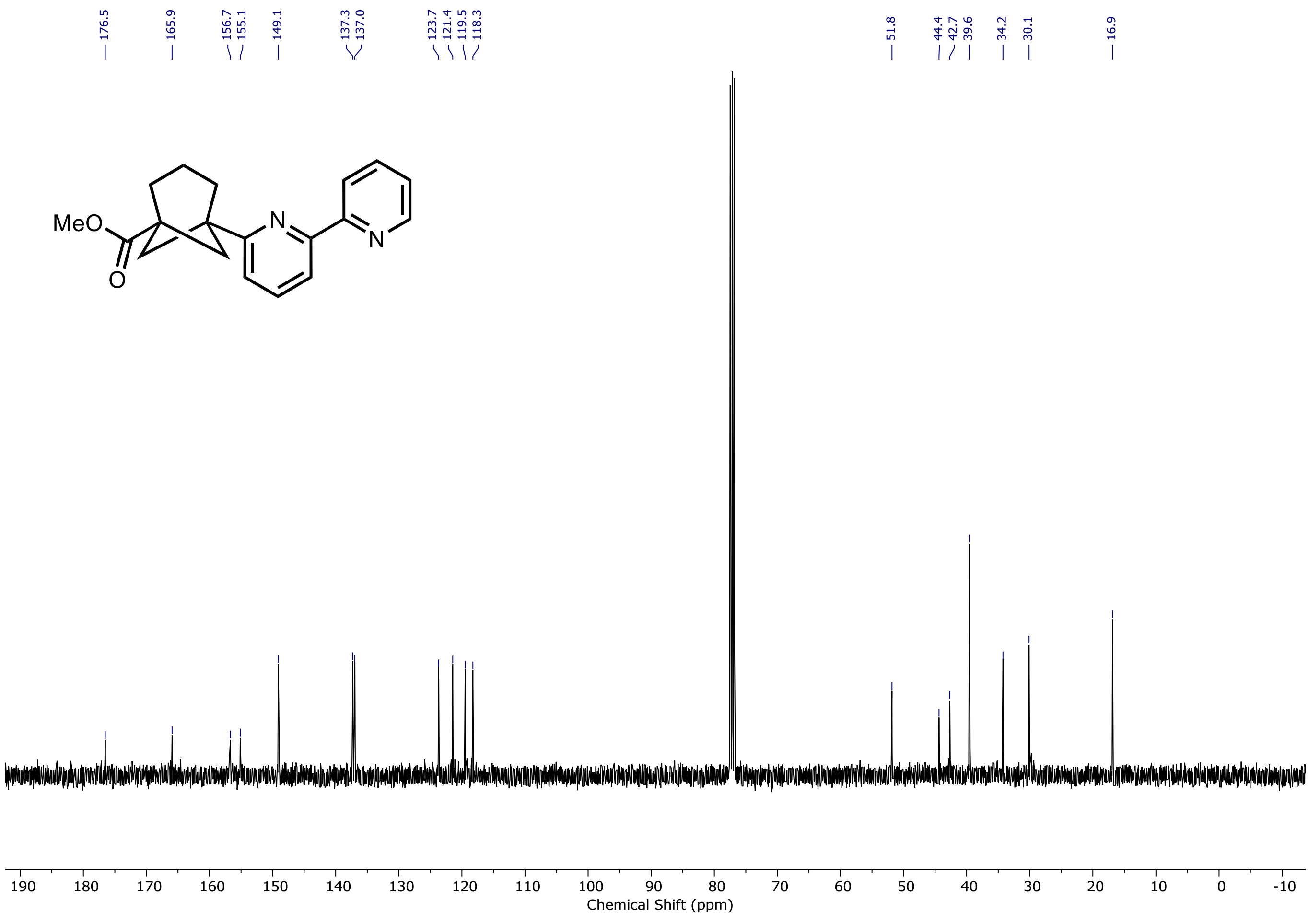

Methyl 5-([2,2'-bipyridin]-4-yl)bicyclo[3.1.1]heptane-1-carboxylate (**3g'**) **^{1}H NMR (400 MHz, CDCl$_3$)**

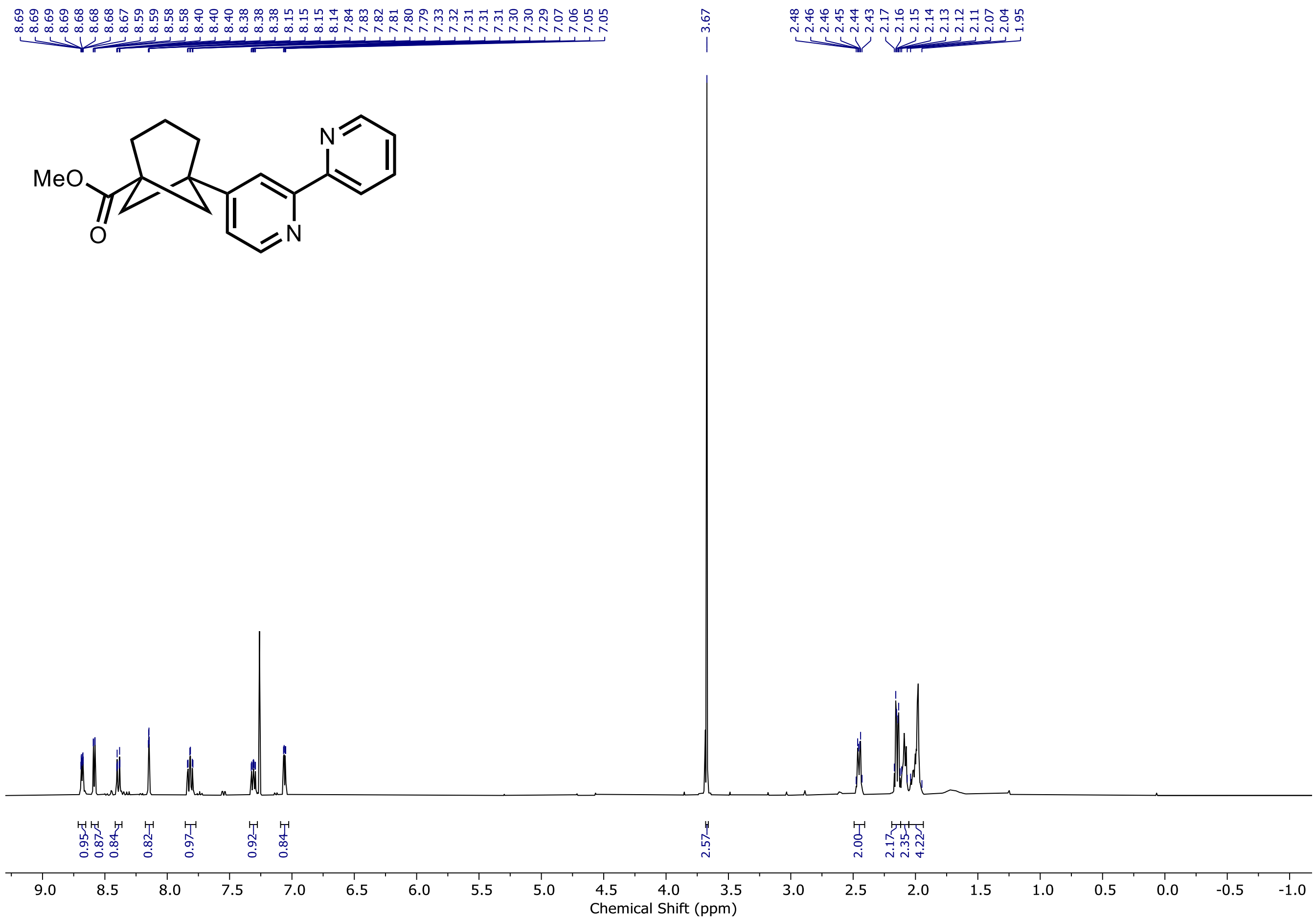

Methyl 5-([2,2'-bipyridin]-4-yl)bicyclo[3.1.1]heptane-1-carboxylate (**3g'**) **^{13}C NMR (151 MHz, CDCl$_3$)**

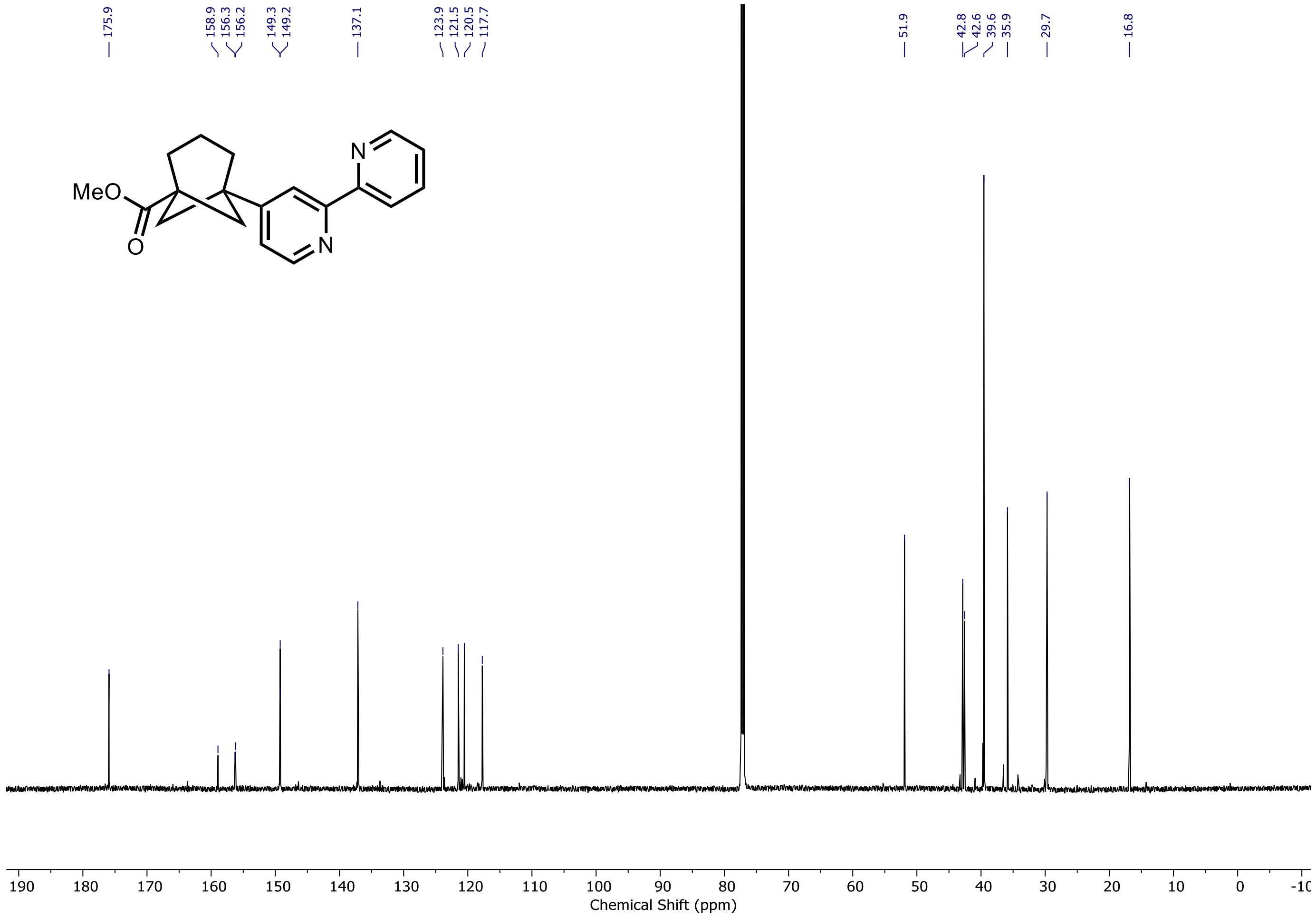

Dimethyl 3-(5-(methoxycarbonyl)bicyclo[3.1.1]heptan-1-yl)pyridine-2,6-dicarboxylate (**3h**) ^{1}**H NMR (400 MHz, CDCl$_3$)**

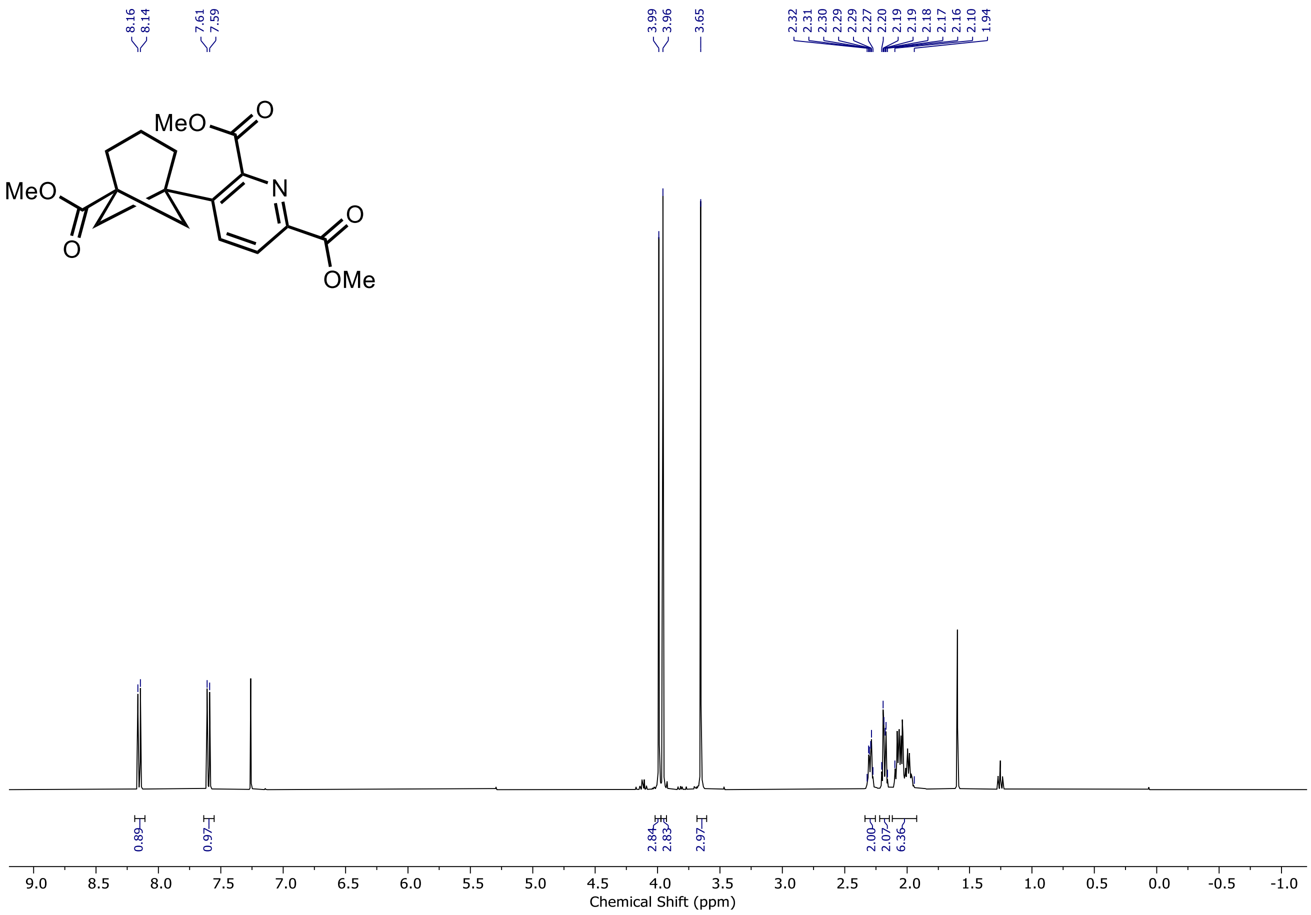

Dimethyl 3-(5-(methoxycarbonyl)bicyclo[3.1.1]heptan-1-yl)pyridine-2,6-dicarboxylate (**3h**) ^{13}**C NMR (151 MHz, CDCl$_3$)**

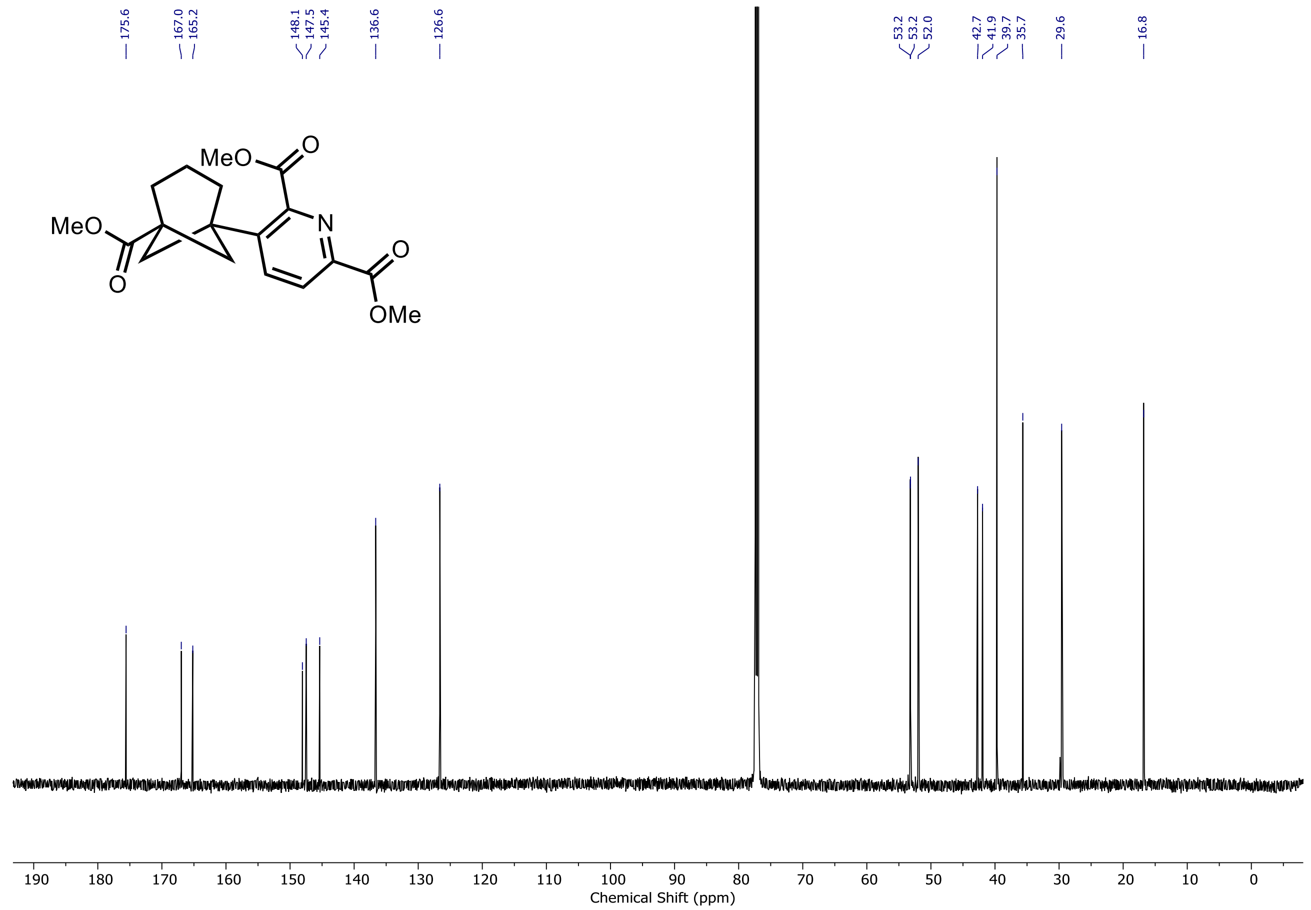

2-(5-(4-Fluorophenyl)bicyclo[3.1.1]heptan-1-yl)-4-methylquinoline (**3i**) ^{1}**H NMR (400 MHz, CDCl$_3$)**

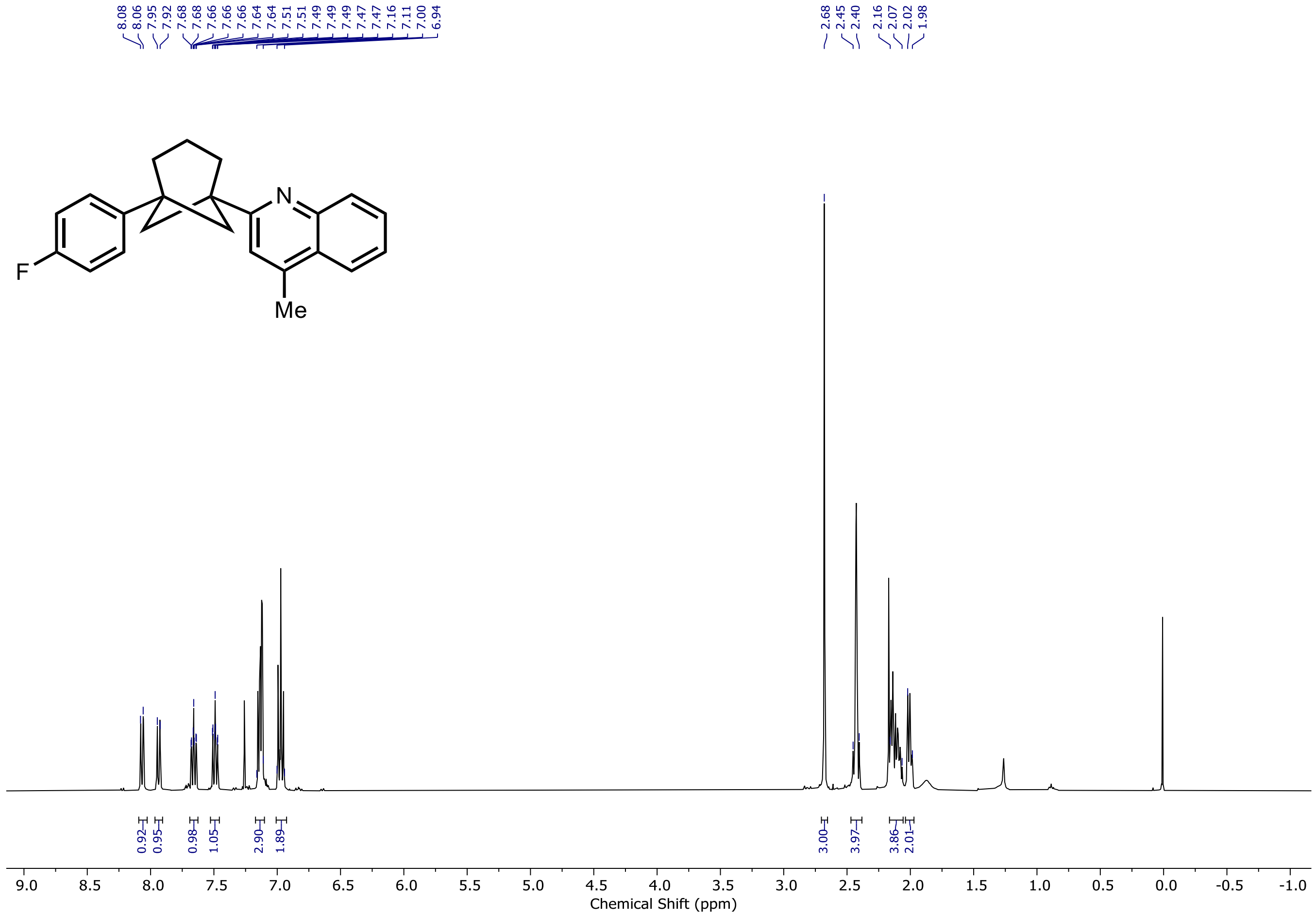

2-(5-(4-Fluorophenyl)bicyclo[3.1.1]heptan-1-yl)-4-methylquinoline (**3i**) **^{13}C NMR (101 MHz, CDCl$_3$)**

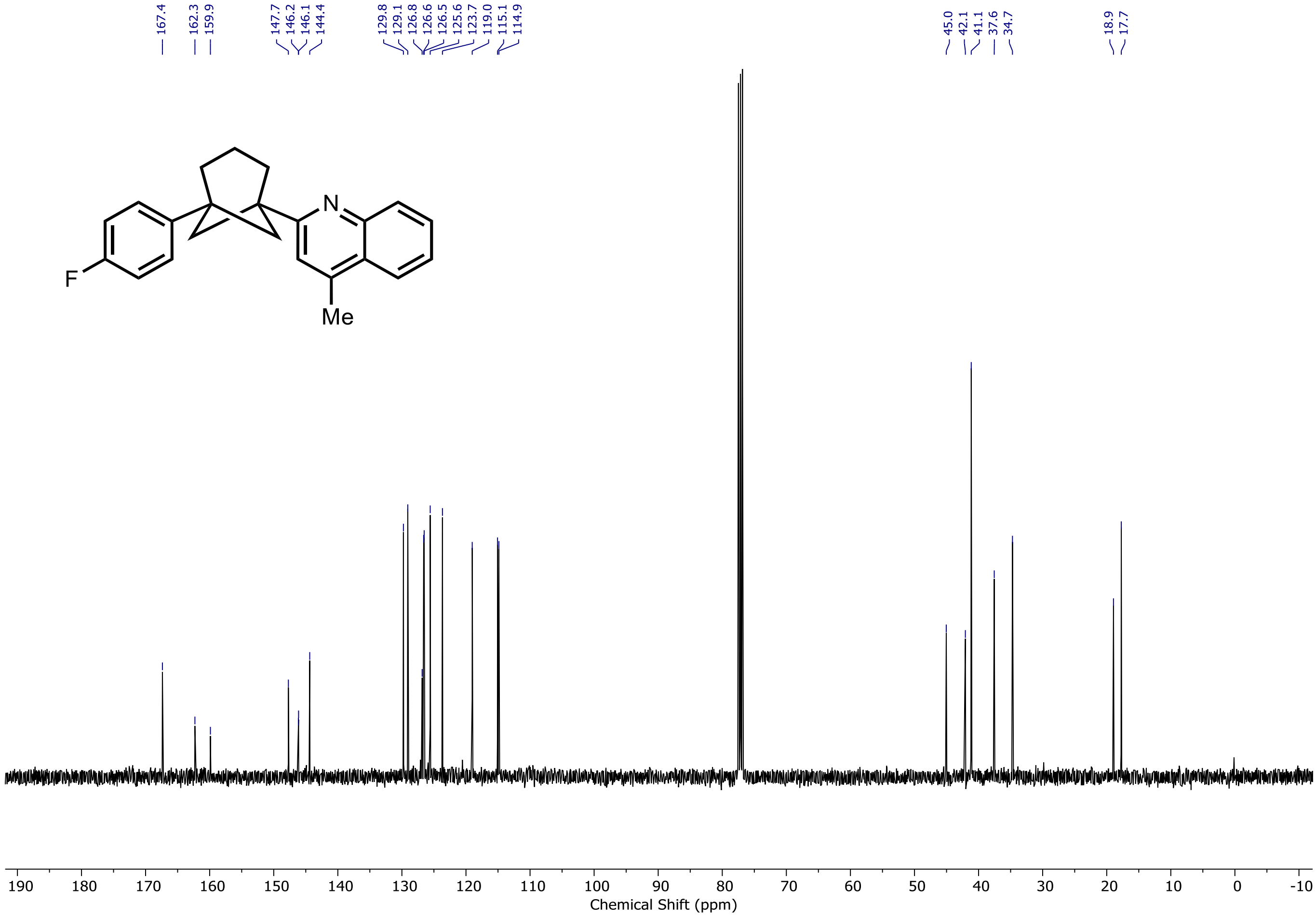

2-(5-(4-Fluorophenyl)bicyclo[3.1.1]heptan-1-yl)-4-methylquinoline (**3i**) **^{19}F NMR (377 MHz, CDCl$_3$)**

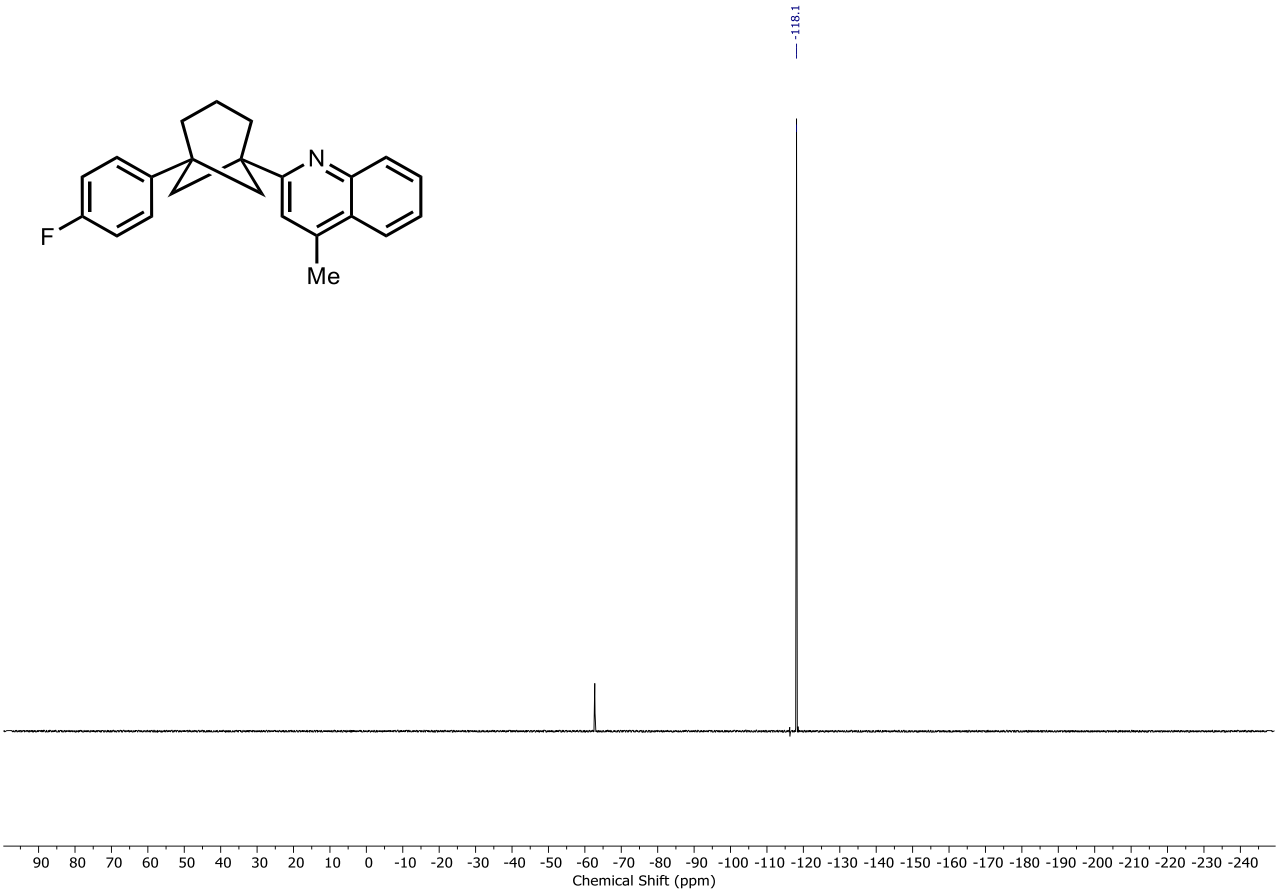
-118.1
F
N
Me
90 80 70 60 50 40 30 20 10 0 -10 -20 -30 -40 -50 -60 -70 -80 -90 -100 -110 -120 -130 -140 -150 -160 -170 -180 -190 -200 -210 -220 -230 -240
Chemical Shift (ppm)

4-Methyl-2-(5-(p-tolyl)bicyclo[3.1.1]heptan-1-yl)quinoline (**3j**) **^{1}H NMR (400 MHz, $CDCl_3$)**

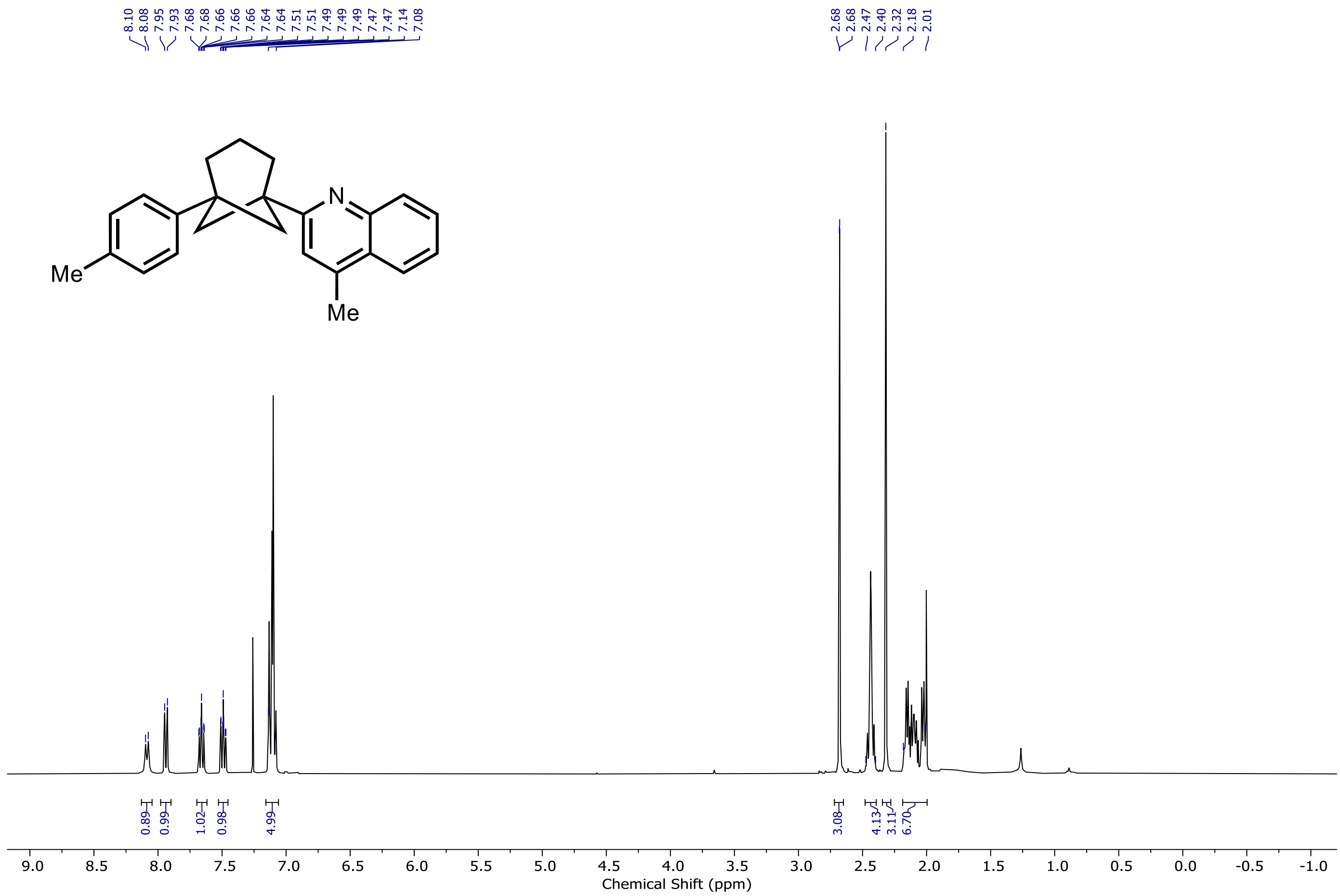

4-Methyl-2-(5-(p-tolyl)bicyclo[3.1.1]heptan-1-yl)quinoline (**3j**) **^{13}C NMR (101 MHz, $CDCl_3$)**

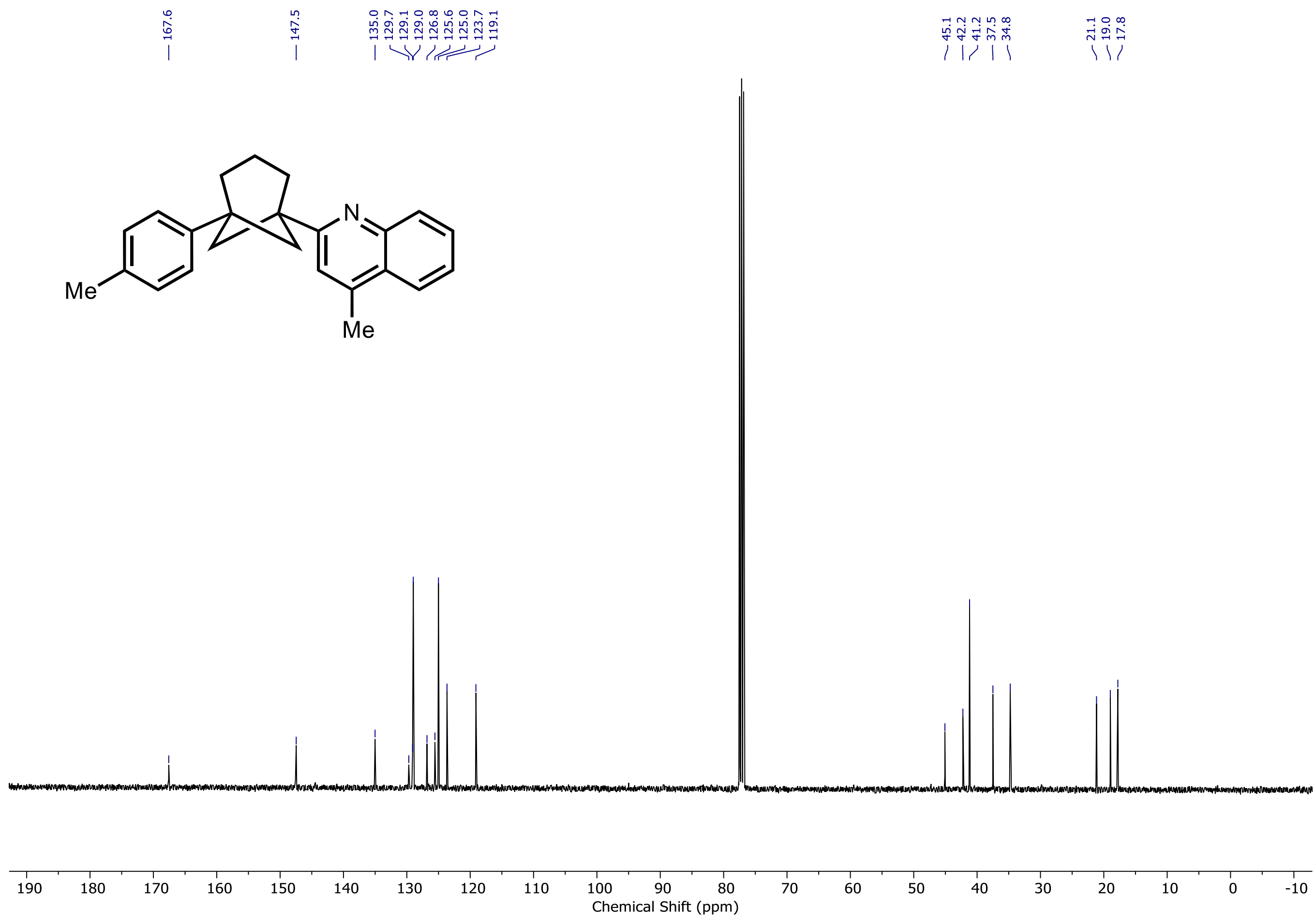

4-Methyl-2-(5-(4-(trifluoromethyl)benzyl)bicyclo[3.1.1]heptan-1-yl)quinoline (**3k**) **^{1}H NMR (400 MHz, $CDCl_3$)**

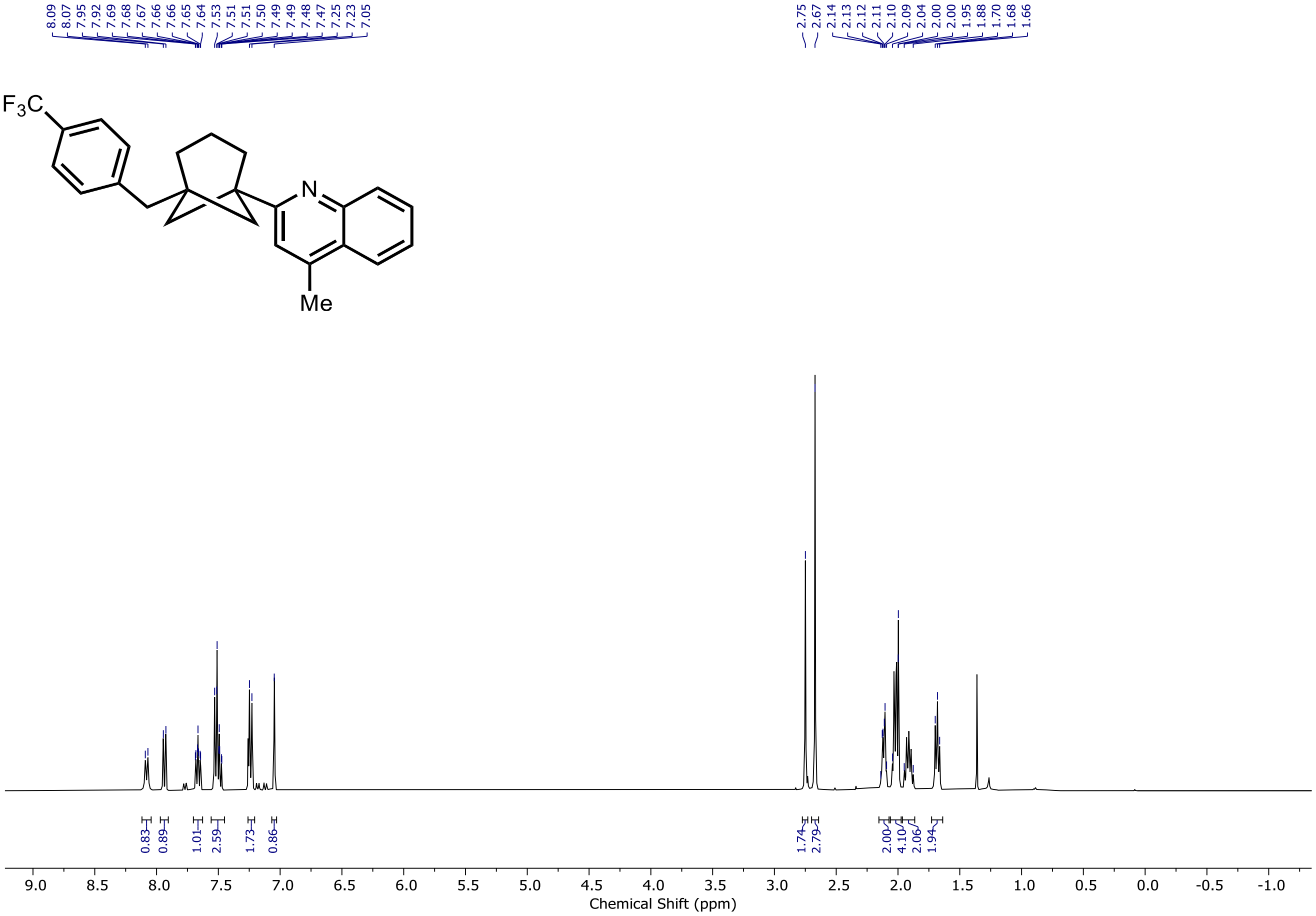

4-Methyl-2-(5-(4-(trifluoromethyl)benzyl)bicyclo[3.1.1]heptan-1-yl)quinoline (**3k**) **^{13}C NMR (101 MHz, $CDCl_3$)**

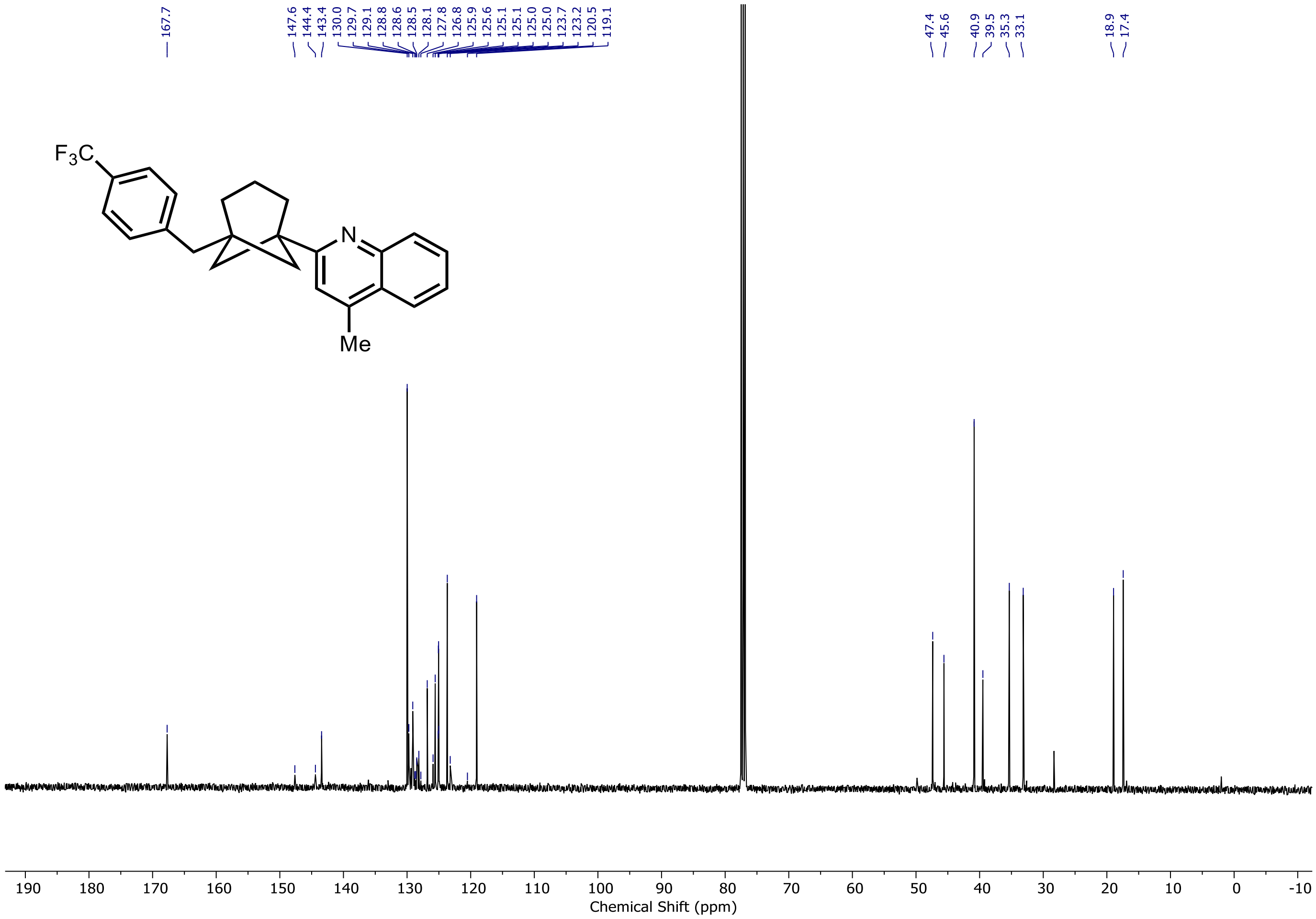

4-Methyl-2-(5-(4-(trifluoromethyl)benzyl)bicyclo[3.1.1]heptan-1-yl)quinoline (**3k**) **^{19}F NMR (377 MHz, $CDCl_3$)**

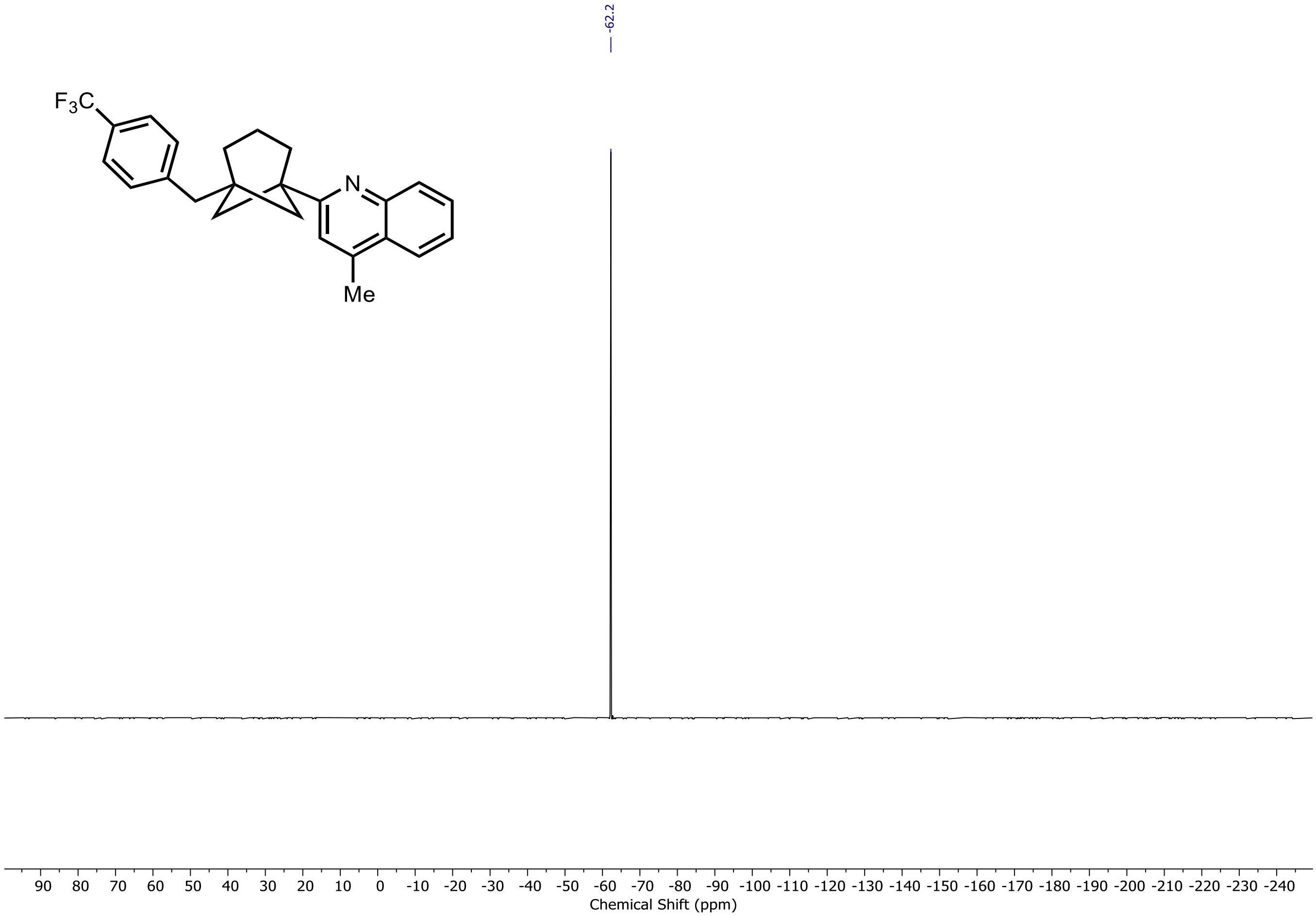

2-(5-(3,5-Bis(trifluoromethyl)benzyl)bicyclo[3.1.1]heptan-1-yl)-4-methylquinoline (**3l**) **^{1}H NMR (400 MHz, $CDCl_3$)**

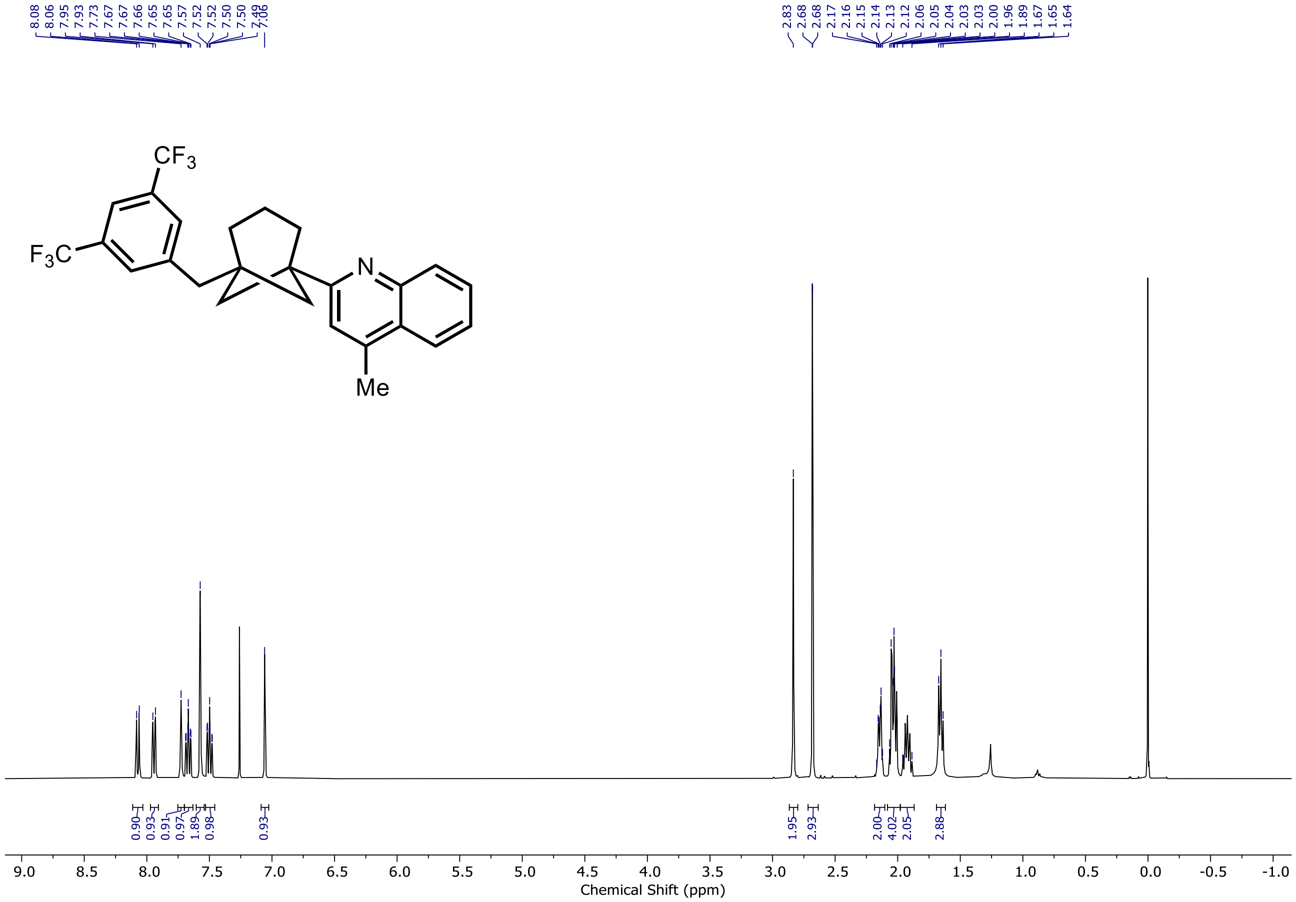

2-(5-(3,5-Bis(trifluoromethyl)benzyl)bicyclo[3.1.1]heptan-1-yl)-4-methylquinoline (**3l**) **^{13}C NMR (101 MHz, $CDCl_3$)**

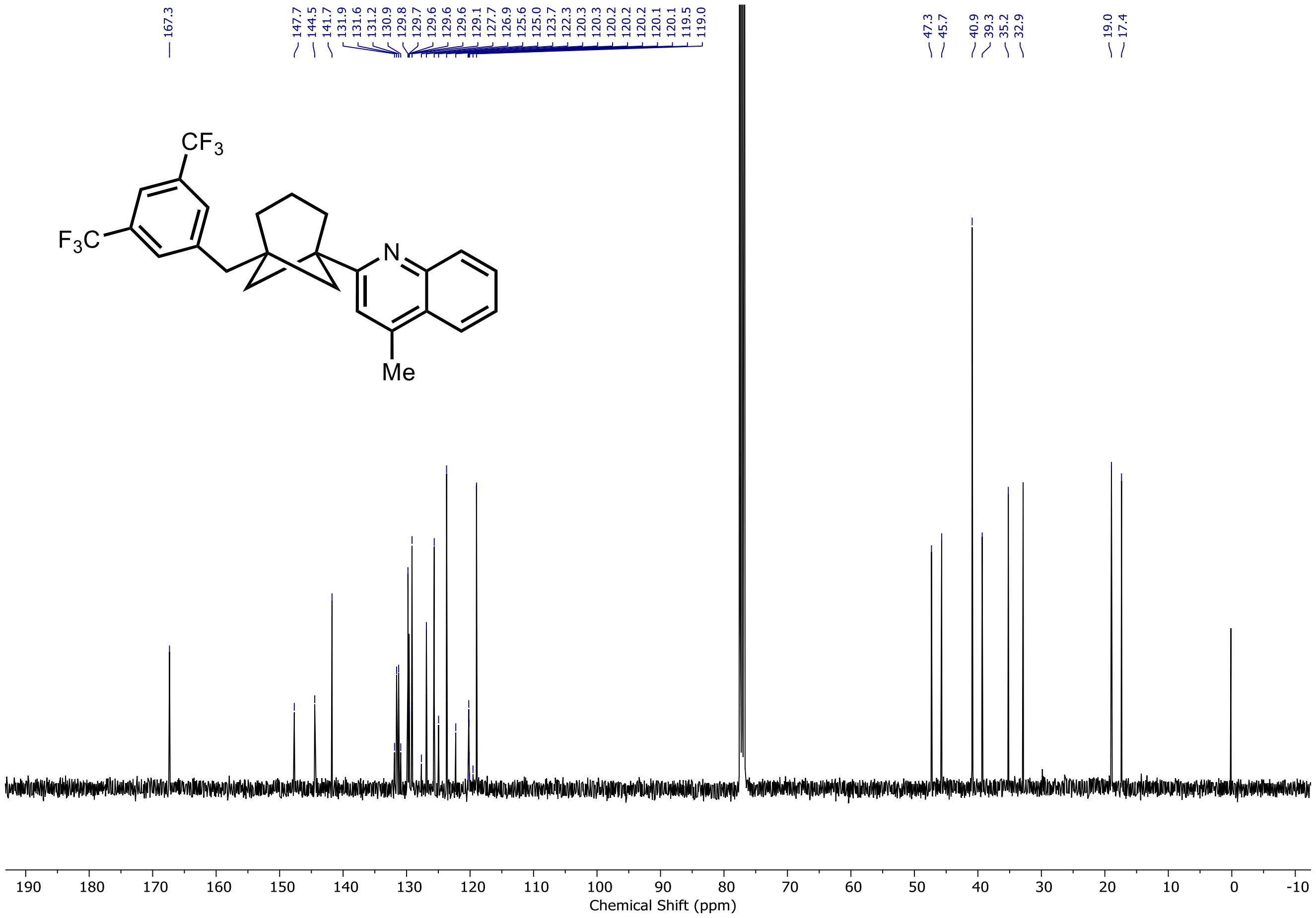

2-(5-(3,5-Bis(trifluoromethyl)benzyl)bicyclo[3.1.1]heptan-1-yl)-4-methylquinoline (**3l**) **^{19}F NMR (377 MHz, $CDCl_3$)**

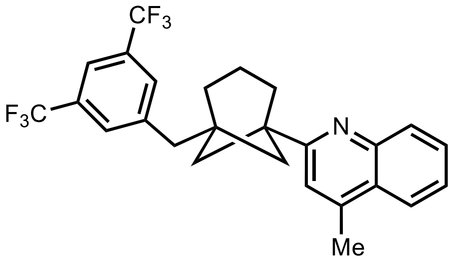

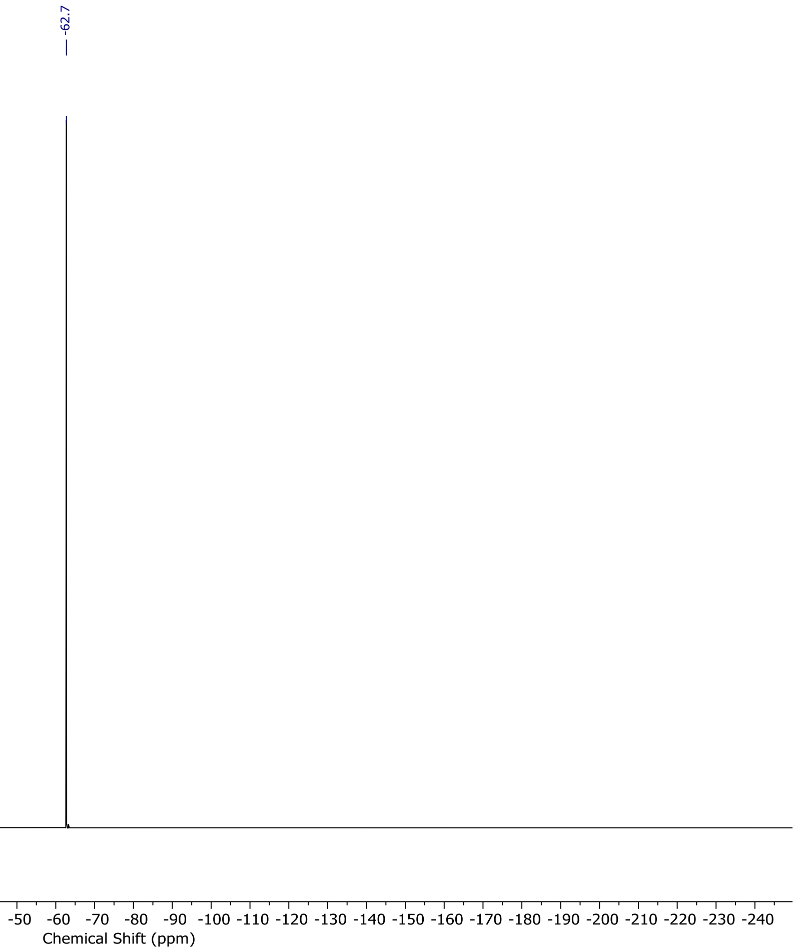
-62.7
90 80 70 60 50 40 30 20 10 0 -10 -20 -30 -40 -50 -60 -70 -80 -90 -100 -110 -120 -130 -140 -150 -160 -170 -180 -190 -200 -210 -220 -230 -240
Chemical Shift (ppm)

4-Methyl-2-(5-(trifluoromethyl)bicyclo[3.1.1]heptan-1-yl)quinoline (**3m**) **^{1}H NMR (400 MHz, CDCl$_3$)**

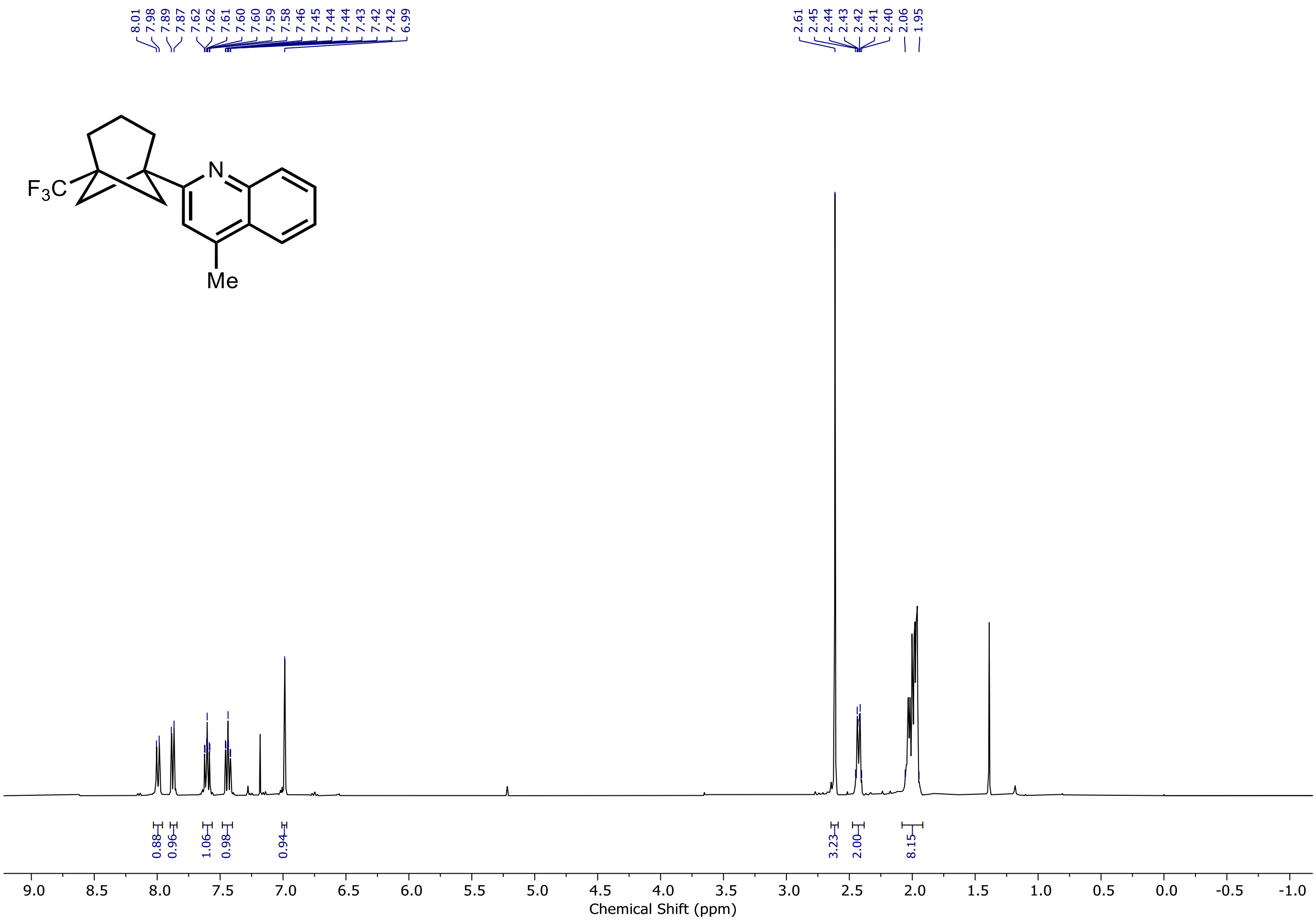

4-Methyl-2-(5-(trifluoromethyl)bicyclo[3.1.1]heptan-1-yl)quinoline (**3m**) **^{13}C NMR (126 MHz, CDCl$_3$)**

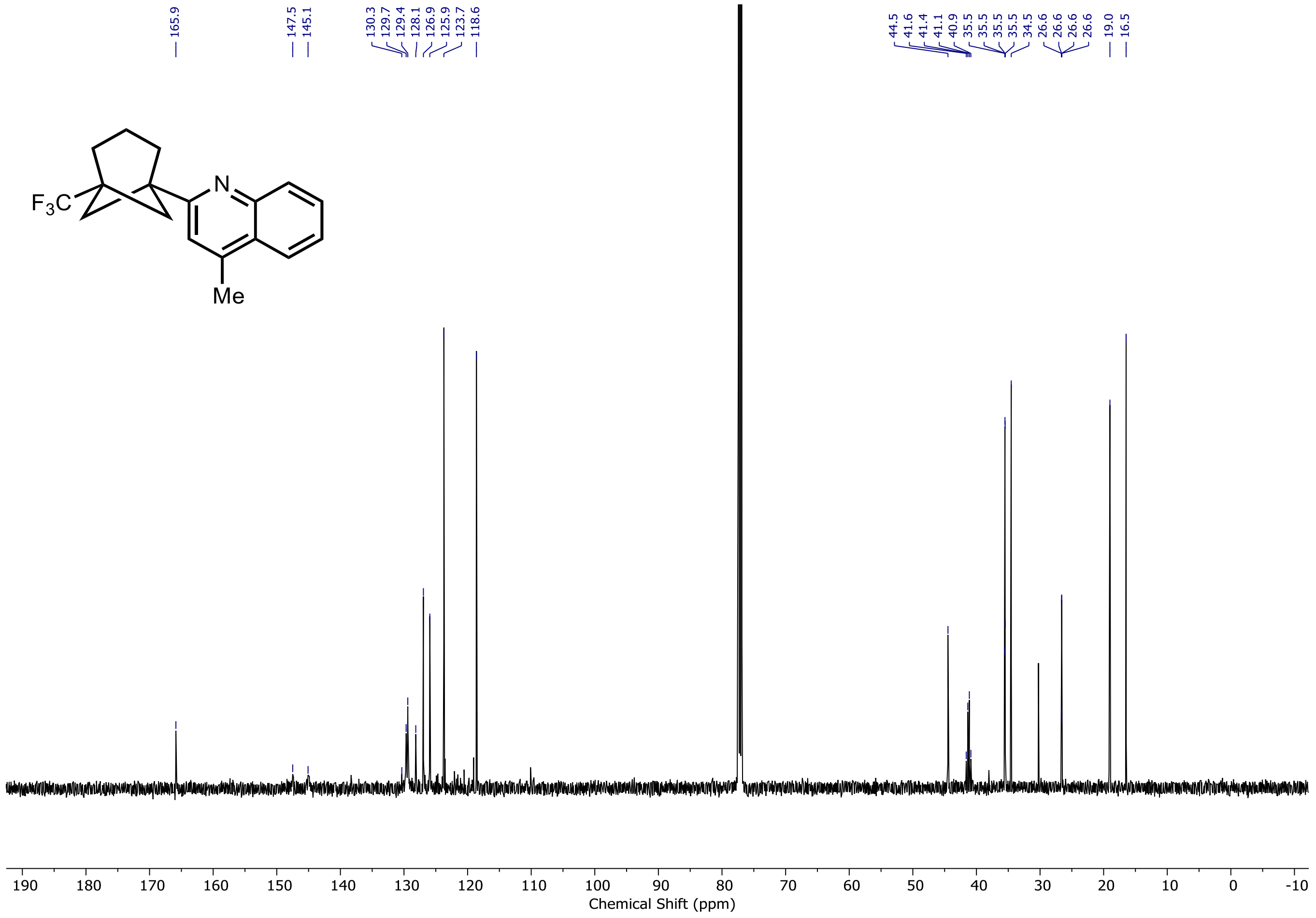

4-Methyl-2-(5-(trifluoromethyl)bicyclo[3.1.1]heptan-1-yl)quinoline (**3m**) ^{19}F **NMR (377 MHz, CDCl$_3$)**

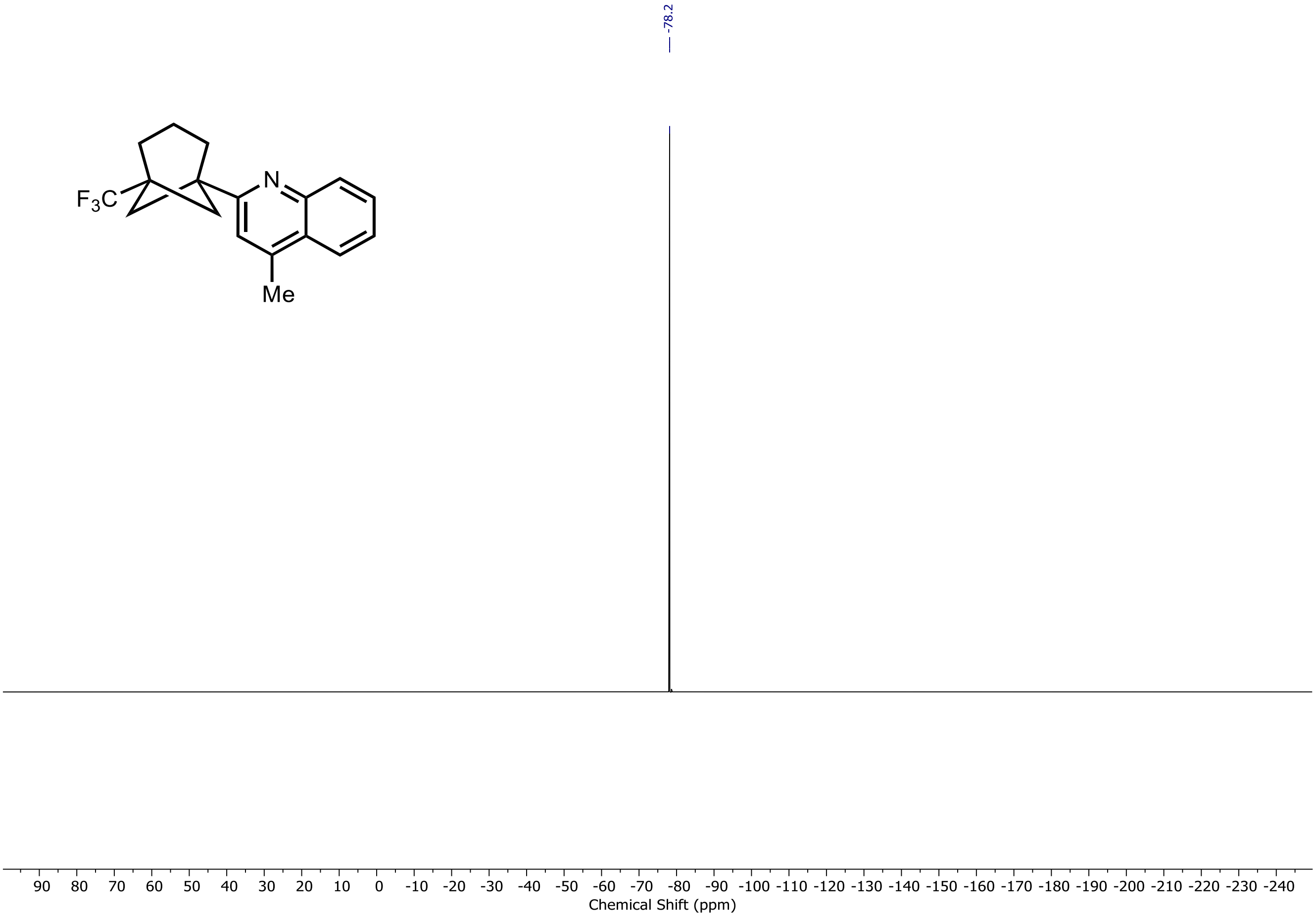

(4-(6-Fluorobenzo[d]isoxazol-3-yl)piperidin-1-yl)(5-(4-methylquinolin-2-yl)bicyclo[3.1.1]heptan-1-yl)methanone **(3n)** ^{1}H **NMR (400 MHz, CDCl$_3$)**

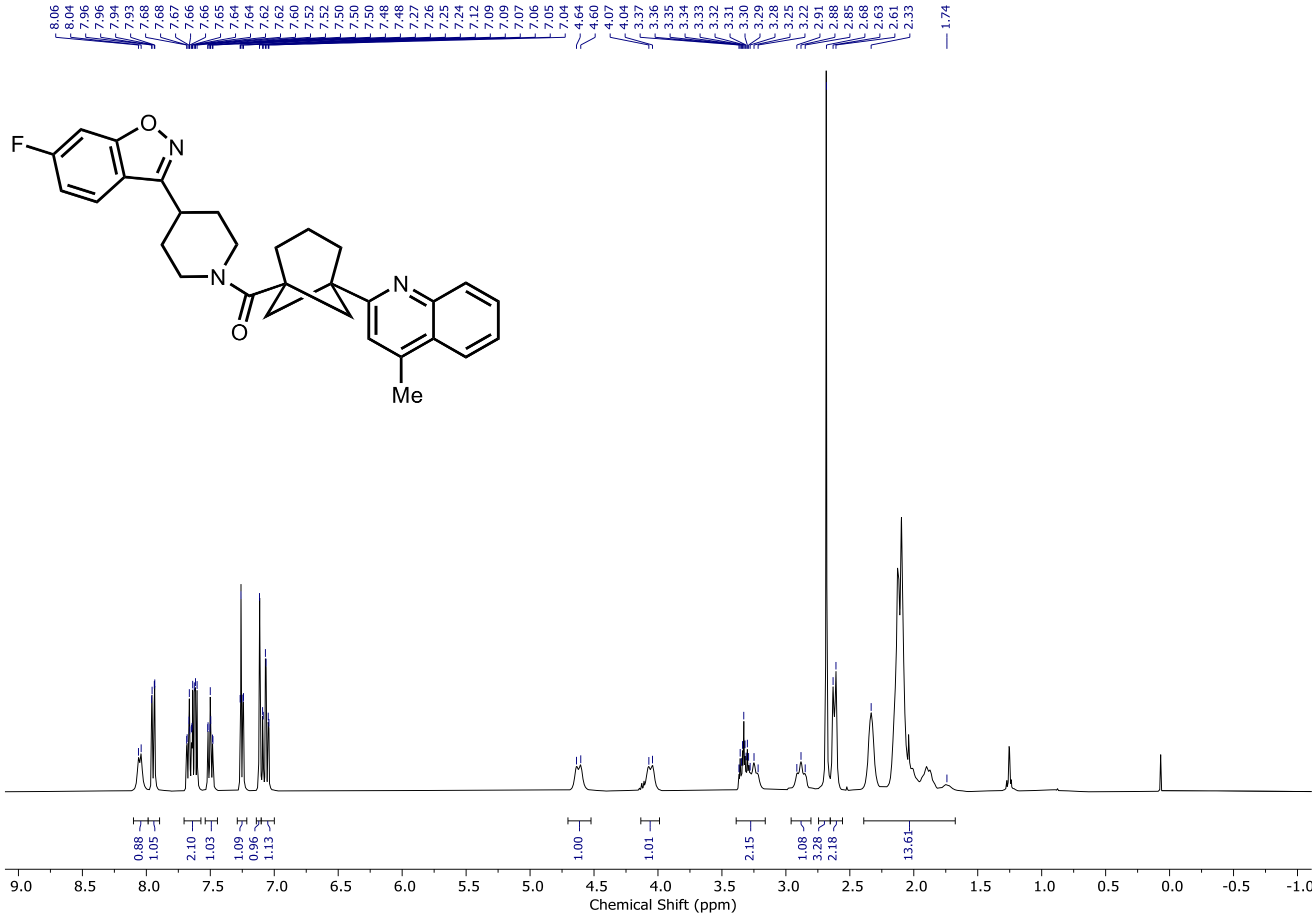

(4-(6-Fluorobenzo[d]isoxazol-3-yl)piperidin-1-yl)(5-(4-methylquinolin-2-yl)bicyclo[3.1.1]heptan-1-yl)methanone **(3n)** ^{13}C **NMR (101 MHz, CDCl$_3$)**

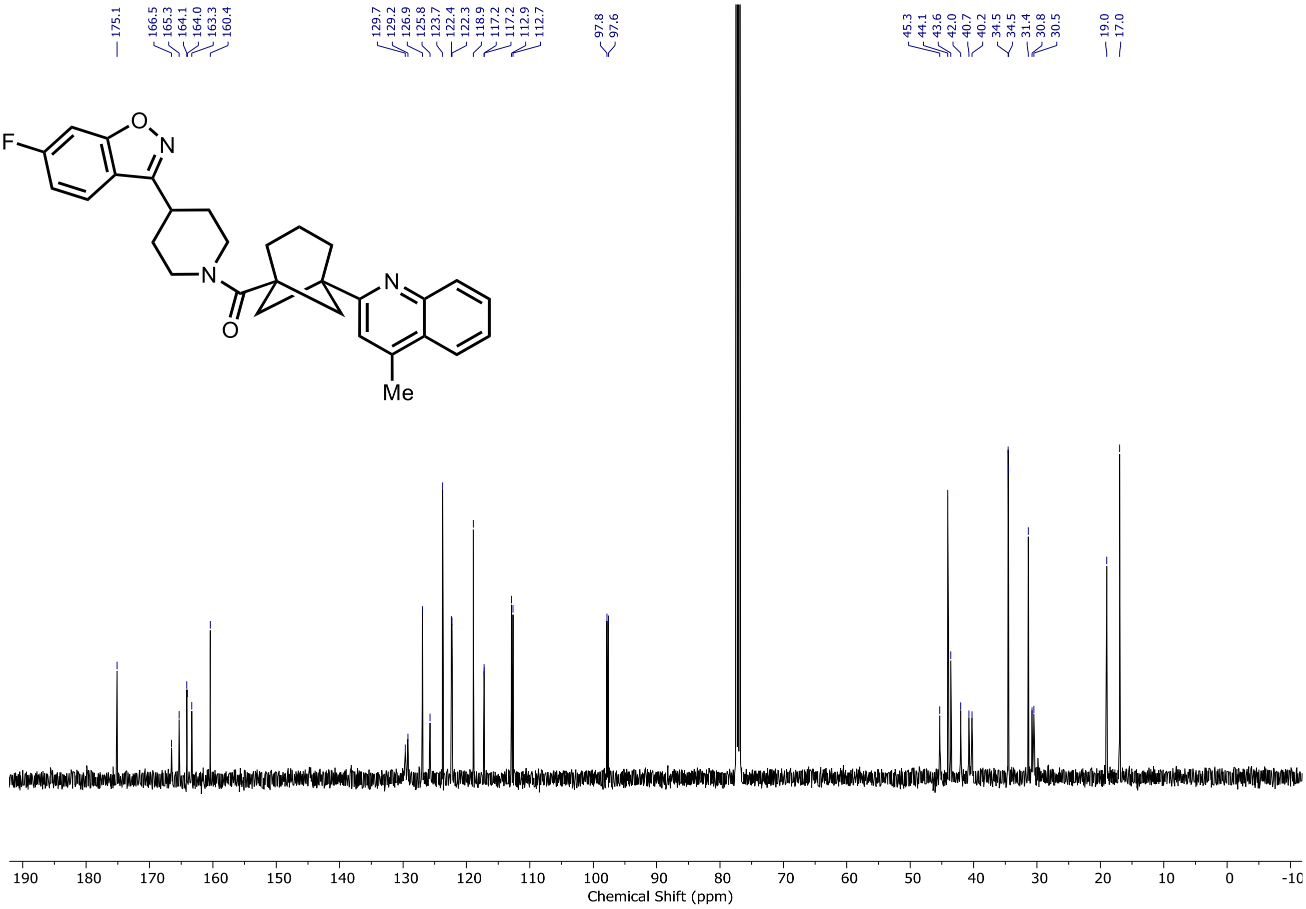

(4-(6-Fluorobenzo[d]isoxazol-3-yl)piperidin-1-yl)(5-(4-methylquinolin-2-yl)bicyclo[3.1.1]heptan-1-yl)methanone **(3n)** **^{19}F NMR (377 MHz, CDCl$_3$)**

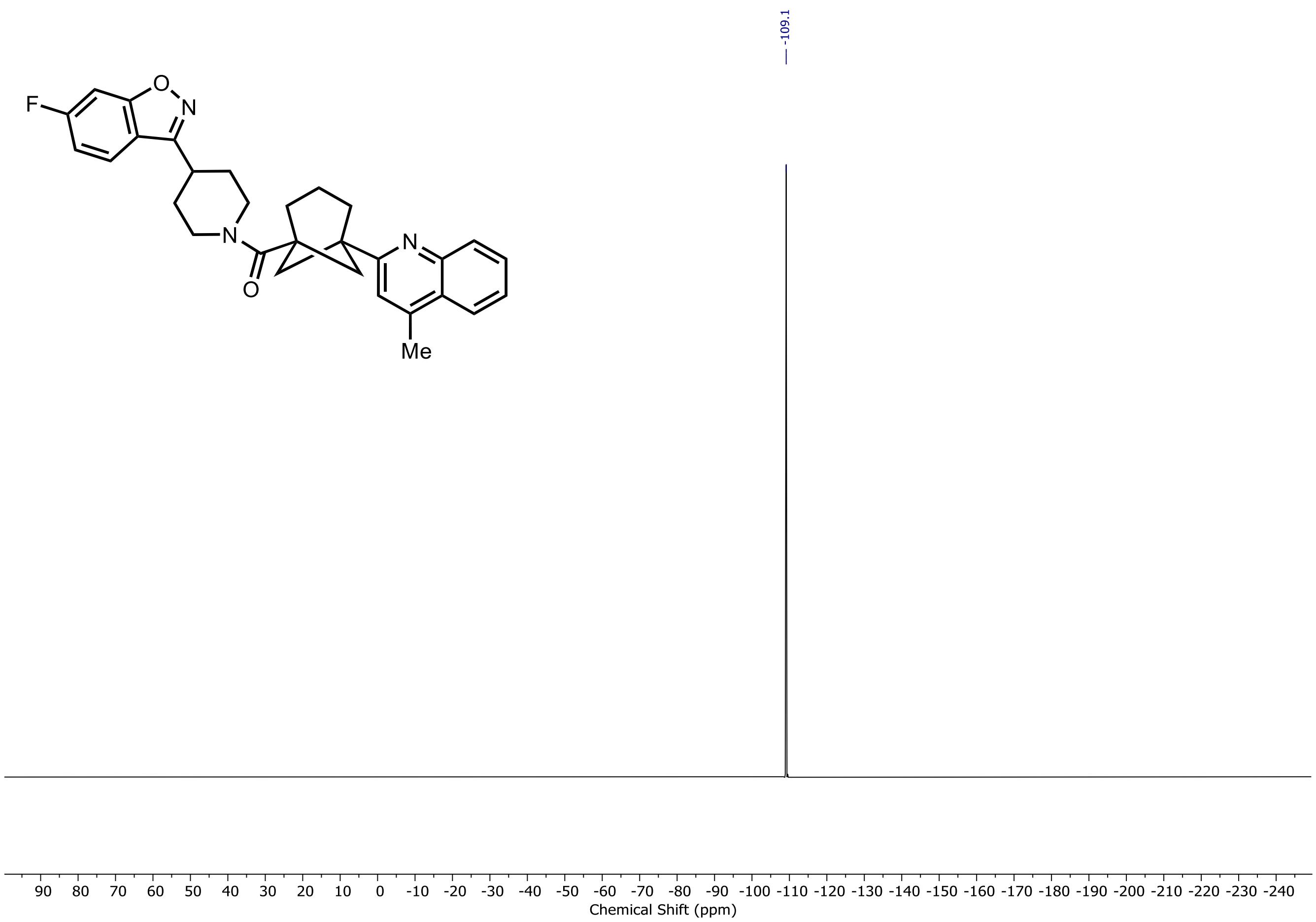

tert-Butyl 1-(4-methylquinolin-2-yl)-3-azabicyclo[3.1.1]heptane-3-carboxylate **(7a)** ^{1}**H NMR (400 MHz, DMSO-d6, 90°C)**

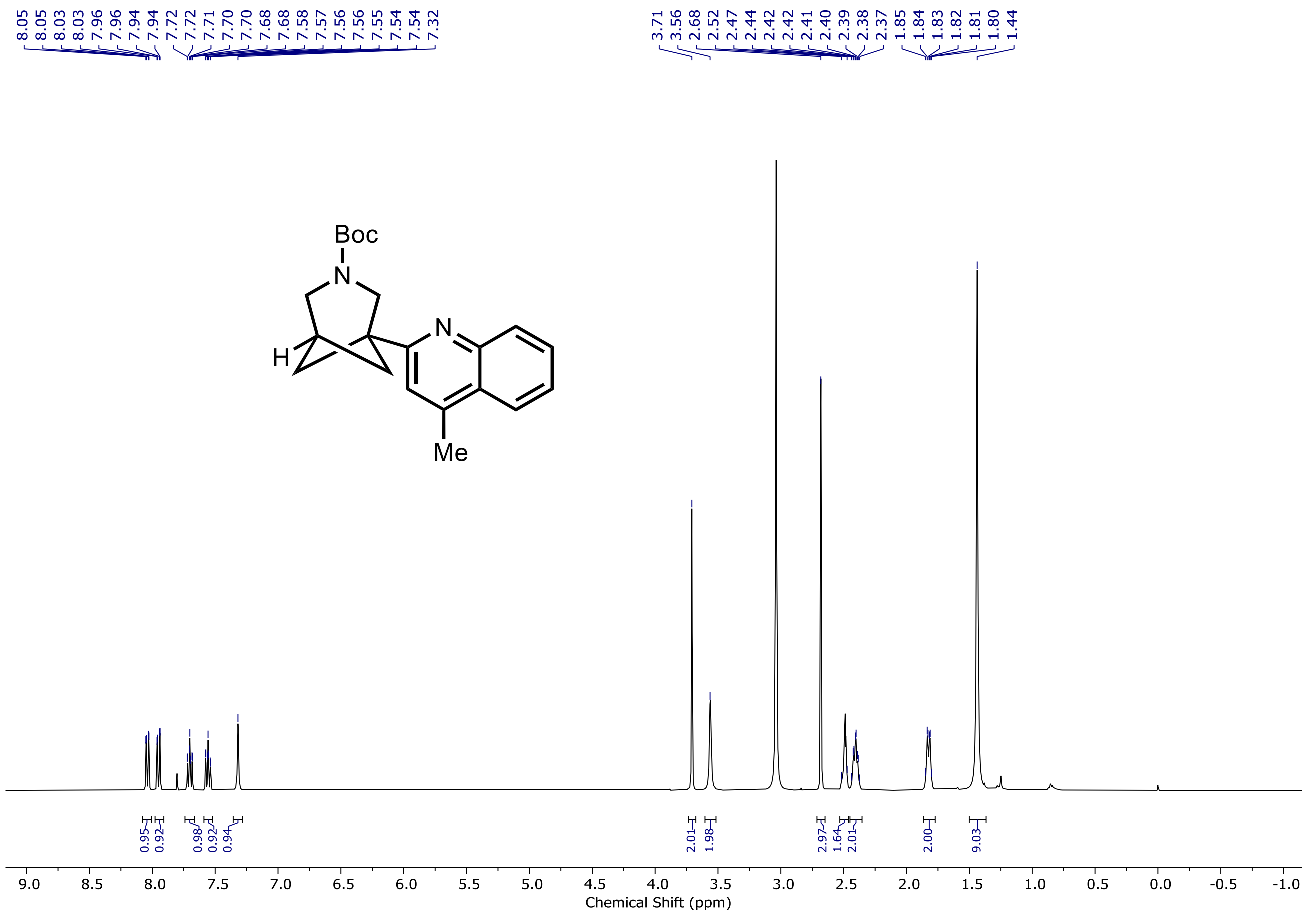

tert-Butyl 1-(4-methylquinolin-2-yl)-3-azabicyclo[3.1.1]heptane-3-carboxylate **(7a)** ^{13}**C NMR (101 MHz, $CDCl_3$)**

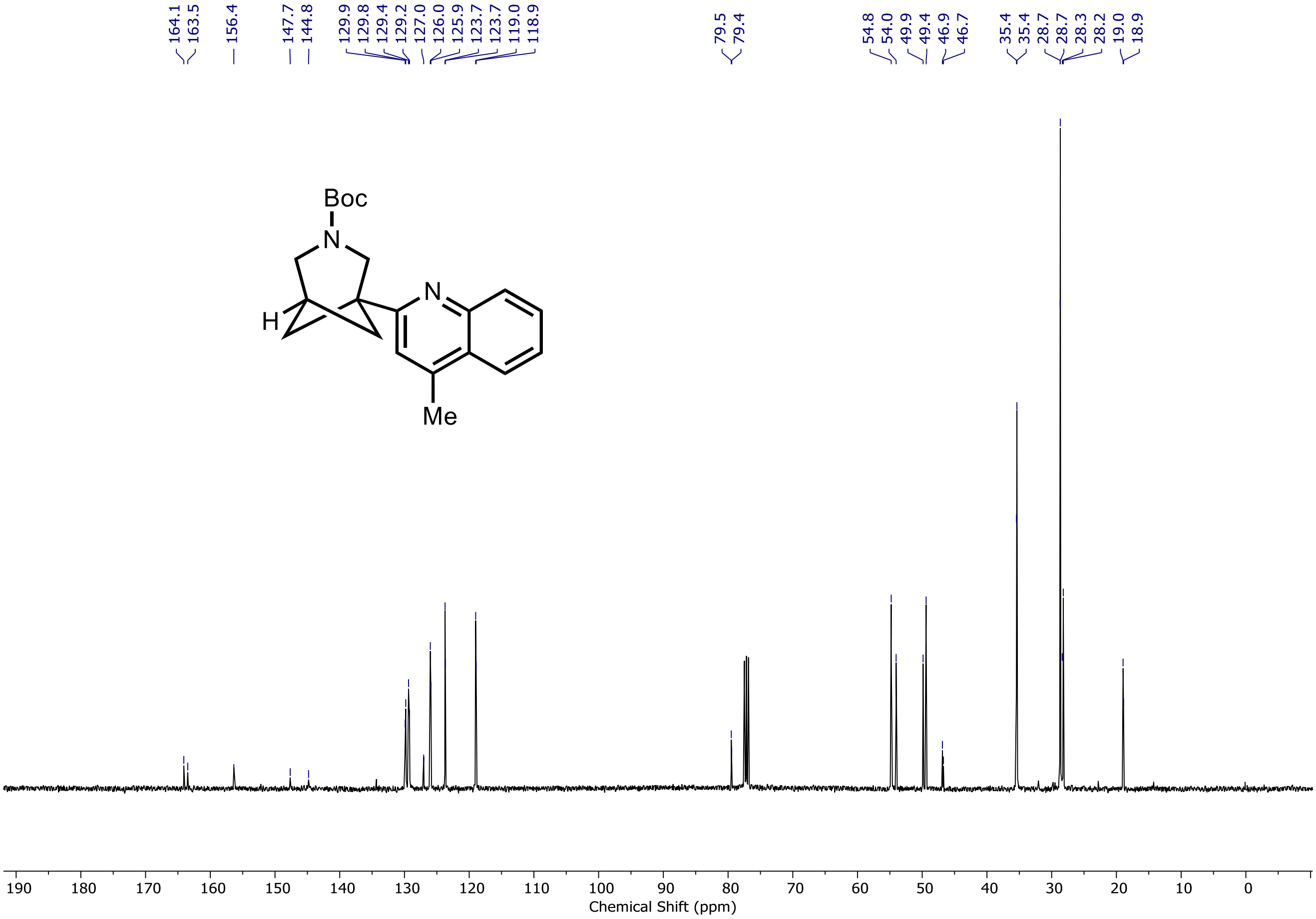

tert-Butyl 1-(4-methylpyridin-2-yl)-3-azabicyclo[3.1.1]heptane-3-carboxylate **(7b)** ^{1}**H NMR (400 MHz, DMSO-d6, 90°C)**

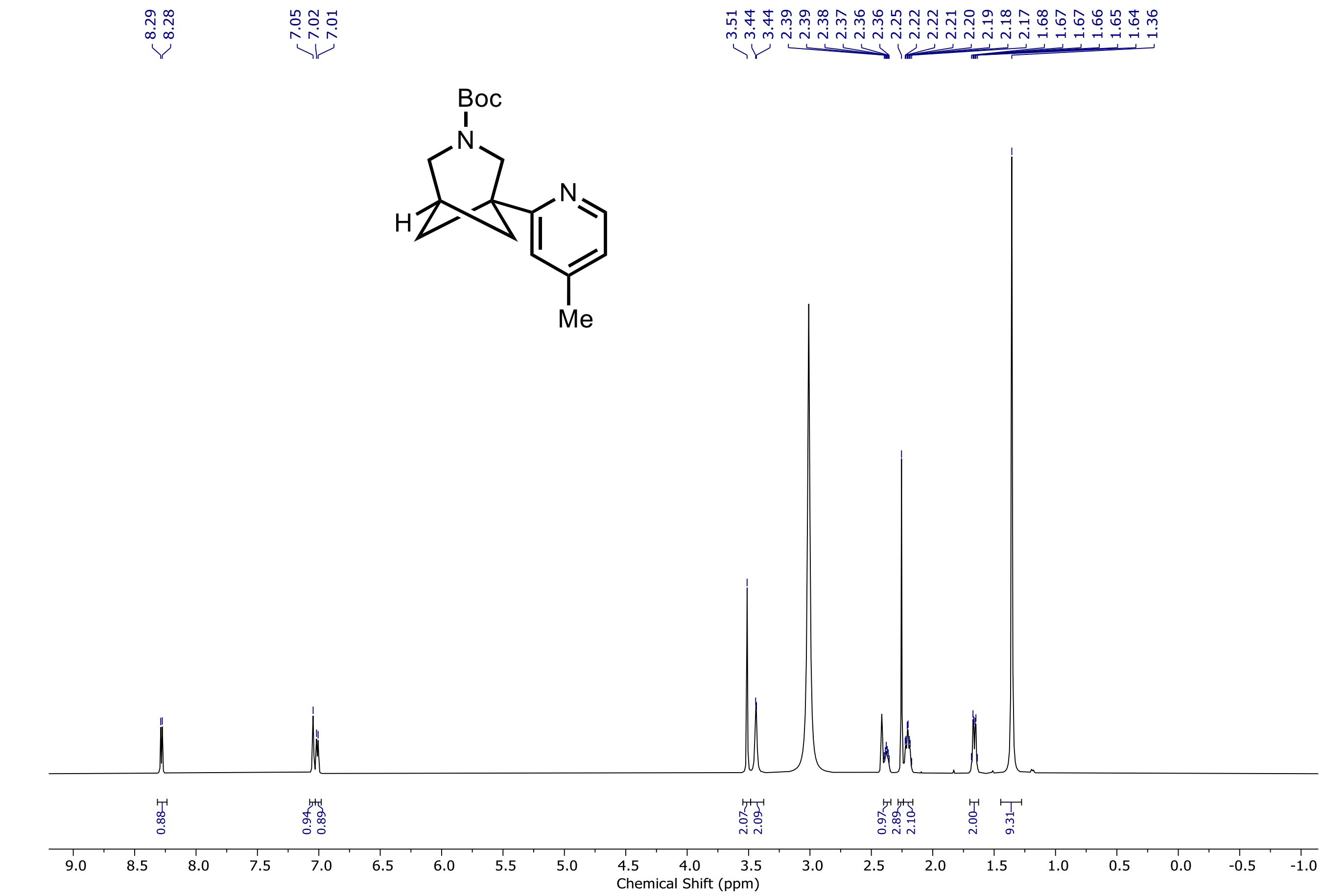

tert-Butyl 1-(4-methylpyridin-2-yl)-3-azabicyclo[3.1.1]heptane-3-carboxylate (**7b**) **^{13}C NMR (101 MHz, CDCl$_3$)**

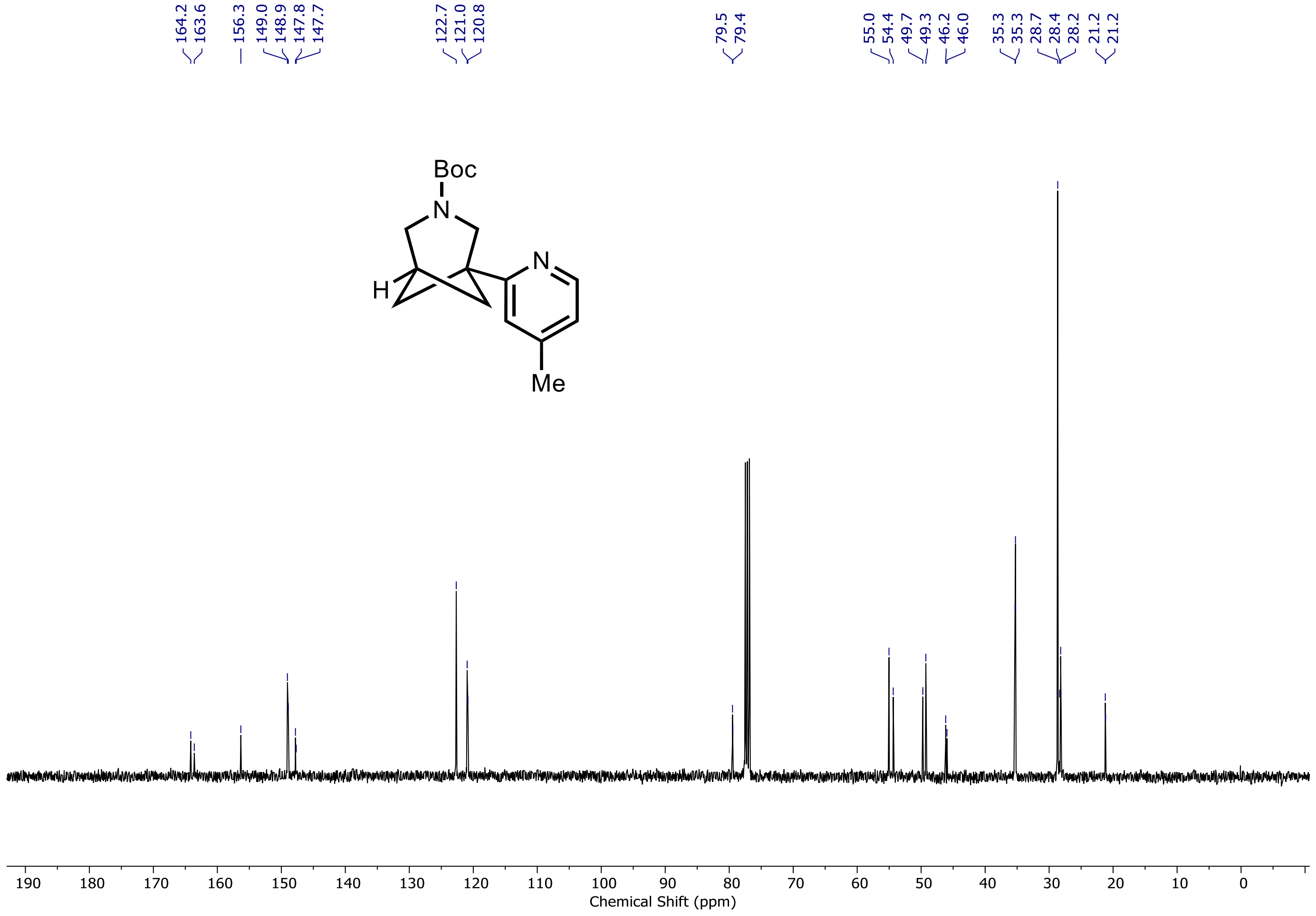

tert-Butyl 1-(phthalazin-1-yl)-3-azabicyclo[3.1.1]heptane-3-carboxylate (**7c**) **^{1}H NMR (400 MHz, CDCl$_3$)**

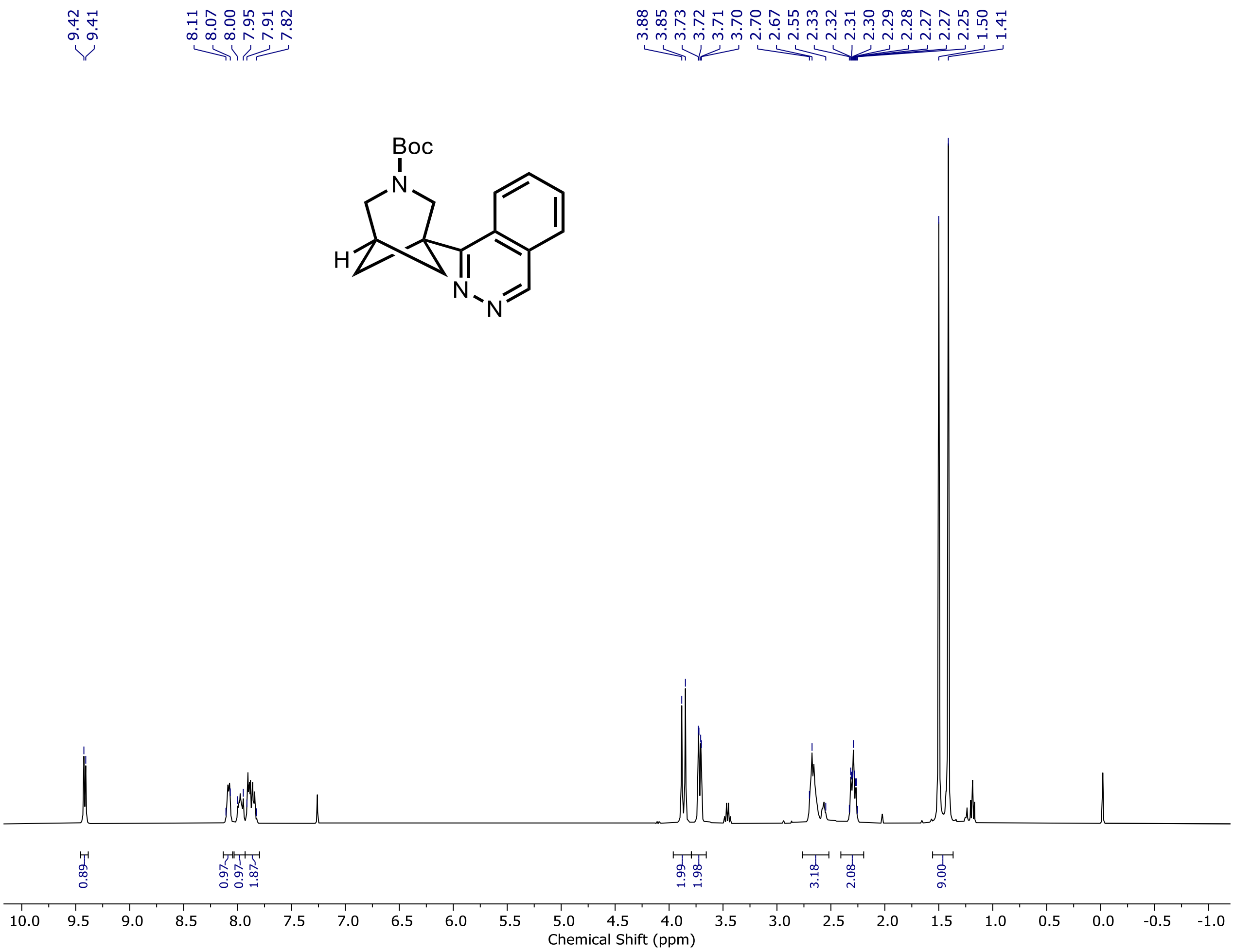

tert-Butyl 1-(phthalazin-1-yl)-3-azabicyclo[3.1.1]heptane-3-carboxylate **(7c)** **^{13}C NMR (101 MHz, $CDCl_3$)**

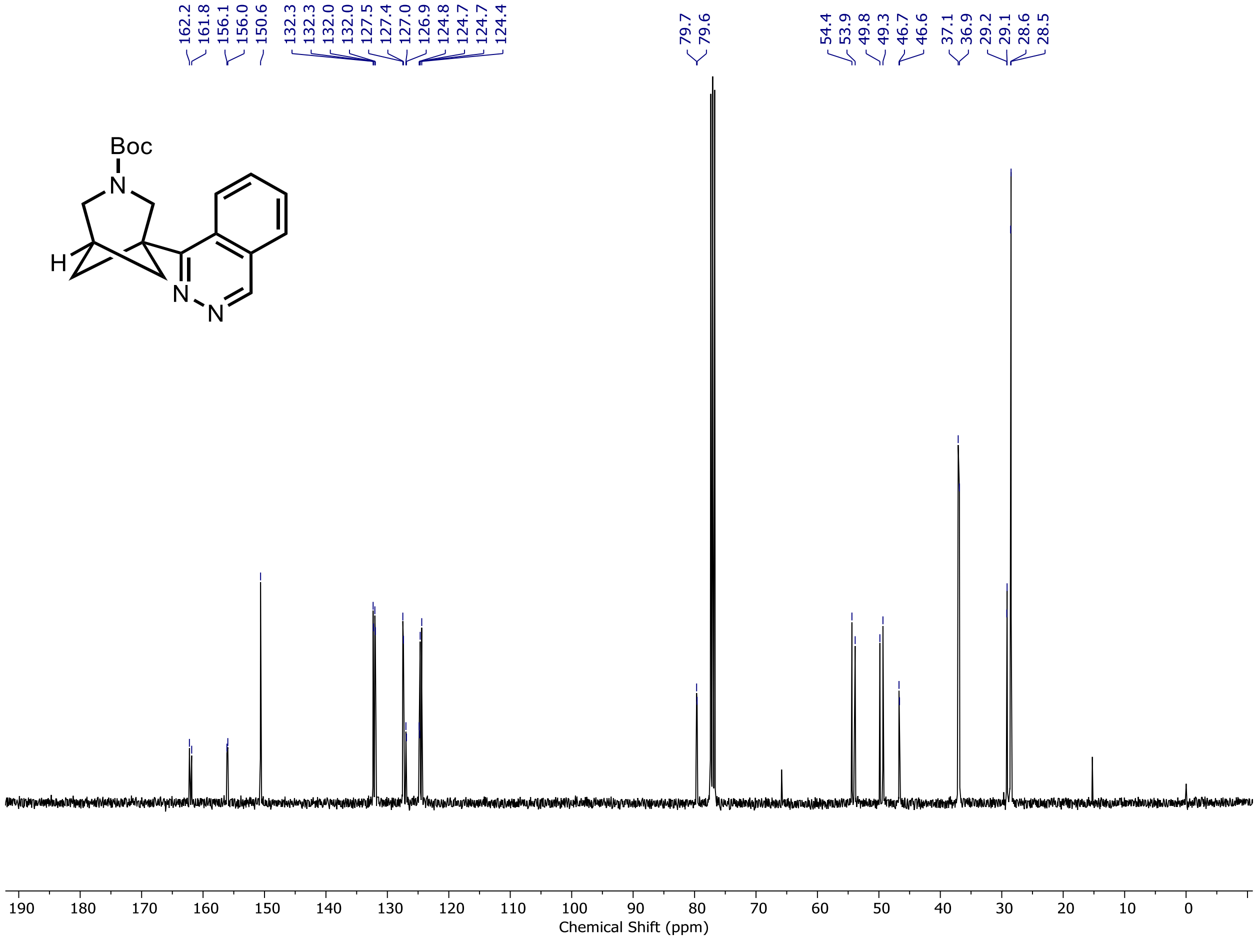

3-(*tert*-Butyl) 1-methyl 5-(4-methylquinolin-2-yl)-3-azabicyclo[3.1.1]heptane-1,3-dicarboxylate **(7d)** **^{1}H NMR (400 MHz, $CDCl_3$)**

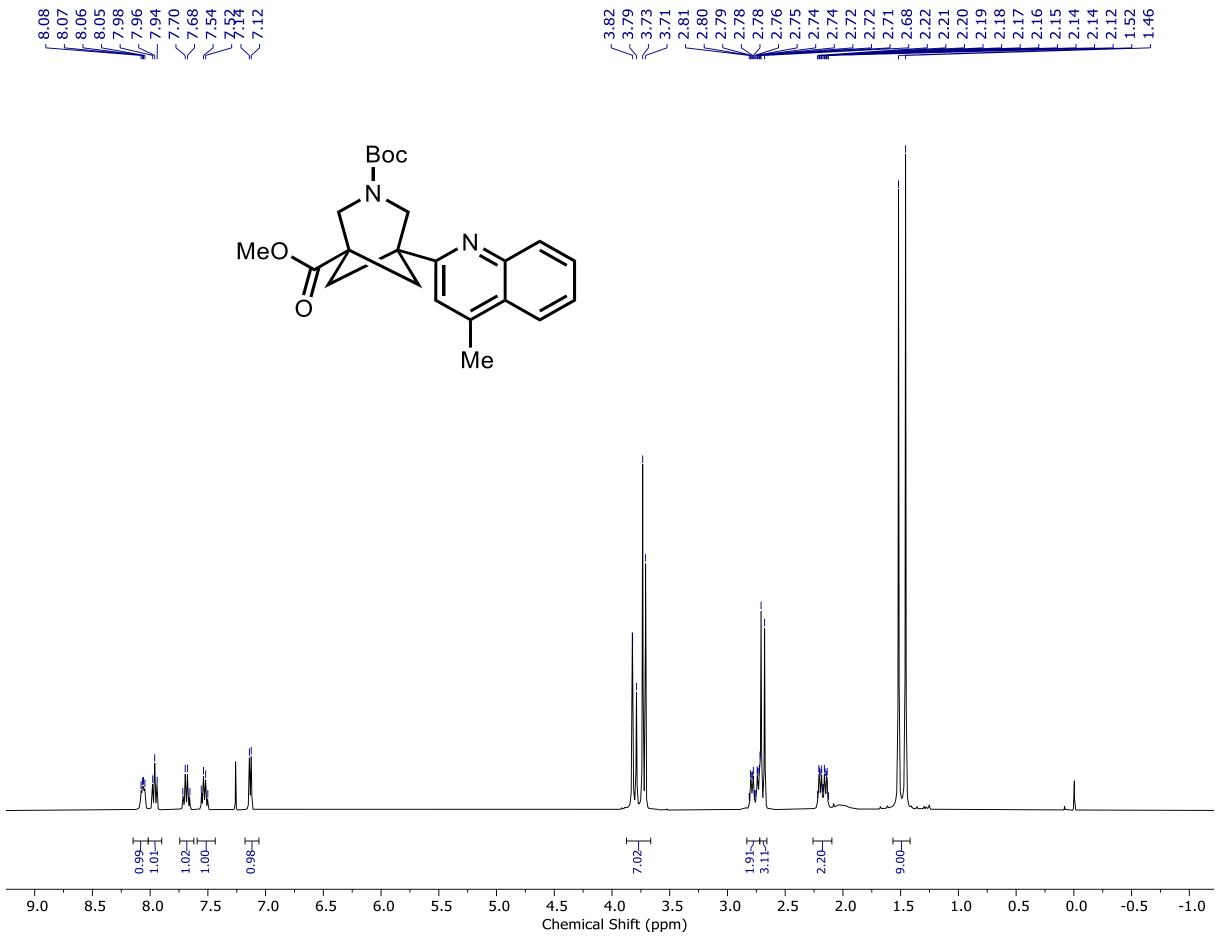

3-(*tert*-Butyl) 1-methyl 5-(4-methylquinolin-2-yl)-3-azabicyclo[3.1.1]heptane-1,3-dicarboxylate **(7d)** **^{1}H NMR (400 MHz, DMSO-d6, 90°C)**

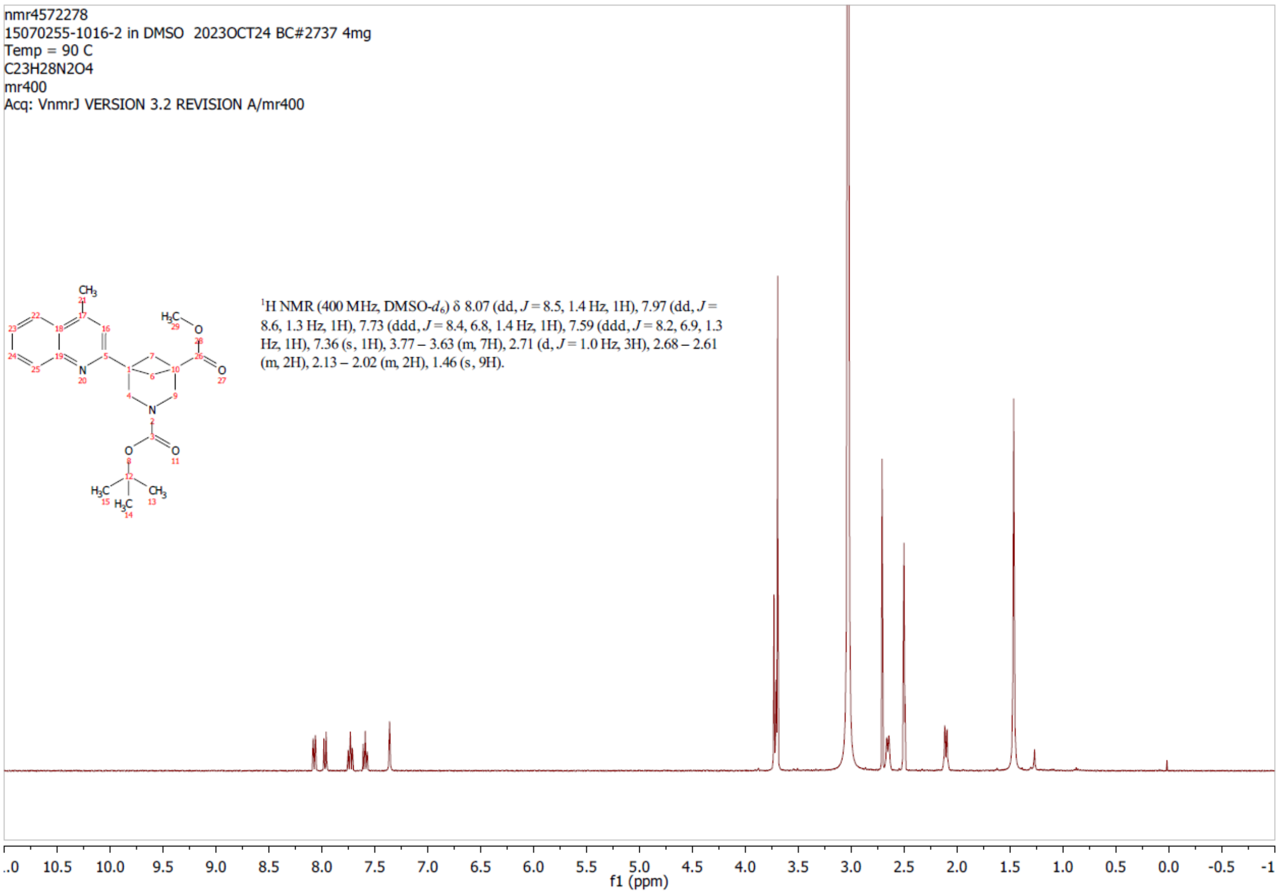

3-(*tert*-Butyl) 1-methyl 5-(4-methylquinolin-2-yl)-3-azabicyclo[3.1.1]heptane-1,3-dicarboxylate **(7d)** **^{13}C NMR (101 MHz, $CDCl_3$)**

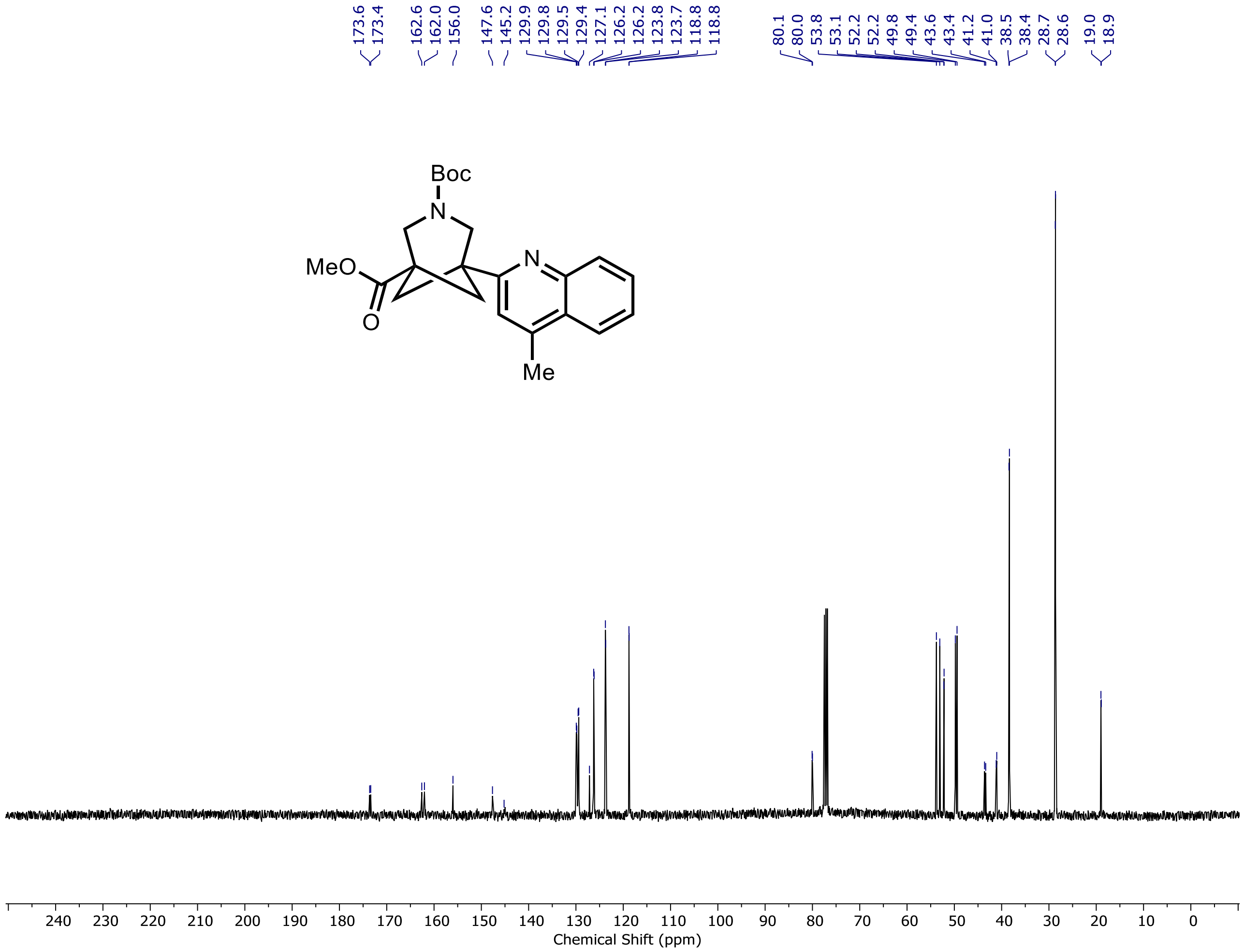

References

(1) Frank, N.; Nugent, J.; Shire, B. R.; Pickford, H. D.; Rabe, P.; Sterling, A. J.; Zarganes-Tzitzikas, T.; Grimes, T.; Thompson, A. L.; Smith, R. C.; et al. Synthesis of meta-substituted arene bioisosteres from [3.1.1]propellane. *Nature* **2022**, *611* (7937), 721-726. DOI: 10.1038/s41586-022-05290-z.
(2) Hunsen, M. Carboxylic Acids from Primary Alcohols and Aldehydes by a Pyridinium Chlorochromate Catalyzed Oxidation. *Synthesis* **2005**, *2005* (15), 2487-2490. DOI: 10.1055/s-2005-872085.
(3) McNamee, R.; Frank, N.; Christensen, K.; Duarte, F.; Anderson, E. Taming non-classical carbocations to control small ring reactivity. American Chemical Society (ACS): 2023.
(4) Senda, Y.; Ishiyama, J.-I.; Imaizumi, S. ^{13}C NMR Spectra of Some Methyl Cyclohexanecarboxylates. *Bulletin of the Chemical Society of Japan* **1976**, *49* (5), 1359-1362. DOI: 10.1246/bcsj.49.1359.
(5) Philip M. Warner, B.-L. C. a. E. W. A General Approach to the Synthesis of Bridgehead-Bridgehead Disubstituted Bicyclo[*n*.1.1]alkanes. *J. Org. Chem.* **1981**, *46*, 4795-4797.
(6) Della, E.; Tsanaktsidis, J. Decarboxylation of Bridgehead Carboxylic Acids by the Barton Procedure. *Australian Journal of Chemistry* **1986**, *39* (12), 2061-2066. DOI: https://doi.org/10.1071/CH9862061.
(7) Munchhof, M. J.; Reiter, L. A.; Shavnya, A.; Jones, C. S.; Li, Q.; Linde, R. G. Benzimidazole Derivatives. 2008.
